# Supplementary material for: Proteomic blood profiling in mild, severe and critical COVID-19 patients
Source: Sci Rep. 2021 Mar 18;11:6357. doi: 10.1038/s41598-021-85877-0 (PMC7973581; doi:10.1038/s41598-021-85877-0)
Supplement: Supplementary file 1 — Supplementary Information. [file 41598_2021_85877_MOESM1_ESM.pdf]

Proteomic blood profiling in mild, severe and critical COVID-19 patients

Hamel Patel, Nicholas J Ashton, Richard J.B Dobson, Lars-Magnus Andersson, Aylin Yilmaz , Kaj Blennow, Magnus Gisslen, Henrik Zetterberg

Supplementary Table 1: Significance testing of age, gender and “days since symptom onset” between disease groups

	**control vs mild**	**control vs severe**	**control vs critical**	**mild vs severe**	**mild vs critical**	**severe vs critical**	**control vs case**
**age**	0.0098	0.758	0.615	0.017	0.008736	0.442	0.146
**gender**	1	0.7023	0.004729	0.4605	0.001765	0.111	0.1596
**days since infection onset**	NA	NA	NA	0.00005406	0.00001604	0.434	NA

Supplementary Table 2: Differential expression results

**Comparison**	**Assay**	**OLINK.ID**	**Protein.ID**	**Uniprot.ID**	**logFC**	**CI.L**	**CI.R**	**AveExpr**	**P.Value**	**adj.P.Val**
Control_v_Case	Immune	OID00981	NF2	P35240	-4.62	-4.70	-4.53	0.91	0.00	0.00
Control_v_Case	Immune	OID01022	KPNA1	P52294	-1.49	-1.56	-1.42	0.26	0.00	0.00
Control_v_Mild	Immune	OID00981	NF2	P35240	-4.62	-4.80	-4.44	1.77	0.00	0.00
Control_v_Critical	Immune	OID00981	NF2	P35240	-4.44	-4.67	-4.22	1.87	0.00	0.00
Control_v_Case	Immune	OID01011	DAPP1	Q9UN19	-6.55	-7.13	-5.97	4.72	0.00	0.00
Control_v_Case	Immune	OID00936	PPP1R9B	Q96SB3	-4.86	-5.29	-4.43	3.31	0.00	0.00
Control_v_Case	Immune	OID00959	ITGA6	P23229	-4.49	-4.94	-4.05	2.17	0.00	0.00
Control_v_Case	Immune	OID00998	SPRY2	O43597	-5.06	-5.58	-4.55	3.81	0.00	0.00
Control_v_Mild	Neurology	OID00374	MANF	P55145	-2.85	-3.08	-2.63	7.97	0.00	0.00
Control_v_Case	Inflammation	OID00487	AXIN1	O15169	-4.86	-5.41	-4.31	4.27	0.00	0.00
Control_v_Case	Inflammation	OID00557	ST1A1	P50225	-4.67	-5.21	-4.12	3.21	0.00	0.00
Control_v_Case	Immune	OID00940	IRAK4	Q9NWZ3	-4.61	-5.15	-4.06	3.51	0.00	0.00
Control_v_Case	Immune	OID00994	SRPK2	P78362	-4.05	-4.55	-3.54	2.49	0.00	0.00
Control_v_Case	Immune	OID00958	DCTN1	Q14203	-4.12	-4.64	-3.60	4.69	0.00	0.00
Control_v_Mild	Immune	OID00979	BIRC2	Q13490	-1.74	-1.90	-1.57	1.33	0.00	0.00
Control_v_Case	Immune	OID00954	FGF2	P09038	-2.99	-3.38	-2.60	1.57	0.00	0.00
Control_v_Mild	Cardiovascular	OID00401	PDGF subunit B	P01127	-3.23	-3.56	-2.90	10.31	0.00	0.00
Control_v_Case	Inflammation	OID00558	STAMBP	O95630	-3.15	-3.58	-2.72	5.85	0.00	0.00
Control_v_Mild	Immune	OID01011	DAPP1	Q9UN19	-6.62	-7.31	-5.92	5.97	0.00	0.00

Control_v_Case	Inflammation	OID00535	CXCL10	P02778	4.39	3.76	5.01	11.20	0.00	0.00
Control_v_Case	Neurology	OID00371	LAT	O43561	-3.91	-4.46	-3.35	7.13	0.00	0.00
Control_v_Case	Cardiovascular	OID00443	ITGB1BP2	Q9UKP3	-4.88	-5.58	-4.18	4.59	0.00	0.00
Control_v_Mild	Immune	OID00936	PPP1R9B	Q96SB3	-5.28	-5.86	-4.69	4.17	0.00	0.00
Control_v_Mild	Immune	OID00994	SRPK2	P78362	-5.25	-5.84	-4.67	3.05	0.00	0.00
Control_v_Case	Immune	OID00989	PRKCQ	Q04759	-1.25	-1.43	-1.07	1.15	0.00	0.00
Control_v_Case	Inflammation	OID00528	IL10	P22301	2.89	2.47	3.31	4.33	0.00	0.00
Control_v_Mild	Immune	OID00962	FXYD5	Q96DB9	-1.97	-2.20	-1.75	1.32	0.00	0.00
Control_v_Case	Immune	OID01003	ICA1	Q05084	-2.44	-2.80	-2.08	1.92	0.00	0.00
Control_v_Case	Immune	OID00979	BIRC2	Q13490	-1.42	-1.63	-1.21	1.09	0.00	0.00
Control_v_Case	Immune	OID00946	EDAR	Q9UNE0	-2.95	-3.39	-2.52	2.53	0.00	0.00
Control_v_Mild	Immune	OID00976	EIF4G1	Q04637	-4.59	-5.14	-4.05	5.27	0.00	0.00
Control_v_Case	Immune	OID01015	LAMP3	Q9UQV4	2.32	1.97	2.68	5.01	0.00	0.00
Control_v_Case	Inflammation	OID00538	SIRT2	Q8IXJ6	-3.85	-4.45	-3.25	5.45	0.00	0.00
Control_v_Case	Immune	OID00999	STC1	P52823	1.81	1.53	2.09	6.62	0.00	0.00
Control_v_Critical	Immune	OID01011	DAPP1	Q9UN19	-6.82	-7.62	-6.03	6.07	0.00	0.00
Control_v_Case	Inflammation	OID00562	CSF-1	P09603	0.88	0.74	1.02	10.37	0.00	0.00
Control_v_Critical	Inflammation	OID00535	CXCL10	P02778	4.14	3.63	4.65	11.51	0.00	0.00
Control_v_Case	Immune	OID00947	IL6	P05231	5.14	4.32	5.97	3.93	0.00	0.00
Control_v_Case	Neurology	OID00315	SIGLEC1	Q9BZZ2	2.08	1.74	2.41	6.48	0.00	0.00
Control_v_Case	Immune	OID00983	SH2B3	Q9UQQ2	-4.04	-4.70	-3.38	4.14	0.00	0.00
Control_v_Case	Inflammation	OID00482	IL6	P05231	5.44	4.54	6.34	4.10	0.00	0.00
Control_v_Case	Cardiovascular	OID00424	DECR1	Q16698	-3.57	-4.16	-2.98	5.13	0.00	0.00
Control_v_Case	Immune	OID00962	FXYD5	Q96DB9	-1.59	-1.86	-1.33	1.08	0.00	0.00
Control_v_Mild	Immune	OID01022	KPNA1	P52294	-1.49	-1.69	-1.29	0.49	0.00	0.00
Control_v_Mild	Cardiovascular	OID00392	STK4	Q13043	-3.41	-3.88	-2.94	4.51	0.00	0.00
Control_v_Mild	Immune	OID00958	DCTN1	Q14203	-4.44	-5.05	-3.84	5.33	0.00	0.00
Control_v_Case	Cardiovascular	OID00406	Gal-9	O00182	1.72	1.43	2.01	8.51	0.00	0.00
Control_v_Critical	Immune	OID00936	PPP1R9B	Q96SB3	-4.81	-5.44	-4.17	4.45	0.00	0.00
Control_v_Case	Neurology	OID00306	PLXNB3	Q9ULL4	-1.75	-2.05	-1.45	4.44	0.00	0.00
Control_v_Case	Inflammation	OID00474	MCP-3	P80098	4.53	3.76	5.31	3.26	0.00	0.00
Control_v_Case	Immune	OID00948	DGKZ	Q13574	-0.92	-1.07	-0.76	0.87	0.00	0.00
Control_v_Case	Immune	OID00950	IRAK1	P51617	-2.39	-2.79	-1.98	2.96	0.00	0.00
Control_v_Mild	Immune	OID00953	PRDX3	P30048	-2.93	-3.34	-2.53	0.98	0.00	0.00

Control_v_Mild	Immune	OID00940	IRAK4	Q9NWZ3	-5.03	-5.73	-4.33	4.33	0.00	0.00
Control_v_Mild	Neurology	OID00371	LAT	O43561	-5.61	-6.40	-4.82	7.55	0.00	0.00
Control_v_Critical	Cardiovascular	OID00443	ITGB1BP2	Q9UKP3	-5.50	-6.25	-4.75	5.76	0.00	0.00
Control_v_Mild	Inflammation	OID00487	AXIN1	O15169	-5.24	-6.00	-4.48	5.08	0.00	0.00
Control_v_Case	Cardiovascular	OID00392	STK4	Q13043	-3.05	-3.59	-2.51	4.04	0.00	0.00
Control_v_Mild	Immune	OID00987	HEXIM1	O94992	-4.67	-5.35	-3.99	6.26	0.00	0.00
Control_v_Case	Immune	OID00953	PRDX3	P30048	-2.18	-2.57	-1.79	0.67	0.00	0.00
Control_v_Critical	Inflammation	OID00487	AXIN1	O15169	-5.11	-5.84	-4.38	5.40	0.00	0.00
Control_v_Critical	Neurology	OID00374	MANF	P55145	-1.81	-2.07	-1.55	8.30	0.00	0.00
Control_v_Case	Cardiovascular	OID00390	IL6	P05231	5.60	4.59	6.62	4.92	0.00	0.00
Control_v_Mild	Immune	OID00948	DGKZ	Q13574	-0.91	-1.05	-0.77	0.98	0.00	0.00
Control_v_Case	Immune	OID00939	ZBTB16	Q05516	-2.65	-3.13	-2.16	2.15	0.00	0.00
Control_v_Case	Immune	OID00993	IL10	P22301	2.86	2.32	3.39	4.53	0.00	0.00
Control_v_Case	Immune	OID00937	GLB1	P16278	-2.25	-2.67	-1.83	1.97	0.00	0.00
Control_v_Mild	Inflammation	OID00538	SIRT2	Q8IXJ6	-5.24	-6.06	-4.42	5.98	0.00	0.00
Control_v_Mild	Inflammation	OID00557	ST1A1	P50225	-5.25	-6.08	-4.41	4.01	0.00	0.00
Control_v_Case	Cardiovascular	OID00420	CD84	Q9UIB8	-1.46	-1.74	-1.18	5.02	0.00	0.00
Control_v_Severe	Neurology	OID00374	MANF	P55145	-4.71	-5.26	-4.16	8.58	0.00	0.00
Control_v_Case	Immune	OID00990	MGMT	P16455	-3.46	-4.11	-2.80	4.98	0.00	0.00
Control_v_Case	Inflammation	OID00550	CASP-8	Q14790	-2.52	-3.00	-2.04	4.43	0.00	0.00
Control_v_Critical	Immune	OID00958	DCTN1	Q14203	-4.18	-4.83	-3.53	5.78	0.00	0.00
Control_v_Case	Immune	OID01023	LAG3	P18627	1.41	1.14	1.68	2.96	0.00	0.00
Control_v_Case	Inflammation	OID05547	IFN-gamma	P01579	4.43	3.57	5.29	8.21	0.00	0.00
Control_v_Mild	Immune	OID00957	TRIM5	Q9C035	-2.44	-2.83	-2.04	2.73	0.00	0.00
Control_v_Case	Inflammation	OID00518	PD-L1	Q9NZQ7	1.61	1.29	1.92	6.00	0.00	0.00
Control_v_Critical	Inflammation	OID00528	IL10	P22301	3.41	2.86	3.96	4.47	0.00	0.00
Control_v_Critical	Neurology	OID00371	LAT	O43561	-4.15	-4.82	-3.48	8.35	0.00	0.00
Control_v_Mild	Immune	OID00954	FGF2	P09038	-3.15	-3.67	-2.62	2.11	0.00	0.00
Control_v_Critical	Immune	OID00993	IL10	P22301	3.42	2.86	3.98	4.70	0.00	0.00
Control_v_Mild	Immune	OID00955	PRDX5	P30044	-4.03	-4.71	-3.36	5.80	0.00	0.00
Control_v_Case	Cardiovascular	OID00437	PTX3	P26022	2.16	1.73	2.59	4.54	0.00	0.00
Control_v_Case	Cardiovascular	OID00389	IL-1ra	P18510	2.95	2.37	3.54	6.01	0.00	0.00
Control_v_Mild	Immune	OID00952	PRDX1	Q06830	-3.96	-4.63	-3.30	3.58	0.00	0.00
Control_v_Case	Immune	OID00976	EIF4G1	Q04637	-3.30	-3.96	-2.64	4.87	0.00	0.00

Control_v_Case	Immune	OID00965	LILRB4	Q8NHJ6	2.42	1.94	2.91	4.10	0.00	0.00
Control_v_Mild	Immune	OID00998	SPRY2	O43597	-5.22	-6.11	-4.33	4.79	0.00	0.00
Control_v_Case	Immune	OID00942	HCLS1	P14317	-2.86	-3.43	-2.28	5.87	0.00	0.00
Control_v_Case	Immune	OID00951	CLEC4A	Q9UMR7	-1.30	-1.57	-1.04	3.57	0.00	0.00
Control_v_Mild	Cardiovascular	OID00420	CD84	Q9UIB8	-1.74	-2.04	-1.43	5.24	0.00	0.00
Control_v_Case	Immune	OID01020	TANK	Q92844	-2.35	-2.84	-1.87	2.10	0.00	0.00
Control_v_Case	Inflammation	OID00484	MCP-1	P13500	2.01	1.60	2.43	11.95	0.00	0.00
Control_v_Mild	Immune	OID01003	ICA1	Q05084	-2.84	-3.35	-2.34	2.34	0.00	0.00
Control_v_Mild	Immune	OID00950	IRAK1	P51617	-3.04	-3.58	-2.50	3.28	0.00	0.00
Control_v_Mild	Immune	OID00939	ZBTB16	Q05516	-3.45	-4.06	-2.83	2.50	0.00	0.00
Control_v_Mild	Cardiovascular	OID00467	NEMO	Q9Y6K9	-3.88	-4.58	-3.17	6.38	0.00	0.00
Control_v_Case	Neurology	OID00323	LXN	Q9BS40	-1.03	-1.25	-0.82	2.06	0.00	0.00
Control_v_Critical	Immune	OID00998	SPRY2	O43597	-5.01	-5.88	-4.13	4.80	0.00	0.00
Control_v_Critical	Inflammation	OID00557	ST1A1	P50225	-4.39	-5.16	-3.62	4.35	0.00	0.00
Control_v_Critical	Inflammation	OID00558	STAMBP	O95630	-3.05	-3.58	-2.51	6.74	0.00	0.00
Control_v_Case	Immune	OID00967	KRT19	P08727	4.00	3.16	4.84	3.39	0.00	0.00
Control_v_Mild	Immune	OID00942	HCLS1	P14317	-4.38	-5.18	-3.58	6.15	0.00	0.00
Control_v_Case	Cardiovascular	OID00439	CCL17	Q92583	-3.45	-4.19	-2.71	9.17	0.00	0.00
Control_v_Case	Immune	OID01014	MASP1	P48740	-1.06	-1.29	-0.83	1.84	0.00	0.00
Control_v_Critical	Immune	OID00959	ITGA6	P23229	-4.29	-5.06	-3.51	2.94	0.00	0.00
Control_v_Case	Cardiovascular	OID00459	CTSL1	P07711	2.29	1.80	2.79	8.11	0.00	0.00
Control_v_Case	Immune	OID00985	CKAP4	Q07065	2.11	1.65	2.57	5.42	0.00	0.00
Control_v_Case	Cardiovascular	OID00395	PAR-1	P25116	-1.20	-1.46	-0.94	8.99	0.00	0.00
Control_v_Mild	Neurology	OID00318	CLEC1B	Q9P126	-3.28	-3.90	-2.66	11.01	0.00	0.00
Control_v_Mild	Immune	OID00959	ITGA6	P23229	-4.46	-5.32	-3.61	3.08	0.00	0.00
Mild_v_Critical	Cardiovascular	OID00459	CTSL1	P07711	1.84	1.53	2.15	8.45	0.00	0.00
Control_v_Critical	Neurology	OID00315	SIGLEC1	Q9BZZ2	1.93	1.57	2.29	6.52	0.00	0.00
Control_v_Case	Cardiovascular	OID00467	NEMO	Q9Y6K9	-2.92	-3.57	-2.27	6.03	0.00	0.00
Control_v_Case	Neurology	OID00357	CDH6	P55285	-1.16	-1.42	-0.90	4.33	0.00	0.00
Control_v_Case	Neurology	OID00344	PVR	P15151	0.98	0.76	1.20	8.45	0.00	0.00
Control_v_Case	Neurology	OID00333	GFR-alpha-1	P56159	1.33	1.03	1.63	7.33	0.00	0.00
Control_v_Critical	Immune	OID00940	IRAK4	Q9NWZ3	-4.72	-5.63	-3.82	4.59	0.00	0.00
Control_v_Critical	Immune	OID00976	EIF4G1	Q04637	-3.33	-3.97	-2.69	6.01	0.00	0.00
Control_v_Mild	Immune	OID00937	GLB1	P16278	-2.95	-3.53	-2.37	2.33	0.00	0.00

Mild_v_Critical	Inflammation	OID00474	MCP-3	P80098	3.95	3.25	4.65	3.80	0.00	0.00
Control_v_Case	Cardiovascular	OID00412	RAGE	Q15109	1.56	1.21	1.91	13.61	0.00	0.00
Mild_v_Critical	Immune	OID00947	IL6	P05231	4.42	3.63	5.22	4.60	0.00	0.00
Mild_v_Critical	Immune	OID00979	BIRC2	Q13490	0.32	0.26	0.38	0.57	0.00	0.00
Control_v_Case	Inflammation	OID00517	IL-18R1	Q13478	1.54	1.19	1.90	8.85	0.00	0.00
Mild_v_Critical	Inflammation	OID00482	IL6	P05231	4.46	3.64	5.28	4.75	0.00	0.00
Control_v_Severe	Cardiovascular	OID00392	STK4	Q13043	-4.46	-5.16	-3.77	5.44	0.00	0.00
Control_v_Case	Immune	OID00955	PRDX5	P30044	-2.43	-2.99	-1.88	5.54	0.00	0.00
Control_v_Critical	Neurology	OID00348	CPM	P14384	-1.23	-1.47	-0.99	6.50	0.00	0.00
Control_v_Mild	Inflammation	OID00520	CXCL5	P42830	-3.65	-4.39	-2.91	11.70	0.00	0.00
Control_v_Mild	Neurology	OID00324	gal-8	O00214	-2.65	-3.19	-2.12	7.15	0.00	0.00
Control_v_Mild	Immune	OID00983	SH2B3	Q9UQQ2	-4.33	-5.20	-3.45	4.84	0.00	0.00
Control_v_Case	Neurology	OID00300	SCARB2	Q14108	1.37	1.05	1.68	5.01	0.00	0.00
Control_v_Case	Neurology	OID00309	SMPD1	P17405	1.06	0.81	1.30	4.76	0.00	0.00
Control_v_Critical	Inflammation	OID00503	TGF-alpha	P01135	1.50	1.20	1.80	3.34	0.00	0.00
Control_v_Case	Cardiovascular	OID00464	CA5A	P35218	2.89	2.21	3.57	3.30	0.00	0.00
Control_v_Case	Immune	OID00963	TRAF2	Q12933	-1.43	-1.77	-1.10	2.26	0.00	0.00
Control_v_Case	Cardiovascular	OID00402	IL-27	Q8NEV9, Q14213	1.34	1.02	1.66	6.29	0.00	0.00
Control_v_Case	Inflammation	OID00521	TRANCE	O14788	-2.14	-2.66	-1.63	3.99	0.00	0.00
Control_v_Case	Inflammation	OID00555	TWEAK	O43508	-1.27	-1.57	-0.96	8.63	0.00	0.00
Control_v_Case	Immune	OID00987	HEXIM1	O94992	-2.77	-3.44	-2.11	6.02	0.00	0.00
Control_v_Mild	Cardiovascular	OID00424	DECR1	Q16698	-4.43	-5.37	-3.48	5.66	0.00	0.00
Control_v_Mild	Cardiovascular	OID00395	PAR-1	P25116	-1.42	-1.73	-1.12	9.18	0.00	0.00
Control_v_Critical	Inflammation	OID00538	SIRT2	Q8IXJ6	-3.93	-4.75	-3.11	6.71	0.00	0.00
Control_v_Critical	Immune	OID01022	KPNA1	P52294	-1.49	-1.80	-1.18	0.61	0.00	0.00
Control_v_Mild	Immune	OID00990	MGMT	P16455	-4.20	-5.09	-3.30	5.45	0.00	0.00
Control_v_Mild	Immune	OID01023	LAG3	P18627	1.37	1.08	1.66	2.70	0.00	0.00
Control_v_Critical	Immune	OID00942	HCLS1	P14317	-2.62	-3.16	-2.07	6.88	0.00	0.00
Control_v_Case	Neurology	OID00324	gal-8	O00214	-1.76	-2.19	-1.33	6.94	0.00	0.00
Control_v_Critical	Cardiovascular	OID00392	STK4	Q13043	-3.43	-4.16	-2.71	4.79	0.00	0.00
Control_v_Critical	Immune	OID00999	STC1	P52823	1.65	1.30	1.99	6.37	0.00	0.00
Control_v_Critical	Neurology	OID00296	EZR	P15311	1.41	1.11	1.71	4.35	0.00	0.00
Control_v_Case	Inflammation	OID00520	CXCL5	P42830	-2.63	-3.28	-1.98	11.22	0.00	0.00
Control_v_Critical	Neurology	OID00306	PLXNB3	Q9ULL4	-1.82	-2.21	-1.42	4.80	0.00	0.00

Control_v_Case	Neurology	OID00296	EZR	P15311	1.17	0.88	1.47	4.18	0.00	0.00
Control_v_Case	Cardiovascular	OID00441	MMP7	P09237	1.50	1.13	1.88	9.26	0.00	0.00
Mild_v_Critical	Inflammation	OID00541	EN-RAGE	P80511	2.84	2.26	3.43	3.80	0.00	0.00
Mild_v_Critical	Immune	OID00985	CKAP4	Q07065	1.60	1.27	1.92	5.77	0.00	0.00
Control_v_Critical	Cardiovascular	OID00412	RAGE	Q15109	2.32	1.81	2.83	13.65	0.00	0.00
Control_v_Mild	Inflammation	OID00558	STAMBP	O95630	-3.77	-4.62	-2.91	6.35	0.00	0.00
Control_v_Case	Immune	OID00952	PRDX1	Q06830	-2.37	-2.97	-1.77	3.32	0.00	0.00
Control_v_Case	Cardiovascular	OID00386	BOC	Q9BWV1	-1.23	-1.55	-0.92	3.40	0.00	0.00
Control_v_Critical	Cardiovascular	OID00459	CTSL1	P07711	2.09	1.63	2.56	8.26	0.00	0.00
Control_v_Critical	Cardiovascular	OID00406	Gal-9	O00182	1.60	1.24	1.96	8.60	0.00	0.00
Control_v_Mild	Cardiovascular	OID00445	Dkk-1	O94907	-2.23	-2.74	-1.72	9.00	0.00	0.00
Control_v_Case	Immune	OID01017	CLEC6A	Q6EIG7	1.60	1.19	2.01	2.51	0.00	0.00
Control_v_Case	Inflammation	OID00541	EN-RAGE	P80511	2.37	1.76	2.98	3.46	0.00	0.00
Control_v_Mild	Immune	OID00989	PRKCQ	Q04759	-1.21	-1.49	-0.93	1.38	0.00	0.00
Control_v_Case	Inflammation	OID00503	TGF-alpha	P01135	1.27	0.94	1.60	3.22	0.00	0.00
Control_v_Critical	Neurology	OID00324	gal-8	O00214	-1.90	-2.33	-1.47	7.69	0.00	0.00
Control_v_Case	Neurology	OID00338	NTRK2	Q16620	-0.96	-1.20	-0.71	6.18	0.00	0.00
Control_v_Mild	Inflammation	OID00550	CASP-8	Q14790	-3.54	-4.36	-2.72	4.76	0.00	0.00
Control_v_Critical	Inflammation	OID05547	IFN-gamma	P01579	5.37	4.15	6.60	8.55	0.00	0.00
Control_v_Critical	Neurology	OID00320	MATN3	O15232	1.90	1.47	2.34	9.74	0.00	0.00
Control_v_Mild	Cardiovascular	OID00443	ITGB1BP2	Q9UKP3	-5.09	-6.28	-3.90	5.42	0.00	0.00
Control_v_Critical	Inflammation	OID00474	MCP-3	P80098	4.22	3.25	5.18	3.82	0.00	0.00
Control_v_Case	Cardiovascular	OID00425	MERTK	Q12866	0.86	0.64	1.09	6.65	0.00	0.00
Mild_v_Critical	Cardiovascular	OID00390	IL6	P05231	4.48	3.51	5.45	5.60	0.00	0.00
Control_v_Case	Inflammation	OID05548	TNF	P01375	1.33	0.98	1.67	3.29	0.00	0.00
Mild_v_Critical	Cardiovascular	OID00396	TRAIL-R2	O14763	2.01	1.57	2.44	6.91	0.00	0.00
Control_v_Critical	Immune	OID00954	FGF2	P09038	-3.21	-3.96	-2.46	2.28	0.00	0.00
Control_v_Critical	Cardiovascular	OID00390	IL6	P05231	4.79	3.67	5.92	5.53	0.00	0.00
Control_v_Critical	Inflammation	OID00482	IL6	P05231	4.49	3.43	5.55	4.71	0.00	0.00
Control_v_Severe	Immune	OID01011	DAPP1	Q9UN19	-8.42	-10.04	-6.81	7.37	0.00	0.00
Mild_v_Critical	Immune	OID00967	KRT19	P08727	3.44	2.68	4.19	3.82	0.00	0.00
Control_v_Case	Inflammation	OID00524	IL-24	Q13007	0.99	0.73	1.26	1.24	0.00	0.00
Control_v_Case	Immune	OID01009	AREG	P15514	2.36	1.73	2.99	4.17	0.00	0.00
Control_v_Case	Cardiovascular	OID00391	TNFRSF10A	O00220	1.53	1.12	1.94	3.35	0.00	0.00

Control_v_Mild	Immune	OID01020	TANK	Q92844	-2.60	-3.22	-1.97	2.47	0.00	0.00
Control_v_Critical	Immune	OID00947	IL6	P05231	4.23	3.22	5.24	4.51	0.00	0.00
Control_v_Case	Neurology	OID00320	MATN3	O15232	1.66	1.21	2.10	9.52	0.00	0.00
Control_v_Case	Inflammation	OID00552	CX3CL1	P78423	1.07	0.78	1.37	4.36	0.00	0.00
Control_v_Mild	Neurology	OID00323	LXN	Q9BS40	-1.13	-1.40	-0.85	2.25	0.00	0.00
Control_v_Critical	Cardiovascular	OID00437	PTX3	P26022	2.07	1.57	2.58	4.64	0.00	0.00
Control_v_Case	Cardiovascular	OID00382	CD40-L	P29965	-3.12	-3.99	-2.26	5.25	0.00	0.00
Control_v_Severe	Immune	OID00936	PPP1R9B	Q96SB3	-6.67	-8.03	-5.31	5.38	0.00	0.00
Control_v_Critical	Inflammation	OID00550	CASP-8	Q14790	-1.85	-2.31	-1.39	5.50	0.00	0.00
Control_v_Case	Immune	OID00982	PLXNA4	Q9HCM2	-2.38	-3.03	-1.72	4.31	0.00	0.00
Control_v_Critical	Immune	OID00990	MGMT	P16455	-3.82	-4.76	-2.87	5.95	0.00	0.00
Control_v_Critical	Neurology	OID00300	SCARB2	Q14108	1.70	1.28	2.12	5.11	0.00	0.00
Control_v_Case	Neurology	OID00374	MANF	P55145	-1.63	-2.09	-1.17	7.78	0.00	0.00
Control_v_Critical	Neurology	OID00340	G-CSF	P09919	2.70	2.01	3.38	3.52	0.00	0.00
Control_v_Critical	Cardiovascular	OID00425	MERTK	Q12866	1.10	0.82	1.38	6.70	0.00	0.00
Mild_v_Critical	Inflammation	OID00506	TNFSF14	O43557	1.85	1.41	2.30	5.20	0.00	0.00
Mild_v_Critical	Cardiovascular	OID00406	Gal-9	O00182	0.80	0.61	0.99	8.70	0.00	0.00
Control_v_Case	Immune	OID01004	DFFA	O00273	-1.79	-2.30	-1.28	5.50	0.00	0.00
Control_v_Mild	Inflammation	OID00534	CXCL6	P80162	-3.10	-3.90	-2.30	8.93	0.00	0.00
Control_v_Critical	Immune	OID00994	SRPK2	P78362	-3.62	-4.55	-2.69	3.74	0.00	0.00
Control_v_Case	Cardiovascular	OID00449	HB-EGF	Q99075	-2.09	-2.69	-1.49	6.53	0.00	0.00
Mild_v_Critical	Cardiovascular	OID00389	IL-1ra	P18510	1.86	1.41	2.32	6.41	0.00	0.00
Control_v_Case	Immune	OID00957	TRIM5	Q9C035	-1.50	-1.94	-1.07	2.61	0.00	0.00
Control_v_Case	Neurology	OID00348	CPM	P14384	-0.77	-0.99	-0.55	6.51	0.00	0.00
Control_v_Critical	Immune	OID01023	LAG3	P18627	1.61	1.20	2.03	2.91	0.00	0.00
Mild_v_Critical	Inflammation	OID00500	SCF	P21583	-1.43	-1.78	-1.08	8.68	0.00	0.00
Control_v_Critical	Immune	OID00967	KRT19	P08727	3.25	2.40	4.10	3.85	0.00	0.00
Control_v_Critical	Neurology	OID00309	SMPD1	P17405	1.14	0.84	1.43	4.80	0.00	0.00
Control_v_Critical	Neurology	OID00357	CDH6	P55285	-0.92	-1.17	-0.68	4.36	0.00	0.00
Control_v_Case	Neurology	OID00360	CTSS	P25774	0.73	0.52	0.95	5.82	0.00	0.00
Mild_v_Severe	Inflammation	OID00541	EN-RAGE	P80511	2.16	1.73	2.59	2.93	0.00	0.00
Control_v_Critical	Cardiovascular	OID00424	DECR1	Q16698	-3.49	-4.42	-2.57	6.29	0.00	0.00
Control_v_Mild	Immune	OID00963	TRAF2	Q12933	-1.90	-2.40	-1.39	2.48	0.00	0.00
Control_v_Critical	Immune	OID00987	HEXIM1	O94992	-2.48	-3.14	-1.82	7.21	0.00	0.00

Control_v_Critical	Immune	OID01015	LAMP3	Q9UQV4	2.03	1.49	2.57	5.08	0.00	0.00
Mild_v_Critical	Immune	OID01009	AREG	P15514	2.40	1.80	3.00	4.47	0.00	0.00
Control_v_Case	Neurology	OID00372	NTRK3	Q16288	-0.86	-1.12	-0.61	7.13	0.00	0.00
Control_v_Critical	Immune	OID00983	SH2B3	Q9UQQ2	-4.28	-5.43	-3.14	4.98	0.00	0.00
Control_v_Case	Neurology	OID00307	CPA2	P48052	-1.77	-2.30	-1.25	9.68	0.00	0.00
Control_v_Case	Neurology	OID00303	ROBO2	Q9HCK4	-1.24	-1.61	-0.87	5.24	0.00	0.00
Control_v_Case	Cardiovascular	OID00396	TRAIL-R2	O14763	1.35	0.95	1.76	6.67	0.00	0.00
Control_v_Case	Neurology	OID00305	RGMA	Q96B86	-0.88	-1.14	-0.62	10.66	0.00	0.00
Control_v_Case	Neurology	OID00317	ADAM 22	Q9P0K1	-0.96	-1.24	-0.67	4.16	0.00	0.00
Control_v_Case	Neurology	OID00342	SCARF2	Q96GP6	-0.96	-1.25	-0.67	6.03	0.00	0.00
Control_v_Case	Immune	OID01019	IL12RB1	P42701	0.93	0.65	1.22	2.52	0.00	0.00
Control_v_Case	Cardiovascular	OID00381	ADM	P35318	1.14	0.79	1.48	8.63	0.00	0.00
Control_v_Case	Neurology	OID00359	JAM-B	P57087	-0.81	-1.05	-0.56	7.80	0.00	0.00
Control_v_Case	Neurology	OID00343	GDNFR-alpha-3	O60609	-1.02	-1.33	-0.71	4.74	0.00	0.00
Control_v_Severe	Cardiovascular	OID00395	PAR-1	P25116	-3.34	-4.10	-2.58	9.52	0.00	0.00
Mild_v_Critical	Inflammation	OID00503	TGF-alpha	P01135	1.24	0.92	1.57	3.51	0.00	0.00
Mild_v_Critical	Neurology	OID00344	PVR	P15151	0.72	0.53	0.91	8.59	0.00	0.00
Mild_v_Critical	Cardiovascular	OID00391	TNFRSF10A	O00220	1.46	1.08	1.84	3.53	0.00	0.00
Control_v_Case	Cardiovascular	OID00416	SPON2	Q9BUD6	0.48	0.33	0.62	8.43	0.00	0.00
Control_v_Mild	Neurology	OID00306	PLXNB3	Q9ULL4	-1.86	-2.38	-1.34	4.74	0.00	0.00
Control_v_Critical	Cardiovascular	OID00388	SRC	P12931	-2.32	-2.97	-1.67	6.76	0.00	0.00
Control_v_Critical	Inflammation	OID00562	CSF-1	P09603	0.72	0.52	0.93	10.43	0.00	0.00
Control_v_Case	Inflammation	OID00511	LIF-R	P42702	0.65	0.45	0.85	4.28	0.00	0.00
Control_v_Case	Inflammation	OID00513	CCL19	Q99731	1.95	1.34	2.56	10.04	0.00	0.00
Control_v_Case	Inflammation	OID00534	CXCL6	P80162	-2.02	-2.65	-1.39	8.76	0.00	0.00
Control_v_Critical	Immune	OID00985	CKAP4	Q07065	1.98	1.41	2.54	5.68	0.00	0.00
Control_v_Case	Neurology	OID00328	GDF-8	O14793	-1.40	-1.85	-0.96	3.21	0.00	0.00
Control_v_Case	Inflammation	OID00479	OPG	O00300	0.90	0.62	1.19	10.40	0.00	0.00
Mild_v_Critical	Inflammation	OID00522	HGF	P14210	2.14	1.55	2.72	9.21	0.00	0.00
Control_v_Critical	Cardiovascular	OID00420	CD84	Q9UIB8	-1.31	-1.69	-0.93	5.30	0.00	0.00
Control_v_Case	Cardiovascular	OID00383	SLAMF7	Q9NQ25	1.52	1.03	2.00	3.65	0.00	0.00
Control_v_Critical	Immune	OID01003	ICA1	Q05084	-2.48	-3.21	-1.76	2.60	0.00	0.00
Control_v_Case	Neurology	OID00302	PRTG	Q2VWP7	-0.51	-0.67	-0.34	6.47	0.00	0.00
Mild_v_Critical	Cardiovascular	OID00386	BOC	Q9BWV1	-0.86	-1.11	-0.62	3.28	0.00	0.00

Control_v_Critical	Immune	OID00965	LILRB4	Q8NHJ6	1.79	1.26	2.32	4.23	0.00	0.00
Control_v_Critical	Immune	OID00950	IRAK1	P51617	-2.33	-3.01	-1.64	3.71	0.00	0.00
Control_v_Mild	Cardiovascular	OID00382	CD40-L	P29965	-4.12	-5.34	-2.90	5.66	0.00	0.00
Control_v_Case	Immune	OID00997	PIK3AP1	Q6ZUJ8	-1.66	-2.21	-1.12	3.68	0.00	0.00
Control_v_Mild	Immune	OID00996	BACH1	O14867	-1.90	-2.47	-1.33	2.00	0.00	0.00
Control_v_Case	Inflammation	OID00477	CD244	Q9BZW8	-0.62	-0.82	-0.42	5.85	0.00	0.00
Control_v_Case	Immune	OID01002	SH2D1A	O60880	-1.38	-1.84	-0.93	2.34	0.00	0.00
Control_v_Case	Cardiovascular	OID00388	SRC	P12931	-1.76	-2.34	-1.18	6.31	0.00	0.00
Control_v_Case	Immune	OID00943	CNTNAP2	Q9UHC6	-0.80	-1.07	-0.54	1.53	0.00	0.00
Control_v_Critical	Cardiovascular	OID00389	IL-1ra	P18510	2.43	1.69	3.17	6.23	0.00	0.00
Control_v_Critical	Neurology	OID00318	CLEC1B	Q9P126	-1.72	-2.24	-1.20	11.81	0.00	0.00
Mild_v_Critical	Cardiovascular	OID00408	SCF	P21583	-1.40	-1.80	-1.00	8.76	0.00	0.00
Mild_v_Critical	Inflammation	OID00479	OPG	O00300	1.06	0.76	1.37	10.52	0.00	0.00
Mild_v_Critical	Neurology	OID00296	EZR	P15311	0.80	0.57	1.03	4.29	0.00	0.00
Mild_v_Critical	Immune	OID00965	LILRB4	Q8NHJ6	1.71	1.22	2.20	4.54	0.00	0.00
Control_v_Mild	Immune	OID01004	DFFA	O00273	-3.02	-3.94	-2.10	5.65	0.00	0.00
Control_v_Case	Inflammation	OID00476	CDCP1	Q9H5V8	1.31	0.87	1.75	3.25	0.00	0.00
Control_v_Critical	Inflammation	OID00520	CXCL5	P42830	-2.73	-3.57	-1.90	11.88	0.00	0.00
Mild_v_Critical	Immune	OID00938	PSIP1	O75475	1.78	1.26	2.29	3.54	0.00	0.00
Mild_v_Critical	Neurology	OID00316	CNTN5	O94779	-1.38	-1.78	-0.98	4.17	0.00	0.00
Control_v_Mild	Immune	OID00946	EDAR	Q9UNE0	-2.87	-3.75	-1.99	3.17	0.00	0.00
Control_v_Mild	Neurology	OID00315	SIGLEC1	Q9BZZ2	1.77	1.23	2.31	6.02	0.00	0.00
Mild_v_Severe	Inflammation	OID00482	IL6	P05231	2.77	2.09	3.45	3.20	0.00	0.00
Mild_v_Severe	Cardiovascular	OID00390	IL6	P05231	2.75	2.09	3.41	4.01	0.00	0.00
Mild_v_Severe	Immune	OID00967	KRT19	P08727	2.45	1.86	3.04	2.69	0.00	0.00
Mild_v_Critical	Inflammation	OID00471	IL8	P10145	2.39	1.69	3.09	5.83	0.00	0.00
Control_v_Critical	Cardiovascular	OID00432	HO-1	P09601	1.53	1.06	2.00	11.99	0.00	0.00
Control_v_Case	Neurology	OID00339	GZMA	P12544	0.88	0.59	1.18	6.28	0.00	0.00
Mild_v_Critical	Inflammation	OID00524	IL-24	Q13007	1.03	0.73	1.34	1.37	0.00	0.00
Mild_v_Critical	Immune	OID01027	BTN3A2	P78410	1.21	0.85	1.57	3.52	0.00	0.00
Mild_v_Critical	Neurology	OID00357	CDH6	P55285	-0.68	-0.88	-0.48	4.18	0.00	0.00
Control_v_Mild	Immune	OID00982	PLXNA4	Q9HCM2	-2.90	-3.81	-2.00	4.65	0.00	0.00
Control_v_Case	Cardiovascular	OID00450	GDF-2	Q9UK05	-1.54	-2.07	-1.01	8.46	0.00	0.00
Mild_v_Severe	Immune	OID00947	IL6	P05231	2.62	1.97	3.28	3.04	0.00	0.00

Control_v_Case	Cardiovascular	OID00432	HO-1	P09601	1.16	0.76	1.55	11.91	0.00	0.00
Control_v_Mild	Immune	OID01002	SH2D1A	O60880	-2.04	-2.68	-1.40	2.49	0.00	0.00
Control_v_Case	Neurology	OID00340	G-CSF	P09919	1.32	0.87	1.78	3.25	0.00	0.00
Control_v_Severe	Neurology	OID00371	LAT	O43561	-6.22	-7.85	-4.58	9.11	0.00	0.00
Control_v_Critical	Inflammation	OID00534	CXCL6	P80162	-2.67	-3.51	-1.83	9.49	0.00	0.00
Mild_v_Critical	Cardiovascular	OID00464	CA5A	P35218	1.79	1.25	2.34	3.70	0.00	0.00
Mild_v_Critical	Neurology	OID00372	NTRK3	Q16288	-0.84	-1.10	-0.58	6.95	0.00	0.00
Control_v_Severe	Neurology	OID00318	CLEC1B	Q9P126	-4.41	-5.59	-3.22	11.99	0.00	0.00
Control_v_Critical	Neurology	OID00307	CPA2	P48052	-2.18	-2.87	-1.48	9.65	0.00	0.00
Control_v_Critical	Cardiovascular	OID00391	TNFRSF10A	O00220	1.51	1.03	1.99	3.56	0.00	0.00
Control_v_Mild	Cardiovascular	OID00388	SRC	P12931	-2.01	-2.65	-1.37	6.55	0.00	0.00
Control_v_Critical	Neurology	OID00360	CTSS	P25774	0.82	0.56	1.08	5.89	0.00	0.00
Control_v_Mild	Inflammation	OID00480	LAP TGF-beta-1	P01137	-1.87	-2.47	-1.27	6.84	0.00	0.00
Mild_v_Critical	Immune	OID00993	IL10	P22301	1.32	0.91	1.72	4.89	0.00	0.00
Mild_v_Critical	Immune	OID00953	PRDX3	P30048	0.68	0.47	0.89	-0.06	0.00	0.00
Control_v_Critical	Immune	OID00955	PRDX5	P30044	-2.20	-2.91	-1.49	6.56	0.00	0.00
Control_v_Critical	Immune	OID00946	EDAR	Q9UNE0	-2.51	-3.32	-1.70	3.01	0.00	0.00
Control_v_Critical	Cardiovascular	OID00467	NEMO	Q9Y6K9	-2.79	-3.69	-1.89	6.90	0.00	0.00
Control_v_Critical	Immune	OID00989	PRKCQ	Q04759	-1.08	-1.42	-0.73	1.47	0.00	0.00
Mild_v_Severe	Inflammation	OID00474	MCP-3	P80098	2.48	1.82	3.13	2.42	0.00	0.00
Mild_v_Severe	Neurology	OID00343	GDNFR-alpha-3	O60609	-0.97	-1.23	-0.72	4.73	0.00	0.00
Control_v_Case	Immune	OID00944	CLEC4G	Q6UXB4	0.95	0.62	1.29	3.75	0.00	0.00
Control_v_Mild	Inflammation	OID00478	IL7	P13232	-1.86	-2.47	-1.26	2.89	0.00	0.00
Control_v_Case	Immune	OID00961	GALNT3	Q14435	0.98	0.63	1.32	3.02	0.00	0.00
Control_v_Case	Inflammation	OID00486	CXCL11	O14625	2.12	1.37	2.86	9.37	0.00	0.00
Mild_v_Critical	Cardiovascular	OID00416	SPON2	Q9BUD6	0.38	0.26	0.50	8.53	0.00	0.00
Mild_v_Severe	Inflammation	OID00500	SCF	P21583	-1.28	-1.62	-0.93	8.88	0.00	0.00
Control_v_Mild	Neurology	OID00311	Alpha-2-MRAP	P30533	-1.88	-2.49	-1.27	8.76	0.00	0.00
Control_v_Critical	Neurology	OID00344	PVR	P15151	0.89	0.60	1.18	8.55	0.00	0.00
Mild_v_Critical	Inflammation	OID00518	PD-L1	Q9NZQ7	1.04	0.71	1.37	6.18	0.00	0.00
Control_v_Critical	Inflammation	OID00484	MCP-1	P13500	1.96	1.32	2.61	12.21	0.00	0.00
Mild_v_Severe	Inflammation	OID00522	HGF	P14210	1.35	0.98	1.72	8.44	0.00	0.00
Control_v_Case	Cardiovascular	OID00397	PRSS27	Q9BQR3	-1.06	-1.44	-0.68	8.75	0.00	0.00
Control_v_Critical	Immune	OID00953	PRDX3	P30048	-1.90	-2.53	-1.27	1.36	0.00	0.00

Control_v_Mild	Inflammation	OID00528	IL10	P22301	1.30	0.87	1.72	3.62	0.00	0.00
Mild_v_Critical	Inflammation	OID00488	TRAIL	P50591	-1.13	-1.49	-0.77	7.44	0.00	0.00
Mild_v_Critical	Inflammation	OID00517	IL-18R1	Q13478	0.84	0.57	1.11	9.04	0.00	0.00
Control_v_Case	Neurology	OID00316	CNTN5	O94779	-1.32	-1.80	-0.85	4.40	0.00	0.00
Control_v_Mild	Immune	OID00997	PIK3AP1	Q6ZUJ8	-2.56	-3.40	-1.71	3.87	0.00	0.00
Control_v_Critical	Inflammation	OID00517	IL-18R1	Q13478	1.19	0.79	1.58	9.01	0.00	0.00
Mild_v_Critical	Immune	OID00987	HEXIM1	O94992	1.62	1.10	2.14	4.80	0.00	0.00
Mild_v_Severe	Immune	OID00979	BIRC2	Q13490	0.21	0.15	0.26	0.46	0.00	0.00
Control_v_Case	Inflammation	OID00494	OSM	P13725	2.13	1.36	2.90	4.97	0.00	0.00
Mild_v_Critical	Neurology	OID00364	PLXNB1	O43157	0.88	0.60	1.16	2.20	0.00	0.00
Control_v_Mild	Cardiovascular	OID00439	CCL17	Q92583	-3.33	-4.45	-2.22	9.75	0.00	0.00
Control_v_Mild	Immune	OID00964	TRIM21	P19474	-2.70	-3.61	-1.80	4.32	0.00	0.00
Control_v_Case	Neurology	OID00295	CLM-6	Q08708	0.60	0.38	0.82	6.09	0.00	0.00
Control_v_Critical	Cardiovascular	OID00402	IL-27	Q8NEV9, Q14213	1.63	1.08	2.18	6.29	0.00	0.00
Control_v_Case	Inflammation	OID00490	CXCL9	Q07325	1.71	1.09	2.34	7.90	0.00	0.00
Control_v_Case	Cardiovascular	OID00387	IL-4RA	P24394	0.80	0.51	1.10	2.41	0.00	0.00
Control_v_Severe	Immune	OID00976	EIF4G1	Q04637	-4.63	-5.95	-3.31	6.63	0.00	0.00
Mild_v_Severe	Cardiovascular	OID00408	SCF	P21583	-1.33	-1.70	-0.95	8.96	0.00	0.00
Control_v_Case	Neurology	OID00319	ADAM 23	O75077	-1.04	-1.42	-0.66	3.90	0.00	0.00
Control_v_Mild	Inflammation	OID00562	CSF-1	P09603	0.59	0.39	0.79	10.14	0.00	0.00
Control_v_Mild	Immune	OID00999	STC1	P52823	1.55	1.02	2.08	6.17	0.00	0.00
Control_v_Case	Neurology	OID00336	SCARA5	Q6ZMJ2	-0.64	-0.88	-0.40	8.51	0.00	0.00
Control_v_Critical	Immune	OID00939	ZBTB16	Q05516	-2.59	-3.49	-1.69	3.04	0.00	0.00
Mild_v_Critical	Neurology	OID00360	CTSS	P25774	0.50	0.34	0.67	5.91	0.00	0.00
Control_v_Case	Inflammation	OID01213	DNER	Q8NFT8	-0.68	-0.93	-0.42	8.47	0.00	0.00
Mild_v_Critical	Inflammation	OID00532	CCL3	P10147	1.51	1.00	2.02	6.27	0.00	0.00
Control_v_Critical	Immune	OID00979	BIRC2	Q13490	-1.38	-1.87	-0.90	1.50	0.00	0.00
Control_v_Critical	Inflammation	OID00518	PD-L1	Q9NZQ7	1.25	0.81	1.69	6.17	0.00	0.00
Control_v_Critical	Cardiovascular	OID00464	CA5A	P35218	2.22	1.44	2.99	3.49	0.00	0.00
Control_v_Case	Neurology	OID00331	TMPRSS5	Q9H3S3	-0.72	-0.99	-0.45	2.54	0.00	0.00
Control_v_Case	Immune	OID01010	IFNLR1	Q8IU57	0.72	0.44	0.99	2.73	0.00	0.00
Control_v_Critical	Immune	OID00951	CLEC4A	Q9UMR7	-0.92	-1.25	-0.60	3.59	0.00	0.00
Control_v_Case	Neurology	OID00311	Alpha-2-MRAP	P30533	-1.04	-1.43	-0.64	8.72	0.00	0.00
Control_v_Case	Immune	OID01021	ITGA11	Q9UKX5	-0.98	-1.35	-0.60	2.72	0.00	0.00

Mild_v_Critical	Neurology	OID00324	gal-8	O00214	0.93	0.61	1.25	6.11	0.00	0.00
Mild_v_Critical	Inflammation	OID00528	IL10	P22301	1.17	0.77	1.57	4.67	0.00	0.00
Control_v_Case	Neurology	OID00308	CD38	P28907	0.81	0.50	1.13	5.99	0.00	0.00
Control_v_Case	Cardiovascular	OID00401	PDGF subunit B	P01127	-1.22	-1.70	-0.75	10.25	0.00	0.00
Control_v_Case	Inflammation	OID00500	SCF	P21583	-1.20	-1.67	-0.74	8.80	0.00	0.00
Control_v_Mild	Cardiovascular	OID00449	HB-EGF	Q99075	-2.88	-3.91	-1.84	6.81	0.00	0.00
Control_v_Mild	Cardiovascular	OID00441	MMP7	P09237	1.51	0.96	2.05	8.90	0.00	0.00
Control_v_Case	Inflammation	OID00471	IL8	P10145	1.73	1.05	2.40	5.80	0.00	0.00
Control_v_Case	Inflammation	OID00522	HGF	P14210	1.33	0.81	1.85	9.04	0.00	0.00
Control_v_Mild	Neurology	OID00322	HAGH	Q16775	-2.25	-3.06	-1.44	6.73	0.00	0.00
Mild_v_Critical	Inflammation	OID00535	CXCL10	P02778	1.83	1.19	2.46	11.60	0.00	0.00
Control_v_Case	Inflammation	OID00480	LAP TGF-beta-1	P01137	-1.05	-1.46	-0.64	6.77	0.00	0.00
Mild_v_Severe	Cardiovascular	OID00391	TNFRSF10A	O00220	1.09	0.76	1.42	3.05	0.00	0.00
Control_v_Case	Immune	OID01001	FAM3B	P58499	-0.83	-1.16	-0.51	4.64	0.00	0.00
Control_v_Critical	Cardiovascular	OID00439	CCL17	Q92583	-3.59	-4.90	-2.29	9.69	0.00	0.00
Control_v_Critical	Immune	OID01020	TANK	Q92844	-2.42	-3.30	-1.54	2.69	0.00	0.00
Control_v_Case	Inflammation	OID00554	NT-3	P20783	-0.81	-1.13	-0.49	2.14	0.00	0.00
Mild_v_Critical	Inflammation	OID00542	CD40	P25942	0.81	0.52	1.09	11.36	0.00	0.00
Control_v_Severe	Inflammation	OID00520	CXCL5	P42830	-5.69	-7.45	-3.94	12.55	0.00	0.00
Mild_v_Critical	Inflammation	OID00494	OSM	P13725	1.81	1.18	2.45	5.17	0.00	0.00
Mild_v_Severe	Neurology	OID00354	IL-5R-alpha	Q01344	1.47	1.02	1.92	4.09	0.00	0.00
Control_v_Case	Inflammation	OID00530	CCL23	P55773	1.12	0.68	1.57	10.78	0.00	0.00
Control_v_Case	Inflammation	OID00549	MCP-2	P80075	1.64	0.99	2.30	9.86	0.00	0.00
Control_v_Case	Neurology	OID00318	CLEC1B	Q9P126	-1.50	-2.09	-0.90	11.05	0.00	0.00
Mild_v_Severe	Cardiovascular	OID00396	TRAIL-R2	O14763	1.09	0.75	1.43	6.23	0.00	0.00
Control_v_Case	Neurology	OID00350	GCP5	P78333	-1.14	-1.59	-0.68	4.70	0.00	0.00
Control_v_Case	Neurology	OID00355	PDGF-R-alpha	P16234	0.57	0.34	0.79	5.42	0.00	0.00
Mild_v_Critical	Inflammation	OID00556	CCL20	P78556	2.39	1.53	3.24	8.51	0.00	0.00
Mild_v_Critical	Inflammation	OID00521	TRANCE	O14788	-1.81	-2.46	-1.16	3.79	0.00	0.00
Mild_v_Critical	Neurology	OID00320	MATN3	O15232	1.34	0.86	1.81	9.77	0.00	0.00
Mild_v_Critical	Inflammation	OID05548	TNF	P01375	1.00	0.64	1.36	3.48	0.00	0.00
Mild_v_Critical	Cardiovascular	OID00437	PTX3	P26022	0.99	0.63	1.35	4.79	0.00	0.00
Control_v_Case	Immune	OID00964	TRIM21	P19474	-1.43	-2.00	-0.85	4.19	0.00	0.00
Control_v_Case	Neurology	OID00298	NBL1	P41271	-0.16	-0.22	-0.09	4.94	0.00	0.00

Control_v_Critical	Immune	OID01014	MASP1	P48740	-0.91	-1.26	-0.57	1.88	0.00	0.00
Control_v_Mild	Immune	OID00945	IRF9	Q00978	-1.45	-1.99	-0.91	2.29	0.00	0.00
Control_v_Critical	Inflammation	OID00494	OSM	P13725	2.56	1.59	3.52	5.19	0.00	0.00
Mild_v_Critical	Cardiovascular	OID00469	PARP-1	P09874	1.32	0.84	1.81	5.04	0.00	0.00
Control_v_Critical	Immune	OID01018	DDX58	O95786	2.15	1.33	2.97	4.62	0.00	0.00
Mild_v_Critical	Neurology	OID00335	Beta-NGF	P01138	0.56	0.35	0.77	1.46	0.00	0.00
Mild_v_Severe	Cardiovascular	OID00459	CTSL1	P07711	1.30	0.88	1.73	7.88	0.00	0.00
Mild_v_Critical	Inflammation	OID00472	VEGFA	P15692	1.06	0.67	1.46	11.41	0.00	0.00
Control_v_Critical	Cardiovascular	OID00416	SPON2	Q9BUD6	0.56	0.34	0.77	8.44	0.00	0.00
Mild_v_Critical	Inflammation	OID00530	CCL23	P55773	1.40	0.88	1.92	10.97	0.00	0.00
Mild_v_Severe	Immune	OID01021	ITGA11	Q9UKX5	-0.82	-1.08	-0.55	2.71	0.00	0.00
Mild_v_Critical	Inflammation	OID00562	CSF-1	P09603	0.35	0.22	0.48	10.45	0.00	0.00
Mild_v_Critical	Immune	OID00996	BACH1	O14867	0.90	0.56	1.24	1.49	0.00	0.00
Control_v_Case	Cardiovascular	OID00466	CD4	P01730	0.65	0.38	0.91	4.84	0.00	0.00
Control_v_Case	Cardiovascular	OID00408	SCF	P21583	-1.18	-1.67	-0.69	8.87	0.00	0.00
Control_v_Critical	Inflammation	OID00521	TRANCE	O14788	-1.94	-2.69	-1.19	3.73	0.00	0.00
Control_v_Case	Neurology	OID00322	HAGH	Q16775	-1.51	-2.13	-0.88	6.54	0.00	0.00
Control_v_Critical	Immune	OID00962	FXYD5	Q96DB9	-1.43	-1.99	-0.88	1.56	0.00	0.00
Mild_v_Critical	Neurology	OID00333	GFR-alpha-1	P56159	1.20	0.75	1.65	7.55	0.00	0.00
Mild_v_Critical	Inflammation	OID01213	DNER	Q8NFT8	-0.53	-0.74	-0.33	8.34	0.00	0.00
Mild_v_Critical	Cardiovascular	OID00457	ACE2	Q9BYF1	1.38	0.86	1.90	3.96	0.00	0.00
Control_v_Case	Cardiovascular	OID00438	PSGL-1	Q14242	-0.37	-0.52	-0.21	4.51	0.00	0.00
Control_v_Mild	Immune	OID01015	LAMP3	Q9UQV4	1.77	1.08	2.46	4.45	0.00	0.00
Mild_v_Severe	Inflammation	OID00489	IL-20RA	Q9UHF4	0.35	0.23	0.47	0.93	0.00	0.00
Control_v_Case	Neurology	OID00297	SMOC2	Q9H3U7	-0.81	-1.15	-0.47	7.71	0.00	0.00
Control_v_Case	Neurology	OID00314	RGMB	Q6NW40	-0.71	-1.01	-0.41	5.80	0.00	0.00
Mild_v_Critical	Neurology	OID00287	NMNAT1	Q9HAN9	1.27	0.78	1.75	3.88	0.00	0.00
Mild_v_Critical	Immune	OID01021	ITGA11	Q9UKX5	-1.00	-1.39	-0.62	2.56	0.00	0.00
Control_v_Mild	Neurology	OID00333	GFR-alpha-1	P56159	0.67	0.41	0.93	6.86	0.00	0.00
Control_v_Critical	Inflammation	OID00511	LIF-R	P42702	0.82	0.49	1.14	4.32	0.00	0.00
Control_v_Severe	Inflammation	OID00487	AXIN1	O15169	-5.91	-7.89	-3.93	6.39	0.00	0.00
Control_v_Case	Inflammation	OID00536	4E-BP1	Q13541	-1.41	-2.02	-0.81	9.19	0.00	0.00
Control_v_Case	Cardiovascular	OID00448	AGRP	O00253	0.73	0.42	1.04	5.58	0.00	0.00
Control_v_Critical	Inflammation	OID00552	CX3CL1	P78423	1.06	0.64	1.49	4.47	0.00	0.00

Control_v_Case	Neurology	OID00377	Nr-CAM	Q92823	-0.34	-0.48	-0.19	9.51	0.00	0.00
Control_v_Critical	Immune	OID01004	DFFA	O00273	-1.63	-2.28	-0.97	6.36	0.00	0.00
Control_v_Critical	Neurology	OID00301	NCAN	O14594	0.69	0.41	0.97	8.50	0.00	0.00
Control_v_Critical	Cardiovascular	OID00431	PRELP	P51888	0.44	0.26	0.61	8.35	0.00	0.00
Mild_v_Severe	Inflammation	OID00506	TNFSF14	O43557	1.70	1.11	2.29	4.69	0.00	0.00
Control_v_Mild	Inflammation	OID00536	4E-BP1	Q13541	-2.20	-3.08	-1.32	9.23	0.00	0.00
Control_v_Case	Immune	OID00969	HNMT	P50135	1.03	0.58	1.48	9.41	0.00	0.00
Control_v_Critical	Immune	OID00961	GALNT3	Q14435	1.40	0.83	1.98	3.06	0.00	0.00
Mild_v_Critical	Neurology	OID00318	CLEC1B	Q9P126	1.83	1.11	2.56	10.34	0.00	0.00
Mild_v_Critical	Neurology	OID00363	N2DL-2	Q9BZM5	1.17	0.71	1.63	3.58	0.00	0.00
Control_v_Case	Immune	OID01018	DDX58	O95786	1.33	0.75	1.90	4.04	0.00	0.00
Control_v_Case	Inflammation	OID00532	CCL3	P10147	1.29	0.73	1.85	6.17	0.00	0.00
Mild_v_Severe	Inflammation	OID00494	OSM	P13725	2.04	1.33	2.76	4.63	0.00	0.00
Control_v_Mild	Inflammation	OID00555	TWEAK	O43508	-0.95	-1.33	-0.57	8.94	0.00	0.00
Mild_v_Critical	Inflammation	OID00486	CXCL11	O14625	1.86	1.11	2.60	9.28	0.00	0.00
Control_v_Critical	Cardiovascular	OID00387	IL-4RA	P24394	0.91	0.54	1.29	2.62	0.00	0.00
Control_v_Case	Cardiovascular	OID00427	THBS2	P35442	0.29	0.16	0.42	5.60	0.00	0.00
Control_v_Case	Neurology	OID00349	CLEC10A	Q8IUN9	-0.86	-1.24	-0.47	4.92	0.00	0.00
Mild_v_Critical	Immune	OID00994	SRPK2	P78362	1.09	0.64	1.53	1.05	0.00	0.00
Mild_v_Critical	Cardiovascular	OID00394	TNFRSF11A	Q9Y6Q6	0.86	0.51	1.21	6.01	0.00	0.00
Mild_v_Critical	Inflammation	OID00490	CXCL9	Q07325	1.37	0.81	1.94	8.07	0.00	0.00
Control_v_Severe	Cardiovascular	OID00388	SRC	P12931	-3.65	-4.94	-2.36	7.10	0.00	0.00
Control_v_Critical	Immune	OID00937	GLB1	P16278	-1.65	-2.34	-0.95	2.72	0.00	0.00
Control_v_Case	Neurology	OID00335	Beta-NGF	P01138	0.37	0.20	0.54	1.39	0.00	0.00
Control_v_Mild	Immune	OID00938	PSIP1	O75475	-1.99	-2.82	-1.16	3.58	0.00	0.00
Mild_v_Critical	Inflammation	OID00513	CCL19	Q99731	1.58	0.93	2.23	10.34	0.00	0.00
Mild_v_Critical	Neurology	OID00366	CLM-1	Q8TDQ1	1.16	0.68	1.64	6.91	0.00	0.00
Control_v_Case	Immune	OID00971	MILR1	Q7Z6M3	0.76	0.41	1.11	3.21	0.00	0.00
Control_v_Critical	Cardiovascular	OID00395	PAR-1	P25116	-1.05	-1.50	-0.60	9.29	0.00	0.00
Control_v_Critical	Neurology	OID00317	ADAM 22	Q9P0K1	-0.98	-1.40	-0.56	4.19	0.00	0.00
Control_v_Mild	Immune	OID00951	CLEC4A	Q9UMR7	-0.72	-1.02	-0.41	3.94	0.00	0.00
Control_v_Case	Inflammation	OID00478	IL7	P13232	-1.00	-1.46	-0.54	2.84	0.00	0.00
Mild_v_Critical	Immune	OID00973	NFATC3	Q12968	0.68	0.39	0.97	1.03	0.00	0.00
Control_v_Case	Immune	OID00974	LY75	O60449	-0.63	-0.93	-0.34	2.84	0.00	0.00

Control_v_Severe	Cardiovascular	OID00401	PDGF subunit B	P01127	-2.93	-4.00	-1.85	10.88	0.00	0.00
Control_v_Case	Inflammation	OID00504	MCP-4	Q99616	-1.32	-1.93	-0.70	14.70	0.00	0.00
Mild_v_Critical	Immune	OID00949	CLEC4C	Q8WTT0	-0.96	-1.37	-0.55	3.61	0.00	0.00
Mild_v_Severe	Inflammation	OID00524	IL-24	Q13007	0.69	0.43	0.95	1.08	0.00	0.00
Mild_v_Severe	Immune	OID00985	CKAP4	Q07065	0.79	0.50	1.09	5.02	0.00	0.00
Control_v_Critical	Neurology	OID00359	JAM-B	P57087	-0.92	-1.33	-0.52	7.90	0.00	0.00
Control_v_Critical	Cardiovascular	OID00386	BOC	Q9BWV1	-0.94	-1.35	-0.53	3.34	0.00	0.00
Mild_v_Critical	Neurology	OID00303	ROBO2	Q9HCK4	-0.88	-1.26	-0.50	5.10	0.00	0.00
Mild_v_Critical	Cardiovascular	OID00440	CCL3	P10147	1.35	0.77	1.93	6.83	0.00	0.00
Control_v_Critical	Immune	OID00971	MILR1	Q7Z6M3	1.00	0.56	1.44	3.22	0.00	0.00
Control_v_Critical	Neurology	OID00316	CNTN5	O94779	-0.69	-1.00	-0.39	4.33	0.00	0.00
Control_v_Case	Neurology	OID00367	SPOCK1	Q08629	0.38	0.20	0.55	2.49	0.00	0.00
Control_v_Critical	Neurology	OID00323	LXN	Q9BS40	-0.97	-1.40	-0.54	2.31	0.00	0.00
Mild_v_Critical	Cardiovascular	OID00445	Dkk-1	O94907	1.24	0.70	1.77	8.78	0.00	0.00
Control_v_Severe	Immune	OID00981	NF2	P35240	-4.41	-6.06	-2.76	2.76	0.00	0.00
Mild_v_Critical	Inflammation	OID00501	IL18	Q14116	0.94	0.53	1.34	9.62	0.00	0.00
Mild_v_Critical	Immune	OID01004	DFFA	O00273	0.91	0.52	1.31	4.64	0.00	0.00
Mild_v_Critical	Immune	OID00951	CLEC4A	Q9UMR7	-0.65	-0.93	-0.37	3.37	0.00	0.00
Mild_v_Critical	Neurology	OID00299	EFNA4	P52798	0.54	0.31	0.78	3.16	0.00	0.00
Control_v_Severe	Immune	OID00958	DCTN1	Q14203	-5.27	-7.26	-3.29	6.45	0.00	0.00
Mild_v_Critical	Neurology	OID00345	TNFRSF12A	Q9NP84	1.06	0.60	1.52	5.26	0.00	0.00
Mild_v_Critical	Neurology	OID00343	GDNFR-alpha-3	O60609	-0.68	-0.97	-0.38	4.63	0.00	0.00
Mild_v_Critical	Neurology	OID00321	RSPO1	Q2MKA7	0.95	0.54	1.37	2.96	0.00	0.00
Control_v_Mild	Immune	OID00943	CNTNAP2	Q9UHC6	-0.83	-1.20	-0.47	1.72	0.00	0.00
Mild_v_Severe	Neurology	OID00317	ADAM 22	Q9P0K1	-0.67	-0.93	-0.41	4.11	0.00	0.00
Control_v_Critical	Neurology	OID00342	SCARF2	Q96GP6	-0.86	-1.25	-0.47	6.09	0.00	0.00
Control_v_Mild	Cardiovascular	OID00406	Gal-9	O00182	0.96	0.53	1.38	8.07	0.00	0.00
Control_v_Critical	Cardiovascular	OID00381	ADM	P35318	1.20	0.66	1.73	8.73	0.00	0.00
Control_v_Case	Inflammation	OID00556	CCL20	P78556	1.95	1.01	2.89	8.16	0.00	0.00
Control_v_Critical	Neurology	OID00347	FLRT2	O43155	-0.54	-0.79	-0.30	2.34	0.00	0.00
Control_v_Severe	Immune	OID00994	SRPK2	P78362	-5.96	-8.25	-3.68	4.40	0.00	0.00
Mild_v_Severe	Immune	OID00987	HEXIM1	O94992	1.23	0.75	1.70	4.20	0.00	0.00
Mild_v_Severe	Immune	OID00996	BACH1	O14867	0.65	0.39	0.90	1.10	0.00	0.00
Control_v_Mild	Inflammation	OID00535	CXCL10	P02778	2.42	1.34	3.51	10.08	0.00	0.00

Control_v_Mild	Cardiovascular	OID00464	CA5A	P35218	1.59	0.87	2.30	2.51	0.00	0.00
Control_v_Mild	Neurology	OID00298	NBL1	P41271	-0.25	-0.36	-0.14	4.95	0.00	0.00
Control_v_Mild	Immune	OID01017	CLEC6A	Q6EIG7	1.12	0.61	1.62	2.08	0.00	0.00
Control_v_Case	Inflammation	OID00472	VEGFA	P15692	0.81	0.41	1.20	11.46	0.00	0.00
Control_v_Mild	Inflammation	OID00506	TNFSF14	O43557	-1.77	-2.58	-0.97	5.17	0.00	0.00
Control_v_Case	Cardiovascular	OID00470	HAOX1	Q9UJM8	2.34	1.19	3.50	6.09	0.00	0.00
Control_v_Case	Immune	OID01027	BTN3A2	P78410	0.89	0.45	1.32	3.40	0.00	0.00
Control_v_Critical	Immune	OID00963	TRAF2	Q12933	-1.31	-1.91	-0.71	2.68	0.00	0.00
Mild_v_Critical	Cardiovascular	OID00409	IL18	Q14116	0.94	0.51	1.36	9.43	0.00	0.00
Mild_v_Critical	Immune	OID00991	TREM1	Q9NP99	0.90	0.49	1.30	2.75	0.00	0.00
Mild_v_Critical	Immune	OID00950	IRAK1	P51617	0.71	0.39	1.03	2.10	0.00	0.00
Mild_v_Severe	Immune	OID01009	AREG	P15514	1.54	0.93	2.15	3.68	0.00	0.00
Mild_v_Severe	Cardiovascular	OID00457	ACE2	Q9BYF1	1.14	0.68	1.59	3.62	0.00	0.00
Mild_v_Critical	Neurology	OID00370	EDA2R	Q9HAV5	0.82	0.45	1.19	4.59	0.00	0.00
Control_v_Case	Cardiovascular	OID00409	IL18	Q14116	0.91	0.46	1.35	9.41	0.00	0.00
Control_v_Critical	Cardiovascular	OID00396	TRAIL-R2	O14763	0.93	0.49	1.36	6.96	0.00	0.00
Control_v_Critical	Immune	OID01009	AREG	P15514	1.69	0.90	2.47	4.49	0.00	0.00
Control_v_Case	Neurology	OID00361	N-CDase	Q9NR71	-0.91	-1.36	-0.45	3.50	0.00	0.00
Control_v_Mild	Cardiovascular	OID00404	CXCL1	P09341	-1.85	-2.71	-1.00	10.62	0.00	0.00
Control_v_Critical	Inflammation	OID00549	MCP-2	P80075	2.20	1.17	3.23	10.10	0.00	0.00
Control_v_Mild	Cardiovascular	OID00389	IL-1ra	P18510	1.45	0.78	2.12	5.22	0.00	0.00
Mild_v_Severe	Inflammation	OID00510	MMP-1	P03956	1.91	1.13	2.70	14.28	0.00	0.00
Mild_v_Severe	Immune	OID01013	SIT1	Q9Y3P8	1.42	0.84	2.00	2.49	0.00	0.00
Mild_v_Severe	Inflammation	OID00471	IL8	P10145	1.30	0.76	1.83	4.87	0.00	0.00
Control_v_Mild	Immune	OID00993	IL10	P22301	1.05	0.56	1.54	3.78	0.00	0.00
Mild_v_Severe	Immune	OID00938	PSIP1	O75475	1.54	0.91	2.17	2.85	0.00	0.00
Mild_v_Severe	Neurology	OID00357	CDH6	P55285	-0.78	-1.09	-0.46	4.28	0.00	0.00
Mild_v_Severe	Cardiovascular	OID00455	BNP	P16860	1.84	1.07	2.60	2.08	0.00	0.00
Control_v_Critical	Immune	OID01017	CLEC6A	Q6EIG7	1.28	0.67	1.89	2.54	0.00	0.00
Mild_v_Critical	Neurology	OID00349	CLEC10A	Q8IUN9	-1.00	-1.47	-0.54	4.81	0.00	0.00
Control_v_Critical	Immune	OID00957	TRIM5	Q9C035	-1.32	-1.95	-0.69	3.34	0.00	0.00
Mild_v_Severe	Inflammation	OID00486	CXCL11	O14625	1.64	0.95	2.33	8.71	0.00	0.00
Mild_v_Critical	Inflammation	OID00498	CCL4	P13236	0.83	0.44	1.22	6.07	0.00	0.00
Control_v_Case	Cardiovascular	OID00404	CXCL1	P09341	-0.99	-1.49	-0.48	10.63	0.00	0.00

Mild_v_Critical	Neurology	OID00354	IL-5R-alpha	Q01344	1.29	0.69	1.90	4.41	0.00	0.00
Control_v_Case	Inflammation	OID00501	IL18	Q14116	0.90	0.44	1.37	9.61	0.00	0.00
Control_v_Case	Neurology	OID00325	BCAN	Q96GW7	-0.66	-1.00	-0.33	4.12	0.00	0.00
Control_v_Case	Immune	OID00988	CLEC4D	Q8WXI8	0.93	0.45	1.40	3.81	0.00	0.00
Control_v_Critical	Immune	OID00982	PLXNA4	Q9HCM2	-2.18	-3.22	-1.13	4.73	0.00	0.00
Mild_v_Severe	Immune	OID00994	SRPK2	P78362	0.84	0.49	1.20	0.59	0.00	0.00
Control_v_Case	Inflammation	OID00483	IL-17C	Q9P0M4	1.06	0.52	1.61	2.67	0.00	0.00
Mild_v_Critical	Immune	OID01019	IL12RB1	P42701	0.65	0.34	0.96	2.67	0.00	0.00
Mild_v_Critical	Immune	OID00988	CLEC4D	Q8WXI8	1.07	0.56	1.57	4.03	0.00	0.00
Control_v_Mild	Inflammation	OID05547	IFN-gamma	P01579	2.35	1.23	3.48	7.11	0.00	0.00
Control_v_Case	Cardiovascular	OID00460	hOSCAR	Q8IYS5	0.31	0.15	0.46	10.84	0.00	0.00
Control_v_Case	Inflammation	OID00493	IL-1 alpha	P01583	-0.17	-0.25	-0.08	0.00	0.00	0.00
Control_v_Case	Neurology	OID00347	FLRT2	O43155	-0.35	-0.53	-0.17	2.29	0.00	0.00
Mild_v_Severe	Immune	OID00972	EGLN1	Q9GZT9	0.62	0.35	0.88	1.36	0.00	0.00
Mild_v_Severe	Neurology	OID00305	RGMA	Q96B86	-0.69	-0.99	-0.40	10.74	0.00	0.00
Control_v_Case	Cardiovascular	OID00405	LOX-1	P78380	0.98	0.47	1.49	7.37	0.00	0.00
Control_v_Case	Immune	OID01013	SIT1	Q9Y3P8	0.95	0.46	1.45	2.39	0.00	0.00
Control_v_Case	Immune	OID00945	IRF9	Q00978	-0.74	-1.13	-0.35	2.30	0.00	0.00
Mild_v_Severe	Cardiovascular	OID00401	PDGF subunit B	P01127	1.30	0.73	1.87	9.68	0.00	0.00
Mild_v_Severe	Inflammation	OID00472	VEGFA	P15692	0.94	0.53	1.34	11.05	0.00	0.00
Mild_v_Severe	Immune	OID00980	HSD11B1	P28845	-0.83	-1.19	-0.47	3.11	0.00	0.00
Mild_v_Severe	Cardiovascular	OID00402	IL-27	Q8NEV9, Q14213	0.70	0.40	1.01	6.29	0.00	0.00
Mild_v_Severe	Immune	OID01014	MASP1	P48740	-0.62	-0.90	-0.35	1.78	0.00	0.00
Control_v_Critical	Cardiovascular	OID00449	HB-EGF	Q99075	-1.48	-2.20	-0.75	7.00	0.00	0.00
Mild_v_Severe	Inflammation	OID00530	CCL23	P55773	1.02	0.57	1.47	10.59	0.00	0.00
Control_v_Critical	Neurology	OID00355	PDGF-R-alpha	P16234	0.68	0.35	1.02	5.49	0.00	0.00
Mild_v_Severe	Neurology	OID00303	ROBO2	Q9HCK4	-0.98	-1.41	-0.55	5.29	0.00	0.00
Mild_v_Critical	Immune	OID00972	EGLN1	Q9GZT9	0.57	0.29	0.85	1.62	0.00	0.00
Mild_v_Critical	Neurology	OID00338	NTRK2	Q16620	-0.71	-1.05	-0.36	6.00	0.00	0.00
Mild_v_Critical	Inflammation	OID00478	IL7	P13232	0.88	0.45	1.31	2.37	0.00	0.00
Mild_v_Critical	Cardiovascular	OID00381	ADM	P35318	0.75	0.38	1.12	8.84	0.00	0.00
Mild_v_Critical	Neurology	OID00351	BMP-4	P12644	-0.93	-1.38	-0.47	4.60	0.00	0.00
Control_v_Critical	Neurology	OID00336	SCARA5	Q6ZMJ2	-0.63	-0.95	-0.31	8.55	0.00	0.00
Control_v_Case	Inflammation	OID00491	CST5	P28325	-0.57	-0.87	-0.26	6.54	0.00	0.00

Control_v_Case	Cardiovascular	OID00461	TNFRSF13B	O14836	0.57	0.26	0.87	9.90	0.00	0.00
Control_v_Severe	Immune	OID00954	FGF2	P09038	-5.15	-7.33	-2.98	3.01	0.00	0.00
Control_v_Mild	Cardiovascular	OID00437	PTX3	P26022	1.09	0.55	1.63	3.99	0.00	0.00
Control_v_Case	Cardiovascular	OID00403	IL-17D	Q8TAD2	-0.38	-0.58	-0.17	2.39	0.00	0.00
Mild_v_Critical	Cardiovascular	OID00436	CEACAM8	P31997	0.67	0.33	1.00	4.52	0.00	0.00
Control_v_Critical	Inflammation	OID00536	4E-BP1	Q13541	-1.85	-2.79	-0.90	9.81	0.00	0.00
Control_v_Case	Neurology	OID00354	IL-5R-alpha	Q01344	1.16	0.53	1.78	4.36	0.00	0.00
Mild_v_Critical	Immune	OID01014	MASP1	P48740	-0.47	-0.71	-0.24	1.70	0.00	0.00
Control_v_Case	Neurology	OID00363	N2DL-2	Q9BZM5	0.61	0.28	0.94	3.40	0.00	0.00
Control_v_Critical	Immune	OID00969	HNMT	P50135	1.31	0.64	1.99	9.61	0.00	0.00
Control_v_Case	Cardiovascular	OID00380	ANGPT1	Q15389	-1.27	-1.97	-0.57	8.66	0.00	0.00
Control_v_Critical	Cardiovascular	OID00405	LOX-1	P78380	1.48	0.71	2.24	7.55	0.00	0.00
Control_v_Mild	Cardiovascular	OID00380	ANGPT1	Q15389	-1.92	-2.90	-0.95	8.81	0.00	0.00
Mild_v_Severe	Inflammation	OID00528	IL10	P22301	0.81	0.44	1.19	4.11	0.00	0.00
Mild_v_Severe	Neurology	OID00344	PVR	P15151	0.58	0.31	0.85	8.30	0.00	0.00
Control_v_Case	Cardiovascular	OID00444	DCN	P07585	0.42	0.19	0.65	4.52	0.00	0.00
Control_v_Case	Cardiovascular	OID00421	PAPPA	Q13219	-0.76	-1.18	-0.34	2.97	0.00	0.00
Mild_v_Critical	Cardiovascular	OID00427	THBS2	P35442	0.20	0.10	0.30	5.64	0.00	0.00
Mild_v_Critical	Inflammation	OID00476	CDCP1	Q9H5V8	1.27	0.62	1.92	3.47	0.00	0.00
Mild_v_Critical	Neurology	OID00378	KYNU	Q16719	0.83	0.41	1.26	8.98	0.00	0.00
Control_v_Severe	Cardiovascular	OID00467	NEMO	Q9Y6K9	-4.94	-7.12	-2.76	7.39	0.00	0.00
Control_v_Severe	Immune	OID00987	HEXIM1	O94992	-4.36	-6.28	-2.44	7.63	0.00	0.00
Control_v_Severe	Neurology	OID00342	SCARF2	Q96GP6	-2.42	-3.48	-1.35	6.21	0.00	0.00
Control_v_Critical	Immune	OID00952	PRDX1	Q06830	-1.65	-2.51	-0.78	4.31	0.00	0.00
Control_v_Critical	Inflammation	OID00488	TRAIL	P50591	-0.60	-0.91	-0.28	7.29	0.00	0.00
Control_v_Case	Neurology	OID00366	CLM-1	Q8TDQ1	0.94	0.41	1.46	6.67	0.00	0.00
Mild_v_Critical	Immune	OID00976	EIF4G1	Q04637	1.26	0.61	1.91	3.55	0.00	0.00
Control_v_Critical	Neurology	OID00333	GFR-alpha-1	P56159	0.99	0.47	1.51	7.43	0.00	0.00
Mild_v_Critical	Cardiovascular	OID00387	IL-4RA	P24394	0.79	0.38	1.21	2.59	0.00	0.00
Mild_v_Critical	Immune	OID00937	GLB1	P16278	0.61	0.29	0.92	1.17	0.00	0.00
Mild_v_Critical	Cardiovascular	OID00433	XCL1	P47992	0.80	0.38	1.21	5.34	0.00	0.00
Mild_v_Critical	Inflammation	OID00484	MCP-1	P13500	1.43	0.69	2.17	12.28	0.00	0.00
Mild_v_Critical	Cardiovascular	OID00383	SLAMF7	Q9NQ25	1.01	0.49	1.54	3.95	0.00	0.00
Control_v_Case	Cardiovascular	OID00426	KIM1	Q96D42	1.01	0.44	1.59	8.62	0.00	0.00

Mild_v_Severe	Immune	OID00950	IRAK1	P51617	0.53	0.28	0.78	1.81	0.00	0.00
Mild_v_Severe	Immune	OID00939	ZBTB16	Q05516	0.76	0.40	1.12	0.79	0.00	0.00
Longitudinal_in_Mild	Cardiovascular	OID00386	BOC	Q9BWV1	1.57	1.25	1.90	3.64	0.00	0.00
Control_v_Critical	Immune	OID00948	DGKZ	Q13574	-0.69	-1.06	-0.32	1.10	0.00	0.00
Control_v_Critical	Inflammation	OID00491	CST5	P28325	-0.89	-1.36	-0.41	6.61	0.00	0.00
Mild_v_Critical	Immune	OID00999	STC1	P52823	0.44	0.21	0.66	7.16	0.00	0.00
Control_v_Case	Immune	OID00992	CXADR	P78310	0.51	0.22	0.79	2.44	0.00	0.00
Mild_v_Critical	Cardiovascular	OID00412	RAGE	Q15109	0.76	0.36	1.16	13.84	0.00	0.00
Control_v_Case	Neurology	OID00310	MSR1	P21757	0.59	0.25	0.93	6.70	0.00	0.00
Mild_v_Severe	Cardiovascular	OID00450	GDF-2	Q9UK05	-1.09	-1.62	-0.57	8.48	0.00	0.00
Control_v_Mild	Immune	OID01012	PADI2	Q9Y2J8	-0.85	-1.30	-0.41	1.22	0.00	0.00
Mild_v_Severe	Neurology	OID00337	CD200	P41217	-0.75	-1.11	-0.39	6.20	0.00	0.00
Mild_v_Critical	Neurology	OID00311	Alpha-2-MRAP	P30533	0.86	0.40	1.32	8.29	0.00	0.00
Severe_v_Critical	Inflammation	OID00517	IL-18R1	Q13478	0.58	0.36	0.80	9.62	0.00	0.00
Severe_v_Critical	Neurology	OID00358	DDR1	Q08345	0.42	0.26	0.58	7.18	0.00	0.00
Control_v_Case	Neurology	OID00356	CTSC	P53634	-0.60	-0.95	-0.26	4.27	0.00	0.00
Mild_v_Severe	Immune	OID00976	EIF4G1	Q04637	1.24	0.64	1.84	3.13	0.00	0.00
Control_v_Mild	Inflammation	OID00488	TRAIL	P50591	0.79	0.37	1.21	7.71	0.00	0.00
Control_v_Critical	Inflammation	OID00555	TWEAK	O43508	-0.95	-1.47	-0.44	8.63	0.00	0.00
Control_v_Case	Neurology	OID00358	DDR1	Q08345	-0.30	-0.48	-0.13	7.25	0.00	0.00
Mild_v_Severe	Inflammation	OID00480	LAP TGF-beta-1	P01137	0.88	0.45	1.31	6.20	0.00	0.00
Control_v_Critical	Neurology	OID00303	ROBO2	Q9HCK4	-0.71	-1.09	-0.32	5.21	0.00	0.00
Mild_v_Critical	Neurology	OID00325	BCAN	Q96GW7	-0.67	-1.02	-0.31	3.97	0.00	0.00
Control_v_Mild	Inflammation	OID00496	CXCL1	P09341	-1.61	-2.47	-0.75	9.85	0.00	0.00
Mild_v_Severe	Immune	OID00991	TREM1	Q9NP99	0.80	0.41	1.20	2.53	0.00	0.00
Control_v_Mild	Inflammation	OID00504	MCP-4	Q99616	-1.78	-2.73	-0.83	14.86	0.00	0.00
Control_v_Mild	Inflammation	OID00542	CD40	P25942	-0.86	-1.31	-0.40	11.35	0.00	0.00
Mild_v_Severe	Immune	OID00965	LILRB4	Q8NHJ6	1.03	0.52	1.53	3.90	0.00	0.00
Mild_v_Critical	Immune	OID00944	CLEC4G	Q6UXB4	0.63	0.29	0.97	3.94	0.00	0.00
Control_v_Case	Inflammation	OID00496	CXCL1	P09341	-0.86	-1.37	-0.36	9.85	0.00	0.00
Control_v_Critical	Cardiovascular	OID00427	THBS2	P35442	0.43	0.19	0.66	5.61	0.00	0.00
Control_v_Case	Neurology	OID00352	FcRL2	Q96LA5	0.59	0.24	0.93	5.11	0.00	0.00
Mild_v_Critical	Cardiovascular	OID00450	GDF-2	Q9UK05	-1.00	-1.54	-0.46	8.23	0.00	0.00
Control_v_Mild	Neurology	OID00369	Dkk-4	Q9UBT3	-0.81	-1.25	-0.37	3.48	0.00	0.00

Mild_v_Severe	Inflammation	OID00521	TRANCE	O14788	-1.23	-1.85	-0.62	4.37	0.00	0.00
Control_v_Critical	Inflammation	OID00486	CXCL11	O14625	1.77	0.79	2.75	9.81	0.00	0.00
Control_v_Critical	Neurology	OID00327	NEP	P08473	-1.27	-1.98	-0.57	2.62	0.00	0.00
Control_v_Mild	Neurology	OID00327	NEP	P08473	1.05	0.48	1.62	2.42	0.00	0.00
Mild_v_Critical	Neurology	OID00305	RGMA	Q96B86	-0.77	-1.20	-0.35	10.54	0.00	0.00
Control_v_Severe	Cardiovascular	OID00428	TM	P07204	-1.80	-2.64	-0.97	9.54	0.00	0.00
Mild_v_Severe	Neurology	OID00372	NTRK3	Q16288	-0.61	-0.92	-0.31	7.15	0.00	0.00
Control_v_Critical	Immune	OID00988	CLEC4D	Q8WXI8	1.56	0.69	2.42	3.95	0.00	0.00
Control_v_Critical	Immune	OID01010	IFNLR1	Q8IU57	0.87	0.39	1.35	2.75	0.00	0.00
Control_v_Mild	Cardiovascular	OID00419	GLO1	Q04760	-1.21	-1.87	-0.55	5.96	0.00	0.00
Control_v_Case	Neurology	OID00378	KYNU	Q16719	0.69	0.28	1.10	9.04	0.00	0.00
Control_v_Severe	Immune	OID00942	HCLS1	P14317	-4.39	-6.45	-2.33	7.44	0.00	0.00
Control_v_Critical	Cardiovascular	OID00382	CD40-L	P29965	-2.32	-3.62	-1.02	5.77	0.00	0.00
Mild_v_Severe	Cardiovascular	OID00397	PRSS27	Q9BQR3	-0.73	-1.11	-0.36	8.76	0.00	0.00
Mild_v_Severe	Neurology	OID00348	CPM	P14384	-0.54	-0.81	-0.27	6.53	0.00	0.00
Control_v_Case	Inflammation	OID00514	IL-15RA	Q13261	0.40	0.16	0.64	1.46	0.00	0.00
Severe_v_Critical	Cardiovascular	OID00389	IL-1ra	P18510	1.05	0.63	1.47	7.32	0.00	0.00
Control_v_Mild	Cardiovascular	OID00438	PSGL-1	Q14242	-0.38	-0.59	-0.17	4.61	0.00	0.00
Control_v_Severe	Immune	OID00939	ZBTB16	Q05516	-5.14	-7.58	-2.70	3.51	0.00	0.00
Mild_v_Critical	Immune	OID00957	TRIM5	Q9C035	0.87	0.38	1.35	1.96	0.00	0.00
Mild_v_Critical	Immune	OID00939	ZBTB16	Q05516	0.85	0.38	1.33	1.12	0.00	0.00
Mild_v_Severe	Cardiovascular	OID00406	Gal-9	O00182	0.59	0.29	0.89	8.38	0.00	0.00
Mild_v_Severe	Immune	OID01003	ICA1	Q05084	0.46	0.23	0.70	0.89	0.00	0.00
Mild_v_Critical	Cardiovascular	OID00417	GH	P01241	2.51	1.10	3.91	7.58	0.00	0.00
Mild_v_Critical	Cardiovascular	OID00460	hOSCAR	Q8IYS5	0.28	0.12	0.44	10.85	0.00	0.00
Mild_v_Critical	Neurology	OID00371	LAT	O43561	1.68	0.74	2.62	5.64	0.00	0.00
Mild_v_Critical	Cardiovascular	OID00384	PGF	P49763	0.41	0.18	0.63	7.63	0.00	0.00
Control_v_Severe	Cardiovascular	OID00420	CD84	Q9UIB8	-2.52	-3.73	-1.32	5.53	0.00	0.00
Control_v_Mild	Inflammation	OID00484	MCP-1	P13500	1.00	0.45	1.56	11.31	0.00	0.00
Control_v_Mild	Neurology	OID00321	RSPO1	Q2MKA7	-0.57	-0.89	-0.25	2.70	0.00	0.00
Control_v_Mild	Immune	OID01014	MASP1	P48740	-0.54	-0.85	-0.24	2.12	0.00	0.00
Control_v_Case	Cardiovascular	OID00457	ACE2	Q9BYF1	0.80	0.31	1.28	3.80	0.00	0.00
Control_v_Case	Cardiovascular	OID00384	PGF	P49763	0.35	0.14	0.56	7.57	0.00	0.00
Control_v_Critical	Neurology	OID00361	N-CDase	Q9NR71	-1.37	-2.15	-0.58	3.53	0.00	0.00

Mild_v_Critical	Immune	OID00952	PRDX1	Q06830	1.26	0.55	1.97	2.32	0.00	0.00
Mild_v_Severe	Immune	OID00993	IL10	P22301	0.89	0.43	1.35	4.27	0.00	0.00
Mild_v_Severe	Inflammation	OID00498	CCL4	P13236	0.85	0.41	1.29	5.89	0.00	0.00
Mild_v_Severe	Neurology	OID00301	NCAN	O14594	-0.54	-0.82	-0.26	8.52	0.00	0.00
Control_v_Mild	Neurology	OID00309	SMPD1	P17405	0.72	0.32	1.13	4.48	0.00	0.00
Mild_v_Critical	Cardiovascular	OID00405	LOX-1	P78380	1.21	0.52	1.91	7.46	0.00	0.00
Mild_v_Critical	Cardiovascular	OID00397	PRSS27	Q9BQR3	-0.77	-1.20	-0.33	8.59	0.00	0.00
Mild_v_Critical	Inflammation	OID00480	LAP TGF-beta-1	P01137	0.69	0.30	1.08	6.33	0.00	0.00
Control_v_Case	Immune	OID00996	BACH1	O14867	-0.77	-1.25	-0.30	1.99	0.00	0.00
Mild_v_Critical	Cardiovascular	OID00414	CTRC	Q99895	-1.09	-1.71	-0.47	9.93	0.00	0.00
Control_v_Critical	Inflammation	OID00524	IL-24	Q13007	0.68	0.29	1.07	1.35	0.00	0.00
Mild_v_Severe	Neurology	OID00302	PRTG	Q2VWP7	-0.33	-0.50	-0.15	6.46	0.00	0.00
Mild_v_Severe	Inflammation	OID00490	CXCL9	Q07325	0.88	0.42	1.35	7.49	0.00	0.00
Control_v_Case	Immune	OID00960	CDSN	Q15517	-0.73	-1.19	-0.28	2.82	0.00	0.00
Mild_v_Severe	Cardiovascular	OID00447	PRSS8	Q16651	0.37	0.18	0.57	8.85	0.00	0.00
Mild_v_Severe	Cardiovascular	OID00419	GLO1	Q04760	1.26	0.59	1.93	5.63	0.00	0.00
Mild_v_Critical	Immune	OID01018	DDX58	O95786	1.25	0.53	1.97	3.85	0.00	0.00
Control_v_Severe	Immune	OID01023	LAG3	P18627	2.43	1.24	3.62	2.65	0.00	0.00
Control_v_Severe	Neurology	OID00324	gal-8	O00214	-3.43	-5.12	-1.75	8.03	0.00	0.00
Control_v_Critical	Neurology	OID00297	SMOC2	Q9H3U7	-0.89	-1.41	-0.37	7.78	0.00	0.00
Longitudinal_in_Mild	Neurology	OID00378	KYNU	Q16719	-1.48	-1.83	-1.14	8.55	0.00	0.00
Control_v_Mild	Immune	OID01019	IL12RB1	P42701	0.58	0.25	0.90	2.23	0.00	0.00
Control_v_Mild	Inflammation	OID00511	LIF-R	P42702	0.44	0.19	0.70	4.12	0.00	0.00
Control_v_Critical	Neurology	OID00352	FcRL2	Q96LA5	0.88	0.36	1.41	5.15	0.00	0.00
Mild_v_Severe	Neurology	OID00328	GDF-8	O14793	-0.97	-1.49	-0.45	3.32	0.00	0.00
Mild_v_Severe	Cardiovascular	OID00386	BOC	Q9BWV1	-0.64	-0.99	-0.30	3.49	0.00	0.00
Control_v_Case	Neurology	OID00287	NMNAT1	Q9HAN9	1.07	0.40	1.74	3.75	0.00	0.00
Control_v_Mild	Neurology	OID00345	TNFRSF12A	Q9NP84	-0.70	-1.10	-0.30	4.91	0.00	0.00
Control_v_Mild	Cardiovascular	OID00435	SORT1	Q99523	-0.67	-1.06	-0.28	8.67	0.00	0.00
Control_v_Mild	Inflammation	OID00552	CX3CL1	P78423	0.62	0.26	0.99	4.10	0.00	0.00
Mild_v_Severe	Neurology	OID00331	TMPRSS5	Q9H3S3	-0.46	-0.71	-0.21	2.55	0.00	0.00
Mild_v_Severe	Immune	OID00951	CLEC4A	Q9UMR7	-0.58	-0.89	-0.27	3.54	0.00	0.00
Severe_v_Critical	Immune	OID00937	GLB1	P16278	0.76	0.44	1.09	1.38	0.00	0.00
Control_v_Severe	Immune	OID00998	SPRY2	O43597	-6.01	-9.02	-3.01	5.80	0.00	0.00

Mild_v_Critical	Immune	OID01008	CXCL12	P48061	0.36	0.15	0.57	1.53	0.00	0.00
Control_v_Critical	Immune	OID00997	PIK3AP1	Q6ZUJ8	-1.28	-2.05	-0.52	4.28	0.00	0.00
Control_v_Severe	Immune	OID00940	IRAK4	Q9NWZ3	-4.71	-7.09	-2.34	5.62	0.00	0.00
Control_v_Severe	Neurology	OID00350	GCP5	P78333	-4.14	-6.23	-2.05	4.98	0.00	0.00
Control_v_Critical	Inflammation	OID00493	IL-1 alpha	P01583	-0.26	-0.41	-0.10	0.02	0.00	0.00
Control_v_Case	Neurology	OID00299	EFNA4	P52798	0.40	0.14	0.65	3.08	0.00	0.00
Control_v_Case	Inflammation	OID00488	TRAIL	P50591	-0.47	-0.77	-0.17	7.48	0.00	0.00
Mild_v_Severe	Cardiovascular	OID00445	Dkk-1	O94907	1.11	0.50	1.72	8.51	0.00	0.00
Mild_v_Severe	Neurology	OID00325	BCAN	Q96GW7	-0.64	-0.99	-0.29	4.08	0.00	0.00
Control_v_Case	Neurology	OID00362	NAAA	Q02083	-0.53	-0.88	-0.19	3.34	0.00	0.00
Control_v_Critical	Neurology	OID00311	Alpha-2-MRAP	P30533	-1.02	-1.64	-0.41	9.26	0.00	0.00
Control_v_Mild	Neurology	OID00342	SCARF2	Q96GP6	-0.48	-0.76	-0.20	6.23	0.00	0.00
Mild_v_Severe	Immune	OID01010	IFNLR1	Q8IU57	0.48	0.22	0.75	2.70	0.00	0.00
Mild_v_Critical	Neurology	OID00309	SMPD1	P17405	0.49	0.20	0.78	4.92	0.00	0.00
Control_v_Case	Cardiovascular	OID00431	PRELP	P51888	0.20	0.07	0.33	8.35	0.00	0.00
Control_v_Mild	Cardiovascular	OID00421	PAPPA	Q13219	-1.08	-1.73	-0.44	2.99	0.00	0.00
Mild_v_Critical	Immune	OID00955	PRDX5	P30044	1.15	0.45	1.84	4.46	0.00	0.00
Mild_v_Critical	Inflammation	OID00552	CX3CL1	P78423	0.50	0.20	0.80	4.49	0.00	0.00
Mild_v_Critical	Inflammation	OID00534	CXCL6	P80162	0.99	0.39	1.58	7.96	0.00	0.00
Control_v_Mild	Neurology	OID00300	SCARB2	Q14108	0.59	0.24	0.95	4.59	0.00	0.00
Control_v_Case	Cardiovascular	OID00455	BNP	P16860	1.01	0.35	1.67	1.82	0.00	0.00
Control_v_Mild	Inflammation	OID00517	IL-18R1	Q13478	0.76	0.30	1.22	8.39	0.00	0.00
Mild_v_Severe	Inflammation	OID00532	CCL3	P10147	0.98	0.43	1.53	5.72	0.00	0.00
Control_v_Severe	Cardiovascular	OID00443	ITGB1BP2	Q9UKP3	-7.76	-11.80	-3.71	6.78	0.00	0.00
Control_v_Severe	Immune	OID00967	KRT19	P08727	3.42	1.64	5.21	2.83	0.00	0.00
Control_v_Severe	Neurology	OID00357	CDH6	P55285	-1.96	-2.98	-0.94	4.52	0.00	0.00
Control_v_Severe	Cardiovascular	OID00455	BNP	P16860	-3.41	-5.20	-1.63	1.80	0.00	0.01
Control_v_Case	Cardiovascular	OID00445	Dkk-1	O94907	-0.66	-1.10	-0.23	9.09	0.00	0.01
Mild_v_Critical	Inflammation	OID00538	SIRT2	Q8IXJ6	0.99	0.39	1.60	3.90	0.00	0.01
Control_v_Case	Neurology	OID00313	EPHB6	O15197	-0.33	-0.54	-0.11	3.88	0.00	0.01
Control_v_Mild	Immune	OID00985	CKAP4	Q07065	0.52	0.21	0.84	4.65	0.00	0.01
Mild_v_Severe	Immune	OID00954	FGF2	P09038	0.60	0.26	0.94	0.49	0.00	0.01
Control_v_Case	Immune	OID01026	ITGB6	P18564	-0.37	-0.61	-0.13	3.03	0.00	0.01
Control_v_Critical	Cardiovascular	OID00399	TF	P13726	0.73	0.28	1.18	6.17	0.00	0.01

Mild_v_Severe	Neurology	OID00342	SCARF2	Q96GP6	-0.52	-0.81	-0.22	5.93	0.00	0.01
Control_v_Severe	Immune	OID00983	SH2B3	Q9UQQ2	-5.07	-7.75	-2.39	5.84	0.00	0.01
Control_v_Case	Inflammation	OID00481	uPA	P00749	0.39	0.13	0.64	9.83	0.00	0.01
Control_v_Severe	Inflammation	OID00557	ST1A1	P50225	-5.03	-7.70	-2.37	5.31	0.00	0.01
Control_v_Critical	Cardiovascular	OID00441	MMP7	P09237	1.14	0.43	1.85	9.12	0.00	0.01
Mild_v_Severe	Cardiovascular	OID00405	LOX-1	P78380	0.89	0.38	1.40	7.10	0.00	0.01
Control_v_Case	Inflammation	OID00499	CD6	P30203	-0.57	-0.95	-0.19	6.02	0.00	0.01
Control_v_Critical	Cardiovascular	OID00444	DCN	P07585	0.68	0.26	1.10	4.58	0.00	0.01
Mild_v_Critical	Cardiovascular	OID00423	REN	P00797	0.98	0.37	1.59	6.76	0.00	0.01
Control_v_Case	Cardiovascular	OID00433	XCL1	P47992	0.51	0.17	0.84	5.13	0.00	0.01
Mild_v_Severe	Inflammation	OID00501	IL18	Q14116	0.93	0.39	1.47	9.18	0.00	0.01
Mild_v_Severe	Neurology	OID00296	EZR	P15311	0.49	0.21	0.77	3.94	0.00	0.01
Mild_v_Critical	Inflammation	OID00510	MMP-1	P03956	1.23	0.46	1.99	14.34	0.00	0.01
Severe_v_Critical	Cardiovascular	OID00441	MMP7	P09237	0.72	0.40	1.04	9.86	0.00	0.01
Mild_v_Critical	Immune	OID01015	LAMP3	Q9UQV4	0.86	0.32	1.39	5.36	0.00	0.01
Control_v_Critical	Inflammation	OID00513	CCL19	Q99731	1.77	0.65	2.88	10.30	0.00	0.01
Control_v_Case	Cardiovascular	OID00446	LPL	P06858	-0.44	-0.73	-0.14	9.66	0.00	0.01
Control_v_Mild	Inflammation	OID00482	IL6	P05231	1.08	0.41	1.75	2.37	0.00	0.01
Control_v_Mild	Immune	OID00965	LILRB4	Q8NHJ6	0.88	0.34	1.43	3.30	0.00	0.01
Control_v_Severe	Immune	OID00968	ITM2A	O43736	2.99	1.38	4.60	2.23	0.00	0.01
Control_v_Case	Neurology	OID00290	CADM3	Q8N126	-0.55	-0.92	-0.18	3.28	0.00	0.01
Mild_v_Severe	Inflammation	OID00562	CSF-1	P09603	0.26	0.11	0.42	10.28	0.00	0.01
Mild_v_Severe	Cardiovascular	OID00416	SPON2	Q9BUD6	0.27	0.11	0.42	8.40	0.00	0.01
Control_v_Case	Inflammation	OID05124	CD8A	P01732	0.82	0.26	1.39	9.73	0.00	0.01
Control_v_Severe	Neurology	OID00365	TNFRSF21	O75509	-1.60	-2.47	-0.73	8.22	0.00	0.01
Control_v_Case	Immune	OID00949	CLEC4C	Q8WTT0	-0.61	-1.03	-0.19	3.68	0.00	0.01
Severe_v_Critical	Neurology	OID00356	CTSC	P53634	0.70	0.37	1.02	4.15	0.00	0.01
Severe_v_Critical	Inflammation	OID00518	PD-L1	Q9NZQ7	0.49	0.26	0.72	6.82	0.00	0.01
Mild_v_Severe	Inflammation	OID00518	PD-L1	Q9NZQ7	0.66	0.27	1.05	5.74	0.00	0.01
Control_v_Critical	Inflammation	OID00479	OPG	O00300	0.85	0.31	1.40	10.58	0.00	0.01
Control_v_Mild	Cardiovascular	OID00390	IL6	P05231	1.18	0.44	1.92	3.15	0.00	0.01
Control_v_Case	Cardiovascular	OID00414	CTRC	Q99895	-0.90	-1.52	-0.28	9.99	0.00	0.01
Longitudinal_in_Mild	Immune	OID00998	SPRY2	O43597	1.34	0.96	1.72	1.94	0.00	0.01

Longitudinal_in_Mild	Cardiovascular	OID00426	KIM1	Q96D42	1.06	0.73	1.40	8.18	0.00	0.01
Mild_v_Severe	Cardiovascular	OID00409	IL18	Q14116	0.89	0.36	1.43	9.00	0.00	0.01
Mild_v_Severe	Cardiovascular	OID00461	TNFRSF13B	O14836	0.58	0.23	0.92	9.85	0.00	0.01
Control_v_Mild	Inflammation	OID00510	MMP-1	P03956	-1.53	-2.49	-0.56	14.52	0.00	0.01
Mild_v_Severe	Neurology	OID00371	LAT	O43561	1.94	0.78	3.10	5.27	0.00	0.01
Mild_v_Severe	Neurology	OID00353	MDGA1	Q8NFP4	-1.22	-1.95	-0.49	4.01	0.00	0.01
Mild_v_Severe	Neurology	OID00318	CLEC1B	Q9P126	1.62	0.64	2.59	9.89	0.00	0.01
Mild_v_Severe	Immune	OID00978	PTH1R	Q03431	-0.36	-0.57	-0.14	4.01	0.00	0.01
Mild_v_Severe	Neurology	OID00316	CNTN5	O94779	-1.01	-1.62	-0.40	4.50	0.00	0.01
Mild_v_Severe	Cardiovascular	OID00440	CCL3	P10147	0.89	0.35	1.43	6.38	0.00	0.01
Mild_v_Severe	Inflammation	OID00503	TGF-alpha	P01135	0.62	0.25	1.00	3.06	0.00	0.01
Mild_v_Severe	Neurology	OID00333	GFR-alpha-1	P56159	0.70	0.28	1.13	7.17	0.00	0.01
Mild_v_Severe	Immune	OID01008	CXCL12	P48061	0.28	0.11	0.45	1.43	0.00	0.01
Control_v_Mild	Cardiovascular	OID00397	PRSS27	Q9BQR3	-0.63	-1.03	-0.23	9.09	0.00	0.01
Mild_v_Severe	Inflammation	OID00545	FGF-19	O95750	-1.53	-2.47	-0.59	8.35	0.00	0.01
Mild_v_Severe	Neurology	OID00361	N-CDase	Q9NR71	-0.61	-0.98	-0.24	3.45	0.00	0.01
Mild_v_Critical	Inflammation	OID00483	IL-17C	Q9P0M4	0.95	0.34	1.57	2.84	0.00	0.01
Mild_v_Severe	Inflammation	OID00488	TRAIL	P50591	-0.69	-1.11	-0.26	7.76	0.00	0.01
Mild_v_Critical	Inflammation	OID00502	SLAMF1	Q13291	0.59	0.21	0.97	2.69	0.00	0.01
Control_v_Severe	Neurology	OID00327	NEP	P08473	-2.86	-4.46	-1.26	2.52	0.00	0.01
Control_v_Critical	Cardiovascular	OID00409	IL18	Q14116	0.76	0.26	1.25	9.68	0.00	0.01
Mild_v_Severe	Cardiovascular	OID00460	hOSCAR	Q8IYS5	0.28	0.11	0.45	10.77	0.00	0.01
Mild_v_Severe	Cardiovascular	OID00407	GIF	P27352	-1.11	-1.79	-0.43	6.75	0.00	0.01
Mild_v_Severe	Cardiovascular	OID00417	GH	P01241	2.45	0.94	3.96	7.54	0.00	0.01
Control_v_Critical	Immune	OID01019	IL12RB1	P42701	0.56	0.19	0.93	2.60	0.00	0.01
Mild_v_Critical	Neurology	OID00308	CD38	P28907	0.63	0.22	1.04	6.20	0.00	0.01
Mild_v_Severe	Inflammation	OID00542	CD40	P25942	0.50	0.19	0.82	11.02	0.00	0.01
Control_v_Critical	Neurology	OID00350	GCP5	P78333	-1.07	-1.77	-0.36	4.78	0.00	0.01
Control_v_Mild	Cardiovascular	OID00452	THPO	P40225	-0.67	-1.10	-0.24	4.09	0.00	0.01
Control_v_Case	Neurology	OID00294	Siglec-9	Q9Y336	0.31	0.09	0.54	5.00	0.01	0.01
Longitudinal_in_Mild	Inflammation	OID00500	SCF	P21583	1.66	1.10	2.22	9.30	0.00	0.01
Control_v_Critical	Inflammation	OID00533	Flt3L	P49771	-0.90	-1.51	-0.30	9.11	0.00	0.01
Mild_v_Critical	Cardiovascular	OID00424	DECR1	Q16698	0.75	0.26	1.25	3.78	0.00	0.01

Mild_v_Critical	Cardiovascular	OID00402	IL-27	Q8NEV9, Q14213	0.60	0.20	1.00	6.45	0.00	0.01
Mild_v_Critical	Cardiovascular	OID00466	CD4	P01730	0.46	0.16	0.76	5.01	0.00	0.01
Longitudinal_in_Mild	Neurology	OID00374	MANF	P55145	2.98	1.98	3.99	7.38	0.00	0.01
Longitudinal_in_Mild	Inflammation	OID00502	SLAMF1	Q13291	-1.23	-1.67	-0.79	2.50	0.00	0.01
Longitudinal_in_Mild	Cardiovascular	OID00420	CD84	Q9UIB8	0.81	0.52	1.11	4.68	0.00	0.01
Severe_v_Critical	Inflammation	OID00483	IL-17C	Q9P0M4	0.86	0.44	1.28	3.34	0.00	0.01
Longitudinal_in_Mild	Cardiovascular	OID00408	SCF	P21583	1.52	0.96	2.09	9.39	0.00	0.01
Mild_v_Severe	Immune	OID01018	DDX58	O95786	0.94	0.35	1.53	3.15	0.00	0.01
Mild_v_Severe	Neurology	OID00321	RSPO1	Q2MKA7	0.47	0.17	0.76	2.61	0.00	0.01
Control_v_Critical	Neurology	OID00295	CLM-6	Q08708	0.46	0.15	0.76	6.15	0.00	0.01
Control_v_Case	Immune	OID00970	CCL11	P51671	-0.46	-0.80	-0.13	7.22	0.01	0.01
Mild_v_Critical	Inflammation	OID00561	TNFB	P01374	-0.56	-0.93	-0.19	4.63	0.00	0.01
Mild_v_Severe	Cardiovascular	OID00469	PARP-1	P09874	1.03	0.38	1.68	4.40	0.00	0.01
Control_v_Case	Cardiovascular	OID00451	FABP2	P12104	-1.01	-1.73	-0.28	7.69	0.01	0.01
Mild_v_Critical	Immune	OID00966	NTF4	P34130	-0.35	-0.59	-0.12	1.83	0.00	0.01
Control_v_Case	Inflammation	OID00539	CCL28	Q9NRJ3	-0.40	-0.70	-0.11	2.41	0.01	0.01
Control_v_Mild	Neurology	OID00319	ADAM 23	O75077	-0.85	-1.42	-0.29	4.12	0.00	0.01
Control_v_Mild	Immune	OID00961	GALNT3	Q14435	0.74	0.25	1.23	2.77	0.00	0.01
Control_v_Mild	Neurology	OID00355	PDGF-R-alpha	P16234	0.44	0.15	0.73	5.26	0.00	0.01
Control_v_Mild	Neurology	OID00302	PRTG	Q2VWP7	-0.34	-0.57	-0.12	6.59	0.00	0.01
Mild_v_Severe	Neurology	OID00330	WFIKKN1	Q96NZ8	-0.56	-0.91	-0.20	3.52	0.00	0.01
Severe_v_Critical	Neurology	OID00347	FLRT2	O43155	0.37	0.19	0.55	2.23	0.00	0.01
Control_v_Critical	Neurology	OID00339	GZMA	P12544	0.72	0.23	1.21	6.25	0.00	0.01
Mild_v_Critical	Cardiovascular	OID00448	AGRP	O00253	0.68	0.22	1.14	5.77	0.00	0.01
Mild_v_Critical	Neurology	OID00376	CD200R1	Q8TD46	-0.35	-0.59	-0.11	4.24	0.00	0.01
Mild_v_Critical	Cardiovascular	OID00399	TF	P13726	0.33	0.11	0.56	6.07	0.00	0.01
Mild_v_Severe	Cardiovascular	OID00400	IL1RL2	Q9HB29	-0.63	-1.04	-0.22	4.33	0.00	0.01
Mild_v_Critical	Neurology	OID00301	NCAN	O14594	-0.34	-0.58	-0.11	8.54	0.00	0.01
Mild_v_Critical	Immune	OID01010	IFNLR1	Q8IU57	0.47	0.15	0.80	2.85	0.01	0.01
Mild_v_Critical	Cardiovascular	OID00446	LPL	P06858	-0.44	-0.74	-0.14	9.76	0.01	0.01
Mild_v_Severe	Inflammation	OID00561	TNFB	P01374	-0.56	-0.92	-0.20	4.76	0.00	0.01

Control_v_Case	Cardiovascular	OID00399	TF	P13726	0.34	0.09	0.60	6.04	0.01	0.01
Control_v_Mild	Neurology	OID00328	GDF-8	O14793	-0.74	-1.23	-0.24	3.65	0.00	0.01
Mild_v_Critical	Cardiovascular	OID00426	KIM1	Q96D42	1.06	0.33	1.80	8.75	0.01	0.01
Mild_v_Critical	Inflammation	OID00547	LIF	P15018	0.33	0.10	0.56	0.50	0.01	0.01
Control_v_Critical	Neurology	OID00367	SPOCK1	Q08629	0.45	0.14	0.76	2.52	0.01	0.01
Mild_v_Critical	Cardiovascular	OID00425	MERTK	Q12866	0.34	0.11	0.58	6.81	0.01	0.01
Mild_v_Critical	Immune	OID01012	PADI2	Q9Y2J8	0.63	0.20	1.06	1.28	0.01	0.01
Control_v_Critical	Cardiovascular	OID00470	HAOX1	Q9UJM8	1.67	0.51	2.82	6.22	0.01	0.01
Mild_v_Critical	Neurology	OID00331	TMPRSS5	Q9H3S3	-0.40	-0.68	-0.12	2.46	0.01	0.01
Mild_v_Severe	Neurology	OID00338	NTRK2	Q16620	-0.55	-0.91	-0.19	6.17	0.00	0.01
Mild_v_Severe	Inflammation	OID00478	IL7	P13232	0.72	0.25	1.18	2.13	0.00	0.01
Mild_v_Severe	Inflammation	OID00533	Flt3L	P49771	-0.55	-0.91	-0.19	9.02	0.00	0.01
Control_v_Case	Immune	OID00995	KLRD1	Q13241	0.48	0.12	0.84	6.80	0.01	0.01
Mild_v_Critical	Inflammation	OID05547	IFN-gamma	P01579	1.58	0.48	2.68	8.74	0.01	0.01
Mild_v_Severe	Immune	OID01004	DFFA	O00273	0.83	0.28	1.37	4.17	0.00	0.01
Control_v_Mild	Cardiovascular	OID00465	HSP 27	P04792	-0.71	-1.20	-0.23	9.97	0.00	0.01
Control_v_Mild	Inflammation	OID00554	NT-3	P20783	-0.70	-1.18	-0.22	2.33	0.00	0.01
Severe_v_Critical	Inflammation	OID00552	CX3CL1	P78423	0.69	0.34	1.05	4.79	0.00	0.01
Control_v_Mild	Inflammation	OID05548	TNF	P01375	0.65	0.21	1.09	2.84	0.00	0.01
Mild_v_Critical	Immune	OID01013	SIT1	Q9Y3P8	0.83	0.25	1.41	2.57	0.01	0.01
Mild_v_Critical	Neurology	OID00300	SCARB2	Q14108	0.63	0.19	1.07	5.26	0.01	0.01
Mild_v_Critical	Neurology	OID00330	WFIKKN1	Q96NZ8	-0.61	-1.04	-0.18	3.40	0.01	0.01
Mild_v_Critical	Cardiovascular	OID00435	SORT1	Q99523	0.39	0.12	0.67	8.55	0.01	0.01
Control_v_Severe	Neurology	OID00362	NAAA	Q02083	3.14	1.30	4.99	3.45	0.00	0.01
Control_v_Critical	Cardiovascular	OID00469	PARP-1	P09874	1.16	0.34	1.97	5.68	0.01	0.01
Mild_v_Severe	Inflammation	OID00502	SLAMF1	Q13291	0.59	0.20	0.98	2.57	0.00	0.01
Mild_v_Critical	Inflammation	OID00555	TWEAK	O43508	-0.47	-0.80	-0.14	8.44	0.01	0.01
Control_v_Case	Inflammation	OID00505	CCL11	P51671	-0.50	-0.88	-0.12	7.63	0.01	0.01
Mild_v_Severe	Cardiovascular	OID00432	HO-1	P09601	0.59	0.20	0.98	11.81	0.00	0.01
Mild_v_Severe	Neurology	OID00364	PLXNB1	O43157	0.35	0.11	0.58	1.85	0.00	0.01
Control_v_Mild	Neurology	OID00359	JAM-B	P57087	-0.46	-0.77	-0.14	7.92	0.01	0.01
Mild_v_Severe	Cardiovascular	OID00446	LPL	P06858	-0.50	-0.83	-0.16	9.92	0.00	0.01
Mild_v_Critical	Cardiovascular	OID00407	GIF	P27352	-0.83	-1.42	-0.24	6.80	0.01	0.01
Mild_v_Critical	Cardiovascular	OID00461	TNFRSF13B	O14836	0.45	0.13	0.77	9.98	0.01	0.01

Mild_v_Severe	Neurology	OID00366	CLM-1	Q8TDQ1	0.80	0.26	1.33	6.52	0.00	0.01
Control_v_Critical	Cardiovascular	OID00383	SLAMF7	Q9NQ25	0.99	0.29	1.69	3.76	0.01	0.02
Severe_v_Critical	Inflammation	OID00471	IL8	P10145	1.37	0.66	2.08	7.00	0.00	0.02
Severe_v_Critical	Immune	OID01026	ITGB6	P18564	0.51	0.24	0.78	3.06	0.00	0.02
Control_v_Critical	Inflammation	OID00504	MCP-4	Q99616	-1.37	-2.34	-0.40	15.10	0.01	0.02
Control_v_Critical	Inflammation	OID00554	NT-3	P20783	-0.72	-1.24	-0.21	2.22	0.01	0.02
Mild_v_Critical	Neurology	OID00337	CD200	P41217	-0.44	-0.76	-0.13	6.22	0.01	0.02
Mild_v_Critical	Cardiovascular	OID00385	ADAM-TS13	Q76LX8	-0.16	-0.28	-0.05	5.02	0.01	0.02
Control_v_Critical	Neurology	OID00319	ADAM 23	O75077	-0.63	-1.08	-0.18	3.93	0.01	0.02
Severe_v_Critical	Cardiovascular	OID00464	CA5A	P35218	1.16	0.53	1.79	4.60	0.00	0.02
Severe_v_Critical	Cardiovascular	OID00396	TRAIL-R2	O14763	0.84	0.37	1.30	7.73	0.00	0.02
Severe_v_Critical	Immune	OID01027	BTN3A2	P78410	0.76	0.34	1.18	4.01	0.00	0.02
Severe_v_Critical	Neurology	OID00300	SCARB2	Q14108	0.73	0.33	1.12	5.68	0.00	0.02
Severe_v_Critical	Cardiovascular	OID00452	THPO	P40225	0.70	0.31	1.08	4.07	0.00	0.02
Severe_v_Critical	Inflammation	OID00479	OPG	O00300	0.65	0.29	1.01	10.93	0.00	0.02
Severe_v_Critical	Neurology	OID00337	CD200	P41217	0.50	0.23	0.78	5.99	0.00	0.02
Severe_v_Critical	Neurology	OID00355	PDGF-R-alpha	P16234	0.43	0.20	0.66	5.67	0.00	0.02
Severe_v_Critical	Neurology	OID00360	CTSS	P25774	0.32	0.14	0.50	6.15	0.00	0.02
Severe_v_Critical	Cardiovascular	OID00431	PRELP	P51888	0.18	0.08	0.28	8.41	0.00	0.02
Control_v_Case	Cardiovascular	OID00393	IDUA	P35475	-0.43	-0.76	-0.10	5.63	0.01	0.02
Severe_v_Critical	Cardiovascular	OID00459	CTSL1	P07711	0.65	0.28	1.01	9.32	0.00	0.02
Control_v_Case	Cardiovascular	OID00440	CCL3	P10147	0.78	0.18	1.38	6.82	0.01	0.02
Mild_v_Critical	Immune	OID01017	CLEC6A	Q6EIG7	0.60	0.17	1.03	2.75	0.01	0.02
Control_v_Case	Inflammation	OID00533	Flt3L	P49771	-0.47	-0.84	-0.11	9.08	0.01	0.02
Control_v_Case	Inflammation	OID00502	SLAMF1	Q13291	0.45	0.11	0.80	2.62	0.01	0.02
Severe_v_Critical	Immune	OID00953	PRDX3	P30048	0.67	0.29	1.05	0.18	0.00	0.02
Severe_v_Critical	Inflammation	OID00535	CXCL10	P02778	0.62	0.27	0.98	13.04	0.00	0.02
Severe_v_Critical	Inflammation	OID00481	uPA	P00749	0.50	0.21	0.78	10.06	0.00	0.02
Mild_v_Critical	Neurology	OID00295	CLM-6	Q08708	0.36	0.10	0.63	6.18	0.01	0.02
Severe_v_Critical	Cardiovascular	OID00390	IL6	P05231	1.80	0.77	2.83	7.82	0.00	0.02
Control_v_Critical	Inflammation	OID00478	IL7	P13232	-1.07	-1.84	-0.30	3.32	0.01	0.02
Mild_v_Critical	Immune	OID01003	ICA1	Q05084	0.39	0.11	0.68	1.02	0.01	0.02
Control_v_Mild	Neurology	OID00338	NTRK2	Q16620	-0.46	-0.78	-0.14	6.49	0.01	0.02
Control_v_Critical	Neurology	OID00343	GDNFR-alpha-3	O60609	-0.64	-1.10	-0.18	4.75	0.01	0.02

Control_v_Case	Cardiovascular	OID00469	PARP-1	P09874	0.74	0.17	1.31	5.17	0.01	0.02
Control_v_Case	Neurology	OID00330	WFIKKN1	Q96NZ8	-0.40	-0.70	-0.09	3.40	0.01	0.02
Mild_v_Severe	Inflammation	OID00538	SIRT2	Q8IXJ6	1.25	0.40	2.11	3.57	0.01	0.02
Mild_v_Severe	Neurology	OID00349	CLEC10A	Q8IUN9	-0.82	-1.38	-0.26	4.99	0.01	0.02
Mild_v_Severe	Cardiovascular	OID00403	IL-17D	Q8TAD2	-0.29	-0.49	-0.09	2.36	0.01	0.02
Control_v_Case	Inflammation	OID00531	CD5	P06127	-0.39	-0.69	-0.09	5.32	0.01	0.02
Severe_v_Critical	Cardiovascular	OID00398	TIE2	Q02763	0.30	0.13	0.47	7.37	0.00	0.02
Mild_v_Severe	Cardiovascular	OID00412	RAGE	Q15109	0.52	0.16	0.87	13.54	0.01	0.02
Mild_v_Severe	Immune	OID00962	FXYD5	Q96DB9	0.11	0.03	0.18	0.34	0.01	0.02
Control_v_Mild	Cardiovascular	OID00436	CEACAM8	P31997	-0.69	-1.18	-0.20	4.14	0.01	0.02
Mild_v_Severe	Cardiovascular	OID00389	IL-1ra	P18510	0.77	0.24	1.30	5.69	0.01	0.02
Mild_v_Severe	Immune	OID01001	FAM3B	P58499	-0.46	-0.77	-0.14	4.60	0.01	0.02
Control_v_Critical	Neurology	OID00358	DDR1	Q08345	-0.31	-0.54	-0.08	7.32	0.01	0.02
Severe_v_Critical	Cardiovascular	OID00428	TM	P07204	0.47	0.19	0.74	9.67	0.00	0.02
Mild_v_Critical	Immune	OID00962	FXYD5	Q96DB9	0.22	0.06	0.38	0.50	0.01	0.02
Mild_v_Severe	Inflammation	OID05548	TNF	P01375	0.54	0.16	0.92	3.10	0.01	0.02
Control_v_Case	Inflammation	OID00553	TNFRSF9	Q07011	-0.40	-0.72	-0.09	6.67	0.01	0.02
Severe_v_Critical	Immune	OID01015	LAMP3	Q9UQV4	0.66	0.27	1.05	5.92	0.00	0.02
Severe_v_Critical	Cardiovascular	OID00384	PGF	P49763	0.45	0.18	0.72	7.84	0.00	0.02
Control_v_Critical	Inflammation	OID00496	CXCL1	P09341	-1.10	-1.91	-0.29	10.45	0.01	0.02
Mild_v_Critical	Cardiovascular	OID00403	IL-17D	Q8TAD2	-0.24	-0.42	-0.06	2.27	0.01	0.02
Control_v_Case	Cardiovascular	OID00394	TNFRSF11A	Q9Y6Q6	0.45	0.09	0.81	5.95	0.01	0.02
Control_v_Case	Immune	OID00991	TREM1	Q9NP99	0.43	0.09	0.78	2.66	0.01	0.02
Control_v_Critical	Immune	OID00944	CLEC4G	Q6UXB4	0.87	0.23	1.51	3.85	0.01	0.02
Control_v_Critical	Immune	OID00974	LY75	O60449	-0.51	-0.89	-0.13	2.90	0.01	0.02
Control_v_Critical	Inflammation	OID00477	CD244	Q9BZW8	-0.49	-0.85	-0.13	5.86	0.01	0.02
Mild_v_Critical	Neurology	OID00346	SKR3	P37023	0.42	0.11	0.73	7.07	0.01	0.02
Mild_v_Critical	Neurology	OID00348	CPM	P14384	-0.41	-0.72	-0.11	6.41	0.01	0.02
Control_v_Mild	Neurology	OID00367	SPOCK1	Q08629	0.33	0.09	0.57	2.40	0.01	0.02
Longitudinal_in_Mild	Immune	OID01012	PADI2	Q9Y2J8	-1.96	-2.75	-1.18	1.00	0.00	0.02
Severe_v_Critical	Inflammation	OID00550	CASP-8	Q14790	0.77	0.31	1.23	3.89	0.00	0.02
Control_v_Severe	Cardiovascular	OID00404	CXCL1	P09341	-3.62	-5.85	-1.39	11.47	0.00	0.02
Mild_v_Severe	Cardiovascular	OID00436	CEACAM8	P31997	0.59	0.17	1.02	4.19	0.01	0.02
Control_v_Mild	Neurology	OID00357	CDH6	P55285	-0.41	-0.70	-0.11	4.61	0.01	0.02

Control_v_Case	Neurology	OID00337	CD200	P41217	-0.38	-0.69	-0.07	6.21	0.02	0.02
Severe_v_Critical	Immune	OID00965	LILRB4	Q8NHJ6	0.73	0.29	1.17	5.39	0.00	0.02
Control_v_Severe	Immune	OID00950	IRAK1	P51617	-3.48	-5.64	-1.32	4.09	0.00	0.02
Mild_v_Critical	Neurology	OID00375	TN-R	Q92752	-0.56	-0.99	-0.14	4.11	0.01	0.02
Mild_v_Critical	Cardiovascular	OID00467	NEMO	Q9Y6K9	0.84	0.20	1.48	5.02	0.01	0.02
Control_v_Critical	Cardiovascular	OID00422	SERPINA12	Q8IW75	-1.47	-2.59	-0.36	2.76	0.01	0.02
Mild_v_Critical	Neurology	OID00328	GDF-8	O14793	-1.14	-2.01	-0.27	3.10	0.01	0.02
Mild_v_Critical	Immune	OID00945	IRF9	Q00978	0.55	0.13	0.97	1.90	0.01	0.02
Longitudinal_in_Mild	Cardiovascular	OID00421	PAPPA	Q13219	2.54	1.44	3.65	2.49	0.00	0.02
Longitudinal_in_Critical	Cardiovascular	OID00424	DECR1	Q16698	0.69	0.48	0.90	4.50	0.00	0.02
Longitudinal_in_Critical	Immune	OID00941	TPSAB1	Q15661	0.67	0.45	0.90	4.31	0.00	0.02
Longitudinal_in_Critical	Cardiovascular	OID00399	TF	P13726	-0.54	-0.72	-0.36	6.60	0.00	0.02
Control_v_Critical	Immune	OID01002	SH2D1A	O60880	-1.31	-2.31	-0.31	2.73	0.01	0.02
Control_v_Critical	Inflammation	OID00480	LAP TGF-beta-1	P01137	-1.03	-1.82	-0.25	7.16	0.01	0.02
Control_v_Critical	Inflammation	OID00472	VEGFA	P15692	0.90	0.22	1.59	11.73	0.01	0.02
Severe_v_Critical	Neurology	OID00287	NMNAT1	Q9HAN9	0.86	0.33	1.38	4.49	0.00	0.02
Severe_v_Critical	Neurology	OID00320	MATN3	O15232	0.85	0.33	1.38	10.53	0.00	0.02
Severe_v_Critical	Cardiovascular	OID00387	IL-4RA	P24394	0.80	0.31	1.29	2.98	0.00	0.02
Severe_v_Critical	Neurology	OID00310	MSR1	P21757	0.42	0.16	0.68	7.03	0.00	0.02
Severe_v_Critical	Neurology	OID00296	EZR	P15311	0.37	0.14	0.60	4.74	0.00	0.02
Control_v_Critical	Cardiovascular	OID00404	CXCL1	P09341	-1.14	-2.02	-0.27	11.27	0.01	0.02
Severe_v_Critical	Immune	OID00985	CKAP4	Q07065	0.82	0.31	1.33	6.65	0.00	0.02
Severe_v_Critical	Inflammation	OID00527	MMP-10	P09238	0.66	0.25	1.08	9.45	0.00	0.02
Mild_v_Critical	Inflammation	OID00527	MMP-10	P09238	0.45	0.11	0.80	9.31	0.01	0.02
Control_v_Mild	Immune	OID01008	CXCL12	P48061	-0.34	-0.60	-0.09	1.46	0.01	0.02
Control_v_Severe	Cardiovascular	OID00439	CCL17	Q92583	-5.33	-8.77	-1.90	10.27	0.00	0.02
Control_v_Severe	Immune	OID01020	TANK	Q92844	-4.04	-6.62	-1.45	3.16	0.00	0.02
Control_v_Severe	Immune	OID00955	PRDX5	P30044	-3.30	-5.41	-1.18	6.96	0.00	0.02
Control_v_Severe	Cardiovascular	OID00450	GDF-2	Q9UK05	-3.12	-5.09	-1.14	8.78	0.00	0.02
Control_v_Severe	Immune	OID01017	CLEC6A	Q6EIG7	2.73	0.97	4.49	2.19	0.00	0.02
Control_v_Severe	Neurology	OID00306	PLXNB3	Q9ULL4	-2.14	-3.49	-0.78	5.08	0.00	0.02
Control_v_Severe	Inflammation	OID00552	CX3CL1	P78423	-2.01	-3.30	-0.72	4.19	0.00	0.02

Control_v_Severe	Neurology	OID00292	UNC5C	O95185	-1.69	-2.76	-0.62	4.56	0.00	0.02
Control_v_Severe	Immune	OID00951	CLEC4A	Q9UMR7	-1.64	-2.68	-0.59	3.83	0.00	0.02
Control_v_Case	Neurology	OID00329	THY 1	P04216	-0.26	-0.47	-0.05	9.90	0.02	0.02
Control_v_Severe	Cardiovascular	OID00380	ANGPT1	Q15389	-3.44	-5.66	-1.22	9.20	0.00	0.02
Mild_v_Severe	Inflammation	OID01213	DNER	Q8NFT8	-0.35	-0.61	-0.10	8.47	0.01	0.02
Control_v_Case	Neurology	OID00375	TN-R	Q92752	-0.40	-0.72	-0.07	4.15	0.02	0.02
Control_v_Case	Neurology	OID00291	GDNF	P39905	0.29	0.05	0.53	1.99	0.02	0.03
Mild_v_Severe	Immune	OID00966	NTF4	P34130	-0.38	-0.65	-0.10	1.90	0.01	0.03
Control_v_Mild	Neurology	OID00339	GZMA	P12544	0.58	0.15	1.01	6.05	0.01	0.03
Mild_v_Severe	Inflammation	OID00479	OPG	O00300	0.32	0.09	0.56	10.14	0.01	0.03
Control_v_Critical	Cardiovascular	OID00460	hOSCAR	Q8IYS5	0.37	0.08	0.65	10.88	0.01	0.03
Control_v_Critical	Neurology	OID00349	CLEC10A	Q8IUN9	-0.46	-0.82	-0.10	4.87	0.01	0.03
Mild_v_Severe	Neurology	OID05024	MAPT	P10636	0.09	0.02	0.16	0.28	0.01	0.03
Mild_v_Critical	Immune	OID00969	HNMT	P50135	0.64	0.14	1.15	9.53	0.01	0.03
Mild_v_Severe	Cardiovascular	OID00467	NEMO	Q9Y6K9	0.88	0.23	1.53	4.73	0.01	0.03
Mild_v_Critical	Neurology	OID00288	NRP2	O60462	0.13	0.03	0.23	8.25	0.01	0.03
Mild_v_Critical	Inflammation	OID00481	uPA	P00749	0.34	0.07	0.61	9.94	0.01	0.03
Control_v_Case	Neurology	OID00365	TNFRSF21	O75509	-0.24	-0.44	-0.04	8.19	0.02	0.03
Control_v_Mild	Inflammation	OID00493	IL-1 alpha	P01583	-0.19	-0.33	-0.05	0.07	0.01	0.03
Mild_v_Severe	Neurology	OID00363	N2DL-2	Q9BZM5	0.48	0.12	0.83	3.08	0.01	0.03
Control_v_Critical	Inflammation	OID00553	TNFRSF9	Q07011	-0.75	-1.34	-0.16	6.71	0.01	0.03
Control_v_Severe	Neurology	OID00332	CDH3	P22223	-1.68	-2.79	-0.57	7.16	0.00	0.03
Mild_v_Severe	Inflammation	OID00477	CD244	Q9BZW8	-0.43	-0.76	-0.11	5.82	0.01	0.03
Mild_v_Severe	Immune	OID00942	HCLS1	P14317	1.49	0.38	2.60	4.31	0.01	0.03
Mild_v_Critical	Neurology	OID00341	DRAXIN	Q8NBI3	0.70	0.15	1.25	3.64	0.01	0.03
Control_v_Critical	Neurology	OID00332	CDH3	P22223	-0.50	-0.90	-0.11	7.27	0.01	0.03
Mild_v_Critical	Cardiovascular	OID00401	PDGF subunit B	P01127	0.93	0.19	1.68	9.79	0.01	0.03
Mild_v_Severe	Neurology	OID00287	NMNAT1	Q9HAN9	0.83	0.21	1.45	3.18	0.01	0.03
Control_v_Severe	Cardiovascular	OID00386	BOC	Q9BWV1	-1.79	-2.98	-0.60	3.56	0.00	0.03
Control_v_Critical	Inflammation	OID00515	IL-10RB	Q08334	-0.51	-0.91	-0.11	6.24	0.01	0.03
Control_v_Severe	Immune	OID01024	IL5	P05113	3.21	1.07	5.35	1.00	0.00	0.03
Control_v_Critical	Inflammation	OID01213	DNER	Q8NFT8	-0.38	-0.68	-0.08	8.46	0.01	0.03
Longitudinal_in_Critical	Neurology	OID00328	GDF-8	O14793	-0.83	-1.14	-0.52	2.78	0.00	0.03
Control_v_Severe	Cardiovascular	OID00397	PRSS27	Q9BQR3	-1.48	-2.47	-0.49	8.96	0.00	0.03

Severe_v_Critical	Neurology	OID00357	CDH6	P55285	0.36	0.13	0.58	3.88	0.00	0.03
Control_v_Case	Cardiovascular	OID00456	MMP12	P39900	-0.59	-1.09	-0.09	6.80	0.02	0.03
Mild_v_Critical	Inflammation	OID00477	CD244	Q9BZW8	-0.34	-0.61	-0.07	5.79	0.02	0.03
Control_v_Mild	Immune	OID00972	EGLN1	Q9GZT9	-0.54	-0.95	-0.13	1.51	0.01	0.03
Control_v_Severe	Inflammation	OID00550	CASP-8	Q14790	-3.24	-5.42	-1.06	5.92	0.00	0.03
Control_v_Severe	Inflammation	OID00524	IL-24	Q13007	1.43	0.47	2.39	1.08	0.00	0.03
Control_v_Severe	Inflammation	OID00496	CXCL1	P09341	-3.41	-5.72	-1.11	10.64	0.00	0.03
Mild_v_Severe	Inflammation	OID00505	CCL11	P51671	-0.48	-0.85	-0.12	7.60	0.01	0.03
Mild_v_Severe	Neurology	OID00314	RGMB	Q6NW40	-0.49	-0.86	-0.12	5.73	0.01	0.03
Severe_v_Critical	Cardiovascular	OID00406	Gal-9	O00182	0.28	0.10	0.45	9.25	0.00	0.03
Control_v_Critical	Immune	OID01024	IL5	P05113	-0.69	-1.24	-0.14	1.02	0.02	0.03
Severe_v_Critical	Inflammation	OID00482	IL6	P05231	1.71	0.60	2.82	6.93	0.00	0.03
Mild_v_Severe	Immune	OID00957	TRIM5	Q9C035	0.39	0.09	0.69	1.50	0.01	0.03
Control_v_Mild	Inflammation	OID00518	PD-L1	Q9NZQ7	0.59	0.13	1.05	5.50	0.01	0.03
Mild_v_Severe	Neurology	OID00324	gal-8	O00214	0.63	0.15	1.11	5.78	0.01	0.03
Mild_v_Severe	Neurology	OID00347	FLRT2	O43155	-0.23	-0.40	-0.05	2.22	0.01	0.03
Severe_v_Critical	Neurology	OID00293	VWC2	Q2TAL6	-0.38	-0.62	-0.13	5.57	0.00	0.03
Control_v_Critical	Cardiovascular	OID00401	PDGF subunit B	P01127	-0.53	-0.95	-0.10	10.64	0.02	0.03
Mild_v_Critical	Immune	OID00942	HCLS1	P14317	0.93	0.18	1.69	4.67	0.02	0.03
Mild_v_Critical	Inflammation	OID00550	CASP-8	Q14790	0.55	0.10	0.99	3.32	0.02	0.03
Mild_v_Critical	Cardiovascular	OID00470	HAOX1	Q9UJM8	1.36	0.26	2.47	6.63	0.02	0.03
Control_v_Severe	Immune	OID00959	ITGA6	P23229	-3.76	-6.33	-1.20	3.79	0.01	0.03
Severe_v_Critical	Neurology	OID00346	SKR3	P37023	0.40	0.14	0.67	7.31	0.00	0.03
Control_v_Mild	Inflammation	OID00549	MCP-2	P80075	1.24	0.28	2.20	9.53	0.01	0.03
Severe_v_Critical	Immune	OID00970	CCL11	P51671	0.49	0.17	0.82	7.05	0.00	0.03
Control_v_Critical	Neurology	OID00287	NMNAT1	Q9HAN9	1.13	0.21	2.04	4.11	0.02	0.03
Mild_v_Critical	Immune	OID00964	TRIM21	P19474	0.87	0.16	1.58	3.33	0.02	0.03
Mild_v_Severe	Immune	OID00949	CLEC4C	Q8WTT0	-0.50	-0.88	-0.11	3.88	0.01	0.03
Severe_v_Critical	Inflammation	OID00474	MCP-3	P80098	1.60	0.53	2.67	5.56	0.00	0.03
Severe_v_Critical	Inflammation	OID00549	MCP-2	P80075	1.18	0.39	1.97	10.41	0.00	0.03
Severe_v_Critical	Cardiovascular	OID00423	REN	P00797	1.12	0.37	1.87	6.94	0.00	0.03
Severe_v_Critical	Cardiovascular	OID00383	SLAMF7	Q9NQ25	0.82	0.27	1.37	4.42	0.00	0.03
Severe_v_Critical	Neurology	OID00364	PLXNB1	O43157	0.52	0.17	0.86	2.49	0.00	0.03
Severe_v_Critical	Neurology	OID00329	THY 1	P04216	0.41	0.14	0.68	9.81	0.00	0.03

Severe_v_Critical	Neurology	OID00335	Beta-NGF	P01138	0.35	0.11	0.58	1.67	0.00	0.03
Severe_v_Critical	Neurology	OID00315	SIGLEC1	Q9BZZ2	0.41	0.13	0.69	7.22	0.01	0.03
Control_v_Severe	Cardiovascular	OID00399	TF	P13726	-1.63	-2.75	-0.51	6.00	0.01	0.03
Control_v_Severe	Immune	OID00947	IL6	P05231	3.36	1.04	5.68	3.05	0.01	0.03
Control_v_Severe	Immune	OID00957	TRIM5	Q9C035	-2.77	-4.68	-0.86	3.48	0.01	0.03
Mild_v_Critical	Cardiovascular	OID00419	GLO1	Q04760	0.66	0.12	1.19	5.73	0.02	0.03
Mild_v_Critical	Neurology	OID00294	Siglec-9	Q9Y336	0.30	0.05	0.54	5.06	0.02	0.03
Control_v_Mild	Cardiovascular	OID00383	SLAMF7	Q9NQ25	0.88	0.19	1.58	3.19	0.01	0.03
Control_v_Mild	Cardiovascular	OID00470	HAOX1	Q9UJM8	1.81	0.39	3.23	5.36	0.01	0.03
Control_v_Mild	Immune	OID00947	IL6	P05231	0.74	0.16	1.33	2.25	0.01	0.03
Longitudinal_in_Mild	Immune	OID00951	CLEC4A	Q9UMR7	0.74	0.40	1.07	3.72	0.00	0.04
Longitudinal_in_Mild	Immune	OID01020	TANK	Q92844	0.69	0.38	1.00	1.23	0.00	0.04
Control_v_Critical	Neurology	OID00322	HAGH	Q16775	-1.11	-2.03	-0.20	7.04	0.02	0.04
Control_v_Critical	Cardiovascular	OID00418	FS	P19883	0.83	0.15	1.51	10.89	0.02	0.04
Mild_v_Critical	Immune	OID01007	NCR1	O76036	0.39	0.07	0.72	3.55	0.02	0.04
Control_v_Mild	Neurology	OID00356	CTSC	P53634	-0.76	-1.36	-0.15	4.35	0.02	0.04
Control_v_Mild	Cardiovascular	OID00425	MERTK	Q12866	0.44	0.09	0.79	6.39	0.02	0.04
Control_v_Critical	Neurology	OID00314	RGMB	Q6NW40	-0.53	-0.98	-0.09	5.85	0.02	0.04
Control_v_Case	Cardiovascular	OID00435	SORT1	Q99523	-0.25	-0.47	-0.03	8.69	0.03	0.04
Control_v_Severe	Cardiovascular	OID00465	HSP 27	P04792	-1.07	-1.82	-0.31	10.27	0.01	0.04
Control_v_Critical	Immune	OID00943	CNTNAP2	Q9UHC6	-0.46	-0.85	-0.07	1.62	0.02	0.04
Mild_v_Critical	Neurology	OID00315	SIGLEC1	Q9BZZ2	0.41	0.06	0.75	6.79	0.02	0.04
Mild_v_Critical	Cardiovascular	OID00465	HSP 27	P04792	0.50	0.07	0.93	9.90	0.02	0.04
Control_v_Mild	Neurology	OID00317	ADAM 22	Q9P0K1	-0.48	-0.86	-0.09	4.38	0.02	0.04
Mild_v_Severe	Immune	OID01006	FCRL6	Q6DN72	-0.79	-1.42	-0.15	3.45	0.02	0.04
Mild_v_Severe	Immune	OID01020	TANK	Q92844	0.52	0.10	0.95	1.20	0.02	0.04
Mild_v_Severe	Cardiovascular	OID00433	XCL1	P47992	0.31	0.06	0.56	5.05	0.02	0.04
Mild_v_Severe	Immune	OID01019	IL12RB1	P42701	0.31	0.06	0.55	2.39	0.02	0.04
Control_v_Critical	Inflammation	OID00501	IL18	Q14116	0.62	0.09	1.14	9.89	0.02	0.04
Control_v_Severe	Immune	OID01022	KPNA1	P52294	-1.80	-3.08	-0.51	0.87	0.01	0.04
Control_v_Case	Inflammation	OID00551	CCL25	O15444	0.42	0.04	0.80	6.28	0.03	0.04
Severe_v_Critical	Immune	OID00947	IL6	P05231	1.74	0.52	2.97	6.63	0.01	0.04
Control_v_Case	Cardiovascular	OID00468	VEGFD	O43915	-0.25	-0.48	-0.02	7.68	0.03	0.04

Control_v_Mild	Cardiovascular	OID00402	IL-27	Q8NEV9, Q14213	0.56	0.10	1.03	5.91	0.02	0.04
Mild_v_Critical	Cardiovascular	OID00463	LEP	P41159	1.02	0.14	1.89	6.06	0.02	0.04
Severe_v_Critical	Immune	OID01006	FCRL6	Q6DN72	0.71	0.21	1.21	3.33	0.01	0.05
Mild_v_Critical	Cardiovascular	OID00441	MMP7	P09237	0.49	0.07	0.92	9.76	0.02	0.05
Mild_v_Critical	Neurology	OID00326	LAYN	Q6UX15	0.48	0.06	0.90	5.55	0.03	0.05
Mild_v_Critical	Immune	OID00992	CXADR	P78310	0.46	0.06	0.87	2.55	0.02	0.05
Mild_v_Critical	Cardiovascular	OID00447	PRSS8	Q16651	0.29	0.04	0.54	8.91	0.03	0.05
Mild_v_Severe	Immune	OID00940	IRAK4	Q9NWZ3	0.77	0.14	1.40	1.87	0.02	0.05
Control_v_Severe	Immune	OID01018	DDX58	O95786	-2.17	-3.74	-0.60	4.28	0.01	0.05
Control_v_Severe	Immune	OID00945	IRF9	Q00978	-2.15	-3.70	-0.59	2.83	0.01	0.05
Severe_v_Critical	Neurology	OID00377	Nr-CAM	Q92823	0.19	0.05	0.32	9.38	0.01	0.05
Mild_v_Severe	Immune	OID00952	PRDX1	Q06830	0.82	0.14	1.50	1.81	0.02	0.05
Severe_v_Critical	Neurology	OID00324	gal-8	O00214	0.32	0.09	0.55	6.59	0.01	0.05
Mild_v_Severe	Neurology	OID00377	Nr-CAM	Q92823	-0.25	-0.46	-0.04	9.44	0.02	0.05
Longitudinal_in_Critical	Neurology	OID00339	GZMA	P12544	0.83	0.48	1.18	6.49	0.00	0.05
Longitudinal_in_Critical	Neurology	OID00325	BCAN	Q96GW7	-0.67	-0.95	-0.39	3.60	0.00	0.05
Control_v_Case	Neurology	OID00353	MDGA1	Q8NFP4	-0.65	-1.27	-0.04	3.90	0.04	0.05
Control_v_Case	Immune	OID00956	DPP10	Q8N608	-0.34	-0.65	-0.02	1.43	0.04	0.05
Severe_v_Critical	Inflammation	OID00484	MCP-1	P13500	1.16	0.32	1.99	12.99	0.01	0.05
Severe_v_Critical	Immune	OID00996	BACH1	O14867	0.50	0.14	0.86	1.97	0.01	0.05
Severe_v_Critical	Cardiovascular	OID00381	ADM	P35318	0.46	0.13	0.79	9.25	0.01	0.05
Severe_v_Critical	Cardiovascular	OID00425	MERTK	Q12866	0.36	0.10	0.62	7.07	0.01	0.05
Control_v_Case	Neurology	OID00301	NCAN	O14594	0.24	0.01	0.47	8.50	0.04	0.05
Control_v_Case	Cardiovascular	OID00385	ADAM-TS13	Q76LX8	-0.13	-0.25	-0.01	5.01	0.04	0.05
Control_v_Mild	Neurology	OID00301	NCAN	O14594	0.39	0.06	0.71	8.56	0.02	0.05
Control_v_Critical	Inflammation	OID00531	CD5	P06127	-0.56	-1.05	-0.07	5.30	0.03	0.05
Severe_v_Critical	Inflammation	OID00545	FGF-19	O95750	1.14	0.31	1.97	8.18	0.01	0.05
Severe_v_Critical	Neurology	OID00345	TNFRSF12A	Q9NP84	0.77	0.21	1.34	5.54	0.01	0.05
Control_v_Critical	Immune	OID00995	KLRD1	Q13241	0.60	0.07	1.13	6.80	0.03	0.05
Control_v_Critical	Neurology	OID00370	EDA2R	Q9HAV5	-0.60	-1.13	-0.07	4.76	0.03	0.05
Mild_v_Severe	Neurology	OID00290	CADM3	Q8N126	-0.54	-0.99	-0.09	3.13	0.02	0.05
Control_v_Critical	Cardiovascular	OID00434	IL16	Q14005	0.77	0.09	1.46	6.95	0.03	0.05

Control_v_Critical	Neurology	OID00313	EPHB6	O15197	-0.37	-0.70	-0.04	3.93	0.03	0.05
Mild_v_Severe	Immune	OID00982	PLXNA4	Q9HCM2	0.86	0.14	1.58	3.66	0.02	0.05
Severe_v_Critical	Immune	OID00952	PRDX1	Q06830	0.85	0.23	1.48	2.91	0.01	0.05
Mild_v_Severe	Inflammation	OID00476	CDCP1	Q9H5V8	0.60	0.09	1.11	2.93	0.02	0.05
Longitudinal_in_Mild	Inflammation	OID00534	CXCL6	P80162	-4.27	-6.48	-2.05	7.36	0.00	0.05
Longitudinal_in_Mild	Neurology	OID00318	CLEC1B	Q9P126	1.41	0.70	2.12	9.57	0.00	0.05
Mild_v_Critical	Neurology	OID00298	NBL1	P41271	0.08	0.01	0.16	4.89	0.03	0.05
Mild_v_Severe	Cardiovascular	OID00437	PTX3	P26022	0.67	0.10	1.24	4.39	0.02	0.05
Control_v_Critical	Cardiovascular	OID00446	LPL	P06858	-0.56	-1.05	-0.06	9.48	0.03	0.05
Longitudinal_in_Mild	Inflammation	OID00504	MCP-4	Q99616	1.88	0.89	2.87	14.16	0.00	0.05
Mild_v_Critical	Immune	OID00963	TRAF2	Q12933	0.40	0.04	0.77	1.73	0.03	0.05
Mild_v_Severe	Inflammation	OID00534	CXCL6	P80162	1.10	0.16	2.04	7.67	0.02	0.05
Mild_v_Severe	Inflammation	OID00485	IL-17A	Q16552	0.67	0.10	1.24	1.58	0.02	0.05
Control_v_Critical	Inflammation	OID00556	CCL20	P78556	1.28	0.14	2.42	8.55	0.03	0.05
Control_v_Critical	Cardiovascular	OID00454	GT	P51161	0.77	0.08	1.46	2.37	0.03	0.05
Control_v_Severe	Cardiovascular	OID00410	FGF-21	Q9NSA1	-4.76	-8.31	-1.21	6.63	0.01	0.05
Control_v_Critical	Cardiovascular	OID00450	GDF-2	Q9UK05	-0.95	-1.80	-0.10	8.45	0.03	0.05
Control_v_Case	Immune	OID00966	NTF4	P34130	-0.26	-0.50	-0.01	1.84	0.04	0.05
Control_v_Critical	Inflammation	OID00541	EN-RAGE	P80511	0.80	0.08	1.51	3.81	0.03	0.05
Control_v_Mild	Cardiovascular	OID00381	ADM	P35318	0.47	0.07	0.88	8.25	0.02	0.05
Mild_v_Severe	Inflammation	OID00535	CXCL10	P02778	1.25	0.18	2.32	10.75	0.02	0.06
Mild_v_Severe	Immune	OID00983	SH2B3	Q9UQQ2	0.71	0.10	1.31	2.84	0.02	0.06
Control_v_Mild	Neurology	OID00361	N-CDase	Q9NR71	-0.75	-1.39	-0.11	3.67	0.02	0.06
Control_v_Mild	Cardiovascular	OID00413	SOD2	P04179	-0.12	-0.23	-0.02	10.16	0.02	0.06
Mild_v_Critical	Neurology	OID00310	MSR1	P21757	0.39	0.04	0.75	6.84	0.03	0.06
Mild_v_Severe	Immune	OID00946	EDAR	Q9UNE0	-0.49	-0.92	-0.07	1.81	0.02	0.06
Severe_v_Critical	Cardiovascular	OID00424	DECR1	Q16698	0.59	0.15	1.03	4.26	0.01	0.06
Severe_v_Critical	Immune	OID00971	MILR1	Q7Z6M3	0.53	0.13	0.92	3.57	0.01	0.06
Control_v_Critical	Neurology	OID00351	BMP-4	P12644	0.67	0.06	1.29	4.26	0.03	0.06
Severe_v_Critical	Neurology	OID00295	CLM-6	Q08708	0.31	0.08	0.54	6.39	0.01	0.06
Control_v_Mild	Neurology	OID00364	PLXNB1	O43157	-0.38	-0.72	-0.05	1.90	0.02	0.06
Mild_v_Severe	Neurology	OID00359	JAM-B	P57087	-0.41	-0.76	-0.05	7.65	0.03	0.06

Control_v_Severe	Inflammation	OID00494	OSM	P13725	4.29	1.03	7.54	4.69	0.01	0.06
Mild_v_Severe	Neurology	OID00376	CD200R1	Q8TD46	-0.30	-0.57	-0.04	4.28	0.03	0.06
Control_v_Critical	Inflammation	OID00530	CCL23	P55773	0.68	0.06	1.31	10.90	0.03	0.06
Mild_v_Severe	Cardiovascular	OID00381	ADM	P35318	0.37	0.04	0.69	8.49	0.03	0.06
Longitudinal_in_Critical	Inflammation	OID00521	TRANCE	O14788	0.80	0.44	1.17	2.28	0.00	0.06
Severe_v_Critical	Inflammation	OID00476	CDCP1	Q9H5V8	0.67	0.16	1.18	4.12	0.01	0.06
Severe_v_Critical	Immune	OID01019	IL12RB1	P42701	0.32	0.08	0.57	2.99	0.01	0.06
Control_v_Mild	Neurology	OID00314	RGMB	Q6NW40	-0.40	-0.75	-0.05	5.94	0.03	0.06
Control_v_Severe	Cardiovascular	OID00449	HB-EGF	Q99075	-3.19	-5.62	-0.76	7.34	0.01	0.06
Control_v_Mild	Immune	OID01001	FAM3B	P58499	-0.47	-0.87	-0.06	4.81	0.03	0.06
Severe_v_Critical	Neurology	OID00341	DRAXIN	Q8NBI3	0.78	0.18	1.38	3.82	0.01	0.06
Severe_v_Critical	Neurology	OID00311	Alpha-2-MRAP	P30533	0.54	0.12	0.95	8.66	0.01	0.06
Severe_v_Critical	Immune	OID01025	CD83	Q01151	0.30	0.07	0.53	2.90	0.01	0.06
Mild_v_Severe	Immune	OID01002	SH2D1A	O60880	0.53	0.06	1.01	1.75	0.03	0.06
Mild_v_Critical	Immune	OID01000	ARNT	P27540	0.20	0.02	0.39	0.94	0.03	0.06
Mild_v_Severe	Neurology	OID00358	DDR1	Q08345	-0.31	-0.57	-0.04	7.13	0.03	0.06
Control_v_Critical	Inflammation	OID00547	LIF	P15018	-0.21	-0.40	-0.02	0.53	0.03	0.06
Severe_v_Critical	Neurology	OID00363	N2DL-2	Q9BZM5	0.60	0.14	1.06	4.07	0.01	0.06
Mild_v_Severe	Immune	OID00997	PIK3AP1	Q6ZUJ8	0.87	0.10	1.65	2.78	0.03	0.06
Mild_v_Severe	Immune	OID00963	TRAF2	Q12933	0.46	0.05	0.87	1.61	0.03	0.06
Control_v_Mild	Inflammation	OID00498	CCL4	P13236	-0.81	-1.52	-0.09	6.01	0.03	0.06
Control_v_Critical	Cardiovascular	OID00393	IDUA	P35475	-0.65	-1.26	-0.04	5.71	0.04	0.06
Control_v_Critical	Neurology	OID00294	Siglec-9	Q9Y336	0.33	0.02	0.63	5.04	0.04	0.06
Control_v_Case	Cardiovascular	OID00422	SERPINA12	Q8IW75	-0.78	-1.56	0.00	2.99	0.05	0.06
Severe_v_Critical	Cardiovascular	OID00400	IL1RL2	Q9HB29	0.43	0.10	0.77	4.30	0.01	0.07
Control_v_Severe	Cardiovascular	OID00424	DECR1	Q16698	-3.82	-6.78	-0.87	6.94	0.01	0.07
Mild_v_Severe	Neurology	OID00335	Beta-NGF	P01138	0.21	0.02	0.41	1.26	0.03	0.07
Severe_v_Critical	Immune	OID00987	HEXIM1	O94992	0.58	0.13	1.03	5.64	0.01	0.07
Control_v_Critical	Immune	OID00964	TRIM21	P19474	-0.75	-1.46	-0.05	5.13	0.04	0.07
Severe_v_Critical	Cardiovascular	OID00429	VSIG2	Q96IQ7	0.69	0.14	1.23	3.88	0.02	0.07
Severe_v_Critical	Immune	OID00961	GALNT3	Q14435	0.57	0.12	1.03	3.43	0.02	0.07
Severe_v_Critical	Inflammation	OID05548	TNF	P01375	0.52	0.11	0.93	4.03	0.02	0.07
Severe_v_Critical	Immune	OID00956	DPP10	Q8N608	0.49	0.10	0.88	1.33	0.02	0.07
Severe_v_Critical	Inflammation	OID00551	CCL25	O15444	0.47	0.10	0.85	6.34	0.01	0.07

Severe_v_Critical	Neurology	OID00343	GDNFR-alpha-3	O60609	0.35	0.07	0.62	4.24	0.02	0.07
Severe_v_Critical	Neurology	OID00340	G-CSF	P09919	1.11	0.22	1.99	3.76	0.02	0.07
Severe_v_Critical	Neurology	OID00309	SMPD1	P17405	0.32	0.07	0.58	5.21	0.02	0.07
Longitudinal_in_Mild	Immune	OID00955	PRDX5	P30044	1.96	0.90	3.03	4.26	0.00	0.07
Longitudinal_in_Mild	Cardiovascular	OID00392	STK4	Q13043	2.27	0.99	3.55	3.55	0.00	0.07
Longitudinal_in_Critical	Inflammation	OID00533	Flt3L	P49771	0.94	0.49	1.39	8.72	0.00	0.07
Mild_v_Severe	Inflammation	OID00549	MCP-2	P80075	-0.82	-1.57	-0.08	9.51	0.03	0.07
Control_v_Mild	Inflammation	OID00475	GDNF	P39905	0.27	0.03	0.51	2.06	0.03	0.07
Mild_v_Severe	Cardiovascular	OID00454	GT	P51161	-0.59	-1.13	-0.05	2.46	0.03	0.07
Control_v_Critical	Immune	OID00960	CDSN	Q15517	-0.86	-1.67	-0.04	2.94	0.04	0.07
Control_v_Critical	Immune	OID01027	BTN3A2	P78410	0.71	0.03	1.38	3.68	0.04	0.07
Control_v_Case	Cardiovascular	OID00458	PD-L2	Q9BQ51	0.25	0.00	0.49	3.52	0.05	0.07
Severe_v_Critical	Immune	OID00999	STC1	P52823	0.24	0.05	0.43	7.34	0.02	0.07
Control_v_Critical	Inflammation	OID00471	IL8	P10145	0.93	0.04	1.83	6.43	0.04	0.07
Longitudinal_in_Critical	Cardiovascular	OID00436	CEACAM8	P31997	0.50	0.26	0.74	5.01	0.00	0.07
Control_v_Mild	Cardiovascular	OID00468	VEGFD	O43915	-0.28	-0.53	-0.03	7.77	0.03	0.07
Mild_v_Severe	Neurology	OID00323	LXN	Q9BS40	0.19	0.02	0.36	1.69	0.03	0.07
Mild_v_Severe	Immune	OID00945	IRF9	Q00978	0.39	0.03	0.76	1.46	0.03	0.07
Mild_v_Critical	Neurology	OID00339	GZMA	P12544	0.42	0.02	0.82	6.49	0.04	0.07
Control_v_Severe	Inflammation	OID00534	CXCL6	P80162	-3.84	-6.85	-0.83	9.84	0.01	0.07
Control_v_Critical	Neurology	OID00356	CTSC	P53634	-0.45	-0.88	-0.02	4.71	0.04	0.07
Control_v_Severe	Inflammation	OID00512	FGF-21	Q9NSA1	-4.17	-7.46	-0.88	5.36	0.01	0.07
Control_v_Critical	Cardiovascular	OID00414	CTRC	Q99895	-0.88	-1.73	-0.03	9.76	0.04	0.07
Mild_v_Critical	Immune	OID00990	MGMT	P16455	0.76	0.03	1.50	3.83	0.04	0.07
Control_v_Critical	Immune	OID01001	FAM3B	P58499	-0.57	-1.12	-0.02	4.68	0.04	0.07
Severe_v_Critical	Immune	OID00967	KRT19	P08727	0.94	0.17	1.71	5.49	0.02	0.07
Control_v_Severe	Cardiovascular	OID00408	SCF	P21583	-2.48	-4.45	-0.51	9.01	0.02	0.07
Mild_v_Severe	Cardiovascular	OID00456	MMP12	P39900	-0.58	-1.13	-0.04	6.72	0.04	0.07
Mild_v_Severe	Immune	OID00970	CCL11	P51671	-0.46	-0.90	-0.03	7.18	0.04	0.07
Mild_v_Severe	Cardiovascular	OID00466	CD4	P01730	0.26	0.02	0.50	4.85	0.04	0.07
Control_v_Critical	Inflammation	OID00490	CXCL9	Q07325	0.98	0.03	1.94	8.17	0.04	0.07
Control_v_Critical	Neurology	OID00372	NTRK3	Q16288	-0.33	-0.64	-0.01	7.11	0.04	0.07

Control_v_Critical	Neurology	OID00302	PRTG	Q2VWP7	-0.26	-0.52	-0.01	6.48	0.04	0.07
Mild_v_Severe	Inflammation	OID00555	TWEAK	O43508	-0.33	-0.63	-0.02	8.64	0.04	0.07
Severe_v_Critical	Neurology	OID00336	SCARA5	Q6ZMJ2	0.28	0.05	0.51	8.31	0.02	0.08
Mild_v_Critical	Inflammation	OID00536	4E-BP1	Q13541	0.70	0.02	1.38	8.47	0.04	0.08
Longitudinal_in_Critical	Immune	OID00970	CCL11	P51671	-0.58	-0.88	-0.29	6.85	0.00	0.08
Control_v_Mild	Neurology	OID00297	SMOC2	Q9H3U7	-0.47	-0.89	-0.04	7.83	0.03	0.08
Mild_v_Severe	Neurology	OID00320	MATN3	O15232	0.55	0.03	1.08	9.19	0.04	0.08
Mild_v_Severe	Cardiovascular	OID00424	DECR1	Q16698	0.49	0.03	0.96	3.39	0.04	0.08
Control_v_Critical	Inflammation	OID00539	CCL28	Q9NRJ3	-0.54	-1.06	-0.01	2.45	0.05	0.08
Severe_v_Critical	Cardiovascular	OID00433	XCL1	P47992	0.56	0.10	1.03	5.58	0.02	0.08
Mild_v_Severe	Inflammation	OID00475	GDNF	P39905	-0.28	-0.55	-0.02	2.12	0.04	0.08
Longitudinal_in_Critical	Neurology	OID00368	IL12	P29460, P29459	1.06	0.50	1.61	8.34	0.00	0.08
Longitudinal_in_Critical	Immune	OID00967	KRT19	P08727	-0.69	-1.05	-0.33	5.74	0.00	0.08
Mild_v_Critical	Cardiovascular	OID00444	DCN	P07585	0.27	0.01	0.53	4.62	0.05	0.08
Control_v_Severe	Neurology	OID00315	SIGLEC1	Q9BZZ2	2.05	0.40	3.70	6.07	0.02	0.08
Mild_v_Severe	Cardiovascular	OID00404	CXCL1	P09341	1.08	0.05	2.10	9.68	0.04	0.08
Mild_v_Severe	Inflammation	OID00557	ST1A1	P50225	0.63	0.03	1.22	1.52	0.04	0.08
Mild_v_Severe	Cardiovascular	OID00435	SORT1	Q99523	0.34	0.02	0.65	8.43	0.04	0.08
Mild_v_Severe	Cardiovascular	OID00385	ADAM-TS13	Q76LX8	-0.14	-0.27	-0.01	5.06	0.04	0.08
Mild_v_Severe	Cardiovascular	OID00414	CTRC	Q99895	-0.73	-1.43	-0.03	10.31	0.04	0.08
Control_v_Case	Cardiovascular	OID00411	PIgR	P01833	-0.08	-0.16	0.00	5.72	0.06	0.08
Control_v_Mild	Cardiovascular	OID00387	IL-4RA	P24394	0.34	0.02	0.66	2.06	0.04	0.08
Severe_v_Critical	Inflammation	OID00556	CCL20	P78556	1.49	0.25	2.74	9.31	0.02	0.08
Control_v_Mild	Immune	OID00960	CDSN	Q15517	-0.67	-1.30	-0.04	2.93	0.04	0.08
Severe_v_Critical	Immune	OID00964	TRIM21	P19474	0.93	0.15	1.71	3.97	0.02	0.08
Mild_v_Severe	Cardiovascular	OID00448	AGRP	O00253	0.53	0.02	1.03	5.52	0.04	0.08
Control_v_Mild	Neurology	OID00344	PVR	P15151	0.29	0.02	0.56	8.12	0.04	0.08
Control_v_Critical	Cardiovascular	OID00448	AGRP	O00253	0.48	0.00	0.96	5.62	0.05	0.08
Mild_v_Severe	Cardiovascular	OID00382	CD40-L	P29965	1.28	0.04	2.51	4.47	0.04	0.08
Severe_v_Critical	Immune	OID00957	TRIM5	Q9C035	0.64	0.10	1.19	2.43	0.02	0.09
Severe_v_Critical	Neurology	OID00326	LAYN	Q6UX15	0.58	0.09	1.07	5.76	0.02	0.09
Mild_v_Severe	Immune	OID00953	PRDX3	P30048	0.22	0.01	0.44	-0.38	0.04	0.09

Mild_v_Critical	Inflammation	OID00511	LIF-R	P42702	0.22	0.00	0.44	4.40	0.05	0.09
Control_v_Case	Inflammation	OID00475	GDNF	P39905	0.19	-0.01	0.39	2.14	0.07	0.09
Control_v_Severe	Neurology	OID00303	ROBO2	Q9HCK4	-1.35	-2.46	-0.24	5.42	0.02	0.09
Control_v_Critical	Neurology	OID00331	TMPRSS5	Q9H3S3	-0.45	-0.90	0.01	2.54	0.05	0.09
Mild_v_Critical	Cardiovascular	OID00404	CXCL1	P09341	0.88	-0.01	1.76	10.00	0.05	0.09
Control_v_Severe	Immune	OID01003	ICA1	Q05084	-2.55	-4.65	-0.45	3.13	0.02	0.09
Control_v_Mild	Immune	OID01021	ITGA11	Q9UKX5	-0.47	-0.93	-0.02	3.07	0.04	0.09
Control_v_Mild	Cardiovascular	OID00469	PARP-1	P09874	-0.75	-1.46	-0.03	4.75	0.04	0.09
Mild_v_Severe	Immune	OID00955	PRDX5	P30044	1.01	0.02	2.01	3.97	0.05	0.09
Mild_v_Critical	Cardiovascular	OID00432	HO-1	P09601	0.37	-0.01	0.75	12.01	0.05	0.09
Mild_v_Severe	Neurology	OID00365	TNFRSF21	O75509	-0.27	-0.54	0.00	8.17	0.05	0.09
Control_v_Critical	Inflammation	OID00507	FGF-23	Q9GZV9	-0.69	-1.40	0.02	2.29	0.06	0.09
Control_v_Critical	Cardiovascular	OID00415	FGF-23	Q9GZV9	-0.64	-1.30	0.02	3.00	0.06	0.09
Control_v_Critical	Inflammation	OID05548	TNF	P01375	0.58	-0.02	1.18	3.41	0.06	0.09
Control_v_Case	Cardiovascular	OID00418	FS	P19883	0.32	-0.03	0.68	10.84	0.07	0.09
Mild_v_Critical	Neurology	OID00373	LAIR-2	Q6ISS4	0.72	-0.02	1.46	5.32	0.06	0.09
Severe_v_Critical	Inflammation	OID00562	CSF-1	P09603	0.14	0.02	0.26	10.75	0.02	0.09
Severe_v_Critical	Inflammation	OID00501	IL18	Q14116	0.38	0.05	0.70	10.31	0.03	0.09
Control_v_Case	Neurology	OID00364	PLXNB1	O43157	0.28	-0.03	0.58	2.12	0.07	0.10
Severe_v_Critical	Immune	OID01012	PADI2	Q9Y2J8	0.65	0.09	1.22	1.50	0.03	0.10
Mild_v_Severe	Cardiovascular	OID00426	KIM1	Q96D42	0.59	0.00	1.17	8.39	0.05	0.10
Control_v_Mild	Neurology	OID00350	GCP5	P78333	-0.87	-1.71	-0.02	5.01	0.04	0.10
Control_v_Critical	Cardiovascular	OID00455	BNP	P16860	0.72	-0.03	1.47	1.65	0.06	0.10
Control_v_Critical	Neurology	OID00377	Nr-CAM	Q92823	-0.24	-0.49	0.01	9.55	0.06	0.10
Mild_v_Severe	Cardiovascular	OID00464	CA5A	P35218	0.61	0.00	1.22	3.03	0.05	0.10
Mild_v_Severe	Neurology	OID00370	EDA2R	Q9HAV5	0.32	0.00	0.64	4.27	0.05	0.10
Mild_v_Severe	Immune	OID01026	ITGB6	P18564	-0.28	-0.56	0.00	2.88	0.05	0.10
Severe_v_Critical	Immune	OID01009	AREG	P15514	0.80	0.10	1.50	5.56	0.03	0.10
Severe_v_Critical	Immune	OID00980	HSD11B1	P28845	0.49	0.06	0.91	2.86	0.03	0.10
Severe_v_Critical	Neurology	OID00290	CADM3	Q8N126	0.44	0.05	0.82	3.10	0.03	0.10
Control_v_Mild	Neurology	OID00331	TMPRSS5	Q9H3S3	-0.41	-0.81	-0.01	2.75	0.05	0.10
Mild_v_Critical	Immune	OID00980	HSD11B1	P28845	-0.35	-0.72	0.01	3.10	0.06	0.10
Control_v_Severe	Neurology	OID00359	JAM-B	P57087	-1.38	-2.54	-0.21	7.91	0.02	0.10
Control_v_Severe	Immune	OID01026	ITGB6	P18564	-1.24	-2.28	-0.19	2.98	0.02	0.10

Mild_v_Severe	Immune	OID00988	CLEC4D	Q8WXI8	0.50	-0.01	1.00	3.60	0.05	0.10
Control_v_Severe	Neurology	OID00316	CNTN5	O94779	-1.89	-3.49	-0.29	4.70	0.02	0.10
Mild_v_Critical	Inflammation	OID00508	IL-10RA	Q13651	0.21	-0.01	0.44	1.03	0.06	0.10
Mild_v_Severe	Neurology	OID00322	HAGH	Q16775	1.14	-0.03	2.30	5.76	0.06	0.10
Mild_v_Severe	Neurology	OID00378	KYNU	Q16719	0.44	-0.01	0.89	8.62	0.06	0.10
Mild_v_Severe	Inflammation	OID00536	4E-BP1	Q13541	1.04	-0.03	2.12	8.26	0.06	0.10
Control_v_Critical	Neurology	OID00354	IL-5R-alpha	Q01344	1.02	-0.06	2.10	4.53	0.06	0.10
Control_v_Severe	Cardiovascular	OID00445	Dkk-1	O94907	-1.98	-3.66	-0.29	9.52	0.02	0.10
Control_v_Severe	Neurology	OID00298	NBL1	P41271	-0.54	-1.00	-0.08	5.00	0.02	0.10
Mild_v_Critical	Immune	OID00936	PPP1R9B	Q96SB3	0.46	-0.03	0.95	1.74	0.06	0.10
Control_v_Mild	Inflammation	OID00514	IL-15RA	Q13261	0.28	0.00	0.56	1.29	0.05	0.10
Severe_v_Critical	Inflammation	OID00511	LIF-R	P42702	0.32	0.03	0.61	4.55	0.03	0.11
Control_v_Case	Inflammation	OID00523	IL-12B	P29460	0.41	-0.05	0.86	6.63	0.08	0.11
Control_v_Severe	Immune	OID00937	GLB1	P16278	-2.85	-5.30	-0.40	3.03	0.02	0.11
Control_v_Mild	Immune	OID00973	NFATC3	Q12968	-0.47	-0.93	0.00	0.90	0.05	0.11
Control_v_Critical	Neurology	OID00290	CADM3	Q8N126	-0.57	-1.18	0.04	3.37	0.07	0.11
Control_v_Critical	Neurology	OID00308	CD38	P28907	0.52	-0.04	1.08	6.07	0.07	0.11
Control_v_Mild	Immune	OID01013	SIT1	Q9Y3P8	0.44	0.00	0.87	1.99	0.05	0.11
Mild_v_Severe	Inflammation	OID00558	STAMBP	O95630	0.60	-0.02	1.22	4.53	0.06	0.11
Longitudinal_in_Critical	Cardiovascular	OID00463	LEP	P41159	-0.80	-1.26	-0.35	6.08	0.00	0.11
Longitudinal_in_Critical	Immune	OID00937	GLB1	P16278	-0.33	-0.52	-0.14	1.56	0.00	0.11
Control_v_Mild	Cardiovascular	OID00466	CD4	P01730	0.34	0.00	0.69	4.61	0.05	0.11
Mild_v_Critical	Neurology	OID00353	MDGA1	Q8NFP4	-0.64	-1.32	0.04	3.88	0.06	0.11
Control_v_Critical	Neurology	OID00292	UNC5C	O95185	-0.34	-0.70	0.03	4.61	0.07	0.11
Severe_v_Critical	Cardiovascular	OID00444	DCN	P07585	0.50	0.05	0.95	4.74	0.03	0.11
Control_v_Mild	Immune	OID01006	FCRL6	Q6DN72	0.71	-0.01	1.42	3.33	0.05	0.11
Longitudinal_in_Critical	Cardiovascular	OID00401	PDGF subunit B	P01127	0.82	0.34	1.29	10.26	0.00	0.11
Longitudinal_in_Critical	Immune	OID00964	TRIM21	P19474	-0.73	-1.18	-0.28	4.32	0.01	0.11
Longitudinal_in_Critical	Neurology	OID00309	SMPD1	P17405	-0.44	-0.70	-0.17	5.25	0.01	0.11
Longitudinal_in_Critical	Inflammation	OID00511	LIF-R	P42702	-0.40	-0.63	-0.16	4.62	0.01	0.11

Longitudinal_in_Critical	Neurology	OID00296	EZR	P15311	-0.34	-0.55	-0.14	4.92	0.01	0.11
Longitudinal_in_Critical	Cardiovascular	OID00464	CA5A	P35218	1.04	0.40	1.69	5.39	0.01	0.11
Longitudinal_in_Critical	Neurology	OID00372	NTRK3	Q16288	-0.40	-0.65	-0.15	6.64	0.01	0.11
Mild_v_Severe	Inflammation	OID00513	CCL19	Q99731	0.52	-0.03	1.07	9.65	0.06	0.11
Longitudinal_in_Critical	Cardiovascular	OID00439	CCL17	Q92583	1.55	0.54	2.56	8.23	0.01	0.11
Longitudinal_in_Critical	Neurology	OID00333	GFR-alpha-1	P56159	0.82	0.30	1.34	8.22	0.01	0.11
Longitudinal_in_Critical	Immune	OID01021	ITGA11	Q9UKX5	-0.45	-0.75	-0.16	2.28	0.01	0.11
Longitudinal_in_Critical	Neurology	OID00350	GCP5	P78333	0.44	0.15	0.72	4.28	0.01	0.11
Longitudinal_in_Critical	Neurology	OID00323	LXN	Q9BS40	0.35	0.13	0.57	1.64	0.01	0.11
Mild_v_Severe	Neurology	OID00336	SCARA5	Q6ZMJ2	-0.36	-0.75	0.02	8.43	0.06	0.11
Mild_v_Severe	Neurology	OID00355	PDGF-R-alpha	P16234	-0.21	-0.44	0.01	5.32	0.06	0.11
Mild_v_Severe	Cardiovascular	OID00399	TF	P13726	0.18	-0.01	0.37	5.85	0.06	0.11
Longitudinal_in_Critical	Inflammation	OID00510	MMP-1	P03956	1.32	0.45	2.19	14.73	0.01	0.11
Longitudinal_in_Critical	Inflammation	OID00472	VEGFA	P15692	1.00	0.32	1.69	12.19	0.01	0.11
Longitudinal_in_Critical	Inflammation	OID00555	TWEAK	O43508	0.61	0.19	1.03	7.97	0.01	0.11
Longitudinal_in_Critical	Cardiovascular	OID00419	GLO1	Q04760	0.61	0.20	1.02	5.88	0.01	0.11
Longitudinal_in_Critical	Neurology	OID00353	MDGA1	Q8NFP4	0.44	0.14	0.74	3.57	0.01	0.11
Longitudinal_in_Critical	Neurology	OID00316	CNTN5	O94779	-0.32	-0.53	-0.11	3.47	0.01	0.11
Longitudinal_in_Critical	Neurology	OID00305	RGMA	Q96B86	-0.30	-0.50	-0.10	10.26	0.01	0.11
Control_v_Severe	Neurology	OID00300	SCARB2	Q14108	1.02	0.13	1.91	4.68	0.03	0.12
Control_v_Severe	Neurology	OID00346	SKR3	P37023	-1.19	-2.23	-0.14	6.96	0.03	0.12
Severe_v_Critical	Immune	OID00950	IRAK1	P51617	0.40	0.03	0.76	2.44	0.03	0.12
Control_v_Case	Immune	OID00941	TPSAB1	Q15661	-0.35	-0.75	0.06	4.61	0.09	0.12
Severe_v_Critical	Cardiovascular	OID00410	FGF-21	Q9NSA1	1.67	0.13	3.20	7.67	0.03	0.12

Severe_v_Critical	Cardiovascular	OID00470	HAOX1	Q9UJM8	1.17	0.09	2.24	7.27	0.03	0.12
Severe_v_Critical	Inflammation	OID00521	TRANCE	O14788	-0.53	-1.02	-0.04	2.88	0.03	0.12
Control_v_Case	Cardiovascular	OID00462	TGM2	P21980	-0.39	-0.84	0.06	8.24	0.09	0.12
Severe_v_Critical	Cardiovascular	OID00416	SPON2	Q9BUD6	0.15	0.01	0.28	8.68	0.03	0.12
Mild_v_Severe	Neurology	OID00307	CPA2	P48052	-0.74	-1.53	0.05	9.73	0.07	0.12
Mild_v_Severe	Inflammation	OID00487	AXIN1	O15169	0.59	-0.04	1.23	2.60	0.07	0.12
Mild_v_Severe	Immune	OID01027	BTN3A2	P78410	0.37	-0.03	0.76	2.98	0.07	0.12
Control_v_Case	Neurology	OID00376	CD200R1	Q8TD46	-0.19	-0.42	0.03	4.23	0.09	0.12
Mild_v_Critical	Neurology	OID00342	SCARF2	Q96GP6	-0.28	-0.60	0.03	5.89	0.07	0.12
Control_v_Critical	Inflammation	OID00522	HGF	P14210	0.87	-0.09	1.82	9.44	0.08	0.12
Control_v_Mild	Immune	OID00968	ITM2A	O43736	0.61	-0.02	1.24	2.32	0.06	0.12
Control_v_Critical	Cardiovascular	OID00384	PGF	P49763	0.31	-0.03	0.66	7.70	0.08	0.12
Longitudinal_in_Critical	Immune	OID00969	HNMT	P50135	0.53	0.16	0.90	10.67	0.01	0.12
Severe_v_Critical	Cardiovascular	OID00455	BNP	P16860	-1.30	-2.54	-0.07	2.59	0.04	0.12
Severe_v_Critical	Inflammation	OID00541	EN-RAGE	P80511	0.82	0.05	1.59	5.11	0.04	0.12
Severe_v_Critical	Cardiovascular	OID00415	FGF-23	Q9GZV9	0.57	0.04	1.10	3.03	0.04	0.12
Severe_v_Critical	Immune	OID00960	CDSN	Q15517	0.54	0.04	1.04	2.64	0.04	0.12
Severe_v_Critical	Neurology	OID00308	CD38	P28907	0.47	0.03	0.92	6.53	0.04	0.12
Severe_v_Critical	Inflammation	OID05124	CD8A	P01732	0.46	0.02	0.90	9.94	0.04	0.12
Severe_v_Critical	Inflammation	OID00505	CCL11	P51671	0.45	0.03	0.88	7.46	0.04	0.12
Severe_v_Critical	Inflammation	OID00507	FGF-23	Q9GZV9	0.41	0.03	0.80	2.33	0.04	0.12
Severe_v_Critical	Inflammation	OID00475	GDNF	P39905	0.31	0.02	0.60	2.27	0.04	0.12
Severe_v_Critical	Neurology	OID00294	Siglec-9	Q9Y336	0.25	0.02	0.48	5.16	0.04	0.12
Control_v_Critical	Cardiovascular	OID00451	FABP2	P12104	0.93	-0.11	1.97	7.60	0.08	0.12
Severe_v_Critical	Inflammation	OID00513	CCL19	Q99731	0.89	0.04	1.73	11.03	0.04	0.12
Mild_v_Critical	Cardiovascular	OID00452	THPO	P40225	0.38	-0.04	0.81	3.91	0.07	0.12
Control_v_Mild	Immune	OID01026	ITGB6	P18564	-0.40	-0.81	0.02	3.01	0.06	0.12
Mild_v_Severe	Cardiovascular	OID00413	SOD2	P04179	0.08	-0.01	0.16	10.09	0.07	0.12
Control_v_Mild	Immune	OID01000	ARNT	P27540	-0.25	-0.51	0.01	0.82	0.06	0.12
Control_v_Mild	Inflammation	OID05124	CD8A	P01732	0.87	-0.04	1.78	9.61	0.06	0.12
Longitudinal_in_Mild	Inflammation	OID00512	FGF-21	Q9NSA1	-2.45	-4.01	-0.90	5.35	0.01	0.12
Control_v_Critical	Immune	OID00978	PTH1R	Q03431	-0.39	-0.83	0.05	3.93	0.08	0.12
Mild_v_Critical	Immune	OID01002	SH2D1A	O60880	0.45	-0.05	0.94	1.90	0.08	0.12

Severe_v_Critical	Immune	OID00982	PLXNA4	Q9HCM2	-0.64	-1.26	-0.03	3.76	0.04	0.13
Severe_v_Critical	Cardiovascular	OID00407	GIF	P27352	0.61	0.02	1.20	6.47	0.04	0.13
Severe_v_Critical	Immune	OID01004	DFFA	O00273	0.41	0.01	0.81	5.26	0.04	0.13
Severe_v_Critical	Neurology	OID00301	NCAN	O14594	0.24	0.01	0.48	8.42	0.04	0.13
Mild_v_Critical	Neurology	OID00377	Nr-CAM	Q92823	-0.17	-0.35	0.02	9.46	0.08	0.13
Mild_v_Severe	Immune	OID00990	MGMT	P16455	0.88	-0.08	1.83	3.51	0.07	0.13
Mild_v_Severe	Inflammation	OID00496	CXCL1	P09341	0.94	-0.09	1.98	8.97	0.07	0.13
Control_v_Severe	Cardiovascular	OID00457	ACE2	Q9BYF1	-2.16	-4.09	-0.22	3.58	0.03	0.13
Control_v_Severe	Cardiovascular	OID00390	IL6	P05231	2.98	0.29	5.67	3.99	0.03	0.13
Control_v_Severe	Neurology	OID00356	CTSC	P53634	-1.94	-3.68	-0.19	4.69	0.03	0.13
Control_v_Case	Inflammation	OID00527	MMP-10	P09238	0.27	-0.05	0.60	9.24	0.10	0.13
Control_v_Mild	Neurology	OID00340	G-CSF	P09919	0.47	-0.03	0.98	2.94	0.06	0.13
Control_v_Mild	Neurology	OID00308	CD38	P28907	0.43	-0.03	0.88	5.66	0.06	0.13
Control_v_Mild	Cardiovascular	OID00386	BOC	Q9BWV1	-0.40	-0.83	0.02	3.72	0.06	0.13
Longitudinal_in_Critical	Immune	OID00952	PRDX1	Q06830	-1.06	-1.83	-0.30	3.09	0.01	0.13
Longitudinal_in_Critical	Neurology	OID00320	MATN3	O15232	-0.76	-1.30	-0.21	10.66	0.01	0.13
Longitudinal_in_Critical	Immune	OID00980	HSD11B1	P28845	-0.65	-1.12	-0.18	2.70	0.01	0.13
Longitudinal_in_Critical	Neurology	OID00338	NTRK2	Q16620	-0.48	-0.83	-0.14	5.64	0.01	0.13
Control_v_Critical	Inflammation	OID05124	CD8A	P01732	0.67	-0.09	1.44	9.67	0.08	0.13
Mild_v_Severe	Neurology	OID00329	THY 1	P04216	-0.28	-0.59	0.03	9.74	0.07	0.13
Mild_v_Critical	Neurology	OID00312	sFRP-3	Q92765	0.24	-0.03	0.51	5.52	0.08	0.13
Severe_v_Critical	Immune	OID00988	CLEC4D	Q8WXI8	0.59	0.01	1.16	4.46	0.05	0.13
Mild_v_Critical	Neurology	OID00340	G-CSF	P09919	0.63	-0.08	1.35	3.44	0.08	0.13
Mild_v_Severe	Immune	OID00977	CD28	P10747	-0.31	-0.66	0.04	1.72	0.08	0.13
Control_v_Critical	Inflammation	OID00532	CCL3	P10147	0.73	-0.11	1.57	6.47	0.09	0.13
Mild_v_Critical	Neurology	OID00291	GDNF	P39905	0.31	-0.04	0.66	2.09	0.08	0.13
Control_v_Case	Neurology	OID00369	Dkk-4	Q9UBT3	-0.32	-0.71	0.07	3.53	0.11	0.13
Control_v_Case	Cardiovascular	OID00452	THPO	P40225	-0.28	-0.62	0.06	4.08	0.11	0.13
Severe_v_Critical	Cardiovascular	OID00409	IL18	Q14116	0.39	0.01	0.77	10.09	0.05	0.14
Mild_v_Severe	Neurology	OID00311	Alpha-2-MRAP	P30533	0.39	-0.05	0.83	7.91	0.08	0.14
Control_v_Case	Neurology	OID00312	sFRP-3	Q92765	0.19	-0.04	0.42	5.50	0.11	0.14
Control_v_Severe	Cardiovascular	OID00382	CD40-L	P29965	-4.23	-8.11	-0.35	6.42	0.03	0.14

Control_v_Severe	Neurology	OID00326	LAYN	Q6UX15	-1.54	-2.96	-0.12	5.40	0.03	0.14
Control_v_Severe	Inflammation	OID00502	SLAMF1	Q13291	1.45	0.11	2.78	2.54	0.03	0.14
Control_v_Case	Immune	OID00978	PTH1R	Q03431	-0.23	-0.52	0.05	3.96	0.11	0.14
Mild_v_Severe	Neurology	OID00297	SMOC2	Q9H3U7	-0.37	-0.79	0.05	7.61	0.08	0.14
Mild_v_Severe	Neurology	OID00299	EFNA4	P52798	0.22	-0.03	0.48	2.88	0.08	0.14
Severe_v_Critical	Cardiovascular	OID00443	ITGB1BP2	Q9UKP3	-0.42	-0.84	0.00	3.24	0.05	0.14
Control_v_Critical	Neurology	OID00335	Beta-NGF	P01138	0.21	-0.03	0.46	1.47	0.09	0.14
Mild_v_Severe	Neurology	OID00360	CTSS	P25774	0.22	-0.03	0.48	5.71	0.08	0.14
Mild_v_Critical	Neurology	OID00317	ADAM 22	Q9P0K1	-0.30	-0.65	0.05	4.06	0.09	0.14
Severe_v_Critical	Cardiovascular	OID00436	CEACAM8	P31997	0.35	0.00	0.69	4.96	0.05	0.14
Mild_v_Severe	Cardiovascular	OID00434	IL16	Q14005	0.44	-0.06	0.94	6.67	0.08	0.14
Mild_v_Severe	Cardiovascular	OID00394	TNFRSF11A	Q9Y6Q6	0.34	-0.05	0.72	5.67	0.08	0.14
Severe_v_Critical	Cardiovascular	OID00458	PD-L2	Q9BQ51	0.28	0.00	0.56	3.61	0.05	0.14
Severe_v_Critical	Immune	OID01014	MASP1	P48740	0.20	0.00	0.41	1.39	0.05	0.14
Control_v_Mild	Inflammation	OID00522	HGF	P14210	-0.49	-1.02	0.04	8.33	0.07	0.14
Mild_v_Severe	Immune	OID00956	DPP10	Q8N608	-0.35	-0.74	0.05	1.24	0.08	0.14
Severe_v_Critical	Neurology	OID00303	ROBO2	Q9HCK4	0.23	0.00	0.47	4.62	0.05	0.14
Control_v_Mild	Inflammation	OID00513	CCL19	Q99731	0.67	-0.06	1.39	9.44	0.07	0.14
Control_v_Case	Cardiovascular	OID00417	GH	P01241	1.13	-0.28	2.55	7.96	0.11	0.14
Mild_v_Severe	Neurology	OID00340	G-CSF	P09919	-0.43	-0.93	0.06	2.85	0.09	0.14
Longitudinal_in_Critical	Inflammation	OID00498	CCL4	P13236	1.20	0.29	2.11	6.45	0.02	0.14
Longitudinal_in_Critical	Cardiovascular	OID00445	Dkk-1	O94907	0.83	0.20	1.45	9.33	0.02	0.14
Longitudinal_in_Critical	Cardiovascular	OID00412	RAGE	Q15109	-0.47	-0.83	-0.12	14.48	0.02	0.14
Control_v_Case	Immune	OID01012	PADI2	Q9Y2J8	-0.32	-0.72	0.08	1.33	0.12	0.14
Control_v_Severe	Immune	OID00990	MGMT	P16455	-3.05	-5.89	-0.21	6.51	0.04	0.14
Control_v_Critical	Cardiovascular	OID00435	SORT1	Q99523	-0.28	-0.61	0.05	8.86	0.09	0.14
Control_v_Mild	Immune	OID00991	TREM1	Q9NP99	-0.41	-0.85	0.04	2.34	0.07	0.15
Mild_v_Severe	Immune	OID00974	LY75	O60449	-0.40	-0.87	0.06	2.74	0.09	0.15
Control_v_Mild	Cardiovascular	OID00450	GDF-2	Q9UK05	-0.54	-1.13	0.05	9.00	0.07	0.15
Mild_v_Severe	Neurology	OID00334	GM-CSF-R-alpha	P15509	-0.53	-1.16	0.09	5.44	0.09	0.15
Control_v_Critical	Cardiovascular	OID00436	CEACAM8	P31997	0.42	-0.08	0.93	4.63	0.10	0.15
Mild_v_Critical	Inflammation	OID00496	CXCL1	P09341	0.71	-0.12	1.54	9.27	0.09	0.15

Mild_v_Critical	Inflammation	OID00504	MCP-4	Q99616	0.52	-0.09	1.13	14.26	0.09	0.15
Severe_v_Critical	Cardiovascular	OID00394	TNFRSF11A	Q9Y6Q6	0.54	-0.01	1.09	6.40	0.05	0.15
Control_v_Severe	Inflammation	OID00478	IL7	P13232	-2.62	-5.09	-0.15	3.48	0.04	0.15
Mild_v_Critical	Immune	OID00971	MILR1	Q7Z6M3	0.35	-0.06	0.77	3.41	0.09	0.15
Control_v_Critical	Immune	OID01008	CXCL12	P48061	0.21	-0.04	0.45	1.63	0.10	0.15
Mild_v_Severe	Immune	OID00973	NFATC3	Q12968	0.25	-0.04	0.54	0.77	0.09	0.15
Mild_v_Critical	Immune	OID00997	PIK3AP1	Q6ZUJ8	0.47	-0.09	1.03	2.95	0.10	0.15
Control_v_Severe	Inflammation	OID00482	IL6	P05231	2.94	0.15	5.72	3.22	0.04	0.15
Control_v_Critical	Inflammation	OID00551	CCL25	O15444	0.54	-0.11	1.19	6.32	0.10	0.15
Control_v_Critical	Inflammation	OID00476	CDCP1	Q9H5V8	0.56	-0.11	1.22	3.47	0.10	0.15
Mild_v_Severe	Cardiovascular	OID00392	STK4	Q13043	0.62	-0.11	1.36	2.91	0.09	0.15
Mild_v_Critical	Cardiovascular	OID00451	FABP2	P12104	-0.74	-1.61	0.14	7.57	0.10	0.15
Control_v_Mild	Neurology	OID00295	CLM-6	Q08708	0.34	-0.04	0.72	5.90	0.08	0.15
Control_v_Mild	Inflammation	OID00560	ADA	P00813	-0.57	-1.21	0.07	5.85	0.08	0.16
Control_v_Mild	Neurology	OID00320	MATN3	O15232	0.41	-0.05	0.86	8.89	0.08	0.16
Control_v_Mild	Inflammation	OID00539	CCL28	Q9NRJ3	-0.41	-0.86	0.05	2.51	0.08	0.16
Control_v_Mild	Neurology	OID00336	SCARA5	Q6ZMJ2	-0.32	-0.69	0.04	8.63	0.08	0.16
Mild_v_Critical	Inflammation	OID00512	FGF-21	Q9NSA1	1.01	-0.20	2.23	5.97	0.10	0.16
Control_v_Critical	Cardiovascular	OID00438	PSGL-1	Q14242	-0.17	-0.37	0.04	4.52	0.10	0.16
Severe_v_Critical	Neurology	OID00344	PVR	P15151	0.24	-0.01	0.48	8.98	0.06	0.16
Control_v_Mild	Neurology	OID00360	CTSS	P25774	0.25	-0.03	0.53	5.61	0.08	0.16
Control_v_Severe	Immune	OID00953	PRDX3	P30048	-2.55	-5.00	-0.10	1.64	0.04	0.16
Control_v_Critical	Cardiovascular	OID00380	ANGPT1	Q15389	-0.78	-1.73	0.17	8.87	0.11	0.16
Control_v_Critical	Cardiovascular	OID00421	PAPPA	Q13219	-0.63	-1.41	0.14	3.10	0.11	0.16
Longitudinal_in_Critical	Inflammation	OID00504	MCP-4	Q99616	1.12	0.23	2.01	14.33	0.02	0.16
Longitudinal_in_Critical	Neurology	OID00354	IL-5R-alpha	Q01344	-1.08	-1.93	-0.22	4.97	0.02	0.16
Longitudinal_in_Critical	Inflammation	OID00523	IL-12B	P29460	1.07	0.21	1.93	6.67	0.02	0.16
Longitudinal_in_Critical	Cardiovascular	OID00380	ANGPT1	Q15389	0.80	0.15	1.45	8.37	0.02	0.16
Longitudinal_in_Critical	Immune	OID01007	NCR1	O76036	0.69	0.13	1.25	3.88	0.02	0.16
Longitudinal_in_Critical	Inflammation	OID00502	SLAMF1	Q13291	0.62	0.12	1.11	2.73	0.02	0.16

Longitudinal_in_Critical	Neurology	OID00359	JAM-B	P57087	0.46	0.10	0.83	7.62	0.02	0.16
Longitudinal_in_Critical	Cardiovascular	OID00381	ADM	P35318	0.36	0.07	0.65	9.39	0.02	0.16
Longitudinal_in_Critical	Cardiovascular	OID00395	PAR-1	P25116	0.33	0.06	0.59	8.95	0.02	0.16
Mild_v_Critical	Cardiovascular	OID00438	PSGL-1	Q14242	-0.14	-0.31	0.03	4.44	0.10	0.16
Control_v_Severe	Inflammation	OID00490	CXCL9	Q07325	3.38	0.11	6.64	7.66	0.04	0.16
Mild_v_Critical	Neurology	OID00307	CPA2	P48052	-0.52	-1.15	0.11	9.51	0.11	0.16
Severe_v_Critical	Inflammation	OID00528	IL10	P22301	0.48	-0.02	0.98	5.47	0.06	0.16
Control_v_Mild	Cardiovascular	OID00432	HO-1	P09601	0.44	-0.07	0.94	11.58	0.09	0.16
Mild_v_Critical	Neurology	OID00374	MANF	P55145	0.74	-0.17	1.65	7.17	0.11	0.16
Mild_v_Critical	Cardiovascular	OID00434	IL16	Q14005	0.35	-0.08	0.79	6.86	0.11	0.16
Control_v_Mild	Inflammation	OID00523	IL-12B	P29460	0.59	-0.09	1.28	6.52	0.09	0.16
Longitudinal_in_Mild	Immune	OID01026	ITGB6	P18564	1.29	0.43	2.14	2.84	0.01	0.17
Mild_v_Severe	Cardiovascular	OID00441	MMP7	P09237	-0.32	-0.71	0.07	9.48	0.10	0.17
Severe_v_Critical	Immune	OID00974	LY75	O60449	0.30	-0.01	0.61	2.59	0.06	0.17
Longitudinal_in_Critical	Immune	OID01015	LAMP3	Q9UQV4	-0.56	-1.01	-0.10	6.02	0.02	0.17
Mild_v_Critical	Cardiovascular	OID00410	FGF-21	Q9NSA1	1.08	-0.25	2.41	7.38	0.11	0.17
Control_v_Mild	Cardiovascular	OID00451	FABP2	P12104	-0.77	-1.65	0.12	8.03	0.09	0.17
Mild_v_Critical	Neurology	OID00292	UNC5C	O95185	0.19	-0.04	0.43	4.49	0.11	0.17
Control_v_Severe	Cardiovascular	OID00383	SLAMF7	Q9NQ25	2.93	0.07	5.80	3.25	0.04	0.17
Mild_v_Severe	Neurology	OID00339	GZMA	P12544	0.27	-0.06	0.59	6.34	0.10	0.17
Control_v_Severe	Cardiovascular	OID00412	RAGE	Q15109	1.60	0.03	3.17	13.30	0.05	0.17
Control_v_Severe	Neurology	OID00302	PRTG	Q2VWP7	-0.64	-1.27	-0.01	6.52	0.05	0.17
Control_v_Mild	Neurology	OID00352	FcRL2	Q96LA5	0.46	-0.07	0.98	4.95	0.09	0.17
Control_v_Severe	Immune	OID00946	EDAR	Q9UNE0	-2.71	-5.38	-0.05	3.52	0.05	0.17
Control_v_Severe	Inflammation	OID00510	MMP-1	P03956	-2.94	-5.88	-0.01	15.14	0.05	0.17
Control_v_Severe	Inflammation	OID00480	LAP TGF-beta-1	P01137	-2.70	-5.39	-0.01	7.37	0.05	0.17
Control_v_Severe	Inflammation	OID00558	STAMBP	O95630	-2.30	-4.58	-0.02	7.44	0.05	0.17
Control_v_Severe	Cardiovascular	OID00436	CEACAM8	P31997	1.57	0.01	3.14	4.36	0.05	0.17
Control_v_Severe	Immune	OID01021	ITGA11	Q9UKX5	-1.36	-2.72	0.00	2.94	0.05	0.17
Mild_v_Severe	Neurology	OID00309	SMPD1	P17405	0.31	-0.07	0.68	4.70	0.11	0.17

Longitudinal_in_Mild	Cardiovascular	OID00465	HSP 27	P04792	1.37	0.43	2.32	9.98	0.01	0.17
Longitudinal_in_Critical	Immune	OID00968	ITM2A	O43736	-1.93	-3.53	-0.32	2.65	0.03	0.17
Longitudinal_in_Critical	Inflammation	OID00522	HGF	P14210	1.61	0.24	2.99	10.19	0.03	0.17
Longitudinal_in_Critical	Cardiovascular	OID00457	ACE2	Q9BYF1	1.16	0.17	2.15	4.55	0.03	0.17
Longitudinal_in_Critical	Cardiovascular	OID00470	HAOX1	Q9UJM8	0.90	0.13	1.67	8.38	0.03	0.17
Longitudinal_in_Critical	Immune	OID01018	DDX58	O95786	-0.56	-1.03	-0.09	5.10	0.03	0.17
Longitudinal_in_Critical	Cardiovascular	OID00403	IL-17D	Q8TAD2	-0.25	-0.46	-0.04	2.02	0.03	0.17
Mild_v_Severe	Immune	OID01017	CLEC6A	Q6EIG7	0.38	-0.09	0.84	2.46	0.11	0.17
Mild_v_Severe	Inflammation	OID00560	ADA	P00813	0.38	-0.09	0.84	5.59	0.11	0.17
Severe_v_Critical	Inflammation	OID00512	FGF-21	Q9NSA1	1.44	-0.09	2.96	6.23	0.06	0.17
Severe_v_Critical	Neurology	OID00333	GFR-alpha-1	P56159	0.46	-0.03	0.94	8.07	0.07	0.17
Severe_v_Critical	Inflammation	OID00488	TRAIL	P50591	-0.21	-0.44	0.01	7.10	0.06	0.17
Severe_v_Critical	Immune	OID00938	PSIP1	O75475	0.57	-0.04	1.18	4.51	0.07	0.17
Longitudinal_in_Critical	Neurology	OID00365	TNFRSF21	O75509	0.26	0.04	0.49	8.10	0.03	0.17
Control_v_Severe	Inflammation	OID00535	CXCL10	P02778	3.01	-0.02	6.04	10.67	0.05	0.18
Severe_v_Critical	Immune	OID01018	DDX58	O95786	0.86	-0.07	1.79	4.71	0.07	0.18
Severe_v_Critical	Immune	OID00946	EDAR	Q9UNE0	0.36	-0.03	0.75	1.51	0.07	0.18
Severe_v_Critical	Neurology	OID00306	PLXNB3	Q9ULL4	0.25	-0.02	0.53	3.94	0.07	0.18
Control_v_Critical	Cardiovascular	OID00447	PRSS8	Q16651	-0.32	-0.73	0.09	9.00	0.12	0.18
Severe_v_Critical	Immune	OID00993	IL10	P22301	0.52	-0.05	1.09	5.73	0.07	0.18
Severe_v_Critical	Neurology	OID00288	NRP2	O60462	0.11	-0.01	0.23	8.28	0.07	0.18
Mild_v_Severe	Immune	OID01024	IL5	P05113	0.29	-0.07	0.65	1.05	0.11	0.18
Mild_v_Severe	Neurology	OID00351	BMP-4	P12644	-0.33	-0.74	0.08	4.89	0.11	0.18
Mild_v_Critical	Inflammation	OID00514	IL-15RA	Q13261	0.27	-0.07	0.62	1.52	0.12	0.18
Mild_v_Severe	Inflammation	OID00517	IL-18R1	Q13478	0.36	-0.09	0.81	8.62	0.12	0.18
Mild_v_Severe	Cardiovascular	OID00429	VSIG2	Q96IQ7	-0.33	-0.74	0.09	3.66	0.12	0.18
Mild_v_Severe	Neurology	OID00362	NAAA	Q02083	-0.43	-0.97	0.11	3.25	0.12	0.18
Severe_v_Critical	Neurology	OID00366	CLM-1	Q8TDQ1	0.39	-0.04	0.83	7.46	0.07	0.18

Longitudinal_in_Critical	Inflammation	OID00500	SCF	P21583	-0.62	-1.17	-0.08	8.35	0.03	0.18
Longitudinal_in_Critical	Neurology	OID00370	EDA2R	Q9HAV5	0.57	0.07	1.08	4.84	0.03	0.18
Control_v_Severe	Immune	OID01014	MASP1	P48740	-0.96	-1.93	0.02	2.03	0.05	0.18
Severe_v_Critical	Inflammation	OID00503	TGF-alpha	P01135	0.61	-0.06	1.28	4.04	0.07	0.18
Control_v_Mild	Cardiovascular	OID00458	PD-L2	Q9BQ51	0.34	-0.07	0.75	3.47	0.10	0.18
Severe_v_Critical	Cardiovascular	OID00456	MMP12	P39900	0.43	-0.05	0.92	6.44	0.07	0.19
Severe_v_Critical	Inflammation	OID00486	CXCL11	O14625	0.36	-0.04	0.76	10.49	0.08	0.19
Severe_v_Critical	Inflammation	OID05547	IFN-gamma	P01579	1.16	-0.13	2.45	10.02	0.08	0.19
Severe_v_Critical	Inflammation	OID00522	HGF	P14210	0.77	-0.10	1.64	10.18	0.08	0.19
Severe_v_Critical	Immune	OID01013	SIT1	Q9Y3P8	-0.60	-1.27	0.07	3.02	0.08	0.19
Severe_v_Critical	Neurology	OID00370	EDA2R	Q9HAV5	0.44	-0.05	0.94	5.01	0.08	0.19
Severe_v_Critical	Neurology	OID00351	BMP-4	P12644	-0.37	-0.79	0.04	4.30	0.08	0.19
Severe_v_Critical	Neurology	OID00325	BCAN	Q96GW7	0.24	-0.03	0.50	3.71	0.08	0.19
Severe_v_Critical	Neurology	OID00316	CNTN5	O94779	-0.21	-0.44	0.03	3.64	0.08	0.19
Control_v_Mild	Inflammation	OID00561	TNFB	P01374	0.47	-0.10	1.04	4.72	0.10	0.19
Control_v_Critical	Cardiovascular	OID00466	CD4	P01730	0.30	-0.09	0.68	4.83	0.13	0.19
Severe_v_Critical	Immune	OID00994	SRPK2	P78362	0.54	-0.07	1.16	1.60	0.08	0.19
Severe_v_Critical	Immune	OID00945	IRF9	Q00978	0.47	-0.06	1.00	2.31	0.08	0.19
Severe_v_Critical	Neurology	OID00313	EPHB6	O15197	0.24	-0.03	0.51	3.80	0.08	0.19
Longitudinal_in_Critical	Cardiovascular	OID00440	CCL3	P10147	1.14	0.12	2.17	7.43	0.03	0.19
Longitudinal_in_Critical	Immune	OID00976	EIF4G1	Q04637	0.77	0.08	1.45	4.36	0.03	0.19
Longitudinal_in_Critical	Cardiovascular	OID00384	PGF	P49763	0.29	0.03	0.55	7.87	0.03	0.19
Control_v_Mild	Neurology	OID00368	IL12	P29460, P29459	0.72	-0.15	1.60	8.33	0.10	0.19
Control_v_Critical	Inflammation	OID00483	IL-17C	Q9P0M4	0.61	-0.18	1.39	2.85	0.13	0.19
Mild_v_Critical	Inflammation	OID00507	FGF-23	Q9GZV9	0.40	-0.12	0.91	2.18	0.13	0.19
Control_v_Critical	Cardiovascular	OID00452	THPO	P40225	0.44	-0.13	1.01	4.37	0.13	0.19
Control_v_Case	Immune	OID01025	CD83	Q01151	-0.18	-0.44	0.07	2.93	0.15	0.19
Mild_v_Severe	Immune	OID00968	ITM2A	O43736	-0.66	-1.52	0.19	2.16	0.12	0.19
Mild_v_Severe	Immune	OID01016	CLEC7A	Q9BXN2	0.27	-0.08	0.61	3.22	0.12	0.19
Longitudinal_in_Mild	Inflammation	OID00510	MMP-1	P03956	4.08	1.18	6.98	13.96	0.01	0.19

Mild_v_Severe	Cardiovascular	OID00468	VEGFD	O43915	0.13	-0.04	0.29	7.77	0.12	0.19
Severe_v_Critical	Neurology	OID00378	KYNU	Q16719	0.36	-0.05	0.78	9.55	0.08	0.19
Severe_v_Critical	Immune	OID00958	DCTN1	Q14203	0.48	-0.07	1.04	3.65	0.08	0.19
Control_v_Case	Inflammation	OID00515	IL-10RB	Q08334	-0.18	-0.44	0.07	6.20	0.16	0.19
Control_v_Case	Inflammation	OID00526	ARTN	Q5T4W7	0.16	-0.07	0.39	0.96	0.16	0.19
Control_v_Severe	Immune	OID00964	TRIM21	P19474	-2.12	-4.32	0.08	5.32	0.06	0.20
Mild_v_Critical	Inflammation	OID00475	GDNF	P39905	0.18	-0.05	0.41	2.24	0.13	0.20
Mild_v_Critical	Immune	OID00946	EDAR	Q9UNE0	-0.31	-0.71	0.09	1.79	0.13	0.20
Mild_v_Severe	Neurology	OID00298	NBL1	P41271	0.08	-0.02	0.17	4.87	0.13	0.20
Control_v_Mild	Neurology	OID00358	DDR1	Q08345	-0.20	-0.45	0.05	7.29	0.11	0.20
Control_v_Critical	Immune	OID00949	CLEC4C	Q8WTT0	-0.48	-1.11	0.15	3.55	0.13	0.20
Control_v_Mild	Cardiovascular	OID00430	AMBP	P02760	0.17	-0.04	0.39	7.60	0.11	0.20
Mild_v_Critical	Inflammation	OID00520	CXCL5	P42830	0.76	-0.24	1.77	10.23	0.13	0.20
Severe_v_Critical	Neurology	OID00299	EFNA4	P52798	0.38	-0.06	0.82	3.45	0.09	0.20
Longitudinal_in_Critical	Neurology	OID00355	PDGF-R-alpha	P16234	-0.21	-0.40	-0.02	5.66	0.04	0.20
Control_v_Severe	Cardiovascular	OID00405	LOX-1	P78380	1.84	-0.10	3.77	7.25	0.06	0.20
Severe_v_Critical	Inflammation	OID00532	CCL3	P10147	0.58	-0.10	1.26	7.09	0.09	0.21
Severe_v_Critical	Neurology	OID00359	JAM-B	P57087	0.34	-0.06	0.75	7.61	0.09	0.21
Control_v_Case	Neurology	OID00368	IL12	P29460, P29459	0.34	-0.15	0.84	8.40	0.17	0.21
Mild_v_Severe	Inflammation	OID00484	MCP-1	P13500	0.38	-0.13	0.88	11.55	0.14	0.21
Mild_v_Severe	Cardiovascular	OID00465	HSP 27	P04792	0.46	-0.15	1.08	9.71	0.14	0.21
Longitudinal_in_Critical	Cardiovascular	OID00465	HSP 27	P04792	-0.32	-0.61	-0.02	10.34	0.04	0.21
Longitudinal_in_Mild	Cardiovascular	OID00449	HB-EGF	Q99075	1.79	0.48	3.10	5.72	0.01	0.21
Mild_v_Critical	Inflammation	OID00560	ADA	P00813	0.34	-0.12	0.79	5.80	0.14	0.21
Mild_v_Critical	Inflammation	OID00505	CCL11	P51671	-0.20	-0.47	0.07	7.63	0.14	0.21
Severe_v_Critical	Neurology	OID00342	SCARF2	Q96GP6	0.29	-0.05	0.63	5.71	0.10	0.21
Severe_v_Critical	Neurology	OID00314	RGMB	Q6NW40	0.27	-0.05	0.60	5.59	0.10	0.21
Longitudinal_in_Critical	Inflammation	OID00494	OSM	P13725	1.44	0.09	2.78	6.48	0.04	0.21
Mild_v_Critical	Inflammation	OID00499	CD6	P30203	-0.33	-0.77	0.12	6.03	0.14	0.21
Mild_v_Severe	Neurology	OID00369	Dkk-4	Q9UBT3	0.37	-0.13	0.86	3.37	0.14	0.21
Severe_v_Critical	Cardiovascular	OID00391	TNFRSF10A	O00220	0.43	-0.08	0.95	4.23	0.10	0.21

Mild_v_Severe	Cardiovascular	OID00380	ANGPT1	Q15389	0.80	-0.28	1.89	8.34	0.14	0.21
Control_v_Case	Cardiovascular	OID00398	TIE2	Q02763	-0.13	-0.31	0.06	7.36	0.18	0.21
Longitudinal_in_Mild	Inflammation	OID00508	IL-10RA	Q13651	-0.59	-1.03	-0.15	1.11	0.01	0.22
Mild_v_Severe	Inflammation	OID00556	CCL20	P78556	0.60	-0.22	1.42	7.57	0.14	0.22
Mild_v_Severe	Cardiovascular	OID00451	FABP2	P12104	-0.51	-1.20	0.18	7.83	0.14	0.22
Control_v_Critical	Neurology	OID00366	CLM-1	Q8TDQ1	0.49	-0.18	1.17	6.76	0.15	0.22
Control_v_Mild	Neurology	OID00291	GDNF	P39905	0.28	-0.08	0.64	1.89	0.12	0.22
Mild_v_Critical	Neurology	OID00355	PDGF-R-alpha	P16234	0.18	-0.07	0.43	5.52	0.15	0.22
Mild_v_Severe	Inflammation	OID00550	CASP-8	Q14790	0.42	-0.16	1.00	2.83	0.15	0.22
Control_v_Mild	Cardiovascular	OID00444	DCN	P07585	0.24	-0.07	0.55	4.39	0.12	0.22
Control_v_Mild	Cardiovascular	OID00412	RAGE	Q15109	0.38	-0.11	0.87	13.21	0.12	0.22
Control_v_Critical	Cardiovascular	OID00426	KIM1	Q96D42	0.82	-0.31	1.95	8.77	0.15	0.22
Control_v_Mild	Neurology	OID00347	FLRT2	O43155	-0.18	-0.41	0.05	2.33	0.12	0.22
Control_v_Mild	Neurology	OID00362	NAAA	Q02083	-0.41	-0.94	0.12	3.47	0.12	0.22
Mild_v_Critical	Inflammation	OID00558	STAMBP	O95630	0.30	-0.11	0.71	4.67	0.15	0.22
Mild_v_Critical	Neurology	OID00336	SCARA5	Q6ZMJ2	-0.23	-0.55	0.09	8.44	0.15	0.22
Control_v_Critical	Inflammation	OID00560	ADA	P00813	0.51	-0.20	1.21	6.20	0.15	0.22
Mild_v_Critical	Cardiovascular	OID00379	BMP-6	P22004	0.19	-0.07	0.45	4.91	0.15	0.22
Control_v_Critical	Immune	OID00992	CXADR	P78310	0.34	-0.14	0.83	2.52	0.16	0.23
Control_v_Critical	Neurology	OID00364	PLXNB1	O43157	0.32	-0.13	0.78	2.30	0.16	0.23
Longitudinal_in_Critical	Cardiovascular	OID00408	SCF	P21583	-0.69	-1.35	-0.03	8.41	0.04	0.23
Longitudinal_in_Critical	Inflammation	OID00487	AXIN1	O15169	0.51	0.02	1.00	2.97	0.04	0.23
Mild_v_Severe	Immune	OID00944	CLEC4G	Q6UXB4	0.31	-0.12	0.73	3.58	0.15	0.23
Longitudinal_in_Mild	Cardiovascular	OID00437	PTX3	P26022	-1.52	-2.67	-0.37	4.46	0.02	0.23
Control_v_Critical	Immune	OID00996	BACH1	O14867	-0.38	-0.91	0.16	2.57	0.16	0.23
Control_v_Critical	Neurology	OID00365	TNFRSF21	O75509	-0.21	-0.50	0.09	8.20	0.16	0.23
Control_v_Mild	Neurology	OID00377	Nr-CAM	Q92823	-0.16	-0.38	0.05	9.59	0.13	0.23
Mild_v_Severe	Cardiovascular	OID00379	BMP-6	P22004	0.26	-0.11	0.62	4.85	0.16	0.23
Control_v_Mild	Immune	OID00941	TPSAB1	Q15661	-0.48	-1.11	0.15	4.67	0.13	0.23
Severe_v_Critical	Immune	OID00992	CXADR	P78310	0.35	-0.08	0.78	2.78	0.11	0.23
Severe_v_Critical	Cardiovascular	OID00466	CD4	P01730	0.24	-0.06	0.53	5.21	0.11	0.23
Control_v_Case	Neurology	OID00288	NRP2	O60462	0.06	-0.03	0.15	8.24	0.19	0.24

Severe_v_Critical	Cardiovascular	OID00463	LEP	P41159	0.78	-0.19	1.74	6.04	0.11	0.24
Severe_v_Critical	Cardiovascular	OID00448	AGRP	O00253	0.36	-0.09	0.80	6.01	0.11	0.24
Severe_v_Critical	Inflammation	OID00547	LIF	P15018	0.34	-0.08	0.76	0.64	0.11	0.24
Control_v_Mild	Cardiovascular	OID00459	CTSL1	P07711	0.30	-0.10	0.69	7.36	0.14	0.24
Severe_v_Critical	Inflammation	OID00477	CD244	Q9BZW8	0.24	-0.06	0.54	5.58	0.11	0.24
Control_v_Severe	Immune	OID00982	PLXNA4	Q9HCM2	-2.78	-5.82	0.27	5.31	0.07	0.24
Control_v_Mild	Neurology	OID00316	CNTN5	O94779	-0.39	-0.92	0.13	4.87	0.14	0.24
Longitudinal_in_Mild	Neurology	OID00371	LAT	O43561	2.33	0.53	4.12	5.08	0.02	0.24
Longitudinal_in_Mild	Immune	OID01021	ITGA11	Q9UKX5	1.53	0.35	2.72	2.86	0.02	0.24
Mild_v_Critical	Cardiovascular	OID00449	HB-EGF	Q99075	0.44	-0.19	1.08	5.92	0.17	0.24
Mild_v_Critical	Inflammation	OID00526	ARTN	Q5T4W7	0.19	-0.08	0.45	1.02	0.17	0.24
Control_v_Mild	Neurology	OID00341	DRAXIN	Q8NBI3	-0.46	-1.07	0.16	3.27	0.14	0.24
Control_v_Mild	Neurology	OID00348	CPM	P14384	-0.15	-0.35	0.05	6.73	0.14	0.24
Control_v_Critical	Cardiovascular	OID00397	PRSS27	Q9BQR3	-0.34	-0.83	0.15	8.74	0.17	0.24
Longitudinal_in_Critical	Inflammation	OID05124	CD8A	P01732	0.45	0.01	0.90	10.01	0.05	0.24
Control_v_Mild	Inflammation	OID00531	CD5	P06127	-0.36	-0.85	0.13	5.37	0.14	0.24
Control_v_Mild	Cardiovascular	OID00428	TM	P07204	-0.28	-0.65	0.10	9.56	0.14	0.24
Control_v_Mild	Inflammation	OID00477	CD244	Q9BZW8	-0.25	-0.60	0.09	6.01	0.14	0.24
Control_v_Critical	Neurology	OID00310	MSR1	P21757	0.30	-0.14	0.74	6.73	0.17	0.24
Mild_v_Severe	Cardiovascular	OID00449	HB-EGF	Q99075	0.54	-0.24	1.31	5.83	0.17	0.24
Control_v_Critical	Immune	OID00956	DPP10	Q8N608	-0.38	-0.94	0.17	1.56	0.17	0.25
Longitudinal_in_Mild	Inflammation	OID00520	CXCL5	P42830	4.05	0.66	7.44	10.61	0.02	0.25
Longitudinal_in_Mild	Cardiovascular	OID00380	ANGPT1	Q15389	2.70	0.41	4.98	8.00	0.03	0.25
Longitudinal_in_Mild	Cardiovascular	OID00470	HAOX1	Q9UJM8	-2.59	-4.75	-0.42	5.51	0.02	0.25
Longitudinal_in_Mild	Immune	OID01011	DAPP1	Q9UN19	2.25	0.42	4.08	3.16	0.02	0.25
Longitudinal_in_Mild	Inflammation	OID05124	CD8A	P01732	-2.11	-3.89	-0.33	10.30	0.03	0.25
Longitudinal_in_Mild	Cardiovascular	OID00418	FS	P19883	-1.98	-3.59	-0.37	10.80	0.02	0.25
Longitudinal_in_Mild	Neurology	OID00340	G-CSF	P09919	-1.70	-3.03	-0.36	3.08	0.02	0.25

Longitudinal_in_Mild	Immune	OID00978	PTH1R	Q03431	1.31	0.21	2.41	4.22	0.03	0.25
Longitudinal_in_Mild	Inflammation	OID00471	IL8	P10145	0.89	0.15	1.63	4.79	0.02	0.25
Longitudinal_in_Mild	Immune	OID01025	CD83	Q01151	0.66	0.11	1.22	3.00	0.02	0.25
Longitudinal_in_Mild	Cardiovascular	OID00457	ACE2	Q9BYF1	0.45	0.08	0.83	3.22	0.02	0.25
Control_v_Critical	Cardiovascular	OID00442	IgG Fc receptor II-b	P31994	0.56	-0.26	1.39	3.17	0.18	0.25
Longitudinal_in_Mild	Cardiovascular	OID00443	ITGB1BP2	Q9UKP3	2.29	0.33	4.24	3.41	0.03	0.25
Mild_v_Critical	Immune	OID00977	CD28	P10747	-0.23	-0.57	0.11	1.69	0.17	0.25
Severe_v_Critical	Cardiovascular	OID00469	PARP-1	P09874	0.67	-0.18	1.52	5.85	0.12	0.25
Severe_v_Critical	Immune	OID00955	PRDX5	P30044	0.55	-0.15	1.25	5.11	0.12	0.25
Mild_v_Critical	Cardiovascular	OID00456	MMP12	P39900	-0.35	-0.86	0.16	6.71	0.18	0.25
Mild_v_Severe	Inflammation	OID00551	CCL25	O15444	-0.36	-0.88	0.16	6.22	0.17	0.25
Mild_v_Severe	Inflammation	OID05547	IFN-gamma	P01579	0.78	-0.36	1.91	7.72	0.17	0.25
Longitudinal_in_Critical	Inflammation	OID00486	CXCL11	O14625	0.76	0.00	1.53	10.44	0.05	0.25
Longitudinal_in_Critical	Cardiovascular	OID00441	MMP7	P09237	-0.53	-1.06	0.00	9.78	0.05	0.25
Control_v_Critical	Inflammation	OID00481	uPA	P00749	0.28	-0.13	0.69	9.90	0.18	0.25
Control_v_Mild	Neurology	OID00325	BCAN	Q96GW7	-0.32	-0.75	0.12	4.37	0.15	0.25
Control_v_Mild	Inflammation	OID01213	DNER	Q8NFT8	-0.23	-0.55	0.09	8.67	0.15	0.25
Control_v_Mild	Inflammation	OID00503	TGF-alpha	P01135	0.23	-0.09	0.55	2.72	0.15	0.25
Control_v_Critical	Inflammation	OID00510	MMP-1	P03956	-0.72	-1.78	0.35	14.95	0.18	0.25
Mild_v_Critical	Cardiovascular	OID00420	CD84	Q9UIB8	0.19	-0.09	0.46	4.63	0.18	0.25
Control_v_Case	Neurology	OID00292	UNC5C	O95185	-0.15	-0.38	0.09	4.52	0.21	0.26
Mild_v_Severe	Inflammation	OID00507	FGF-23	Q9GZV9	0.21	-0.10	0.51	1.94	0.18	0.26
Severe_v_Critical	Inflammation	OID00542	CD40	P25942	0.41	-0.12	0.95	11.82	0.12	0.26
Severe_v_Critical	Inflammation	OID00534	CXCL6	P80162	0.41	-0.12	0.95	8.49	0.12	0.26
Mild_v_Critical	Immune	OID00943	CNTNAP2	Q9UHC6	-0.20	-0.50	0.10	1.33	0.18	0.26
Mild_v_Critical	Cardiovascular	OID00413	SOD2	P04179	0.05	-0.02	0.12	10.11	0.18	0.26
Longitudinal_in_Critical	Cardiovascular	OID00405	LOX-1	P78380	1.28	-0.02	2.59	7.98	0.05	0.26
Longitudinal_in_Critical	Immune	OID00978	PTH1R	Q03431	-0.40	-0.81	0.01	3.83	0.05	0.26

Control_v_Critical	Cardiovascular	OID00461	TNFRSF13B	O14836	0.34	-0.17	0.86	9.93	0.19	0.26
Control_v_Severe	Inflammation	OID05547	IFN-gamma	P01579	2.74	-0.35	5.83	7.50	0.08	0.26
Longitudinal_in_Critical	Neurology	OID00321	RSPO1	Q2MKA7	0.86	-0.02	1.75	3.15	0.05	0.26
Control_v_Critical	Inflammation	OID00506	TNFSF14	O43557	0.48	-0.24	1.19	6.13	0.19	0.26
Control_v_Critical	Inflammation	OID00542	CD40	P25942	-0.43	-1.09	0.22	11.87	0.19	0.26
Control_v_Mild	Cardiovascular	OID00453	MARCO	Q9UEW3	0.19	-0.08	0.45	6.87	0.16	0.26
Control_v_Case	Neurology	OID00332	CDH3	P22223	-0.16	-0.42	0.10	7.26	0.22	0.26
Control_v_Critical	Immune	OID00991	TREM1	Q9NP99	0.38	-0.20	0.97	2.75	0.19	0.26
Control_v_Severe	Immune	OID01001	FAM3B	P58499	1.35	-0.18	2.87	4.68	0.08	0.26
Mild_v_Critical	Immune	OID01001	FAM3B	P58499	-0.27	-0.69	0.14	4.62	0.19	0.27
Control_v_Mild	Neurology	OID00310	MSR1	P21757	0.42	-0.17	1.00	6.49	0.16	0.27
Longitudinal_in_Mild	Cardiovascular	OID00435	SORT1	Q99523	0.69	0.08	1.30	8.49	0.03	0.27
Longitudinal_in_Mild	Neurology	OID00299	EFNA4	P52798	-0.38	-0.71	-0.05	2.89	0.03	0.27
Longitudinal_in_Mild	Cardiovascular	OID00410	FGF-21	Q9NSA1	-2.47	-4.71	-0.23	6.74	0.03	0.27
Longitudinal_in_Mild	Inflammation	OID00478	IL7	P13232	-2.16	-4.10	-0.22	2.07	0.03	0.27
Longitudinal_in_Mild	Inflammation	OID00533	Flt3L	P49771	0.73	0.07	1.39	9.21	0.03	0.27
Longitudinal_in_Mild	Neurology	OID00373	LAIR-2	Q6ISS4	-0.56	-1.06	-0.06	5.07	0.03	0.27
Longitudinal_in_Mild	Cardiovascular	OID00403	IL-17D	Q8TAD2	0.39	0.04	0.73	2.34	0.03	0.27
Control_v_Severe	Neurology	OID00317	ADAM 22	Q9P0K1	-0.91	-1.94	0.13	4.29	0.08	0.27
Severe_v_Critical	Immune	OID01017	CLEC6A	Q6EIG7	0.32	-0.10	0.75	3.19	0.13	0.27
Longitudinal_in_Mild	Cardiovascular	OID00388	SRC	P12931	2.06	0.15	3.98	6.26	0.04	0.27
Longitudinal_in_Mild	Immune	OID00946	EDAR	Q9UNE0	1.79	0.16	3.42	2.23	0.04	0.27
Longitudinal_in_Mild	Cardiovascular	OID00393	IDUA	P35475	-1.27	-2.48	-0.07	5.77	0.04	0.27
Longitudinal_in_Mild	Immune	OID00958	DCTN1	Q14203	1.03	0.06	2.00	3.19	0.04	0.27
Longitudinal_in_Mild	Neurology	OID00294	Siglec-9	Q9Y336	-0.57	-1.09	-0.04	4.99	0.04	0.27

Longitudinal_in_Mild	Neurology	OID00372	NTRK3	Q16288	0.52	0.03	1.02	7.38	0.04	0.27
Longitudinal_in_Mild	Cardiovascular	OID00436	CEACAM8	P31997	0.40	0.03	0.77	3.99	0.04	0.27
Longitudinal_in_Mild	Cardiovascular	OID00411	PIgR	P01833	0.37	0.02	0.71	5.75	0.04	0.27
Mild_v_Critical	Cardiovascular	OID00400	IL1RL2	Q9HB29	-0.19	-0.48	0.10	4.44	0.19	0.27
Longitudinal_in_Mild	Cardiovascular	OID00401	PDGF subunit B	P01127	2.55	0.10	5.00	9.37	0.04	0.27
Longitudinal_in_Mild	Inflammation	OID00479	OPG	O00300	0.58	0.02	1.14	10.14	0.04	0.27
Longitudinal_in_Mild	Inflammation	OID00552	CX3CL1	P78423	0.36	0.02	0.70	4.40	0.04	0.27
Control_v_Severe	Immune	OID00979	BIRC2	Q13490	-1.51	-3.25	0.22	1.78	0.09	0.27
Control_v_Severe	Cardiovascular	OID00438	PSGL-1	Q14242	-0.72	-1.55	0.11	4.61	0.09	0.27
Severe_v_Critical	Neurology	OID00332	CDH3	P22223	0.38	-0.12	0.87	7.28	0.13	0.27
Control_v_Critical	Neurology	OID00298	NBL1	P41271	-0.08	-0.19	0.04	4.99	0.20	0.27
Mild_v_Severe	Inflammation	OID00520	CXCL5	P42830	0.91	-0.47	2.29	10.24	0.19	0.27
Longitudinal_in_Critical	Neurology	OID00371	LAT	O43561	1.21	-0.08	2.51	6.64	0.06	0.27
Longitudinal_in_Critical	Inflammation	OID00491	CST5	P28325	0.84	-0.05	1.73	6.39	0.06	0.27
Longitudinal_in_Critical	Cardiovascular	OID00421	PAPPA	Q13219	0.72	-0.04	1.49	2.89	0.06	0.27
Longitudinal_in_Critical	Inflammation	OID00506	TNFSF14	O43557	0.64	-0.03	1.31	6.39	0.06	0.27
Longitudinal_in_Critical	Immune	OID00961	GALNT3	Q14435	-0.52	-1.07	0.03	3.89	0.06	0.27
Longitudinal_in_Critical	Neurology	OID00332	CDH3	P22223	0.48	-0.03	0.99	7.35	0.06	0.27
Longitudinal_in_Critical	Immune	OID00996	BACH1	O14867	-0.45	-0.93	0.03	2.20	0.06	0.27
Longitudinal_in_Critical	Neurology	OID00317	ADAM 22	Q9P0K1	0.32	-0.01	0.66	3.92	0.06	0.27
Severe_v_Critical	Immune	OID00968	ITM2A	O43736	0.91	-0.30	2.13	2.38	0.13	0.27
Severe_v_Critical	Neurology	OID00361	N-CDase	Q9NR71	0.44	-0.15	1.03	3.23	0.14	0.27
Severe_v_Critical	Cardiovascular	OID00468	VEGFD	O43915	-0.30	-0.70	0.10	7.52	0.14	0.27
Severe_v_Critical	Immune	OID00973	NFATC3	Q12968	0.29	-0.10	0.67	1.31	0.14	0.27
Severe_v_Critical	Cardiovascular	OID00437	PTX3	P26022	0.27	-0.09	0.63	5.44	0.14	0.27

Severe_v_Critical	Neurology	OID00291	GDNF	P39905	0.27	-0.09	0.62	2.16	0.14	0.27
Control_v_Severe	Immune	OID01004	DFFA	O00273	-2.33	-5.02	0.36	6.63	0.09	0.27
Control_v_Mild	Cardiovascular	OID00403	IL-17D	Q8TAD2	-0.19	-0.46	0.08	2.48	0.16	0.27
Control_v_Mild	Neurology	OID00370	EDA2R	Q9HAV5	-0.26	-0.63	0.11	4.29	0.17	0.27
Control_v_Mild	Inflammation	OID00551	CCL25	O15444	0.47	-0.20	1.15	6.24	0.17	0.27
Severe_v_Critical	Immune	OID01007	NCR1	O76036	0.27	-0.09	0.62	3.77	0.14	0.27
Control_v_Case	Immune	OID00980	HSD11B1	P28845	-0.19	-0.51	0.12	3.05	0.23	0.27
Longitudinal_in_Critical	Immune	OID00987	HEXIM1	O94992	-0.53	-1.09	0.04	5.95	0.06	0.27
Control_v_Mild	Inflammation	OID00472	VEGFA	P15692	-0.42	-1.02	0.19	11.10	0.17	0.28
Control_v_Mild	Neurology	OID00343	GDNFR-alpha-3	O60609	-0.28	-0.68	0.12	5.06	0.17	0.28
Control_v_Mild	Immune	OID00980	HSD11B1	P28845	0.25	-0.11	0.61	3.17	0.17	0.28
Severe_v_Critical	Neurology	OID00321	RSPO1	Q2MKA7	0.47	-0.16	1.10	3.38	0.14	0.28
Control_v_Critical	Inflammation	OID00509	FGF-5	P12034	-0.19	-0.49	0.11	1.06	0.20	0.28
Control_v_Critical	Cardiovascular	OID00398	TIE2	Q02763	-0.17	-0.43	0.09	7.40	0.20	0.28
Severe_v_Critical	Immune	OID00936	PPP1R9B	Q96SB3	0.37	-0.13	0.88	1.91	0.14	0.28
Control_v_Severe	Inflammation	OID00488	TRAIL	P50591	0.87	-0.14	1.88	7.52	0.09	0.28
Mild_v_Critical	Neurology	OID00361	N-CDase	Q9NR71	0.36	-0.20	0.91	3.47	0.20	0.28
Mild_v_Critical	Cardiovascular	OID00398	TIE2	Q02763	0.11	-0.06	0.27	7.38	0.20	0.28
Longitudinal_in_Critical	Inflammation	OID00547	LIF	P15018	0.67	-0.06	1.39	0.69	0.07	0.28
Longitudinal_in_Critical	Neurology	OID00308	CD38	P28907	0.23	-0.02	0.48	6.61	0.07	0.28
Longitudinal_in_Mild	Inflammation	OID00482	IL6	P05231	-1.59	-3.15	-0.03	2.69	0.05	0.28
Longitudinal_in_Mild	Neurology	OID00315	SIGLEC1	Q9BZZ2	-1.38	-2.73	-0.03	6.69	0.05	0.28
Control_v_Mild	Immune	OID00977	CD28	P10747	0.29	-0.13	0.72	1.77	0.17	0.28
Control_v_Severe	Inflammation	OID00500	SCF	P21583	-1.86	-4.03	0.32	8.96	0.09	0.28
Mild_v_Severe	Cardiovascular	OID00427	THBS2	P35442	0.10	-0.05	0.25	5.58	0.19	0.28
Mild_v_Critical	Neurology	OID00314	RGMB	Q6NW40	-0.22	-0.57	0.12	5.75	0.20	0.28
Longitudinal_in_Mild	Cardiovascular	OID00439	CCL17	Q92583	2.00	0.02	3.98	8.38	0.05	0.28
Longitudinal_in_Mild	Cardiovascular	OID00428	TM	P07204	1.57	0.02	3.12	9.59	0.05	0.28
Longitudinal_in_Mild	Immune	OID01008	CXCL12	P48061	-0.33	-0.65	0.00	1.30	0.05	0.28

Control_v_Mild	Immune	OID00944	CLEC4G	Q6UXB4	0.32	-0.15	0.80	3.42	0.18	0.28
Control_v_Case	Cardiovascular	OID00434	IL16	Q14005	0.29	-0.20	0.78	6.84	0.24	0.28
Mild_v_Critical	Immune	OID00978	PTH1R	Q03431	-0.19	-0.49	0.11	4.00	0.21	0.28
Mild_v_Severe	Immune	OID00969	HNMT	P50135	0.32	-0.18	0.81	9.11	0.20	0.29
Mild_v_Critical	Inflammation	OID00554	NT-3	P20783	-0.19	-0.50	0.11	1.91	0.21	0.29
Longitudinal_in_Critical	Cardiovascular	OID00426	KIM1	Q96D42	1.14	-0.13	2.40	8.99	0.07	0.29
Longitudinal_in_Critical	Inflammation	OID00545	FGF-19	O95750	-0.77	-1.63	0.08	8.74	0.07	0.29
Longitudinal_in_Critical	Neurology	OID00334	GM-CSF-R-alpha	P15509	0.73	-0.08	1.54	5.70	0.07	0.29
Longitudinal_in_Critical	Cardiovascular	OID00468	VEGFD	O43915	-0.39	-0.83	0.05	7.57	0.07	0.29
Longitudinal_in_Critical	Inflammation	OID00485	IL-17A	Q16552	0.32	-0.04	0.68	1.42	0.07	0.29
Longitudinal_in_Critical	Cardiovascular	OID00385	ADAM-TS13	Q76LX8	-0.21	-0.45	0.03	4.92	0.07	0.29
Longitudinal_in_Critical	Cardiovascular	OID00402	IL-27	Q8NEV9, Q14213	-0.35	-0.74	0.04	6.95	0.07	0.29
Mild_v_Critical	Immune	OID01005	DCBLD2	Q96PD2	-0.17	-0.44	0.10	7.93	0.21	0.29
Control_v_Mild	Inflammation	OID00491	CST5	P28325	-0.31	-0.77	0.15	6.60	0.18	0.29
Control_v_Mild	Inflammation	OID00481	uPA	P00749	0.24	-0.11	0.59	9.69	0.18	0.29
Control_v_Mild	Inflammation	OID00521	TRANCE	O14788	-0.47	-1.16	0.23	4.66	0.18	0.29
Longitudinal_in_Mild	Immune	OID00977	CD28	P10747	3.15	-0.11	6.40	2.31	0.06	0.29
Longitudinal_in_Mild	Neurology	OID00328	GDF-8	O14793	2.90	-0.03	5.84	3.58	0.05	0.29
Longitudinal_in_Mild	Cardiovascular	OID00445	Dkk-1	O94907	1.64	-0.06	3.35	8.15	0.06	0.29
Longitudinal_in_Mild	Cardiovascular	OID00390	IL6	P05231	-1.53	-3.13	0.07	3.53	0.06	0.29
Longitudinal_in_Mild	Neurology	OID00350	GCP5	P78333	1.10	-0.02	2.23	4.88	0.05	0.29
Longitudinal_in_Mild	Immune	OID00960	CDSN	Q15517	1.08	-0.06	2.21	2.77	0.06	0.29
Longitudinal_in_Mild	Immune	OID00938	PSIP1	O75475	-0.88	-1.80	0.04	2.60	0.06	0.29
Longitudinal_in_Mild	Immune	OID00950	IRAK1	P51617	0.75	-0.02	1.52	1.88	0.05	0.29

Longitudinal_in_Mild	Neurology	OID00304	CRTAM	O95727	-0.72	-1.48	0.04	5.48	0.06	0.29
Longitudinal_in_Mild	Immune	OID00956	DPP10	Q8N608	0.66	-0.01	1.34	1.44	0.05	0.29
Longitudinal_in_Mild	Cardiovascular	OID00395	PAR-1	P25116	0.62	-0.03	1.27	8.71	0.06	0.29
Longitudinal_in_Mild	Neurology	OID00367	SPOCK1	Q08629	-0.55	-1.14	0.03	2.58	0.06	0.29
Longitudinal_in_Mild	Inflammation	OID00509	FGF-5	P12034	0.39	-0.01	0.78	1.10	0.06	0.29
Mild_v_Critical	Immune	OID00958	DCTN1	Q14203	0.37	-0.22	0.95	3.35	0.21	0.29
Mild_v_Severe	Immune	OID00958	DCTN1	Q14203	0.37	-0.21	0.95	3.01	0.20	0.29
Control_v_Case	Inflammation	OID00506	TNFSF14	O43557	0.34	-0.24	0.93	5.55	0.24	0.29
Severe_v_Critical	Cardiovascular	OID00414	CTRC	Q99895	-0.53	-1.26	0.20	9.41	0.15	0.29
Mild_v_Severe	Neurology	OID00332	CDH3	P22223	-0.20	-0.53	0.12	7.23	0.20	0.29
Severe_v_Critical	Neurology	OID00367	SPOCK1	Q08629	0.18	-0.07	0.43	2.65	0.15	0.29
Control_v_Mild	Cardiovascular	OID00393	IDUA	P35475	-0.30	-0.74	0.15	5.77	0.19	0.29
Control_v_Mild	Cardiovascular	OID00400	IL1RL2	Q9HB29	0.26	-0.13	0.65	4.43	0.19	0.29
Longitudinal_in_Critical	Inflammation	OID00532	CCL3	P10147	1.05	-0.15	2.26	7.11	0.08	0.29
Longitudinal_in_Critical	Inflammation	OID00476	CDCP1	Q9H5V8	0.72	-0.10	1.55	4.11	0.08	0.29
Longitudinal_in_Critical	Neurology	OID00312	sFRP-3	Q92765	0.27	-0.04	0.59	5.71	0.08	0.29
Control_v_Mild	Inflammation	OID00490	CXCL9	Q07325	0.64	-0.32	1.61	7.24	0.19	0.29
Longitudinal_in_Mild	Neurology	OID00362	NAAA	Q02083	-1.25	-2.58	0.09	3.33	0.06	0.29
Longitudinal_in_Mild	Cardiovascular	OID00446	LPL	P06858	0.86	-0.07	1.78	10.01	0.07	0.29
Longitudinal_in_Mild	Cardiovascular	OID00456	MMP12	P39900	0.85	-0.06	1.76	7.04	0.06	0.29
Longitudinal_in_Mild	Inflammation	OID00522	HGF	P14210	0.61	-0.05	1.26	8.19	0.06	0.29
Severe_v_Critical	Immune	OID00978	PTH1R	Q03431	0.23	-0.09	0.55	3.84	0.15	0.29
Mild_v_Critical	Inflammation	OID00485	IL-17A	Q16552	0.30	-0.19	0.79	1.56	0.22	0.30
Control_v_Mild	Cardiovascular	OID00440	CCL3	P10147	-0.50	-1.27	0.26	6.37	0.19	0.30
Control_v_Mild	Immune	OID00971	MILR1	Q7Z6M3	0.30	-0.16	0.76	2.98	0.19	0.30
Control_v_Mild	Neurology	OID00329	THY 1	P04216	-0.21	-0.53	0.11	9.96	0.19	0.30

Longitudinal_in_Critical	Neurology	OID00301	NCAN	O14594	-0.30	-0.66	0.05	8.47	0.08	0.30
Mild_v_Critical	Cardiovascular	OID00415	FGF-23	Q9GZV9	0.32	-0.20	0.85	2.97	0.22	0.30
Mild_v_Severe	Neurology	OID00374	MANF	P55145	0.80	-0.48	2.08	6.98	0.21	0.30
Longitudinal_in_Critical	Inflammation	OID00509	FGF-5	P12034	0.24	-0.04	0.52	0.84	0.08	0.30
Control_v_Severe	Immune	OID00999	STC1	P52823	1.50	-0.31	3.32	5.91	0.10	0.30
Control_v_Severe	Immune	OID00992	CXADR	P78310	-1.37	-3.02	0.28	2.30	0.10	0.30
Control_v_Case	Immune	OID01007	NCR1	O76036	0.16	-0.12	0.44	3.53	0.26	0.30
Longitudinal_in_Critical	Cardiovascular	OID00414	CTRC	Q99895	-0.30	-0.66	0.05	9.26	0.08	0.30
Control_v_Case	Immune	OID01016	CLEC7A	Q9BXN2	0.20	-0.15	0.55	3.23	0.26	0.31
Control_v_Mild	Inflammation	OID00515	IL-10RB	Q08334	-0.26	-0.67	0.14	6.14	0.20	0.31
Control_v_Severe	Neurology	OID00343	GDNFR-alpha-3	O60609	-0.95	-2.09	0.20	4.89	0.10	0.31
Mild_v_Critical	Neurology	OID00306	PLXNB3	Q9ULL4	0.23	-0.15	0.61	3.95	0.23	0.31
Severe_v_Critical	Cardiovascular	OID00412	RAGE	Q15109	0.25	-0.11	0.61	14.26	0.16	0.31
Control_v_Mild	Cardiovascular	OID00462	TGM2	P21980	-0.41	-1.05	0.23	8.25	0.20	0.31
Control_v_Mild	Inflammation	OID00483	IL-17C	Q9P0M4	0.44	-0.25	1.13	2.27	0.21	0.31
Control_v_Mild	Cardiovascular	OID00447	PRSS8	Q16651	-0.20	-0.52	0.12	8.87	0.21	0.31
Mild_v_Severe	Immune	OID00961	GALNT3	Q14435	-0.29	-0.78	0.19	2.96	0.22	0.31
Mild_v_Severe	Immune	OID00995	KLRD1	Q13241	0.37	-0.24	0.98	6.80	0.22	0.31
Mild_v_Severe	Neurology	OID00313	EPHB6	O15197	-0.21	-0.55	0.13	3.80	0.22	0.31
Mild_v_Critical	Neurology	OID00369	Dkk-4	Q9UBT3	0.28	-0.19	0.74	3.44	0.24	0.31
Mild_v_Critical	Neurology	OID00362	NAAA	Q02083	-0.27	-0.73	0.18	3.25	0.23	0.31
Control_v_Critical	Cardiovascular	OID00407	GIF	P27352	0.49	-0.32	1.30	6.81	0.23	0.31
Mild_v_Severe	Cardiovascular	OID00443	ITGB1BP2	Q9UKP3	0.57	-0.37	1.52	2.86	0.23	0.31
Control_v_Severe	Inflammation	OID00515	IL-10RB	Q08334	-1.00	-2.22	0.23	6.23	0.11	0.32
Control_v_Mild	Cardiovascular	OID00418	FS	P19883	0.28	-0.17	0.73	10.75	0.21	0.32
Control_v_Critical	Immune	OID00966	NTF4	P34130	-0.25	-0.66	0.17	1.80	0.24	0.32
Mild_v_Critical	Cardiovascular	OID00421	PAPPA	Q13219	0.32	-0.22	0.86	2.88	0.24	0.32
Mild_v_Critical	Neurology	OID00365	TNFRSF21	O75509	-0.14	-0.39	0.10	8.16	0.24	0.32
Mild_v_Critical	Cardiovascular	OID00468	VEGFD	O43915	-0.13	-0.34	0.09	7.59	0.24	0.32
Longitudinal_in_Mild	Neurology	OID00347	FLRT2	O43155	0.73	-0.09	1.54	2.32	0.07	0.32
Longitudinal_in_Mild	Cardiovascular	OID00399	TF	P13726	0.48	-0.06	1.01	5.79	0.07	0.32

Control_v_Critical	Immune	OID01025	CD83	Q01151	-0.19	-0.51	0.13	2.97	0.24	0.32
Control_v_Severe	Cardiovascular	OID00461	TNFRSF13B	O14836	-1.39	-3.11	0.33	9.78	0.11	0.32
Control_v_Severe	Cardiovascular	OID00411	PIgR	P01833	-0.44	-0.99	0.11	5.74	0.11	0.32
Severe_v_Critical	Immune	OID01022	KPNA1	P52294	0.06	-0.03	0.14	-0.10	0.17	0.32
Mild_v_Critical	Neurology	OID00302	PRTG	Q2VWP7	-0.09	-0.25	0.07	6.43	0.25	0.32
Longitudinal_in_Mild	Cardiovascular	OID00467	NEMO	Q9Y6K9	1.10	-0.14	2.35	4.87	0.08	0.32
Longitudinal_in_Mild	Immune	OID01013	SIT1	Q9Y3P8	-0.84	-1.79	0.11	2.14	0.08	0.32
Control_v_Mild	Neurology	OID00303	ROBO2	Q9HCK4	-0.28	-0.73	0.17	5.63	0.22	0.32
Control_v_Mild	Immune	OID00949	CLEC4C	Q8WTT0	0.27	-0.16	0.70	3.96	0.22	0.33
Longitudinal_in_Critical	Cardiovascular	OID00467	NEMO	Q9Y6K9	0.68	-0.14	1.51	5.34	0.09	0.33
Longitudinal_in_Critical	Inflammation	OID00542	CD40	P25942	0.35	-0.07	0.77	11.84	0.09	0.33
Control_v_Mild	Cardiovascular	OID00427	THBS2	P35442	0.12	-0.07	0.31	5.52	0.22	0.33
Control_v_Mild	Cardiovascular	OID00407	GIF	P27352	0.53	-0.33	1.40	6.98	0.22	0.33
Control_v_Critical	Neurology	OID00338	NTRK2	Q16620	-0.18	-0.48	0.13	6.19	0.25	0.33
Longitudinal_in_Mild	Inflammation	OID01213	DNER	Q8NFT8	0.26	-0.04	0.56	8.67	0.08	0.34
Longitudinal_in_Critical	Inflammation	OID00553	TNFRSF9	Q07011	1.10	-0.26	2.45	6.64	0.10	0.34
Longitudinal_in_Critical	Neurology	OID00287	NMNAT1	Q9HAN9	0.54	-0.13	1.20	4.75	0.10	0.34
Longitudinal_in_Critical	Cardiovascular	OID00437	PTX3	P26022	-0.37	-0.83	0.09	5.76	0.10	0.34
Longitudinal_in_Critical	Immune	OID01027	BTN3A2	P78410	0.31	-0.07	0.69	4.29	0.10	0.34
Control_v_Critical	Neurology	OID00334	GM-CSF-R-alpha	P15509	0.46	-0.35	1.27	5.53	0.26	0.34
Control_v_Critical	Neurology	OID00325	BCAN	Q96GW7	-0.26	-0.72	0.20	4.14	0.26	0.34
Control_v_Critical	Cardiovascular	OID00462	TGM2	P21980	-0.37	-1.01	0.28	8.47	0.26	0.34
Mild_v_Severe	Neurology	OID00373	LAIR-2	Q6ISS4	0.55	-0.40	1.49	5.15	0.25	0.34
Mild_v_Critical	Cardiovascular	OID00431	PRELP	P51888	0.09	-0.07	0.24	8.37	0.26	0.34
Longitudinal_in_Critical	Neurology	OID00362	NAAA	Q02083	0.53	-0.13	1.19	3.27	0.10	0.34
Longitudinal_in_Critical	Neurology	OID00347	FLRT2	O43155	0.16	-0.04	0.36	2.18	0.10	0.34
Severe_v_Critical	Cardiovascular	OID00393	IDUA	P35475	0.32	-0.16	0.80	5.38	0.18	0.34

Mild_v_Severe	Cardiovascular	OID00411	PIgR	P01833	-0.04	-0.12	0.03	5.69	0.25	0.34
Longitudinal_in_Critical	Inflammation	OID00480	LAP TGF-beta-1	P01137	0.58	-0.16	1.31	6.63	0.11	0.35
Longitudinal_in_Critical	Inflammation	OID00528	IL10	P22301	0.46	-0.13	1.06	5.73	0.11	0.35
Longitudinal_in_Critical	Inflammation	OID00561	TNFB	P01374	0.40	-0.11	0.92	4.30	0.11	0.35
Longitudinal_in_Critical	Inflammation	OID00508	IL-10RA	Q13651	0.22	-0.06	0.50	0.98	0.11	0.35
Severe_v_Critical	Immune	OID00969	HNMT	P50135	0.43	-0.22	1.07	10.07	0.19	0.35
Mild_v_Severe	Neurology	OID00319	ADAM 23	O75077	-0.33	-0.91	0.25	3.87	0.25	0.35
Control_v_Case	Neurology	OID00327	NEP	P08473	0.34	-0.30	0.99	2.68	0.29	0.35
Control_v_Severe	Inflammation	OID00538	SIRT2	Q8IXJ6	-2.58	-5.88	0.71	7.53	0.12	0.35
Mild_v_Severe	Immune	OID00998	SPRY2	O43597	0.30	-0.23	0.84	2.29	0.25	0.35
Mild_v_Severe	Inflammation	OID00514	IL-15RA	Q13261	0.16	-0.12	0.44	1.39	0.25	0.35
Control_v_Case	Inflammation	OID00485	IL-17A	Q16552	0.19	-0.17	0.54	1.63	0.30	0.35
Control_v_Severe	Neurology	OID00375	TN-R	Q92752	-1.04	-2.36	0.29	4.21	0.12	0.35
Mild_v_Critical	Neurology	OID00319	ADAM 23	O75077	-0.28	-0.79	0.23	3.76	0.27	0.35
Mild_v_Critical	Immune	OID01024	IL5	P05113	0.17	-0.14	0.49	1.06	0.27	0.35
Mild_v_Critical	Inflammation	OID00545	FGF-19	O95750	-0.46	-1.28	0.37	8.53	0.27	0.35
Mild_v_Critical	Inflammation	OID00489	IL-20RA	Q9UHF4	0.12	-0.09	0.33	0.95	0.27	0.35
Control_v_Case	Neurology	OID00346	SKR3	P37023	0.12	-0.11	0.35	7.02	0.30	0.35
Longitudinal_in_Mild	Immune	OID00983	SH2B3	Q9UQQ2	1.63	-0.29	3.55	2.85	0.08	0.35
Control_v_Severe	Cardiovascular	OID00389	IL-1ra	P18510	2.14	-0.64	4.93	5.47	0.13	0.35
Control_v_Severe	Inflammation	OID00508	IL-10RA	Q13651	1.53	-0.45	3.52	1.15	0.13	0.35
Control_v_Severe	Inflammation	OID00553	TNFRSF9	Q07011	-1.48	-3.38	0.43	6.70	0.12	0.35
Control_v_Severe	Cardiovascular	OID00432	HO-1	P09601	-1.41	-3.25	0.43	11.78	0.13	0.35
Control_v_Severe	Immune	OID01015	LAMP3	Q9UQV4	-1.23	-2.82	0.37	4.55	0.13	0.35
Longitudinal_in_Critical	Immune	OID00939	ZBTB16	Q05516	-0.61	-1.39	0.18	1.75	0.11	0.35
Control_v_Critical	Neurology	OID00373	LAIR-2	Q6ISS4	-0.42	-1.16	0.33	5.28	0.27	0.35
Severe_v_Critical	Inflammation	OID00490	CXCL9	Q07325	0.53	-0.28	1.33	8.97	0.19	0.35
Severe_v_Critical	Neurology	OID00334	GM-CSF-R-alpha	P15509	0.35	-0.19	0.90	5.48	0.19	0.35
Mild_v_Severe	Immune	OID00960	CDSN	Q15517	-0.28	-0.76	0.21	2.64	0.26	0.35
Mild_v_Severe	Immune	OID01005	DCBLD2	Q96PD2	-0.17	-0.47	0.13	7.95	0.26	0.35

Severe_v_Critical	Cardiovascular	OID00417	GH	P01241	-0.74	-1.89	0.40	8.55	0.19	0.35
Control_v_Case	Cardiovascular	OID00436	CEACAM8	P31997	0.21	-0.19	0.61	4.45	0.30	0.36
Severe_v_Critical	Cardiovascular	OID00465	HSP 27	P04792	0.21	-0.12	0.54	10.21	0.19	0.36
Mild_v_Severe	Immune	OID00964	TRIM21	P19474	0.41	-0.33	1.14	2.75	0.27	0.36
Mild_v_Severe	Neurology	OID00308	CD38	P28907	0.18	-0.14	0.51	5.87	0.27	0.36
Mild_v_Severe	Inflammation	OID00515	IL-10RB	Q08334	0.14	-0.11	0.38	6.12	0.27	0.36
Mild_v_Severe	Neurology	OID00304	CRTAM	O95727	-0.26	-0.73	0.21	5.44	0.27	0.36
Mild_v_Critical	Cardiovascular	OID00454	GT	P51161	-0.32	-0.91	0.27	2.51	0.28	0.36
Mild_v_Critical	Inflammation	OID00515	IL-10RB	Q08334	0.15	-0.13	0.42	6.17	0.28	0.36
Control_v_Case	Inflammation	OID00489	IL-20RA	Q9UHF4	0.13	-0.12	0.37	0.94	0.31	0.36
Mild_v_Critical	Inflammation	OID00557	ST1A1	P50225	0.34	-0.29	0.97	1.66	0.28	0.36
Mild_v_Critical	Neurology	OID00352	FcRL2	Q96LA5	0.24	-0.21	0.70	5.23	0.29	0.36
Control_v_Critical	Cardiovascular	OID00458	PD-L2	Q9BQ51	0.19	-0.16	0.55	3.56	0.28	0.37
Control_v_Case	Inflammation	OID00512	FGF-21	Q9NSA1	0.42	-0.41	1.26	5.71	0.32	0.37
Control_v_Case	Cardiovascular	OID00410	FGF-21	Q9NSA1	0.48	-0.47	1.42	7.07	0.32	0.37
Control_v_Severe	Cardiovascular	OID00452	THPO	P40225	-1.34	-3.13	0.45	4.31	0.14	0.37
Control_v_Severe	Inflammation	OID00475	GDNF	P39905	0.68	-0.23	1.59	2.01	0.14	0.37
Control_v_Severe	Neurology	OID00358	DDR1	Q08345	-0.65	-1.53	0.22	7.24	0.14	0.37
Mild_v_Severe	Cardiovascular	OID00383	SLAMF7	Q9NQ25	0.30	-0.25	0.84	3.49	0.28	0.37
Mild_v_Severe	Immune	OID00936	PPP1R9B	Q96SB3	0.36	-0.31	1.04	1.56	0.28	0.37
Mild_v_Severe	Cardiovascular	OID00444	DCN	P07585	-0.13	-0.36	0.11	4.44	0.28	0.37
Severe_v_Critical	Immune	OID01023	LAG3	P18627	0.32	-0.19	0.83	3.37	0.21	0.37
Mild_v_Severe	Cardiovascular	OID00395	PAR-1	P25116	0.22	-0.19	0.62	8.55	0.28	0.38
Control_v_Case	Cardiovascular	OID00423	REN	P00797	0.21	-0.22	0.64	6.64	0.33	0.38
Control_v_Critical	Immune	OID01006	FCRL6	Q6DN72	0.42	-0.37	1.21	3.25	0.29	0.38
Mild_v_Severe	Neurology	OID00293	VWC2	Q2TAL6	0.32	-0.28	0.91	5.55	0.29	0.38
Mild_v_Severe	Cardiovascular	OID00418	FS	P19883	-0.21	-0.61	0.19	10.76	0.29	0.38
Control_v_Critical	Cardiovascular	OID00453	MARCO	Q9UEW3	-0.16	-0.45	0.14	6.87	0.29	0.38
Control_v_Critical	Cardiovascular	OID00385	ADAM-TS13	Q76LX8	0.11	-0.10	0.32	4.98	0.29	0.38
Longitudinal_in_Mild	Neurology	OID00334	GM-CSF-R-alpha	P15509	-1.48	-3.27	0.31	5.41	0.09	0.38
Severe_v_Critical	Cardiovascular	OID00450	GDF-2	Q9UK05	0.38	-0.23	1.00	7.59	0.21	0.38
Severe_v_Critical	Neurology	OID00292	UNC5C	O95185	0.19	-0.11	0.48	4.59	0.21	0.38
Control_v_Mild	Neurology	OID00296	EZR	P15311	0.22	-0.17	0.61	3.84	0.26	0.38
Severe_v_Critical	Immune	OID00990	MGMT	P16455	0.43	-0.26	1.12	4.23	0.21	0.38

Control_v_Mild	Inflammation	OID00474	MCP-3	P80098	0.34	-0.26	0.95	1.85	0.26	0.38
Severe_v_Critical	Cardiovascular	OID00380	ANGPT1	Q15389	-0.42	-1.09	0.25	8.41	0.21	0.38
Severe_v_Critical	Neurology	OID00339	GZMA	P12544	0.27	-0.16	0.69	6.65	0.22	0.38
Severe_v_Critical	Neurology	OID00365	TNFRSF21	O75509	0.14	-0.09	0.37	8.08	0.21	0.38
Control_v_Mild	Inflammation	OID00545	FGF-19	O95750	0.51	-0.39	1.41	8.71	0.26	0.38
Control_v_Case	Inflammation	OID00510	MMP-1	P03956	-0.35	-1.07	0.37	14.68	0.33	0.38
Control_v_Case	Neurology	OID00351	BMP-4	P12644	-0.25	-0.76	0.26	4.51	0.34	0.38
Longitudinal_in_Mild	Immune	OID01006	FCRL6	Q6DN72	-0.96	-2.15	0.22	3.88	0.10	0.39
Longitudinal_in_Mild	Cardiovascular	OID00394	TNFRSF11A	Q9Y6Q6	0.66	-0.15	1.47	5.61	0.10	0.39
Longitudinal_in_Mild	Cardiovascular	OID00416	SPON2	Q9BUD6	0.30	-0.07	0.67	8.39	0.10	0.39
Mild_v_Severe	Neurology	OID00356	CTSC	P53634	-0.27	-0.79	0.25	3.60	0.30	0.39
Longitudinal_in_Mild	Neurology	OID00310	MSR1	P21757	-0.85	-1.92	0.21	6.67	0.10	0.39
Longitudinal_in_Mild	Neurology	OID00321	RSPO1	Q2MKA7	-0.39	-0.88	0.10	2.42	0.10	0.39
Mild_v_Critical	Immune	OID00954	FGF2	P09038	0.14	-0.13	0.41	0.49	0.31	0.39
Longitudinal_in_Mild	Neurology	OID00343	GDNFR-alpha-3	O60609	0.95	-0.24	2.13	5.04	0.10	0.39
Longitudinal_in_Mild	Neurology	OID00311	Alpha-2-MRAP	P30533	0.62	-0.16	1.39	7.89	0.10	0.39
Control_v_Severe	Cardiovascular	OID00396	TRAIL-R2	O14763	-0.85	-2.01	0.31	6.34	0.15	0.39
Control_v_Mild	Cardiovascular	OID00405	LOX-1	P78380	-0.36	-1.01	0.29	6.94	0.27	0.39
Control_v_Mild	Immune	OID00956	DPP10	Q8N608	-0.27	-0.75	0.22	1.50	0.27	0.39
Mild_v_Severe	Cardiovascular	OID00452	THPO	P40225	-0.15	-0.45	0.14	3.66	0.30	0.39
Longitudinal_in_Mild	Inflammation	OID00535	CXCL10	P02778	-1.98	-4.48	0.52	10.66	0.10	0.39
Longitudinal_in_Mild	Neurology	OID00357	CDH6	P55285	0.83	-0.23	1.89	4.48	0.11	0.39
Severe_v_Critical	Inflammation	OID00478	IL7	P13232	0.29	-0.19	0.77	2.76	0.22	0.39
Control_v_Case	Cardiovascular	OID00442	IgG Fc receptor II-b	P31994	0.28	-0.31	0.88	3.15	0.35	0.40
Control_v_Case	Cardiovascular	OID00428	TM	P07204	-0.11	-0.33	0.12	9.60	0.35	0.40
Control_v_Mild	Inflammation	OID00476	CDCP1	Q9H5V8	0.40	-0.33	1.12	2.72	0.28	0.40
Control_v_Mild	Neurology	OID00313	EPHB6	O15197	-0.19	-0.54	0.16	3.93	0.28	0.40
Control_v_Case	Neurology	OID00334	GM-CSF-R-alpha	P15509	-0.24	-0.76	0.27	5.49	0.35	0.40

Longitudinal_in_Critical	Inflammation	OID00478	IL7	P13232	0.44	-0.16	1.05	3.00	0.13	0.40
Control_v_Case	Immune	OID01006	FCRL6	Q6DN72	0.23	-0.26	0.72	3.33	0.36	0.40
Control_v_Case	Immune	OID00984	FCRL3	Q96P31	-0.11	-0.35	0.13	1.04	0.36	0.40
Longitudinal_in_Critical	Immune	OID00993	IL10	P22301	0.36	-0.13	0.86	5.95	0.13	0.40
Longitudinal_in_Critical	Inflammation	OID00515	IL-10RB	Q08334	0.36	-0.13	0.85	6.23	0.13	0.40
Mild_v_Severe	Cardiovascular	OID00398	TIE2	Q02763	-0.13	-0.38	0.12	7.30	0.31	0.40
Mild_v_Severe	Cardiovascular	OID00442	IgG Fc receptor II-b	P31994	0.37	-0.36	1.09	3.10	0.31	0.40
Control_v_Mild	Immune	OID00969	HNMT	P50135	0.28	-0.24	0.80	9.00	0.28	0.40
Control_v_Mild	Inflammation	OID00553	TNFRSF9	Q07011	-0.25	-0.70	0.21	6.70	0.28	0.40
Control_v_Mild	Cardiovascular	OID00461	TNFRSF13B	O14836	0.23	-0.19	0.64	9.68	0.29	0.40
Mild_v_Severe	Cardiovascular	OID00388	SRC	P12931	0.47	-0.47	1.41	5.64	0.31	0.41
Longitudinal_in_Critical	Inflammation	OID00503	TGF-alpha	P01135	0.95	-0.37	2.27	4.17	0.13	0.41
Longitudinal_in_Critical	Neurology	OID00327	NEP	P08473	0.41	-0.16	0.97	2.76	0.13	0.41
Control_v_Critical	Neurology	OID00299	EFNA4	P52798	0.20	-0.20	0.60	3.20	0.31	0.41
Severe_v_Critical	Immune	OID00977	CD28	P10747	0.20	-0.13	0.52	1.50	0.23	0.41
Control_v_Mild	Immune	OID00974	LY75	O60449	-0.27	-0.78	0.24	2.99	0.29	0.41
Longitudinal_in_Mild	Neurology	OID00297	SMOC2	Q9H3U7	1.02	-0.30	2.34	7.68	0.11	0.41
Control_v_Case	Cardiovascular	OID00419	GLO1	Q04760	-0.22	-0.70	0.26	6.08	0.36	0.41
Severe_v_Critical	Neurology	OID00302	PRTG	Q2VWP7	0.11	-0.08	0.31	6.28	0.23	0.41
Severe_v_Critical	Neurology	OID00327	NEP	P08473	-0.39	-1.04	0.27	3.10	0.24	0.41
Mild_v_Severe	Inflammation	OID00483	IL-17C	Q9P0M4	0.35	-0.36	1.07	2.41	0.32	0.41
Severe_v_Critical	Immune	OID01000	ARNT	P27540	0.21	-0.15	0.56	1.17	0.24	0.41
Severe_v_Critical	Immune	OID00941	TPSAB1	Q15661	-0.18	-0.49	0.13	4.53	0.24	0.41
Control_v_Critical	Cardiovascular	OID00403	IL-17D	Q8TAD2	-0.19	-0.57	0.19	2.41	0.32	0.41
Control_v_Case	Immune	OID00973	NFATC3	Q12968	0.17	-0.21	0.55	1.06	0.37	0.41
Mild_v_Severe	Cardiovascular	OID00428	TM	P07204	-0.21	-0.64	0.22	9.47	0.32	0.41
Severe_v_Critical	Inflammation	OID00510	MMP-1	P03956	-0.48	-1.30	0.34	14.94	0.24	0.41
Severe_v_Critical	Cardiovascular	OID00399	TF	P13726	0.28	-0.20	0.75	6.33	0.24	0.41
Severe_v_Critical	Inflammation	OID00506	TNFSF14	O43557	0.25	-0.18	0.67	6.18	0.25	0.41
Severe_v_Critical	Neurology	OID00331	TMPRSS5	Q9H3S3	0.17	-0.12	0.45	2.20	0.24	0.41

Severe_v_Critical	Immune	OID00979	BIRC2	Q13490	0.16	-0.12	0.45	0.70	0.25	0.41
Mild_v_Critical	Immune	OID00970	CCL11	P51671	-0.15	-0.45	0.15	7.23	0.33	0.41
Severe_v_Critical	Neurology	OID00352	FcRL2	Q96LA5	0.24	-0.17	0.65	5.36	0.25	0.42
Severe_v_Critical	Cardiovascular	OID00453	MARCO	Q9UEW3	0.11	-0.08	0.31	6.90	0.25	0.42
Severe_v_Critical	Neurology	OID00318	CLEC1B	Q9P126	0.34	-0.26	0.94	11.12	0.25	0.42
Longitudinal_in_Critical	Cardiovascular	OID00382	CD40-L	P29965	1.40	-0.63	3.44	4.41	0.15	0.42
Longitudinal_in_Critical	Neurology	OID00366	CLM-1	Q8TDQ1	0.73	-0.32	1.78	7.44	0.15	0.42
Longitudinal_in_Critical	Immune	OID01024	IL5	P05113	-0.59	-1.43	0.25	0.91	0.14	0.42
Longitudinal_in_Critical	Immune	OID00958	DCTN1	Q14203	-0.54	-1.33	0.24	4.16	0.15	0.42
Longitudinal_in_Critical	Neurology	OID00299	EFNA4	P52798	0.52	-0.23	1.28	3.52	0.15	0.42
Longitudinal_in_Critical	Inflammation	OID00557	ST1A1	P50225	0.47	-0.21	1.14	2.09	0.15	0.42
Longitudinal_in_Critical	Cardiovascular	OID00420	CD84	Q9UIB8	-0.37	-0.90	0.15	4.76	0.14	0.42
Longitudinal_in_Critical	Inflammation	OID00505	CCL11	P51671	-0.26	-0.63	0.11	7.24	0.14	0.42
Longitudinal_in_Critical	Inflammation	OID00526	ARTN	Q5T4W7	0.21	-0.09	0.50	1.09	0.14	0.42
Longitudinal_in_Critical	Neurology	OID00344	PVR	P15151	-0.13	-0.31	0.05	9.06	0.14	0.42
Control_v_Severe	Immune	OID00952	PRDX1	Q06830	-1.78	-4.28	0.73	4.65	0.16	0.42
Control_v_Severe	Neurology	OID00311	Alpha-2-MRAP	P30533	-1.28	-3.08	0.52	9.31	0.16	0.42
Longitudinal_in_Critical	Immune	OID00988	CLEC4D	Q8WXI8	0.95	-0.44	2.34	5.01	0.15	0.42
Longitudinal_in_Critical	Neurology	OID00377	Nr-CAM	Q92823	-0.17	-0.41	0.08	9.32	0.15	0.42
Mild_v_Severe	Neurology	OID00350	GCP5	P78333	-0.39	-1.19	0.41	4.57	0.33	0.42
Mild_v_Severe	Immune	OID00999	STC1	P52823	0.22	-0.23	0.66	7.01	0.33	0.42
Severe_v_Critical	Inflammation	OID00533	Flt3L	P49771	0.34	-0.26	0.93	8.86	0.25	0.42
Control_v_Case	Immune	OID01008	CXCL12	P48061	0.08	-0.09	0.24	1.55	0.37	0.42
Longitudinal_in_Critical	Inflammation	OID00538	SIRT2	Q8IXJ6	0.64	-0.30	1.59	4.73	0.15	0.42
Longitudinal_in_Critical	Inflammation	OID00562	CSF-1	P09603	0.14	-0.07	0.35	10.78	0.15	0.42

Severe_v_Critical	Cardiovascular	OID00421	PAPPA	Q13219	0.32	-0.25	0.88	2.94	0.26	0.42
Severe_v_Critical	Immune	OID00944	CLEC4G	Q6UXB4	0.27	-0.21	0.74	4.28	0.26	0.42
Control_v_Mild	Immune	OID00992	CXADR	P78310	0.20	-0.18	0.57	2.23	0.30	0.42
Longitudinal_in_Critical	Cardiovascular	OID00466	CD4	P01730	0.21	-0.10	0.53	5.14	0.15	0.42
Mild_v_Critical	Immune	OID01020	TANK	Q92844	0.25	-0.27	0.78	1.30	0.33	0.42
Severe_v_Critical	Cardiovascular	OID00408	SCF	P21583	0.36	-0.29	1.01	8.05	0.26	0.42
Severe_v_Critical	Cardiovascular	OID00467	NEMO	Q9Y6K9	0.34	-0.27	0.95	5.45	0.26	0.42
Severe_v_Critical	Neurology	OID00307	CPA2	P48052	-0.28	-0.77	0.22	9.04	0.26	0.42
Severe_v_Critical	Inflammation	OID00485	IL-17A	Q16552	-0.22	-0.62	0.18	1.82	0.26	0.42
Severe_v_Critical	Immune	OID00976	EIF4G1	Q04637	0.36	-0.29	1.00	4.23	0.27	0.42
Control_v_Severe	Cardiovascular	OID00469	PARP-1	P09874	-1.82	-4.47	0.83	5.34	0.17	0.42
Control_v_Severe	Inflammation	OID00513	CCL19	Q99731	1.72	-0.78	4.23	9.64	0.17	0.42
Control_v_Severe	Neurology	OID00351	BMP-4	P12644	1.42	-0.64	3.49	4.39	0.17	0.42
Control_v_Severe	Neurology	OID00340	G-CSF	P09919	1.40	-0.61	3.41	3.01	0.17	0.42
Control_v_Severe	Inflammation	OID00541	EN-RAGE	P80511	1.35	-0.58	3.27	3.00	0.16	0.42
Control_v_Severe	Immune	OID00989	PRKCQ	Q04759	-1.02	-2.48	0.43	1.68	0.16	0.42
Control_v_Severe	Cardiovascular	OID00458	PD-L2	Q9BQ51	-1.00	-2.43	0.44	3.49	0.17	0.42
Control_v_Severe	Cardiovascular	OID00425	MERTK	Q12866	-0.90	-2.20	0.39	6.43	0.17	0.42
Control_v_Severe	Neurology	OID00336	SCARA5	Q6ZMJ2	-0.74	-1.82	0.33	8.60	0.17	0.42
Severe_v_Critical	Neurology	OID00372	NTRK3	Q16288	-0.09	-0.26	0.07	6.68	0.27	0.42
Control_v_Mild	Immune	OID00984	FCRL3	Q96P31	-0.18	-0.52	0.17	1.03	0.30	0.42
Mild_v_Severe	Neurology	OID00291	GDNF	P39905	-0.13	-0.41	0.15	1.95	0.34	0.43
Control_v_Mild	Neurology	OID00305	RGMA	Q96B86	-0.18	-0.54	0.17	10.96	0.30	0.43
Mild_v_Critical	Immune	OID00974	LY75	O60449	-0.14	-0.45	0.16	2.76	0.34	0.43
Longitudinal_in_Critical	Neurology	OID00302	PRTG	Q2VWP7	0.28	-0.14	0.70	6.30	0.16	0.43
Longitudinal_in_Critical	Cardiovascular	OID00394	TNFRSF11A	Q9Y6Q6	0.20	-0.10	0.50	6.59	0.16	0.43
Severe_v_Critical	Neurology	OID00373	LAIR-2	Q6ISS4	0.27	-0.22	0.76	5.60	0.27	0.43
Severe_v_Critical	Neurology	OID00317	ADAM 22	Q9P0K1	0.19	-0.16	0.54	3.80	0.27	0.43
Severe_v_Critical	Cardiovascular	OID00442	IgG Fc receptor II-b	P31994	-0.20	-0.57	0.17	3.38	0.27	0.43
Control_v_Case	Neurology	OID00321	RSPO1	Q2MKA7	0.14	-0.17	0.45	2.96	0.39	0.43
Longitudinal_in_Critical	Neurology	OID00310	MSR1	P21757	0.15	-0.08	0.37	6.91	0.16	0.43
Mild_v_Critical	Inflammation	OID00487	AXIN1	O15169	0.30	-0.33	0.92	2.70	0.35	0.43

Longitudinal_in_Critical	Neurology	OID00341	DRAXIN	Q8NBI3	0.41	-0.21	1.03	4.17	0.16	0.43
Control_v_Mild	Cardiovascular	OID00431	PRELP	P51888	0.09	-0.09	0.28	8.30	0.31	0.43
Mild_v_Critical	Neurology	OID00358	DDR1	Q08345	0.11	-0.13	0.35	7.25	0.35	0.43
Longitudinal_in_Critical	Cardiovascular	OID00455	BNP	P16860	0.66	-0.35	1.66	1.56	0.17	0.43
Longitudinal_in_Critical	Immune	OID00936	PPP1R9B	Q96SB3	0.42	-0.22	1.05	1.94	0.17	0.43
Severe_v_Critical	Immune	OID00939	ZBTB16	Q05516	0.35	-0.30	0.99	1.58	0.28	0.44
Severe_v_Critical	Immune	OID00984	FCRL3	Q96P31	0.19	-0.16	0.53	1.06	0.28	0.44
Mild_v_Critical	Immune	OID00941	TPSAB1	Q15661	-0.19	-0.60	0.22	4.49	0.35	0.44
Longitudinal_in_Critical	Cardiovascular	OID00451	FABP2	P12104	-0.84	-2.14	0.46	7.09	0.17	0.44
Longitudinal_in_Critical	Cardiovascular	OID00461	TNFRSF13B	O14836	0.50	-0.28	1.29	10.21	0.17	0.44
Longitudinal_in_Critical	Neurology	OID00293	VWC2	Q2TAL6	-0.21	-0.54	0.12	5.58	0.17	0.44
Longitudinal_in_Mild	Neurology	OID00313	EPHB6	O15197	0.62	-0.21	1.45	3.87	0.12	0.44
Longitudinal_in_Mild	Inflammation	OID05547	IFN-gamma	P01579	-2.16	-5.04	0.72	7.74	0.12	0.44
Longitudinal_in_Mild	Immune	OID00947	IL6	P05231	-1.39	-3.26	0.48	2.52	0.12	0.44
Longitudinal_in_Mild	Cardiovascular	OID00382	CD40-L	P29965	2.38	-0.81	5.58	4.36	0.12	0.44
Control_v_Severe	Neurology	OID00319	ADAM 23	O75077	-1.15	-2.86	0.56	4.10	0.18	0.44
Control_v_Case	Inflammation	OID00561	TNFB	P01374	-0.14	-0.46	0.18	4.58	0.40	0.44
Control_v_Mild	Neurology	OID00376	CD200R1	Q8TD46	0.16	-0.16	0.47	4.32	0.32	0.44
Control_v_Severe	Neurology	OID00333	GFR-alpha-1	P56159	-0.59	-1.48	0.29	7.03	0.18	0.44
Mild_v_Severe	Neurology	OID00312	sFRP-3	Q92765	0.17	-0.20	0.53	5.36	0.35	0.44
Longitudinal_in_Mild	Neurology	OID00356	CTSC	P53634	1.43	-0.51	3.36	3.92	0.13	0.44
Control_v_Mild	Neurology	OID00373	LAIR-2	Q6ISS4	-0.43	-1.29	0.43	5.00	0.32	0.44
Severe_v_Critical	Inflammation	OID00500	SCF	P21583	0.33	-0.30	0.96	7.98	0.29	0.45
Severe_v_Critical	Neurology	OID00348	CPM	P14384	0.17	-0.15	0.49	6.15	0.29	0.45
Control_v_Severe	Immune	OID00993	IL10	P22301	1.18	-0.62	2.99	4.05	0.19	0.45
Control_v_Severe	Immune	OID00963	TRAF2	Q12933	-1.18	-2.98	0.62	2.97	0.19	0.45
Control_v_Severe	Immune	OID00949	CLEC4C	Q8WTT0	-1.17	-2.96	0.62	3.78	0.19	0.45

Control_v_Severe	Cardiovascular	OID00437	PTX3	P26022	1.16	-0.61	2.94	4.21	0.19	0.45
Control_v_Severe	Cardiovascular	OID00402	IL-27	Q8NEV9, Q14213	1.12	-0.60	2.84	6.08	0.19	0.45
Control_v_Severe	Inflammation	OID00528	IL10	P22301	1.07	-0.58	2.72	3.86	0.20	0.45
Control_v_Severe	Cardiovascular	OID00381	ADM	P35318	-0.92	-2.31	0.47	8.34	0.19	0.45
Control_v_Severe	Neurology	OID00314	RGMB	Q6NW40	-0.86	-2.18	0.46	5.88	0.19	0.45
Control_v_Severe	Neurology	OID00305	RGMA	Q96B86	-0.83	-2.12	0.45	10.82	0.20	0.45
Control_v_Severe	Neurology	OID00377	Nr-CAM	Q92823	-0.49	-1.23	0.26	9.57	0.19	0.45
Control_v_Severe	Inflammation	OID05124	CD8A	P01732	1.92	-1.05	4.89	9.36	0.20	0.45
Mild_v_Critical	Neurology	OID00356	CTSC	P53634	0.25	-0.30	0.79	3.95	0.36	0.45
Control_v_Severe	Cardiovascular	OID00444	DCN	P07585	0.78	-0.43	1.99	4.39	0.20	0.45
Severe_v_Critical	Inflammation	OID00472	VEGFA	P15692	0.21	-0.19	0.60	12.04	0.29	0.45
Longitudinal_in_Mild	Neurology	OID00349	CLEC10A	Q8IUN9	1.53	-0.58	3.65	5.01	0.13	0.45
Control_v_Severe	Immune	OID00938	PSIP1	O75475	-1.92	-4.95	1.11	4.47	0.21	0.45
Control_v_Severe	Inflammation	OID00527	MMP-10	P09238	-1.60	-4.12	0.93	9.16	0.21	0.45
Control_v_Severe	Inflammation	OID00499	CD6	P30203	-1.35	-3.47	0.77	6.01	0.21	0.45
Control_v_Severe	Immune	OID00961	GALNT3	Q14435	-0.98	-2.54	0.57	2.70	0.21	0.45
Control_v_Severe	Immune	OID00948	DGKZ	Q13574	-0.94	-2.41	0.53	1.20	0.20	0.45
Control_v_Mild	Cardiovascular	OID00463	LEP	P41159	-0.52	-1.59	0.54	5.97	0.33	0.45
Mild_v_Severe	Neurology	OID00345	TNFRSF12A	Q9NP84	0.18	-0.22	0.59	4.83	0.36	0.45
Mild_v_Severe	Cardiovascular	OID00423	REN	P00797	-0.17	-0.56	0.21	6.19	0.36	0.45
Mild_v_Severe	Cardiovascular	OID00430	AMBP	P02760	-0.08	-0.25	0.09	7.58	0.36	0.45
Severe_v_Critical	Neurology	OID00353	MDGA1	Q8NFP4	0.33	-0.31	0.97	3.40	0.30	0.45
Severe_v_Critical	Cardiovascular	OID00435	SORT1	Q99523	0.13	-0.12	0.37	8.72	0.30	0.45
Control_v_Severe	Inflammation	OID00551	CCL25	O15444	1.48	-0.89	3.86	6.19	0.21	0.45
Control_v_Severe	Neurology	OID00290	CADM3	Q8N126	-1.00	-2.59	0.60	3.40	0.21	0.45
Control_v_Severe	Neurology	OID00347	FLRT2	O43155	-0.46	-1.19	0.27	2.30	0.21	0.45
Control_v_Mild	Inflammation	OID00499	CD6	P30203	-0.34	-1.05	0.36	6.20	0.33	0.45
Severe_v_Critical	Inflammation	OID00524	IL-24	Q13007	0.27	-0.26	0.80	1.81	0.30	0.46
Control_v_Mild	Neurology	OID00307	CPA2	P48052	-0.42	-1.28	0.44	10.08	0.33	0.46
Mild_v_Critical	Immune	OID01006	FCRL6	Q6DN72	-0.29	-0.94	0.36	3.54	0.37	0.46
Mild_v_Critical	Immune	OID00940	IRAK4	Q9NWZ3	0.26	-0.33	0.85	1.92	0.37	0.46
Mild_v_Critical	Inflammation	OID00533	Flt3L	P49771	-0.18	-0.59	0.23	9.00	0.37	0.46
Mild_v_Critical	Inflammation	OID00509	FGF-5	P12034	-0.07	-0.23	0.09	1.02	0.37	0.46

Longitudinal_in_Critical	Immune	OID00997	PIK3AP1	Q6ZUJ8	0.73	-0.43	1.88	3.28	0.18	0.46
Longitudinal_in_Critical	Neurology	OID00358	DDR1	Q08345	0.27	-0.16	0.70	7.15	0.18	0.46
Longitudinal_in_Critical	Inflammation	OID05548	TNF	P01375	0.40	-0.24	1.05	4.06	0.18	0.46
Control_v_Severe	Cardiovascular	OID00453	MARCO	Q9UEW3	0.50	-0.31	1.31	6.84	0.22	0.46
Mild_v_Critical	Cardiovascular	OID00428	TM	P07204	0.11	-0.14	0.37	9.65	0.38	0.46
Control_v_Severe	Inflammation	OID00511	LIF-R	P42702	0.68	-0.42	1.79	4.13	0.22	0.46
Control_v_Case	Immune	OID01000	ARNT	P27540	-0.08	-0.26	0.11	0.95	0.42	0.46
Control_v_Mild	Inflammation	OID00479	OPG	O00300	0.18	-0.20	0.56	10.07	0.34	0.46
Longitudinal_in_Critical	Cardiovascular	OID00456	MMP12	P39900	1.06	-0.65	2.77	5.93	0.19	0.46
Longitudinal_in_Critical	Immune	OID00995	KLRD1	Q13241	0.56	-0.35	1.47	7.17	0.19	0.46
Longitudinal_in_Mild	Immune	OID01023	LAG3	P18627	-1.37	-3.32	0.58	3.33	0.14	0.46
Longitudinal_in_Mild	Immune	OID00982	PLXNA4	Q9HCM2	1.19	-0.48	2.86	3.67	0.14	0.46
Longitudinal_in_Mild	Neurology	OID00336	SCARA5	Q6ZMJ2	0.72	-0.30	1.74	8.53	0.14	0.46
Longitudinal_in_Mild	Immune	OID00984	FCRL3	Q96P31	0.51	-0.21	1.24	0.91	0.14	0.46
Longitudinal_in_Mild	Neurology	OID00323	LXN	Q9BS40	-0.36	-0.88	0.15	1.58	0.14	0.46
Longitudinal_in_Mild	Inflammation	OID00517	IL-18R1	Q13478	-0.24	-0.57	0.10	8.61	0.14	0.46
Longitudinal_in_Critical	Inflammation	OID00482	IL6	P05231	1.38	-0.88	3.63	7.71	0.19	0.47
Longitudinal_in_Critical	Neurology	OID00322	HAGH	Q16775	0.87	-0.55	2.28	6.16	0.19	0.47
Control_v_Severe	Immune	OID00996	BACH1	O14867	-1.17	-3.09	0.75	2.65	0.22	0.47
Mild_v_Severe	Neurology	OID00295	CLM-6	Q08708	0.16	-0.21	0.54	5.99	0.38	0.47
Longitudinal_in_Critical	Inflammation	OID00551	CCL25	O15444	0.36	-0.24	0.96	6.45	0.19	0.47
Mild_v_Severe	Neurology	OID00346	SKR3	P37023	0.13	-0.17	0.44	6.85	0.38	0.47
Control_v_Severe	Cardiovascular	OID00434	IL16	Q14005	-1.31	-3.47	0.85	6.80	0.23	0.47
Control_v_Severe	Cardiovascular	OID00447	PRSS8	Q16651	-0.71	-1.87	0.46	8.99	0.23	0.47
Control_v_Severe	Neurology	OID00354	IL-5R-alpha	Q01344	2.20	-1.53	5.92	4.29	0.24	0.47

Control_v_Severe	Inflammation	OID00474	MCP-3	P80098	-1.53	-4.12	1.06	2.52	0.24	0.47
Control_v_Severe	Cardiovascular	OID00459	CTSL1	P07711	0.89	-0.62	2.39	7.64	0.24	0.47
Control_v_Severe	Neurology	OID00321	RSPO1	Q2MKA7	-0.85	-2.27	0.57	2.96	0.23	0.47
Control_v_Severe	Neurology	OID00363	N2DL-2	Q9BZM5	-0.83	-2.22	0.57	3.15	0.24	0.47
Control_v_Severe	Cardiovascular	OID00384	PGF	P49763	-0.74	-1.97	0.49	7.49	0.23	0.47
Control_v_Severe	Neurology	OID00313	EPHB6	O15197	-0.74	-1.96	0.49	3.94	0.23	0.47
Control_v_Severe	Immune	OID00974	LY75	O60449	-0.72	-1.92	0.49	2.94	0.24	0.47
Control_v_Severe	Neurology	OID00291	GDNF	P39905	0.66	-0.44	1.77	1.86	0.23	0.47
Control_v_Severe	Neurology	OID00328	GDF-8	O14793	-0.63	-1.67	0.42	3.36	0.23	0.47
Control_v_Case	Inflammation	OID00508	IL-10RA	Q13651	0.09	-0.13	0.31	1.08	0.43	0.47
Longitudinal_in_Critical	Neurology	OID00326	LAYN	Q6UX15	0.38	-0.25	1.00	5.88	0.20	0.47
Mild_v_Severe	Neurology	OID00375	TN-R	Q92752	-0.27	-0.88	0.35	4.26	0.38	0.47
Longitudinal_in_Critical	Immune	OID00962	FXYD5	Q96DB9	-0.71	-1.88	0.47	0.95	0.20	0.47
Longitudinal_in_Critical	Neurology	OID00303	ROBO2	Q9HCK4	-0.22	-0.57	0.14	4.49	0.20	0.47
Control_v_Critical	Inflammation	OID00527	MMP-10	P09238	0.34	-0.41	1.10	9.39	0.37	0.47
Longitudinal_in_Critical	Immune	OID00959	ITGA6	P23229	0.35	-0.24	0.95	0.88	0.20	0.47
Control_v_Severe	Inflammation	OID00523	IL-12B	P29460	-1.21	-3.29	0.87	6.56	0.25	0.48
Control_v_Severe	Neurology	OID00376	CD200R1	Q8TD46	-0.69	-1.87	0.49	4.22	0.24	0.48
Control_v_Severe	Inflammation	OID00556	CCL20	P78556	1.84	-1.33	5.00	7.68	0.25	0.48
Longitudinal_in_Critical	Inflammation	OID00520	CXCL5	P42830	1.03	-0.71	2.78	10.10	0.21	0.48
Longitudinal_in_Critical	Cardiovascular	OID00449	HB-EGF	Q99075	0.68	-0.47	1.83	6.32	0.21	0.48
Longitudinal_in_Critical	Inflammation	OID00527	MMP-10	P09238	0.51	-0.36	1.39	9.49	0.21	0.48
Mild_v_Severe	Immune	OID01011	DAPP1	Q9UN19	0.36	-0.48	1.20	2.65	0.39	0.48
Longitudinal_in_Mild	Neurology	OID00366	CLM-1	Q8TDQ1	-0.94	-2.30	0.42	6.16	0.15	0.48
Longitudinal_in_Critical	Immune	OID00972	EGLN1	Q9GZT9	0.50	-0.36	1.36	2.13	0.21	0.48
Longitudinal_in_Critical	Inflammation	OID00531	CD5	P06127	0.45	-0.32	1.21	5.13	0.21	0.48
Longitudinal_in_Critical	Cardiovascular	OID00390	IL6	P05231	1.29	-0.93	3.51	8.59	0.21	0.48

Control_v_Case	Immune	OID00968	ITM2A	O43736	0.27	-0.43	0.98	2.34	0.44	0.49
Mild_v_Severe	Inflammation	OID00504	MCP-4	Q99616	0.31	-0.43	1.05	14.11	0.40	0.49
Severe_v_Critical	Neurology	OID00376	CD200R1	Q8TD46	0.12	-0.13	0.37	4.09	0.33	0.49
Control_v_Critical	Cardiovascular	OID00428	TM	P07204	0.11	-0.15	0.38	9.69	0.39	0.49
Control_v_Critical	Neurology	OID00376	CD200R1	Q8TD46	-0.16	-0.52	0.20	4.20	0.39	0.49
Control_v_Case	Cardiovascular	OID00413	SOD2	P04179	-0.02	-0.09	0.04	10.17	0.45	0.49
Control_v_Mild	Cardiovascular	OID00417	GH	P01241	-0.82	-2.64	0.99	7.59	0.37	0.50
Control_v_Mild	Cardiovascular	OID00396	TRAIL-R2	O14763	-0.16	-0.50	0.19	6.01	0.37	0.50
Mild_v_Severe	Immune	OID01015	LAMP3	Q9UQV4	0.32	-0.46	1.10	4.91	0.41	0.50
Mild_v_Critical	Cardiovascular	OID00395	PAR-1	P25116	0.13	-0.18	0.43	8.60	0.41	0.50
Control_v_Mild	Immune	OID01005	DCBLD2	Q96PD2	0.15	-0.19	0.49	7.90	0.37	0.50
Control_v_Severe	Neurology	OID00337	CD200	P41217	-0.97	-2.69	0.76	6.19	0.26	0.50
Control_v_Critical	Inflammation	OID00508	IL-10RA	Q13651	-0.24	-0.79	0.32	1.14	0.40	0.50
Mild_v_Critical	Neurology	OID00350	GCP5	P78333	-0.24	-0.82	0.35	4.49	0.42	0.50
Mild_v_Critical	Neurology	OID00323	LXN	Q9BS40	0.09	-0.13	0.31	1.68	0.42	0.50
Control_v_Severe	Immune	OID00973	NFATC3	Q12968	0.95	-0.75	2.66	1.10	0.26	0.50
Control_v_Mild	Cardiovascular	OID00398	TIE2	Q02763	-0.12	-0.40	0.16	7.35	0.38	0.51
Longitudinal_in_Critical	Immune	OID00966	NTF4	P34130	-0.29	-0.80	0.23	1.66	0.23	0.51
Longitudinal_in_Critical	Neurology	OID00311	Alpha-2-MRAP	P30533	0.14	-0.11	0.38	8.80	0.23	0.51
Severe_v_Critical	Cardiovascular	OID00440	CCL3	P10147	0.42	-0.47	1.31	7.55	0.34	0.51
Longitudinal_in_Critical	Cardiovascular	OID00430	AMBP	P02760	-0.18	-0.52	0.15	7.50	0.23	0.51
Longitudinal_in_Mild	Inflammation	OID00483	IL-17C	Q9P0M4	1.32	-0.63	3.26	2.53	0.16	0.51
Mild_v_Severe	Cardiovascular	OID00453	MARCO	Q9UEW3	-0.08	-0.28	0.12	6.91	0.42	0.51
Severe_v_Critical	Cardiovascular	OID00427	THBS2	P35442	0.08	-0.09	0.25	5.73	0.34	0.51
Longitudinal_in_Critical	Immune	OID00947	IL6	P05231	1.28	-1.03	3.60	7.43	0.23	0.51
Control_v_Mild	Neurology	OID00326	LAYN	Q6UX15	-0.20	-0.66	0.26	5.31	0.38	0.51
Control_v_Case	Inflammation	OID00542	CD40	P25942	-0.13	-0.48	0.22	11.53	0.47	0.51
Mild_v_Critical	Cardiovascular	OID00462	TGM2	P21980	-0.19	-0.66	0.29	7.96	0.43	0.51
Mild_v_Critical	Inflammation	OID00553	TNFRSF9	Q07011	0.13	-0.19	0.45	6.64	0.43	0.51
Mild_v_Critical	Cardiovascular	OID00458	PD-L2	Q9BQ51	-0.12	-0.42	0.18	3.54	0.43	0.51
Control_v_Critical	Immune	OID00980	HSD11B1	P28845	-0.18	-0.61	0.25	3.02	0.41	0.51

Longitudinal_in_Mild	Immune	OID00949	CLEC4C	Q8WTT0	-1.19	-2.98	0.61	4.20	0.16	0.52
Longitudinal_in_Mild	Cardiovascular	OID00384	PGF	P49763	0.46	-0.23	1.15	7.44	0.16	0.52
Control_v_Critical	Inflammation	OID00545	FGF-19	O95750	-0.40	-1.37	0.57	8.62	0.41	0.52
Control_v_Mild	Cardiovascular	OID00415	FGF-23	Q9GZV9	0.22	-0.29	0.72	2.79	0.39	0.52
Longitudinal_in_Mild	Cardiovascular	OID00383	SLAMF7	Q9NQ25	-0.81	-2.04	0.42	3.64	0.17	0.52
Longitudinal_in_Mild	Immune	OID00999	STC1	P52823	-0.77	-1.95	0.41	7.04	0.16	0.52
Severe_v_Critical	Cardiovascular	OID00422	SERPINA12	Q8IW75	0.37	-0.42	1.16	2.42	0.35	0.52
Longitudinal_in_Critical	Neurology	OID00318	CLEC1B	Q9P126	0.71	-0.59	2.01	11.32	0.24	0.52
Control_v_Severe	Cardiovascular	OID00394	TNFRSF11A	Q9Y6Q6	-0.98	-2.77	0.81	5.88	0.27	0.52
Control_v_Case	Inflammation	OID00545	FGF-19	O95750	-0.24	-0.92	0.43	8.51	0.48	0.52
Mild_v_Critical	Immune	OID00968	ITM2A	O43736	0.41	-0.65	1.47	2.43	0.44	0.52
Severe_v_Critical	Immune	OID01001	FAM3B	P58499	0.27	-0.31	0.85	4.38	0.35	0.52
Severe_v_Critical	Inflammation	OID00526	ARTN	Q5T4W7	0.13	-0.15	0.42	1.09	0.35	0.52
Longitudinal_in_Critical	Cardiovascular	OID00396	TRAIL-R2	O14763	0.65	-0.55	1.85	7.96	0.24	0.52
Longitudinal_in_Critical	Immune	OID00981	NF2	P35240	-0.50	-1.41	0.42	-0.22	0.24	0.52
Control_v_Critical	Inflammation	OID00499	CD6	P30203	-0.28	-0.96	0.40	5.89	0.42	0.52
Control_v_Critical	Neurology	OID00363	N2DL-2	Q9BZM5	0.25	-0.36	0.86	3.61	0.42	0.52
Longitudinal_in_Mild	Cardiovascular	OID00461	TNFRSF13B	O14836	-0.93	-2.36	0.49	9.91	0.17	0.53
Mild_v_Critical	Cardiovascular	OID00382	CD40-L	P29965	0.44	-0.71	1.60	4.38	0.44	0.53
Control_v_Severe	Neurology	OID00339	GZMA	P12544	0.91	-0.77	2.58	6.01	0.28	0.53
Control_v_Severe	Immune	OID00971	MILR1	Q7Z6M3	-0.81	-2.30	0.69	2.94	0.28	0.53
Longitudinal_in_Mild	Inflammation	OID00494	OSM	P13725	-0.93	-2.36	0.50	4.10	0.17	0.53
Longitudinal_in_Mild	Neurology	OID00312	sFRP-3	Q92765	-0.50	-1.29	0.28	5.34	0.17	0.53
Control_v_Mild	Immune	OID00967	KRT19	P08727	0.26	-0.36	0.89	2.09	0.40	0.53
Longitudinal_in_Critical	Neurology	OID00337	CD200	P41217	-0.32	-0.91	0.28	6.00	0.25	0.53
Control_v_Severe	Neurology	OID00345	TNFRSF12A	Q9NP84	-0.79	-2.28	0.69	5.01	0.28	0.53
Mild_v_Critical	Cardiovascular	OID00443	ITGB1BP2	Q9UKP3	0.27	-0.44	0.98	2.98	0.45	0.53

Control_v_Case	Cardiovascular	OID00447	PRSS8	Q16651	-0.08	-0.33	0.16	8.94	0.49	0.53
Control_v_Mild	Inflammation	OID00471	IL8	P10145	-0.25	-0.85	0.35	5.07	0.40	0.53
Longitudinal_in_Critical	Immune	OID01013	SIT1	Q9Y3P8	1.09	-0.96	3.14	2.79	0.25	0.53
Control_v_Mild	Inflammation	OID00556	CCL20	P78556	0.42	-0.59	1.43	7.45	0.41	0.53
Control_v_Mild	Neurology	OID00346	SKR3	P37023	-0.14	-0.49	0.20	6.84	0.41	0.53
Control_v_Mild	Immune	OID01010	IFNLR1	Q8IU57	0.14	-0.19	0.47	2.50	0.41	0.53
Longitudinal_in_Critical	Neurology	OID00373	LAIR-2	Q6ISS4	0.43	-0.38	1.25	5.40	0.25	0.53
Mild_v_Severe	Immune	OID01025	CD83	Q01151	-0.18	-0.63	0.28	2.87	0.44	0.54
Longitudinal_in_Critical	Inflammation	OID00541	EN-RAGE	P80511	-0.29	-0.83	0.26	5.33	0.25	0.54
Control_v_Mild	Immune	OID01018	DDX58	O95786	-0.29	-0.99	0.41	3.62	0.42	0.54
Control_v_Mild	Neurology	OID00304	CRTAM	O95727	0.25	-0.37	0.87	5.32	0.42	0.54
Longitudinal_in_Mild	Neurology	OID00319	ADAM 23	O75077	1.63	-0.97	4.23	3.83	0.18	0.54
Longitudinal_in_Mild	Inflammation	OID00523	IL-12B	P29460	1.60	-0.90	4.10	6.81	0.18	0.54
Longitudinal_in_Mild	Cardiovascular	OID00429	VSIG2	Q96IQ7	1.15	-0.66	2.96	3.90	0.18	0.54
Longitudinal_in_Mild	Immune	OID00988	CLEC4D	Q8WXI8	-0.59	-1.53	0.35	3.56	0.18	0.54
Control_v_Case	Inflammation	OID00509	FGF-5	P12034	-0.05	-0.19	0.09	1.06	0.50	0.54
Control_v_Mild	Inflammation	OID00494	OSM	P13725	-0.30	-1.03	0.44	4.12	0.42	0.54
Control_v_Mild	Cardiovascular	OID00456	MMP12	P39900	-0.28	-0.96	0.41	7.01	0.42	0.54
Control_v_Mild	Cardiovascular	OID00434	IL16	Q14005	-0.27	-0.95	0.41	6.64	0.42	0.54
Control_v_Mild	Neurology	OID00290	CADM3	Q8N126	-0.22	-0.75	0.32	3.39	0.42	0.54
Control_v_Mild	Cardiovascular	OID00394	TNFRSF11A	Q9Y6Q6	-0.18	-0.62	0.26	5.68	0.42	0.54
Control_v_Mild	Inflammation	OID00524	IL-24	Q13007	0.10	-0.15	0.34	0.90	0.43	0.54
Longitudinal_in_Critical	Neurology	OID00348	CPM	P14384	0.24	-0.22	0.70	5.94	0.26	0.54
Control_v_Severe	Neurology	OID00344	PVR	P15151	-0.75	-2.17	0.67	8.28	0.29	0.54
Control_v_Severe	Cardiovascular	OID00454	GT	P51161	1.23	-1.11	3.56	2.27	0.29	0.54
Longitudinal_in_Mild	Cardiovascular	OID00431	PRELP	P51888	0.23	-0.14	0.61	8.36	0.19	0.54
Severe_v_Critical	Cardiovascular	OID00457	ACE2	Q9BYF1	0.27	-0.34	0.89	4.56	0.37	0.54
Severe_v_Critical	Neurology	OID05024	MAPT	P10636	-0.08	-0.25	0.10	0.39	0.37	0.54

Control_v_Severe	Cardiovascular	OID00464	CA5A	P35218	-1.37	-4.00	1.26	2.76	0.30	0.54
Control_v_Severe	Immune	OID00965	LILRB4	Q8NHJ6	0.80	-0.74	2.35	3.52	0.30	0.54
Control_v_Severe	Inflammation	OID00517	IL-18R1	Q13478	0.96	-0.89	2.81	8.60	0.30	0.55
Mild_v_Critical	Immune	OID00995	KLRD1	Q13241	0.21	-0.36	0.78	6.90	0.46	0.55
Longitudinal_in_Mild	Cardiovascular	OID00448	AGRP	O00253	-0.88	-2.30	0.55	5.54	0.19	0.55
Longitudinal_in_Mild	Immune	OID00942	HCLS1	P14317	0.74	-0.47	1.96	4.23	0.19	0.55
Longitudinal_in_Mild	Neurology	OID00317	ADAM 22	Q9P0K1	0.73	-0.47	1.93	4.33	0.19	0.55
Longitudinal_in_Mild	Immune	OID00974	LY75	O60449	0.83	-0.54	2.21	2.95	0.20	0.55
Longitudinal_in_Mild	Inflammation	OID00527	MMP-10	P09238	0.67	-0.42	1.76	9.07	0.20	0.55
Longitudinal_in_Critical	Neurology	OID00360	CTSS	P25774	0.13	-0.12	0.37	6.16	0.26	0.55
Mild_v_Severe	Cardiovascular	OID00422	SERPINA12	Q8IW75	-0.38	-1.42	0.66	3.34	0.46	0.55
Severe_v_Critical	Cardiovascular	OID00382	CD40-L	P29965	-0.53	-1.75	0.69	4.58	0.38	0.55
Severe_v_Critical	Cardiovascular	OID00418	FS	P19883	0.22	-0.28	0.72	10.98	0.38	0.55
Severe_v_Critical	Cardiovascular	OID00411	PIgR	P01833	0.05	-0.07	0.18	5.70	0.38	0.55
Mild_v_Severe	Inflammation	OID00508	IL-10RA	Q13651	0.07	-0.12	0.25	1.00	0.46	0.55
Longitudinal_in_Critical	Inflammation	OID00513	CCL19	Q99731	0.77	-0.74	2.28	11.23	0.27	0.56
Mild_v_Severe	Cardiovascular	OID00425	MERTK	Q12866	0.12	-0.21	0.46	6.57	0.46	0.56
Control_v_Critical	Neurology	OID00362	NAAA	Q02083	0.22	-0.35	0.79	3.40	0.45	0.56
Control_v_Severe	Immune	OID00944	CLEC4G	Q6UXB4	1.07	-1.03	3.16	3.50	0.31	0.56
Control_v_Case	Cardiovascular	OID00453	MARCO	Q9UEW3	0.05	-0.11	0.22	6.88	0.52	0.56
Severe_v_Critical	Cardiovascular	OID00426	KIM1	Q96D42	0.38	-0.51	1.26	9.34	0.39	0.56
Severe_v_Critical	Immune	OID00983	SH2B3	Q9UQQ2	-0.28	-0.93	0.38	3.01	0.39	0.56
Control_v_Case	Cardiovascular	OID00463	LEP	P41159	0.20	-0.42	0.82	6.00	0.52	0.56
Longitudinal_in_Critical	Cardiovascular	OID00410	FGF-21	Q9NSA1	0.74	-0.73	2.21	7.65	0.27	0.56
Longitudinal_in_Critical	Immune	OID01009	AREG	P15514	0.46	-0.46	1.37	5.97	0.28	0.56
Longitudinal_in_Critical	Cardiovascular	OID00383	SLAMF7	Q9NQ25	0.42	-0.43	1.28	4.28	0.28	0.56
Longitudinal_in_Critical	Neurology	OID00361	N-CDase	Q9NR71	-0.42	-1.27	0.43	2.89	0.28	0.56

Longitudinal_in_Critical	Inflammation	OID00514	IL-15RA	Q13261	0.42	-0.43	1.26	1.83	0.28	0.56
Longitudinal_in_Critical	Immune	OID01019	IL12RB1	P42701	0.40	-0.41	1.20	3.02	0.28	0.56
Longitudinal_in_Critical	Inflammation	OID00490	CXCL9	Q07325	0.33	-0.34	1.00	9.02	0.28	0.56
Longitudinal_in_Critical	Neurology	OID00367	SPOCK1	Q08629	-0.26	-0.79	0.27	2.53	0.28	0.56
Control_v_Severe	Neurology	OID00323	LXN	Q9BS40	-0.87	-2.61	0.86	2.56	0.31	0.56
Mild_v_Critical	Immune	OID00959	ITGA6	P23229	-0.15	-0.59	0.28	0.95	0.48	0.57
Control_v_Severe	Immune	OID00988	CLEC4D	Q8WXI8	1.07	-1.08	3.22	3.52	0.32	0.57
Control_v_Severe	Immune	OID01013	SIT1	Q9Y3P8	-0.78	-2.34	0.78	2.14	0.32	0.57
Control_v_Severe	Immune	OID01019	IL12RB1	P42701	0.57	-0.57	1.72	2.32	0.32	0.57
Longitudinal_in_Critical	Inflammation	OID00501	IL18	Q14116	0.80	-0.86	2.46	10.67	0.29	0.57
Longitudinal_in_Critical	Immune	OID01012	PADI2	Q9Y2J8	0.70	-0.75	2.14	1.94	0.29	0.57
Longitudinal_in_Critical	Neurology	OID00340	G-CSF	P09919	-0.67	-2.08	0.74	4.09	0.30	0.57
Longitudinal_in_Critical	Inflammation	OID00536	4E-BP1	Q13541	0.55	-0.60	1.70	8.93	0.30	0.57
Longitudinal_in_Critical	Cardiovascular	OID00433	XCL1	P47992	0.54	-0.60	1.68	5.32	0.30	0.57
Longitudinal_in_Critical	Cardiovascular	OID00400	IL1RL2	Q9HB29	0.43	-0.48	1.33	4.32	0.30	0.57
Longitudinal_in_Critical	Neurology	OID00352	FcRL2	Q96LA5	0.37	-0.39	1.12	5.42	0.29	0.57
Longitudinal_in_Critical	Neurology	OID00356	CTSC	P53634	-0.29	-0.89	0.31	4.08	0.30	0.57
Longitudinal_in_Critical	Cardiovascular	OID00393	IDUA	P35475	-0.24	-0.76	0.27	5.36	0.30	0.57
Longitudinal_in_Critical	Neurology	OID00346	SKR3	P37023	0.24	-0.27	0.74	7.30	0.30	0.57
Longitudinal_in_Critical	Neurology	OID00351	BMP-4	P12644	-0.27	-0.83	0.30	4.33	0.30	0.57
Severe_v_Critical	Immune	OID01021	ITGA11	Q9UKX5	-0.12	-0.40	0.16	2.17	0.40	0.57
Longitudinal_in_Mild	Immune	OID00987	HEXIM1	O94992	0.83	-0.57	2.23	4.21	0.21	0.57
Control_v_Severe	Immune	OID01009	AREG	P15514	0.98	-1.01	2.97	3.78	0.32	0.57
Control_v_Severe	Cardiovascular	OID00416	SPON2	Q9BUD6	-0.31	-0.93	0.32	8.29	0.32	0.57

Longitudinal_in_Mild	Cardiovascular	OID00427	THBS2	P35442	0.20	-0.14	0.54	5.54	0.21	0.57
Control_v_Critical	Immune	OID00938	PSIP1	O75475	0.35	-0.61	1.32	4.65	0.46	0.57
Control_v_Critical	Neurology	OID00329	THY 1	P04216	-0.11	-0.42	0.20	10.00	0.47	0.58
Control_v_Mild	Cardiovascular	OID00433	XCL1	P47992	0.17	-0.29	0.64	4.86	0.46	0.58
Control_v_Mild	Cardiovascular	OID00411	PIgR	P01833	-0.05	-0.17	0.08	5.73	0.46	0.58
Mild_v_Critical	Immune	OID01016	CLEC7A	Q9BXN2	0.17	-0.32	0.65	3.26	0.50	0.58
Mild_v_Critical	Immune	OID00998	SPRY2	O43597	0.16	-0.31	0.63	2.31	0.49	0.58
Longitudinal_in_Mild	Inflammation	OID00531	CD5	P06127	0.46	-0.32	1.24	5.39	0.21	0.58
Longitudinal_in_Critical	Cardiovascular	OID00409	IL18	Q14116	0.67	-0.78	2.12	10.42	0.31	0.58
Mild_v_Severe	Immune	OID00959	ITGA6	P23229	-0.20	-0.79	0.38	1.00	0.48	0.58
Control_v_Mild	Inflammation	OID00485	IL-17A	Q16552	-0.17	-0.64	0.30	1.52	0.46	0.58
Control_v_Mild	Neurology	OID00292	UNC5C	O95185	-0.12	-0.45	0.21	4.48	0.46	0.58
Longitudinal_in_Mild	Neurology	OID00308	CD38	P28907	-0.30	-0.81	0.22	5.92	0.21	0.58
Control_v_Case	Neurology	OID00293	VWC2	Q2TAL6	0.09	-0.21	0.40	5.52	0.54	0.58
Longitudinal_in_Mild	Immune	OID01027	BTN3A2	P78410	-0.84	-2.29	0.61	2.98	0.22	0.58
Mild_v_Critical	Immune	OID00982	PLXNA4	Q9HCM2	0.26	-0.50	1.02	3.55	0.50	0.58
Control_v_Critical	Immune	OID00973	NFATC3	Q12968	0.17	-0.30	0.65	1.24	0.47	0.58
Control_v_Critical	Cardiovascular	OID00440	CCL3	P10147	0.39	-0.69	1.46	7.12	0.47	0.58
Control_v_Severe	Inflammation	OID00476	CDCP1	Q9H5V8	0.80	-0.86	2.45	2.96	0.33	0.58
Control_v_Severe	Immune	OID00984	FCRL3	Q96P31	-0.51	-1.56	0.55	1.08	0.33	0.58
Control_v_Case	Cardiovascular	OID00407	GIF	P27352	-0.19	-0.83	0.44	6.79	0.55	0.58
Mild_v_Critical	Inflammation	OID00549	MCP-2	P80075	0.22	-0.43	0.86	10.03	0.51	0.58
Mild_v_Critical	Cardiovascular	OID00453	MARCO	Q9UEW3	-0.06	-0.25	0.13	6.91	0.50	0.58
Mild_v_Severe	Cardiovascular	OID00439	CCL17	Q92583	0.22	-0.43	0.88	8.39	0.49	0.58
Longitudinal_in_Mild	Immune	OID00969	HNMT	P50135	0.68	-0.51	1.86	9.15	0.22	0.58
Control_v_Severe	Inflammation	OID00489	IL-20RA	Q9UHF4	0.58	-0.64	1.81	0.93	0.34	0.58
Severe_v_Critical	Inflammation	OID00514	IL-15RA	Q13261	0.14	-0.20	0.48	1.74	0.41	0.58
Mild_v_Severe	Inflammation	OID05124	CD8A	P01732	-0.26	-1.05	0.53	9.83	0.50	0.59
Mild_v_Severe	Neurology	OID00294	Siglec-9	Q9Y336	0.14	-0.28	0.56	4.93	0.50	0.59
Mild_v_Severe	Neurology	OID00300	SCARB2	Q14108	0.14	-0.28	0.56	4.86	0.50	0.59

Mild_v_Severe	Immune	OID01012	PADI2	Q9Y2J8	0.12	-0.24	0.49	0.87	0.49	0.59
Mild_v_Severe	Immune	OID01007	NCR1	O76036	0.11	-0.22	0.45	3.36	0.50	0.59
Mild_v_Severe	Cardiovascular	OID00420	CD84	Q9UIB8	0.11	-0.22	0.43	4.59	0.50	0.59
Mild_v_Severe	Inflammation	OID00491	CST5	P28325	-0.10	-0.41	0.21	6.42	0.50	0.59
Mild_v_Severe	Inflammation	OID00553	TNFRSF9	Q07011	-0.09	-0.35	0.18	6.60	0.51	0.59
Longitudinal_in_Mild	Neurology	OID00307	CPA2	P48052	0.83	-0.63	2.28	10.21	0.22	0.59
Control_v_Mild	Cardiovascular	OID00409	IL18	Q14116	-0.21	-0.79	0.37	8.98	0.47	0.59
Longitudinal_in_Mild	Inflammation	OID00477	CD244	Q9BZW8	-0.52	-1.43	0.39	6.02	0.22	0.59
Longitudinal_in_Critical	Inflammation	OID00539	CCL28	Q9NRJ3	0.13	-0.15	0.40	1.87	0.32	0.59
Longitudinal_in_Critical	Immune	OID00954	FGF2	P09038	-0.36	-1.15	0.43	0.69	0.32	0.59
Longitudinal_in_Critical	Immune	OID01004	DFFA	O00273	-0.19	-0.61	0.23	5.54	0.32	0.59
Control_v_Severe	Immune	OID00962	FXYD5	Q96DB9	-1.00	-3.11	1.12	1.84	0.35	0.59
Control_v_Severe	Neurology	OID00309	SMPD1	P17405	0.64	-0.72	1.99	4.55	0.35	0.59
Control_v_Severe	Cardiovascular	OID00435	SORT1	Q99523	-0.57	-1.77	0.63	8.88	0.34	0.59
Control_v_Case	Neurology	OID00370	EDA2R	Q9HAV5	0.09	-0.22	0.40	4.57	0.56	0.59
Longitudinal_in_Critical	Immune	OID00960	CDSN	Q15517	0.26	-0.32	0.84	2.40	0.33	0.59
Severe_v_Critical	Immune	OID01024	IL5	P05113	0.24	-0.37	0.86	1.16	0.42	0.59
Severe_v_Critical	Immune	OID00972	EGLN1	Q9GZT9	0.16	-0.24	0.56	2.00	0.42	0.59
Mild_v_Critical	Cardiovascular	OID00422	SERPINA12	Q8IW75	-0.25	-1.03	0.53	2.95	0.52	0.60
Severe_v_Critical	Neurology	OID00304	CRTAM	O95727	0.22	-0.34	0.77	5.31	0.43	0.60
Mild_v_Critical	Inflammation	OID00491	CST5	P28325	-0.15	-0.62	0.32	6.46	0.52	0.60
Longitudinal_in_Mild	Cardiovascular	OID00414	CTRC	Q99895	-0.73	-2.03	0.56	10.76	0.23	0.60
Severe_v_Critical	Cardiovascular	OID00430	AMBP	P02760	0.07	-0.11	0.26	7.56	0.43	0.60
Control_v_Severe	Inflammation	OID00526	ARTN	Q5T4W7	0.61	-0.71	1.93	0.87	0.35	0.60
Control_v_Severe	Immune	OID01010	IFNLR1	Q8IU57	-0.63	-2.00	0.74	2.57	0.36	0.60
Control_v_Severe	Neurology	OID00301	NCAN	O14594	0.59	-0.69	1.88	8.46	0.36	0.60
Severe_v_Critical	Cardiovascular	OID00454	GT	P51161	0.27	-0.42	0.95	2.33	0.43	0.60
Control_v_Critical	Cardiovascular	OID00419	GLO1	Q04760	-0.22	-0.88	0.43	6.39	0.49	0.61
Longitudinal_in_Critical	Cardiovascular	OID00462	TGM2	P21980	0.33	-0.42	1.07	8.15	0.34	0.61

Control_v_Severe	Inflammation	OID00506	TNFSF14	O43557	-1.20	-3.84	1.44	6.04	0.36	0.61
Control_v_Severe	Immune	OID00972	EGLN1	Q9GZT9	-0.85	-2.73	1.04	1.78	0.37	0.61
Control_v_Severe	Neurology	OID00320	MATN3	O15232	0.61	-0.75	1.97	9.20	0.37	0.61
Control_v_Mild	Neurology	OID00372	NTRK3	Q16288	-0.11	-0.43	0.21	7.40	0.49	0.61
Control_v_Mild	Cardiovascular	OID00416	SPON2	Q9BUD6	0.07	-0.13	0.26	8.27	0.49	0.61
Severe_v_Critical	Inflammation	OID00538	SIRT2	Q8IXJ6	0.24	-0.39	0.87	4.58	0.44	0.61
Severe_v_Critical	Neurology	OID00362	NAAA	Q02083	0.13	-0.21	0.48	3.12	0.44	0.61
Severe_v_Critical	Cardiovascular	OID00446	LPL	P06858	0.12	-0.19	0.43	9.43	0.44	0.61
Mild_v_Critical	Cardiovascular	OID00455	BNP	P16860	0.27	-0.59	1.13	1.84	0.53	0.61
Longitudinal_in_Mild	Inflammation	OID00538	SIRT2	Q8IXJ6	0.69	-0.55	1.93	3.57	0.23	0.61
Severe_v_Critical	Neurology	OID00374	MANF	P55145	0.30	-0.50	1.10	7.46	0.45	0.61
Severe_v_Critical	Immune	OID00963	TRAF2	Q12933	0.14	-0.24	0.53	1.91	0.45	0.61
Mild_v_Severe	Inflammation	OID00554	NT-3	P20783	-0.10	-0.41	0.22	2.01	0.53	0.61
Mild_v_Critical	Neurology	OID00359	JAM-B	P57087	-0.12	-0.50	0.26	7.72	0.54	0.61
Longitudinal_in_Critical	Neurology	OID00335	Beta-NGF	P01138	0.13	-0.17	0.44	1.60	0.34	0.61
Mild_v_Critical	Neurology	OID00322	HAGH	Q16775	0.24	-0.55	1.03	5.79	0.54	0.61
Control_v_Case	Immune	OID01024	IL5	P05113	0.11	-0.28	0.50	1.03	0.58	0.62
Control_v_Case	Neurology	OID00326	LAYN	Q6UX15	0.08	-0.21	0.37	5.49	0.58	0.62
Control_v_Case	Immune	OID00977	CD28	P10747	-0.07	-0.32	0.18	1.67	0.58	0.62
Longitudinal_in_Critical	Immune	OID00942	HCLS1	P14317	0.36	-0.47	1.18	5.31	0.34	0.62
Longitudinal_in_Mild	Immune	OID00997	PIK3AP1	Q6ZUJ8	-0.80	-2.26	0.67	2.62	0.24	0.62
Control_v_Severe	Inflammation	OID00485	IL-17A	Q16552	-0.70	-2.27	0.88	1.73	0.38	0.62
Control_v_Critical	Cardiovascular	OID00417	GH	P01241	-0.52	-2.11	1.06	8.24	0.51	0.62
Control_v_Severe	Cardiovascular	OID00446	LPL	P06858	-0.84	-2.75	1.07	9.52	0.38	0.62
Control_v_Severe	Neurology	OID00368	IL12	P29460, P29459	-1.10	-3.65	1.44	8.36	0.38	0.62
Control_v_Severe	Neurology	OID00297	SMOC2	Q9H3U7	-0.56	-1.84	0.73	7.82	0.38	0.62
Control_v_Severe	Neurology	OID00312	sFRP-3	Q92765	-0.53	-1.74	0.69	5.48	0.38	0.62
Longitudinal_in_Mild	Inflammation	OID00472	VEGFA	P15692	0.72	-0.60	2.03	10.88	0.24	0.63
Mild_v_Critical	Neurology	OID00304	CRTAM	O95727	-0.16	-0.72	0.39	5.42	0.56	0.63
Mild_v_Critical	Cardiovascular	OID00430	AMBP	P02760	-0.06	-0.26	0.14	7.58	0.56	0.63

Mild_v_Critical	Cardiovascular	OID00380	ANGPT1	Q15389	0.29	-0.69	1.26	8.26	0.56	0.63
Longitudinal_in_Critical	Inflammation	OID00507	FGF-23	Q9GZV9	0.21	-0.29	0.70	2.58	0.35	0.63
Control_v_Critical	Cardiovascular	OID00413	SOD2	P04179	0.04	-0.08	0.15	10.23	0.52	0.63
Longitudinal_in_Mild	Inflammation	OID00530	CCL23	P55773	0.64	-0.54	1.82	10.51	0.25	0.63
Control_v_Mild	Cardiovascular	OID00448	AGRP	O00253	0.16	-0.32	0.64	5.31	0.51	0.63
Control_v_Severe	Cardiovascular	OID00406	Gal-9	O00182	0.66	-0.88	2.20	8.26	0.39	0.63
Control_v_Severe	Immune	OID00966	NTF4	P34130	0.52	-0.70	1.73	1.84	0.39	0.63
Control_v_Critical	Neurology	OID00346	SKR3	P37023	-0.13	-0.53	0.27	7.14	0.52	0.63
Control_v_Mild	Immune	OID00995	KLRD1	Q13241	0.17	-0.34	0.67	6.60	0.52	0.63
Severe_v_Critical	Neurology	OID00354	IL-5R-alpha	Q01344	-0.25	-0.93	0.44	5.14	0.47	0.64
Mild_v_Severe	Neurology	OID00288	NRP2	O60462	0.03	-0.08	0.15	8.20	0.55	0.64
Longitudinal_in_Mild	Cardiovascular	OID00451	FABP2	P12104	1.48	-1.32	4.28	8.00	0.26	0.64
Longitudinal_in_Mild	Neurology	OID00352	FcRL2	Q96LA5	-0.81	-2.37	0.75	5.14	0.26	0.64
Longitudinal_in_Mild	Neurology	OID00375	TN-R	Q92752	0.35	-0.32	1.03	4.53	0.26	0.64
Longitudinal_in_Mild	Inflammation	OID00541	EN-RAGE	P80511	-0.35	-1.00	0.31	2.31	0.26	0.64
Severe_v_Critical	Inflammation	OID00555	TWEAK	O43508	-0.15	-0.56	0.26	8.12	0.47	0.64
Longitudinal_in_Mild	Inflammation	OID00526	ARTN	Q5T4W7	0.47	-0.43	1.38	0.92	0.26	0.64
Control_v_Mild	Cardiovascular	OID00399	TF	P13726	-0.09	-0.39	0.20	5.86	0.52	0.64
Longitudinal_in_Mild	Cardiovascular	OID00464	CA5A	P35218	-1.51	-4.67	1.65	3.01	0.30	0.64
Longitudinal_in_Mild	Inflammation	OID00545	FGF-19	O95750	-1.12	-3.43	1.18	9.21	0.29	0.64
Longitudinal_in_Mild	Immune	OID01004	DFFA	O00273	-0.91	-2.83	1.02	4.23	0.30	0.64
Longitudinal_in_Mild	Immune	OID00944	CLEC4G	Q6UXB4	-0.80	-2.39	0.79	3.65	0.27	0.64
Longitudinal_in_Mild	Inflammation	OID00484	MCP-1	P13500	0.75	-0.75	2.26	11.66	0.28	0.64
Longitudinal_in_Mild	Neurology	OID00341	DRAXIN	Q8NBI3	0.74	-0.79	2.26	3.19	0.29	0.64
Longitudinal_in_Mild	Inflammation	OID00487	AXIN1	O15169	0.63	-0.64	1.90	2.84	0.29	0.64

Longitudinal_in_Mild	Cardiovascular	OID00434	IL16	Q14005	-0.62	-1.86	0.61	6.55	0.28	0.64
Longitudinal_in_Mild	Immune	OID01001	FAM3B	P58499	-0.59	-1.80	0.62	4.65	0.28	0.64
Longitudinal_in_Mild	Cardiovascular	OID00458	PD-L2	Q9BQ51	0.56	-0.58	1.70	3.71	0.29	0.64
Longitudinal_in_Mild	Inflammation	OID00542	CD40	P25942	0.49	-0.45	1.43	10.95	0.27	0.64
Longitudinal_in_Mild	Immune	OID00995	KLRD1	Q13241	-0.46	-1.44	0.51	6.53	0.30	0.64
Longitudinal_in_Mild	Cardiovascular	OID00381	ADM	P35318	0.45	-0.46	1.36	8.48	0.29	0.64
Longitudinal_in_Mild	Inflammation	OID00490	CXCL9	Q07325	-0.42	-1.26	0.42	7.38	0.28	0.64
Longitudinal_in_Mild	Immune	OID00991	TREM1	Q9NP99	-0.40	-1.21	0.41	2.22	0.28	0.64
Longitudinal_in_Mild	Immune	OID00966	NTF4	P34130	0.40	-0.44	1.23	1.86	0.30	0.64
Longitudinal_in_Mild	Cardiovascular	OID00444	DCN	P07585	0.39	-0.40	1.18	4.48	0.29	0.64
Longitudinal_in_Mild	Neurology	OID00331	TMPRSS5	Q9H3S3	0.35	-0.34	1.04	2.67	0.27	0.64
Longitudinal_in_Mild	Neurology	OID00369	Dkk-4	Q9UBT3	-0.28	-0.87	0.31	3.21	0.30	0.64
Longitudinal_in_Mild	Immune	OID00954	FGF2	P09038	0.24	-0.26	0.74	0.49	0.30	0.64
Longitudinal_in_Mild	Inflammation	OID00514	IL-15RA	Q13261	0.24	-0.24	0.72	1.35	0.29	0.64
Longitudinal_in_Mild	Cardiovascular	OID00441	MMP7	P09237	0.23	-0.23	0.70	9.71	0.28	0.64
Longitudinal_in_Mild	Neurology	OID00360	CTSS	P25774	-0.14	-0.42	0.15	5.68	0.30	0.64
Longitudinal_in_Critical	Immune	OID00974	LY75	O60449	-0.21	-0.73	0.30	2.59	0.36	0.64
Control_v_Critical	Immune	OID01026	ITGB6	P18564	-0.13	-0.53	0.28	3.12	0.53	0.64
Control_v_Critical	Neurology	OID00304	CRTAM	O95727	0.19	-0.42	0.79	5.24	0.53	0.64
Mild_v_Severe	Inflammation	OID00539	CCL28	Q9NRJ3	0.08	-0.19	0.35	2.36	0.56	0.64
Severe_v_Critical	Neurology	OID00297	SMOC2	Q9H3U7	0.16	-0.29	0.61	7.52	0.48	0.65
Control_v_Case	Cardiovascular	OID00465	HSP 27	P04792	-0.09	-0.43	0.25	10.06	0.61	0.65
Longitudinal_in_Mild	Immune	OID00964	TRIM21	P19474	-1.55	-4.88	1.78	2.92	0.31	0.65

Severe_v_Critical	Cardiovascular	OID00402	IL-27	Q8NEV9, Q14213	-0.18	-0.70	0.34	6.92	0.48	0.65
Control_v_Case	Cardiovascular	OID00379	BMP-6	P22004	0.10	-0.29	0.49	4.79	0.62	0.65
Control_v_Severe	Cardiovascular	OID00409	IL18	Q14116	-0.71	-2.42	1.01	9.37	0.41	0.65
Control_v_Severe	Neurology	OID00288	NRP2	O60462	-0.23	-0.80	0.33	8.22	0.41	0.65
Control_v_Severe	Cardiovascular	OID00391	TNFRSF10A	O00220	-0.44	-1.53	0.64	3.12	0.41	0.65
Severe_v_Critical	Neurology	OID00338	NTRK2	Q16620	-0.07	-0.27	0.13	5.69	0.49	0.65
Severe_v_Critical	Immune	OID01010	IFNLR1	Q8IU57	0.13	-0.25	0.51	3.10	0.49	0.65
Longitudinal_in_Critical	Cardiovascular	OID00407	GIF	P27352	-0.36	-1.23	0.52	6.37	0.37	0.65
Longitudinal_in_Critical	Neurology	OID00319	ADAM 23	O75077	-0.29	-1.01	0.43	3.60	0.37	0.65
Longitudinal_in_Critical	Cardiovascular	OID00416	SPON2	Q9BUD6	0.07	-0.11	0.25	8.75	0.37	0.65
Longitudinal_in_Mild	Neurology	OID00320	MATN3	O15232	0.79	-0.95	2.53	8.83	0.32	0.65
Longitudinal_in_Mild	Cardiovascular	OID00466	CD4	P01730	-0.68	-2.14	0.78	4.87	0.31	0.65
Longitudinal_in_Mild	Inflammation	OID00499	CD6	P30203	0.68	-0.79	2.15	6.33	0.32	0.65
Longitudinal_in_Mild	Neurology	OID00306	PLXNB3	Q9ULL4	0.67	-0.79	2.14	4.14	0.31	0.65
Longitudinal_in_Mild	Neurology	OID00296	EZR	P15311	-0.37	-1.20	0.45	3.82	0.32	0.65
Severe_v_Critical	Neurology	OID00312	sFRP-3	Q92765	0.12	-0.23	0.46	5.69	0.49	0.65
Mild_v_Severe	Cardiovascular	OID00393	IDUA	P35475	-0.12	-0.56	0.31	5.51	0.57	0.65
Control_v_Critical	Inflammation	OID00505	CCL11	P51671	-0.19	-0.81	0.44	7.65	0.55	0.65
Control_v_Critical	Cardiovascular	OID00445	Dkk-1	O94907	-0.18	-0.76	0.41	9.49	0.55	0.65
Longitudinal_in_Critical	Neurology	OID00363	N2DL-2	Q9BZM5	0.58	-0.87	2.03	4.34	0.38	0.66
Control_v_Critical	Neurology	OID00375	TN-R	Q92752	-0.14	-0.59	0.32	4.08	0.55	0.66
Longitudinal_in_Mild	Inflammation	OID00539	CCL28	Q9NRJ3	0.60	-0.72	1.92	2.44	0.33	0.66
Longitudinal_in_Mild	Inflammation	OID00557	ST1A1	P50225	0.58	-0.71	1.86	1.79	0.33	0.66
Longitudinal_in_Mild	Neurology	OID00329	THY 1	P04216	0.43	-0.55	1.42	9.91	0.33	0.66
Longitudinal_in_Mild	Neurology	OID00348	CPM	P14384	-0.32	-1.05	0.41	6.74	0.33	0.66

Longitudinal_in_Mild	Immune	OID01014	MASP1	P48740	-0.14	-0.46	0.18	1.95	0.33	0.66
Control_v_Severe	Immune	OID01002	SH2D1A	O60880	-0.97	-3.37	1.44	2.93	0.42	0.66
Severe_v_Critical	Inflammation	OID00553	TNFRSF9	Q07011	0.17	-0.35	0.70	6.61	0.50	0.66
Severe_v_Critical	Cardiovascular	OID00447	PRSS8	Q16651	0.08	-0.16	0.31	9.06	0.50	0.66
Severe_v_Critical	Cardiovascular	OID00460	hOSCAR	Q8IYS5	0.04	-0.08	0.16	11.03	0.50	0.66
Control_v_Mild	Neurology	OID00351	BMP-4	P12644	0.16	-0.36	0.67	4.64	0.54	0.66
Control_v_Mild	Immune	OID01025	CD83	Q01151	-0.13	-0.56	0.30	2.94	0.54	0.66
Longitudinal_in_Critical	Neurology	OID00329	THY 1	P04216	0.16	-0.24	0.55	9.84	0.38	0.66
Severe_v_Critical	Cardiovascular	OID00405	LOX-1	P78380	0.30	-0.61	1.20	8.08	0.51	0.66
Severe_v_Critical	Inflammation	OID00504	MCP-4	Q99616	0.18	-0.37	0.73	14.44	0.51	0.66
Severe_v_Critical	Cardiovascular	OID00434	IL16	Q14005	0.17	-0.35	0.68	7.15	0.51	0.66
Mild_v_Critical	Immune	OID01026	ITGB6	P18564	0.08	-0.23	0.39	3.07	0.59	0.66
Control_v_Severe	Cardiovascular	OID00440	CCL3	P10147	-1.27	-4.47	1.92	6.81	0.42	0.66
Control_v_Severe	Neurology	OID00366	CLM-1	Q8TDQ1	0.79	-1.24	2.83	6.35	0.43	0.67
Control_v_Severe	Inflammation	OID00471	IL8	P10145	-0.79	-2.80	1.22	5.77	0.43	0.67
Control_v_Severe	Immune	OID01025	CD83	Q01151	-0.45	-1.58	0.69	2.93	0.43	0.67
Severe_v_Critical	Inflammation	OID00508	IL-10RA	Q13651	0.08	-0.18	0.34	1.17	0.52	0.67
Severe_v_Critical	Cardiovascular	OID00445	Dkk-1	O94907	0.19	-0.42	0.80	9.25	0.52	0.67
Control_v_Severe	Cardiovascular	OID00419	GLO1	Q04760	0.80	-1.28	2.89	6.56	0.44	0.68
Longitudinal_in_Critical	Neurology	OID00324	gal-8	O00214	-0.24	-0.87	0.39	6.73	0.40	0.68
Longitudinal_in_Critical	Neurology	OID00313	EPHB6	O15197	-0.16	-0.57	0.26	3.82	0.40	0.68
Longitudinal_in_Critical	Inflammation	OID00477	CD244	Q9BZW8	0.16	-0.25	0.56	5.57	0.40	0.68
Longitudinal_in_Critical	Cardiovascular	OID00411	PIgR	P01833	-0.15	-0.53	0.24	5.70	0.40	0.68
Longitudinal_in_Critical	Cardiovascular	OID00428	TM	P07204	-0.12	-0.44	0.20	9.81	0.40	0.68
Longitudinal_in_Critical	Inflammation	OID00475	GDNF	P39905	0.07	-0.11	0.25	2.26	0.39	0.68
Control_v_Severe	Cardiovascular	OID00407	GIF	P27352	0.90	-1.44	3.24	6.77	0.44	0.68
Severe_v_Critical	Cardiovascular	OID00420	CD84	Q9UIB8	0.11	-0.24	0.45	4.65	0.53	0.68
Control_v_Critical	Cardiovascular	OID00394	TNFRSF11A	Q9Y6Q6	0.18	-0.45	0.80	6.14	0.57	0.68
Control_v_Critical	Inflammation	OID00498	CCL4	P13236	-0.23	-1.03	0.58	6.45	0.58	0.68

Longitudinal_in_Critical	Inflammation	OID00484	MCP-1	P13500	0.62	-1.05	2.29	13.15	0.41	0.68
Longitudinal_in_Critical	Immune	OID00955	PRDX5	P30044	0.15	-0.26	0.56	5.33	0.41	0.68
Longitudinal_in_Mild	Neurology	OID00368	IL12	P29460, P29459	1.23	-1.65	4.11	8.61	0.35	0.68
Longitudinal_in_Mild	Cardiovascular	OID00391	TNFRSF10A	O00220	-0.60	-1.99	0.79	2.87	0.35	0.68
Control_v_Mild	Neurology	OID00363	N2DL-2	Q9BZM5	-0.11	-0.51	0.28	2.98	0.56	0.68
Mild_v_Severe	Cardiovascular	OID00458	PD-L2	Q9BQ51	-0.10	-0.48	0.28	3.46	0.60	0.68
Longitudinal_in_Mild	Cardiovascular	OID00417	GH	P01241	2.74	-3.65	9.13	7.45	0.35	0.68
Longitudinal_in_Mild	Cardiovascular	OID00409	IL18	Q14116	-0.92	-3.09	1.24	8.86	0.36	0.68
Longitudinal_in_Mild	Cardiovascular	OID00405	LOX-1	P78380	-0.87	-2.92	1.18	6.88	0.36	0.68
Longitudinal_in_Mild	Immune	OID01005	DCBLD2	Q96PD2	-0.34	-1.18	0.49	8.02	0.36	0.68
Longitudinal_in_Mild	Immune	OID00945	IRF9	Q00978	-0.33	-1.12	0.47	1.54	0.36	0.68
Longitudinal_in_Mild	Cardiovascular	OID00433	XCL1	P47992	-0.32	-1.07	0.44	4.97	0.36	0.68
Longitudinal_in_Mild	Immune	OID00943	CNTNAP2	Q9UHC6	0.22	-0.31	0.75	1.36	0.36	0.68
Mild_v_Severe	Immune	OID01023	LAG3	P18627	-0.12	-0.60	0.35	3.02	0.60	0.69
Longitudinal_in_Critical	Inflammation	OID00552	CX3CL1	P78423	-0.20	-0.73	0.34	4.78	0.41	0.69
Mild_v_Critical	Neurology	OID00313	EPHB6	O15197	-0.07	-0.33	0.20	3.83	0.61	0.69
Control_v_Severe	Inflammation	OID00518	PD-L1	Q9NZQ7	0.42	-0.70	1.54	5.76	0.45	0.69
Control_v_Severe	Neurology	OID00372	NTRK3	Q16288	-0.40	-1.46	0.66	7.35	0.45	0.69
Longitudinal_in_Mild	Neurology	OID00290	CADM3	Q8N126	0.55	-0.79	1.88	3.42	0.37	0.69
Control_v_Severe	Cardiovascular	OID00414	CTRC	Q99895	1.09	-1.83	4.01	10.06	0.45	0.69
Longitudinal_in_Critical	Neurology	OID00331	TMPRSS5	Q9H3S3	-0.20	-0.74	0.34	2.12	0.42	0.69
Longitudinal_in_Critical	Immune	OID00984	FCRL3	Q96P31	0.09	-0.16	0.35	1.08	0.42	0.69
Longitudinal_in_Mild	Inflammation	OID00501	IL18	Q14116	-0.82	-2.78	1.15	9.04	0.37	0.69

Longitudinal_in_Mild	Immune	OID00976	EIF4G1	Q04637	0.67	-1.04	2.38	3.27	0.39	0.69
Longitudinal_in_Mild	Inflammation	OID00554	NT-3	P20783	0.66	-1.00	2.32	1.87	0.38	0.69
Longitudinal_in_Mild	Neurology	OID00314	RGMB	Q6NW40	0.56	-0.84	1.96	5.82	0.38	0.69
Longitudinal_in_Mild	Inflammation	OID00555	TWEAK	O43508	0.48	-0.71	1.68	8.74	0.38	0.69
Longitudinal_in_Mild	Immune	OID00937	GLB1	P16278	0.48	-0.73	1.69	0.86	0.38	0.69
Longitudinal_in_Mild	Neurology	OID00338	NTRK2	Q16620	0.48	-0.75	1.71	6.31	0.39	0.69
Longitudinal_in_Mild	Immune	OID00980	HSD11B1	P28845	0.43	-0.67	1.53	3.38	0.39	0.69
Longitudinal_in_Mild	Inflammation	OID00491	CST5	P28325	-0.41	-1.42	0.61	6.56	0.38	0.69
Longitudinal_in_Mild	Inflammation	OID00511	LIF-R	P42702	-0.20	-0.71	0.30	4.37	0.38	0.69
Longitudinal_in_Mild	Immune	OID00953	PRDX3	P30048	-0.14	-0.48	0.21	-0.46	0.39	0.69
Control_v_Mild	Immune	OID00970	CCL11	P51671	-0.15	-0.68	0.38	7.32	0.57	0.69
Longitudinal_in_Critical	Immune	OID00944	CLEC4G	Q6UXB4	-0.55	-2.14	1.03	4.48	0.44	0.69
Longitudinal_in_Critical	Immune	OID01016	CLEC7A	Q9BXN2	0.55	-1.02	2.11	3.50	0.44	0.69
Longitudinal_in_Critical	Cardiovascular	OID00388	SRC	P12931	-0.38	-1.47	0.71	5.94	0.44	0.69
Longitudinal_in_Critical	Immune	OID01006	FCRL6	Q6DN72	-0.37	-1.42	0.68	3.21	0.44	0.69
Longitudinal_in_Critical	Inflammation	OID00530	CCL23	P55773	0.35	-0.64	1.34	11.84	0.44	0.69
Longitudinal_in_Critical	Neurology	OID00292	UNC5C	O95185	0.18	-0.33	0.69	4.63	0.43	0.69
Longitudinal_in_Critical	Neurology	OID00291	GDNF	P39905	0.18	-0.32	0.68	2.15	0.43	0.69
Longitudinal_in_Critical	Cardiovascular	OID00406	Gal-9	O00182	-0.13	-0.48	0.23	9.30	0.44	0.69
Longitudinal_in_Critical	Neurology	OID00378	KYNU	Q16719	0.12	-0.23	0.47	9.83	0.44	0.69
Longitudinal_in_Critical	Inflammation	OID00524	IL-24	Q13007	0.38	-0.72	1.48	2.11	0.44	0.69

Longitudinal_in_Critical	Cardiovascular	OID00444	DCN	P07585	-0.25	-0.97	0.47	4.65	0.44	0.69
Mild_v_Critical	Cardiovascular	OID00439	CCL17	Q92583	0.23	-0.70	1.16	8.35	0.62	0.69
Control_v_Critical	Inflammation	OID00475	GDNF	P39905	-0.08	-0.39	0.23	2.16	0.59	0.69
Mild_v_Critical	Cardiovascular	OID00388	SRC	P12931	0.22	-0.68	1.11	5.73	0.63	0.69
Mild_v_Critical	Immune	OID00960	CDSN	Q15517	0.14	-0.43	0.71	2.73	0.63	0.69
Mild_v_Critical	Cardiovascular	OID00429	VSIG2	Q96IQ7	0.10	-0.32	0.52	3.84	0.63	0.69
Mild_v_Critical	Neurology	OID00347	FLRT2	O43155	0.06	-0.17	0.28	2.29	0.63	0.69
Control_v_Critical	Neurology	OID00291	GDNF	P39905	0.12	-0.32	0.55	2.02	0.59	0.70
Control_v_Critical	Inflammation	OID00523	IL-12B	P29460	0.18	-0.50	0.86	6.64	0.60	0.70
Longitudinal_in_Critical	Immune	OID01002	SH2D1A	O60880	0.32	-0.63	1.27	2.18	0.46	0.70
Longitudinal_in_Critical	Neurology	OID00364	PLXNB1	O43157	0.28	-0.55	1.10	2.73	0.46	0.70
Longitudinal_in_Critical	Neurology	OID00307	CPA2	P48052	-0.21	-0.84	0.42	8.94	0.46	0.70
Longitudinal_in_Critical	Cardiovascular	OID00431	PRELP	P51888	-0.19	-0.76	0.38	8.27	0.45	0.70
Longitudinal_in_Critical	Immune	OID00943	CNTNAP2	Q9UHC6	-0.10	-0.38	0.19	1.15	0.46	0.70
Severe_v_Critical	Inflammation	OID00531	CD5	P06127	0.09	-0.21	0.40	5.23	0.55	0.70
Severe_v_Critical	Inflammation	OID01213	DNER	Q8NFT8	-0.05	-0.20	0.11	8.12	0.55	0.70
Control_v_Mild	Neurology	OID00287	NMNAT1	Q9HAN9	-0.26	-1.19	0.68	3.29	0.58	0.70
Control_v_Case	Cardiovascular	OID00429	VSIG2	Q96IQ7	-0.08	-0.45	0.29	3.82	0.67	0.70
Mild_v_Severe	Cardiovascular	OID00415	FGF-23	Q9GZV9	-0.08	-0.38	0.23	2.71	0.62	0.70
Longitudinal_in_Mild	Inflammation	OID00474	MCP-3	P80098	-0.84	-3.04	1.36	2.05	0.41	0.70
Longitudinal_in_Mild	Cardiovascular	OID00406	Gal-9	O00182	-0.46	-1.68	0.75	8.37	0.41	0.70
Longitudinal_in_Mild	Inflammation	OID00488	TRAIL	P50591	0.39	-0.63	1.41	8.06	0.40	0.70
Longitudinal_in_Mild	Cardiovascular	OID00400	IL1RL2	Q9HB29	-0.23	-0.85	0.38	4.71	0.41	0.70
Longitudinal_in_Mild	Neurology	OID00326	LAYN	Q6UX15	-0.18	-0.64	0.29	5.23	0.40	0.70
Longitudinal_in_Critical	Immune	OID01001	FAM3B	P58499	0.19	-0.39	0.78	4.48	0.46	0.71
Control_v_Severe	Cardiovascular	OID00448	AGRP	O00253	-0.55	-2.06	0.96	5.33	0.47	0.71

Control_v_Severe	Immune	OID00943	CNTNAP2	Q9UHC6	0.48	-0.85	1.81	1.81	0.47	0.71
Control_v_Severe	Neurology	OID00338	NTRK2	Q16620	-0.35	-1.34	0.63	6.42	0.47	0.71
Control_v_Severe	Immune	OID00941	TPSAB1	Q15661	0.66	-1.20	2.52	4.78	0.48	0.71
Control_v_Severe	Inflammation	OID00514	IL-15RA	Q13261	-0.31	-1.19	0.57	1.38	0.48	0.71
Severe_v_Critical	Immune	OID00951	CLEC4A	Q9UMR7	0.07	-0.17	0.32	2.97	0.56	0.71
Longitudinal_in_Mild	Immune	OID01024	IL5	P05113	-1.30	-4.85	2.24	1.34	0.41	0.71
Longitudinal_in_Mild	Inflammation	OID00480	LAP TGF-beta-1	P01137	0.56	-0.96	2.08	6.01	0.42	0.71
Longitudinal_in_Mild	Neurology	OID00325	BCAN	Q96GW7	0.55	-0.97	2.07	4.30	0.42	0.71
Longitudinal_in_Mild	Inflammation	OID00550	CASP-8	Q14790	0.48	-0.83	1.80	3.07	0.42	0.71
Longitudinal_in_Mild	Cardiovascular	OID00397	PRSS27	Q9BQR3	0.42	-0.72	1.56	9.11	0.42	0.71
Longitudinal_in_Mild	Inflammation	OID00481	uPA	P00749	0.39	-0.68	1.47	9.82	0.43	0.71
Longitudinal_in_Mild	Cardiovascular	OID00379	BMP-6	P22004	0.36	-0.60	1.33	4.74	0.41	0.71
Longitudinal_in_Mild	Immune	OID01015	LAMP3	Q9UQV4	0.33	-0.59	1.24	5.14	0.42	0.71
Longitudinal_in_Mild	Cardiovascular	OID00468	VEGFD	O43915	0.14	-0.24	0.51	7.72	0.43	0.71
Mild_v_Critical	Immune	OID00983	SH2B3	Q9UQQ2	0.19	-0.64	1.01	2.85	0.65	0.71
Control_v_Critical	Neurology	OID00378	KYNU	Q16719	0.15	-0.45	0.76	9.31	0.61	0.71
Control_v_Mild	Immune	OID00978	PTH1R	Q03431	0.11	-0.29	0.50	4.04	0.59	0.71
Longitudinal_in_Critical	Cardiovascular	OID00448	AGRP	O00253	-0.09	-0.37	0.19	6.10	0.47	0.71
Longitudinal_in_Critical	Inflammation	OID00535	CXCL10	P02778	-0.15	-0.60	0.31	13.24	0.47	0.71
Longitudinal_in_Mild	Neurology	OID00353	MDGA1	Q8NFP4	1.35	-2.58	5.29	4.29	0.44	0.71
Longitudinal_in_Mild	Immune	OID00959	ITGA6	P23229	1.20	-2.38	4.77	1.40	0.46	0.71
Longitudinal_in_Mild	Immune	OID00961	GALNT3	Q14435	-0.53	-2.06	1.00	3.30	0.44	0.71
Longitudinal_in_Mild	Immune	OID01018	DDX58	O95786	-0.46	-1.84	0.92	3.32	0.45	0.71
Longitudinal_in_Mild	Inflammation	OID00558	STAMBP	O95630	0.35	-0.67	1.36	4.64	0.45	0.71

Longitudinal_in_Mild	Immune	OID00963	TRAF2	Q12933	0.35	-0.68	1.37	1.38	0.45	0.71
Longitudinal_in_Mild	Inflammation	OID00503	TGF-alpha	P01135	-0.33	-1.29	0.63	2.90	0.45	0.71
Longitudinal_in_Mild	Immune	OID01019	IL12RB1	P42701	-0.32	-1.29	0.64	2.45	0.45	0.71
Longitudinal_in_Mild	Cardiovascular	OID00438	PSGL-1	Q14242	0.28	-0.52	1.08	4.51	0.44	0.71
Longitudinal_in_Mild	Inflammation	OID00536	4E-BP1	Q13541	-0.25	-0.95	0.45	8.08	0.43	0.71
Longitudinal_in_Mild	Immune	OID01016	CLEC7A	Q9BXN2	-0.23	-0.90	0.44	3.18	0.45	0.71
Longitudinal_in_Mild	Inflammation	OID00553	TNFRSF9	Q07011	0.17	-0.33	0.67	6.69	0.45	0.71
Longitudinal_in_Mild	Immune	OID01022	KPNA1	P52294	-0.17	-0.65	0.32	-0.25	0.44	0.71
Severe_v_Critical	Neurology	OID00322	HAGH	Q16775	-0.24	-1.10	0.61	6.23	0.56	0.71
Control_v_Case	Immune	OID00938	PSIP1	O75475	0.13	-0.51	0.77	3.94	0.69	0.71
Control_v_Case	Immune	OID00972	EGLN1	Q9GZT9	0.06	-0.25	0.38	1.69	0.69	0.71
Control_v_Case	Cardiovascular	OID00454	GT	P51161	-0.10	-0.57	0.38	2.41	0.69	0.71
Longitudinal_in_Mild	Neurology	OID00344	PVR	P15151	-0.31	-1.22	0.61	8.31	0.46	0.72
Longitudinal_in_Critical	Immune	OID00977	CD28	P10747	0.24	-0.52	1.00	1.51	0.48	0.72
Control_v_Mild	Cardiovascular	OID00460	hOSCAR	Q8IYS5	-0.08	-0.38	0.22	10.72	0.60	0.72
Mild_v_Critical	Cardiovascular	OID00442	IgG Fc receptor II-b	P31994	0.13	-0.47	0.73	3.14	0.66	0.72
Mild_v_Critical	Immune	OID01022	KPNA1	P52294	0.01	-0.04	0.06	-0.20	0.66	0.72
Severe_v_Critical	Immune	OID01008	CXCL12	P48061	0.04	-0.11	0.19	1.70	0.57	0.72
Control_v_Critical	Inflammation	OID00502	SLAMF1	Q13291	-0.14	-0.68	0.41	2.66	0.62	0.72
Longitudinal_in_Critical	Neurology	OID00300	SCARB2	Q14108	-0.16	-0.65	0.34	5.77	0.48	0.72
Severe_v_Critical	Inflammation	OID00502	SLAMF1	Q13291	-0.11	-0.49	0.28	3.02	0.57	0.72
Severe_v_Critical	Cardiovascular	OID00379	BMP-6	P22004	-0.09	-0.41	0.23	5.04	0.57	0.72
Longitudinal_in_Critical	Immune	OID01005	DCBLD2	Q96PD2	-0.13	-0.55	0.29	7.75	0.48	0.72
Control_v_Case	Cardiovascular	OID00400	IL1RL2	Q9HB29	-0.05	-0.31	0.21	4.38	0.70	0.72
Mild_v_Severe	Immune	OID00992	CXADR	P78310	0.11	-0.35	0.56	2.31	0.64	0.72
Longitudinal_in_Mild	Neurology	OID00361	N-CDase	Q9NR71	-0.93	-3.79	1.93	3.57	0.47	0.72

Longitudinal_in_Mild	Cardiovascular	OID00385	ADAM-TS13	Q76LX8	0.08	-0.16	0.32	5.13	0.46	0.72
Control_v_Mild	Inflammation	OID00530	CCL23	P55773	0.16	-0.46	0.78	10.31	0.61	0.72
Control_v_Mild	Inflammation	OID00533	Flt3L	P49771	0.09	-0.26	0.43	9.21	0.61	0.72
Severe_v_Critical	Neurology	OID00369	Dkk-4	Q9UBT3	0.12	-0.32	0.57	3.60	0.58	0.72
Control_v_Critical	Immune	OID01016	CLEC7A	Q9BXN2	0.16	-0.50	0.83	3.24	0.62	0.72
Longitudinal_in_Mild	Cardiovascular	OID00455	BNP	P16860	0.94	-2.11	3.98	1.48	0.50	0.73
Longitudinal_in_Mild	Inflammation	OID00507	FGF-23	Q9GZV9	0.78	-1.64	3.19	2.28	0.48	0.73
Longitudinal_in_Mild	Immune	OID00936	PPP1R9B	Q96SB3	0.68	-1.54	2.90	1.91	0.49	0.73
Longitudinal_in_Mild	Inflammation	OID00521	TRANCE	O14788	0.67	-1.39	2.73	4.84	0.48	0.73
Longitudinal_in_Mild	Inflammation	OID00496	CXCL1	P09341	0.57	-1.27	2.40	9.14	0.50	0.73
Longitudinal_in_Mild	Neurology	OID00305	RGMA	Q96B86	0.49	-1.11	2.09	10.98	0.50	0.73
Longitudinal_in_Mild	Inflammation	OID00528	IL10	P22301	0.48	-1.09	2.05	4.13	0.50	0.73
Longitudinal_in_Mild	Immune	OID00940	IRAK4	Q9NWZ3	-0.47	-2.02	1.08	2.08	0.50	0.73
Longitudinal_in_Mild	Immune	OID01002	SH2D1A	O60880	-0.46	-1.89	0.98	1.70	0.48	0.73
Longitudinal_in_Mild	Inflammation	OID00551	CCL25	O15444	0.42	-0.89	1.72	6.41	0.48	0.73
Longitudinal_in_Mild	Inflammation	OID00556	CCL20	P78556	-0.40	-1.67	0.88	7.59	0.49	0.73
Longitudinal_in_Mild	Neurology	OID00295	CLM-6	Q08708	0.34	-0.73	1.41	6.04	0.47	0.73
Longitudinal_in_Mild	Neurology	OID00354	IL-5R-alpha	Q01344	0.32	-0.69	1.33	3.69	0.48	0.73
Longitudinal_in_Mild	Inflammation	OID00505	CCL11	P51671	0.30	-0.69	1.28	7.88	0.51	0.73
Longitudinal_in_Mild	Inflammation	OID00524	IL-24	Q13007	-0.27	-1.17	0.63	0.86	0.51	0.73
Longitudinal_in_Mild	Cardiovascular	OID00425	MERTK	Q12866	0.25	-0.56	1.06	6.63	0.50	0.73
Longitudinal_in_Mild	Immune	OID00972	EGLN1	Q9GZT9	0.13	-0.30	0.56	1.24	0.49	0.73

Longitudinal_in_Mild	Neurology	OID00288	NRP2	O60462	-0.10	-0.43	0.23	8.24	0.51	0.73
Longitudinal_in_Mild	Inflammation	OID00476	CDCP1	Q9H5V8	-0.69	-3.02	1.63	2.85	0.51	0.73
Longitudinal_in_Mild	Immune	OID00971	MILR1	Q7Z6M3	-0.50	-2.21	1.21	3.32	0.51	0.73
Longitudinal_in_Mild	Neurology	OID00358	DDR1	Q08345	-0.20	-0.88	0.48	7.30	0.51	0.73
Longitudinal_in_Critical	Inflammation	OID00534	CXCL6	P80162	0.37	-0.85	1.59	8.31	0.49	0.73
Longitudinal_in_Critical	Immune	OID00957	TRIM5	Q9C035	-0.22	-0.92	0.49	2.57	0.49	0.73
Longitudinal_in_Critical	Cardiovascular	OID00386	BOC	Q9BWV1	0.14	-0.32	0.61	2.86	0.49	0.73
Longitudinal_in_Mild	Neurology	OID00316	CNTN5	O94779	0.68	-1.69	3.04	4.81	0.52	0.73
Longitudinal_in_Mild	Immune	OID01009	AREG	P15514	-0.37	-1.65	0.91	3.22	0.52	0.73
Longitudinal_in_Mild	Neurology	OID00359	JAM-B	P57087	0.30	-0.73	1.33	7.76	0.52	0.73
Control_v_Severe	Inflammation	OID00504	MCP-4	Q99616	-1.06	-4.19	2.06	15.29	0.49	0.73
Control_v_Critical	Cardiovascular	OID00457	ACE2	Q9BYF1	0.13	-0.42	0.68	3.92	0.64	0.73
Mild_v_Critical	Immune	OID00961	GALNT3	Q14435	0.11	-0.41	0.63	3.26	0.68	0.74
Mild_v_Critical	Inflammation	OID00531	CD5	P06127	0.07	-0.26	0.40	5.32	0.68	0.74
Mild_v_Critical	Neurology	OID00367	SPOCK1	Q08629	0.05	-0.18	0.27	2.55	0.68	0.74
Longitudinal_in_Critical	Inflammation	OID00512	FGF-21	Q9NSA1	0.54	-1.26	2.34	6.19	0.50	0.74
Control_v_Critical	Immune	OID01012	PADI2	Q9Y2J8	0.18	-0.58	0.93	1.62	0.64	0.74
Longitudinal_in_Mild	Inflammation	OID00489	IL-20RA	Q9UHF4	-0.54	-2.45	1.36	1.05	0.53	0.74
Mild_v_Severe	Cardiovascular	OID00438	PSGL-1	Q14242	0.05	-0.17	0.27	4.49	0.66	0.74
Mild_v_Critical	Inflammation	OID00539	CCL28	Q9NRJ3	0.07	-0.26	0.39	2.29	0.69	0.74
Mild_v_Critical	Neurology	OID00297	SMOC2	Q9H3U7	-0.09	-0.56	0.38	7.62	0.69	0.74
Control_v_Critical	Neurology	OID00353	MDGA1	Q8NFP4	-0.22	-1.18	0.74	3.83	0.65	0.74
Longitudinal_in_Critical	Cardiovascular	OID00446	LPL	P06858	-0.15	-0.64	0.35	9.19	0.51	0.75
Longitudinal_in_Mild	Immune	OID00965	LILRB4	Q8NHJ6	-0.54	-2.51	1.42	3.65	0.54	0.75
Control_v_Mild	Inflammation	OID00501	IL18	Q14116	-0.16	-0.83	0.51	9.18	0.63	0.75

Mild_v_Severe	Immune	OID00941	TPSAB1	Q15661	-0.08	-0.46	0.30	4.56	0.67	0.75
Mild_v_Critical	Neurology	OID00290	CADM3	Q8N126	-0.08	-0.51	0.35	3.18	0.70	0.75
Longitudinal_in_Mild	Cardiovascular	OID00419	GLO1	Q04760	-0.44	-2.01	1.14	5.35	0.54	0.75
Severe_v_Critical	Inflammation	OID00558	STAMBP	O95630	0.10	-0.29	0.50	5.03	0.60	0.75
Severe_v_Critical	Immune	OID00966	NTF4	P34130	0.07	-0.20	0.34	1.67	0.60	0.75
Longitudinal_in_Critical	Inflammation	OID00560	ADA	P00813	-0.18	-0.79	0.43	6.32	0.52	0.75
Control_v_Severe	Inflammation	OID00521	TRANCE	O14788	0.58	-1.20	2.35	4.25	0.51	0.75
Control_v_Severe	Immune	OID00956	DPP10	Q8N608	0.56	-1.16	2.27	1.50	0.51	0.75
Control_v_Severe	Neurology	OID00369	Dkk-4	Q9UBT3	0.53	-1.09	2.14	3.64	0.51	0.75
Mild_v_Severe	Cardiovascular	OID00462	TGM2	P21980	0.17	-0.65	0.99	7.89	0.67	0.75
Mild_v_Severe	Cardiovascular	OID00463	LEP	P41159	0.12	-0.46	0.70	5.82	0.67	0.75
Control_v_Mild	Neurology	OID00337	CD200	P41217	0.10	-0.33	0.53	6.35	0.64	0.75
Longitudinal_in_Mild	Neurology	OID00291	GDNF	P39905	-0.24	-1.12	0.65	2.07	0.55	0.75
Longitudinal_in_Mild	Inflammation	OID00562	CSF-1	P09603	-0.08	-0.37	0.21	10.33	0.55	0.75
Longitudinal_in_Mild	Inflammation	OID00560	ADA	P00813	0.26	-0.69	1.20	5.58	0.55	0.75
Longitudinal_in_Critical	Cardiovascular	OID00443	ITGB1BP2	Q9UKP3	0.22	-0.54	0.97	3.38	0.52	0.76
Longitudinal_in_Critical	Inflammation	OID01213	DNER	Q8NFT8	-0.08	-0.35	0.19	8.06	0.52	0.76
Control_v_Mild	Cardiovascular	OID00426	KIM1	Q96D42	0.18	-0.60	0.95	8.18	0.64	0.76
Control_v_Mild	Inflammation	OID00486	CXCL11	O14625	0.25	-0.86	1.37	8.68	0.65	0.76
Longitudinal_in_Critical	Immune	OID00953	PRDX3	P30048	-0.20	-0.92	0.52	0.48	0.53	0.76
Longitudinal_in_Critical	Cardiovascular	OID00418	FS	P19883	0.15	-0.40	0.71	11.15	0.53	0.76
Longitudinal_in_Critical	Cardiovascular	OID00447	PRSS8	Q16651	0.07	-0.18	0.31	8.97	0.53	0.76
Longitudinal_in_Mild	Neurology	OID00351	BMP-4	P12644	0.23	-0.66	1.13	4.85	0.56	0.76
Control_v_Severe	Inflammation	OID00498	CCL4	P13236	-0.90	-3.85	2.06	6.43	0.54	0.76
Control_v_Severe	Cardiovascular	OID00456	MMP12	P39900	-0.66	-2.83	1.51	6.91	0.54	0.76
Control_v_Severe	Inflammation	OID00472	VEGFA	P15692	-0.57	-2.43	1.29	11.52	0.54	0.76
Control_v_Severe	Inflammation	OID00542	CD40	P25942	-0.57	-2.40	1.27	11.76	0.53	0.76

Control_v_Severe	Inflammation	OID00531	CD5	P06127	-0.51	-2.15	1.13	5.31	0.53	0.76
Control_v_Severe	Neurology	OID00293	VWC2	Q2TAL6	-0.50	-2.14	1.14	5.61	0.54	0.76
Control_v_Severe	Inflammation	OID01213	DNER	Q8NFT8	-0.39	-1.62	0.84	8.63	0.53	0.76
Control_v_Severe	Immune	OID00980	HSD11B1	P28845	-0.38	-1.61	0.85	3.00	0.53	0.76
Control_v_Severe	Neurology	OID05024	MAPT	P10636	-0.13	-0.56	0.30	0.30	0.53	0.76
Control_v_Case	Cardiovascular	OID00430	AMBP	P02760	0.02	-0.12	0.17	7.58	0.74	0.76
Control_v_Critical	Inflammation	OID00526	ARTN	Q5T4W7	0.08	-0.28	0.43	1.01	0.67	0.76
Control_v_Critical	Neurology	OID00326	LAYN	Q6UX15	0.11	-0.39	0.60	5.64	0.67	0.76
Control_v_Critical	Inflammation	OID00485	IL-17A	Q16552	0.10	-0.38	0.58	1.66	0.67	0.76
Longitudinal_in_Critical	Immune	OID00979	BIRC2	Q13490	-0.14	-0.67	0.38	0.74	0.54	0.76
Longitudinal_in_Critical	Neurology	OID00295	CLM-6	Q08708	0.11	-0.30	0.52	6.45	0.54	0.76
Control_v_Critical	Neurology	OID00293	VWC2	Q2TAL6	0.09	-0.35	0.53	5.50	0.67	0.76
Mild_v_Critical	Immune	OID00956	DPP10	Q8N608	0.08	-0.36	0.51	1.38	0.72	0.76
Longitudinal_in_Mild	Neurology	OID00324	gal-8	O00214	0.32	-0.91	1.55	5.90	0.56	0.77
Longitudinal_in_Mild	Neurology	OID00342	SCARF2	Q96GP6	0.44	-1.32	2.21	6.05	0.57	0.77
Longitudinal_in_Mild	Inflammation	OID00485	IL-17A	Q16552	-0.34	-1.69	1.00	1.44	0.57	0.77
Longitudinal_in_Mild	Immune	OID00992	CXADR	P78310	-0.24	-1.20	0.72	2.31	0.57	0.77
Longitudinal_in_Mild	Immune	OID00962	FXYD5	Q96DB9	-0.11	-0.55	0.32	0.35	0.57	0.77
Severe_v_Critical	Immune	OID00954	FGF2	P09038	-0.13	-0.68	0.41	0.72	0.62	0.77
Severe_v_Critical	Inflammation	OID00489	IL-20RA	Q9UHF4	-0.08	-0.43	0.26	1.05	0.62	0.77
Mild_v_Critical	Neurology	OID00332	CDH3	P22223	0.07	-0.34	0.49	7.33	0.72	0.77
Severe_v_Critical	Immune	OID00942	HCLS1	P14317	0.18	-0.58	0.94	5.41	0.63	0.77
Severe_v_Critical	Inflammation	OID00530	CCL23	P55773	0.12	-0.40	0.65	11.55	0.63	0.77
Severe_v_Critical	Inflammation	OID00560	ADA	P00813	0.10	-0.32	0.52	6.13	0.63	0.77
Longitudinal_in_Critical	Cardiovascular	OID00469	PARP-1	P09874	0.53	-1.47	2.53	6.26	0.55	0.77
Longitudinal_in_Critical	Immune	OID00951	CLEC4A	Q9UMR7	0.07	-0.19	0.32	3.01	0.55	0.77
Control_v_Critical	Immune	OID00972	EGLN1	Q9GZT9	0.10	-0.38	0.57	1.91	0.69	0.78
Control_v_Critical	Neurology	OID00288	NRP2	O60462	0.04	-0.14	0.21	8.27	0.69	0.78

Control_v_Mild	Inflammation	OID00505	CCL11	P51671	-0.11	-0.63	0.41	7.73	0.67	0.78
Control_v_Mild	Neurology	OID00330	WFIKKN1	Q96NZ8	0.10	-0.38	0.58	3.60	0.67	0.78
Control_v_Mild	Immune	OID01007	NCR1	O76036	-0.08	-0.47	0.31	3.38	0.67	0.78
Mild_v_Severe	Neurology	OID00327	NEP	P08473	0.21	-0.91	1.34	2.77	0.70	0.78
Mild_v_Severe	Cardiovascular	OID00387	IL-4RA	P24394	-0.08	-0.50	0.35	2.10	0.71	0.78
Mild_v_Severe	Cardiovascular	OID00421	PAPPA	Q13219	0.08	-0.34	0.49	2.77	0.71	0.78
Mild_v_Severe	Immune	OID00943	CNTNAP2	Q9UHC6	-0.07	-0.43	0.30	1.40	0.71	0.78
Mild_v_Severe	Cardiovascular	OID00431	PRELP	P51888	0.03	-0.15	0.22	8.35	0.71	0.78
Longitudinal_in_Critical	Cardiovascular	OID00460	hOSCAR	Q8IYS5	0.05	-0.15	0.25	10.98	0.56	0.78
Control_v_Severe	Inflammation	OID00509	FGF-5	P12034	0.28	-0.69	1.26	1.11	0.56	0.78
Severe_v_Critical	Cardiovascular	OID00451	FABP2	P12104	-0.21	-1.16	0.73	7.14	0.65	0.79
Mild_v_Severe	Inflammation	OID00511	LIF-R	P42702	-0.04	-0.28	0.19	4.23	0.72	0.79
Longitudinal_in_Critical	Immune	OID00999	STC1	P52823	-0.19	-0.93	0.55	7.22	0.57	0.79
Longitudinal_in_Critical	Cardiovascular	OID00391	TNFRSF10A	O00220	0.26	-0.77	1.29	4.35	0.57	0.79
Control_v_Mild	Inflammation	OID00526	ARTN	Q5T4W7	0.08	-0.32	0.49	0.88	0.68	0.79
Longitudinal_in_Mild	Neurology	OID00298	NBL1	P41271	-0.06	-0.30	0.18	4.89	0.59	0.79
Control_v_Severe	Cardiovascular	OID00422	SERPINA12	Q8IW75	-1.14	-5.17	2.89	3.04	0.57	0.79
Control_v_Severe	Cardiovascular	OID00415	FGF-23	Q9GZV9	-0.53	-2.40	1.34	2.77	0.57	0.79
Control_v_Severe	Neurology	OID00378	KYNU	Q16719	0.51	-1.28	2.29	9.12	0.57	0.79
Control_v_Critical	Immune	OID01000	ARNT	P27540	-0.13	-0.79	0.53	1.09	0.70	0.79
Control_v_Critical	Neurology	OID00312	sFRP-3	Q92765	0.07	-0.30	0.45	5.59	0.71	0.79
Control_v_Severe	Neurology	OID00335	Beta-NGF	P01138	-0.20	-0.90	0.51	1.29	0.57	0.79
Longitudinal_in_Critical	Cardiovascular	OID00392	STK4	Q13043	0.28	-0.87	1.43	3.29	0.58	0.79
Longitudinal_in_Critical	Cardiovascular	OID00423	REN	P00797	0.24	-0.76	1.25	7.21	0.59	0.79
Longitudinal_in_Critical	Immune	OID00982	PLXNA4	Q9HCM2	0.20	-0.63	1.03	3.62	0.58	0.79
Longitudinal_in_Critical	Inflammation	OID00518	PD-L1	Q9NZQ7	0.18	-0.56	0.92	6.90	0.58	0.79
Longitudinal_in_Critical	Cardiovascular	OID00432	HO-1	P09601	-0.17	-0.88	0.54	12.56	0.59	0.79
Longitudinal_in_Critical	Cardiovascular	OID00425	MERTK	Q12866	-0.13	-0.67	0.40	7.19	0.58	0.79

Longitudinal_in_Critical	Inflammation	OID00558	STAMBP	O95630	-0.09	-0.46	0.28	5.02	0.59	0.79
Longitudinal_in_Critical	Immune	OID00946	EDAR	Q9UNE0	-0.09	-0.45	0.27	1.53	0.58	0.79
Longitudinal_in_Critical	Inflammation	OID00479	OPG	O00300	0.24	-0.76	1.24	10.91	0.59	0.79
Control_v_Critical	Inflammation	OID00489	IL-20RA	Q9UHF4	-0.07	-0.47	0.33	0.95	0.71	0.79
Longitudinal_in_Mild	Neurology	OID00345	TNFRSF12A	Q9NP84	0.41	-1.34	2.16	4.81	0.60	0.79
Control_v_Critical	Immune	OID00970	CCL11	P51671	-0.10	-0.66	0.46	7.24	0.71	0.79
Longitudinal_in_Critical	Neurology	OID00330	WFIKKN1	Q96NZ8	-0.20	-1.06	0.65	3.14	0.60	0.80
Longitudinal_in_Mild	Neurology	OID00293	VWC2	Q2TAL6	0.36	-1.24	1.96	5.58	0.61	0.80
Longitudinal_in_Mild	Neurology	OID00327	NEP	P08473	-0.33	-1.80	1.14	2.55	0.61	0.80
Longitudinal_in_Mild	Inflammation	OID00498	CCL4	P13236	0.30	-0.99	1.59	5.73	0.61	0.80
Longitudinal_in_Mild	Cardiovascular	OID00387	IL-4RA	P24394	0.18	-0.60	0.96	2.22	0.61	0.80
Longitudinal_in_Mild	Cardiovascular	OID00396	TRAIL-R2	O14763	0.15	-0.51	0.81	5.98	0.61	0.80
Longitudinal_in_Mild	Neurology	OID00339	GZMA	P12544	-0.12	-0.67	0.42	6.38	0.62	0.80
Longitudinal_in_Mild	Cardiovascular	OID00462	TGM2	P21980	0.22	-0.77	1.21	8.12	0.62	0.80
Longitudinal_in_Mild	Cardiovascular	OID00430	AMBP	P02760	-0.11	-0.61	0.39	7.71	0.62	0.80
Longitudinal_in_Mild	Cardiovascular	OID00413	SOD2	P04179	0.07	-0.23	0.36	10.13	0.62	0.80
Severe_v_Critical	Inflammation	OID00557	ST1A1	P50225	0.16	-0.59	0.92	1.90	0.66	0.80
Control_v_Critical	Immune	OID01013	SIT1	Q9Y3P8	-0.12	-0.78	0.54	2.32	0.72	0.80
Control_v_Critical	Immune	OID01005	DCBLD2	Q96PD2	0.07	-0.31	0.45	7.86	0.73	0.80
Control_v_Severe	Neurology	OID00353	MDGA1	Q8NFP4	-0.77	-3.66	2.11	3.92	0.59	0.80
Control_v_Severe	Neurology	OID00308	CD38	P28907	0.46	-1.26	2.19	5.72	0.59	0.80
Control_v_Severe	Inflammation	OID00491	CST5	P28325	-0.41	-1.95	1.12	6.64	0.59	0.80
Control_v_Severe	Inflammation	OID00484	MCP-1	P13500	-0.37	-1.77	1.02	11.49	0.59	0.80
Longitudinal_in_Critical	Immune	OID01025	CD83	Q01151	0.12	-0.41	0.66	2.90	0.61	0.81

Control_v_Case	Neurology	OID00304	CRTAM	O95727	0.06	-0.35	0.46	5.32	0.78	0.81
Longitudinal_in_Critical	Immune	OID00998	SPRY2	O43597	-0.45	-2.42	1.53	2.43	0.61	0.81
Longitudinal_in_Mild	Neurology	OID00287	NMNAT1	Q9HAN9	-0.38	-2.19	1.44	3.07	0.64	0.81
Longitudinal_in_Mild	Cardiovascular	OID00423	REN	P00797	-0.34	-1.91	1.24	6.23	0.64	0.81
Longitudinal_in_Mild	Neurology	OID00355	PDGF-R-alpha	P16234	0.25	-0.92	1.41	5.45	0.64	0.81
Longitudinal_in_Mild	Cardiovascular	OID00453	MARCO	Q9UEW3	-0.19	-1.05	0.68	6.96	0.63	0.81
Control_v_Severe	Inflammation	OID05548	TNF	P01375	0.45	-1.28	2.18	3.03	0.60	0.81
Longitudinal_in_Critical	Inflammation	OID00556	CCL20	P78556	0.57	-1.99	3.12	9.70	0.62	0.81
Longitudinal_in_Critical	Immune	OID00950	IRAK1	P51617	-0.17	-0.94	0.60	2.58	0.62	0.81
Mild_v_Severe	Neurology	OID00368	IL12	P29460, P29459	-0.11	-0.80	0.58	8.40	0.75	0.82
Longitudinal_in_Mild	Cardiovascular	OID00404	CXCL1	P09341	0.37	-1.42	2.16	9.87	0.65	0.82
Mild_v_Critical	Neurology	OID00293	VWC2	Q2TAL6	0.06	-0.35	0.47	5.45	0.77	0.82
Longitudinal_in_Critical	Neurology	OID00375	TN-R	Q92752	-0.20	-1.14	0.73	3.77	0.63	0.82
Longitudinal_in_Critical	Neurology	OID00369	Dkk-4	Q9UBT3	-0.08	-0.44	0.29	3.32	0.63	0.82
Mild_v_Critical	Neurology	OID00334	GM-CSF-R-alpha	P15509	-0.09	-0.72	0.54	5.57	0.77	0.82
Longitudinal_in_Critical	Cardiovascular	OID00450	GDF-2	Q9UK05	-0.32	-1.85	1.20	7.25	0.63	0.82
Longitudinal_in_Critical	Immune	OID00938	PSIP1	O75475	-0.27	-1.55	1.00	4.87	0.63	0.82
Control_v_Mild	Cardiovascular	OID00414	CTRC	Q99895	0.15	-0.65	0.95	10.34	0.71	0.82
Control_v_Mild	Neurology	OID00299	EFNA4	P52798	0.05	-0.22	0.32	2.84	0.71	0.82
Mild_v_Severe	Inflammation	OID00499	CD6	P30203	-0.09	-0.68	0.50	6.22	0.75	0.82
Mild_v_Severe	Neurology	OID00367	SPOCK1	Q08629	-0.04	-0.29	0.21	2.45	0.76	0.82
Longitudinal_in_Mild	Neurology	OID00335	Beta-NGF	P01138	-0.16	-0.95	0.64	1.18	0.65	0.82
Severe_v_Critical	Cardiovascular	OID00462	TGM2	P21980	-0.11	-0.64	0.42	8.22	0.68	0.82
Longitudinal_in_Critical	Immune	OID00945	IRF9	Q00978	0.61	-2.36	3.59	2.88	0.64	0.82

Longitudinal_in_Critical	Cardiovascular	OID00387	IL-4RA	P24394	-0.27	-1.54	1.01	3.47	0.64	0.82
Longitudinal_in_Critical	Immune	OID01003	ICA1	Q05084	-0.21	-1.25	0.83	1.30	0.64	0.82
Longitudinal_in_Critical	Immune	OID00965	LILRB4	Q8NHJ6	-0.11	-0.68	0.45	5.47	0.65	0.82
Longitudinal_in_Critical	Cardiovascular	OID00453	MARCO	Q9UEW3	0.11	-0.45	0.67	6.82	0.65	0.83
Longitudinal_in_Critical	Neurology	OID00357	CDH6	P55285	-0.10	-0.62	0.42	3.68	0.65	0.83
Longitudinal_in_Critical	Immune	OID00973	NFATC3	Q12968	-0.08	-0.48	0.32	1.47	0.65	0.83
Longitudinal_in_Critical	Cardiovascular	OID00429	VSIG2	Q96IQ7	0.18	-0.75	1.12	3.62	0.66	0.83
Control_v_Critical	Cardiovascular	OID00463	LEP	P41159	0.21	-1.12	1.55	6.12	0.75	0.83
Control_v_Severe	Inflammation	OID00486	CXCL11	O14625	0.78	-2.32	3.88	9.48	0.61	0.83
Longitudinal_in_Critical	Immune	OID00992	CXADR	P78310	0.19	-0.78	1.15	2.95	0.66	0.83
Longitudinal_in_Critical	Cardiovascular	OID00379	BMP-6	P22004	0.13	-0.56	0.82	5.08	0.67	0.83
Longitudinal_in_Critical	Immune	OID01017	CLEC6A	Q6EIG7	0.06	-0.25	0.37	3.40	0.66	0.83
Control_v_Mild	Cardiovascular	OID00446	LPL	P06858	0.07	-0.35	0.50	9.80	0.72	0.83
Control_v_Mild	Neurology	OID00354	IL-5R-alpha	Q01344	-0.17	-1.11	0.78	3.87	0.73	0.83
Control_v_Mild	Cardiovascular	OID00408	SCF	P21583	-0.07	-0.47	0.33	9.37	0.73	0.83
Control_v_Mild	Inflammation	OID00532	CCL3	P10147	-0.09	-0.63	0.44	5.60	0.73	0.83
Control_v_Severe	Cardiovascular	OID00417	GH	P01241	1.45	-4.44	7.35	8.46	0.62	0.83
Longitudinal_in_Mild	Neurology	OID00309	SMPD1	P17405	-0.22	-1.35	0.92	4.70	0.67	0.83
Control_v_Severe	Cardiovascular	OID00470	HAOX1	Q9UJM8	1.08	-3.56	5.72	5.35	0.64	0.83
Control_v_Severe	Inflammation	OID00522	HGF	P14210	0.53	-1.76	2.82	8.80	0.64	0.83
Control_v_Severe	Immune	OID01006	FCRL6	Q6DN72	0.50	-1.62	2.63	3.04	0.63	0.83
Control_v_Severe	Neurology	OID00341	DRAXIN	Q8NBI3	-0.43	-2.21	1.35	3.28	0.63	0.83
Control_v_Severe	Immune	OID00969	HNMT	P50135	0.42	-1.38	2.21	9.25	0.64	0.83
Control_v_Severe	Neurology	OID00299	EFNA4	P52798	0.27	-0.86	1.39	2.97	0.63	0.83
Control_v_Severe	Neurology	OID00295	CLM-6	Q08708	-0.24	-1.27	0.78	5.97	0.63	0.83
Control_v_Severe	Cardiovascular	OID00427	THBS2	P35442	-0.17	-0.91	0.57	5.54	0.64	0.83
Control_v_Severe	Inflammation	OID00493	IL-1 alpha	P01583	-0.15	-0.76	0.46	0.08	0.62	0.83

Control_v_Mild	Neurology	OID00378	KYNU	Q16719	0.10	-0.50	0.70	8.72	0.74	0.84
Control_v_Mild	Cardiovascular	OID00379	BMP-6	P22004	-0.10	-0.72	0.52	4.63	0.74	0.84
Control_v_Mild	Inflammation	OID00489	IL-20RA	Q9UHF4	-0.05	-0.36	0.26	0.87	0.75	0.84
Longitudinal_in_Critical	Immune	OID01008	CXCL12	P48061	0.05	-0.24	0.35	1.70	0.68	0.84
Control_v_Critical	Immune	OID00984	FCRL3	Q96P31	-0.05	-0.37	0.27	1.08	0.77	0.84
Control_v_Critical	Cardiovascular	OID00400	IL1RL2	Q9HB29	-0.05	-0.36	0.27	4.42	0.77	0.84
Control_v_Mild	Neurology	OID00353	MDGA1	Q8NFP4	0.14	-0.76	1.04	4.21	0.75	0.84
Control_v_Mild	Cardiovascular	OID00455	BNP	P16860	0.10	-0.52	0.71	1.35	0.76	0.84
Control_v_Mild	Neurology	OID00365	TNFRSF21	O75509	0.05	-0.25	0.34	8.25	0.75	0.84
Severe_v_Critical	Immune	OID01002	SH2D1A	O60880	0.12	-0.50	0.74	2.11	0.70	0.84
Longitudinal_in_Mild	Neurology	OID00377	Nr-CAM	Q92823	0.09	-0.40	0.59	9.59	0.67	0.84
Longitudinal_in_Mild	Neurology	OID00301	NCAN	O14594	-0.07	-0.46	0.32	8.82	0.68	0.84
Longitudinal_in_Critical	Immune	OID00994	SRPK2	P78362	-0.18	-1.19	0.82	1.85	0.68	0.84
Mild_v_Severe	Inflammation	OID00481	uPA	P00749	-0.04	-0.32	0.24	9.73	0.78	0.84
Longitudinal_in_Critical	Immune	OID00956	DPP10	Q8N608	-0.10	-0.64	0.45	1.37	0.69	0.84
Longitudinal_in_Critical	Neurology	OID05024	MAPT	P10636	0.09	-0.41	0.58	0.42	0.69	0.84
Longitudinal_in_Critical	Inflammation	OID00517	IL-18R1	Q13478	-0.06	-0.38	0.27	9.70	0.68	0.84
Control_v_Critical	Immune	OID00977	CD28	P10747	-0.06	-0.44	0.33	1.64	0.77	0.84
Control_v_Severe	Cardiovascular	OID00466	CD4	P01730	0.25	-0.88	1.38	4.60	0.65	0.84
Control_v_Severe	Cardiovascular	OID00460	hOSCAR	Q8IYS5	0.22	-0.78	1.22	10.82	0.65	0.84
Longitudinal_in_Mild	Cardiovascular	OID00442	IgG Fc receptor II-b	P31994	0.47	-2.12	3.06	2.68	0.69	0.84
Longitudinal_in_Mild	Neurology	OID00346	SKR3	P37023	0.27	-1.20	1.73	6.70	0.68	0.84
Longitudinal_in_Mild	Cardiovascular	OID00389	IL-1ra	P18510	-0.21	-1.37	0.95	5.63	0.69	0.84
Longitudinal_in_Mild	Immune	OID00985	CKAP4	Q07065	0.15	-0.69	0.99	4.77	0.68	0.84
Longitudinal_in_Mild	Immune	OID00952	PRDX1	Q06830	0.12	-0.58	0.82	1.52	0.69	0.84
Longitudinal_in_Critical	Inflammation	OID00499	CD6	P30203	0.19	-0.93	1.32	5.60	0.70	0.85

Longitudinal_in_Critical	Neurology	OID00315	SIGLEC1	Q9BZZ2	-0.11	-0.74	0.53	7.28	0.70	0.85
Longitudinal_in_Critical	Immune	OID01022	KPNA1	P52294	-0.04	-0.29	0.21	-0.26	0.69	0.85
Control_v_Severe	Neurology	OID00322	HAGH	Q16775	0.74	-2.66	4.15	7.53	0.66	0.85
Control_v_Severe	Neurology	OID00373	LAIR-2	Q6ISS4	0.62	-2.28	3.53	5.10	0.66	0.85
Control_v_Severe	Inflammation	OID00532	CCL3	P10147	-0.53	-2.98	1.92	6.03	0.66	0.85
Control_v_Severe	Cardiovascular	OID00421	PAPPA	Q13219	-0.46	-2.66	1.74	3.09	0.67	0.85
Control_v_Severe	Cardiovascular	OID00400	IL1RL2	Q9HB29	0.29	-1.11	1.69	4.31	0.67	0.85
Control_v_Severe	Inflammation	OID00477	CD244	Q9BZW8	-0.28	-1.62	1.06	5.93	0.67	0.85
Control_v_Severe	Cardiovascular	OID00403	IL-17D	Q8TAD2	-0.28	-1.60	1.04	2.55	0.67	0.85
Control_v_Severe	Immune	OID00985	CKAP4	Q07065	0.27	-1.04	1.58	4.95	0.67	0.85
Severe_v_Critical	Cardiovascular	OID00385	ADAM-TS13	Q76LX8	0.03	-0.12	0.17	4.90	0.71	0.85
Severe_v_Critical	Cardiovascular	OID00413	SOD2	P04179	0.02	-0.07	0.10	10.18	0.71	0.85
Control_v_Critical	Cardiovascular	OID00423	REN	P00797	0.11	-0.65	0.87	6.95	0.78	0.85
Control_v_Critical	Neurology	OID00328	GDF-8	O14793	-0.10	-0.78	0.59	3.14	0.78	0.85
Control_v_Severe	Neurology	OID00355	PDGF-R-alpha	P16234	0.21	-0.82	1.24	5.29	0.68	0.85
Mild_v_Critical	Cardiovascular	OID00411	PIgR	P01833	-0.01	-0.11	0.09	5.70	0.81	0.85
Control_v_Severe	Cardiovascular	OID00442	IgG Fc receptor II-b	P31994	0.59	-2.31	3.49	3.15	0.68	0.85
Severe_v_Critical	Cardiovascular	OID00404	CXCL1	P09341	0.15	-0.69	1.00	10.63	0.71	0.85
Severe_v_Critical	Inflammation	OID00496	CXCL1	P09341	0.14	-0.65	0.93	9.86	0.72	0.85
Severe_v_Critical	Cardiovascular	OID00461	TNFRSF13B	O14836	0.10	-0.45	0.64	10.25	0.72	0.85
Severe_v_Critical	Neurology	OID00375	TN-R	Q92752	-0.06	-0.42	0.29	3.90	0.72	0.85
Longitudinal_in_Mild	Cardiovascular	OID00459	CTSL1	P07711	0.26	-1.24	1.76	7.68	0.70	0.85
Longitudinal_in_Mild	Neurology	OID00370	EDA2R	Q9HAV5	0.25	-1.19	1.69	4.10	0.70	0.85
Control_v_Critical	Cardiovascular	OID00456	MMP12	P39900	0.10	-0.60	0.79	6.84	0.78	0.85
Control_v_Critical	Neurology	OID00305	RGMA	Q96B86	0.05	-0.31	0.40	10.61	0.79	0.85
Longitudinal_in_Mild	Immune	OID01017	CLEC6A	Q6EIG7	-0.27	-1.88	1.34	2.53	0.70	0.85
Longitudinal_in_Mild	Inflammation	OID05548	TNF	P01375	0.24	-1.18	1.67	3.09	0.71	0.85
Severe_v_Critical	Cardiovascular	OID00419	GLO1	Q04760	-0.08	-0.55	0.39	6.29	0.73	0.85
Severe_v_Critical	Immune	OID00959	ITGA6	P23229	0.07	-0.35	0.49	0.72	0.73	0.85
Severe_v_Critical	Neurology	OID00305	RGMA	Q96B86	-0.07	-0.49	0.35	10.19	0.73	0.85

Longitudinal_in_Mild	Immune	OID00994	SRPK2	P78362	0.14	-0.70	0.97	0.48	0.71	0.85
Longitudinal_in_Mild	Immune	OID01007	NCR1	O76036	0.08	-0.42	0.58	3.30	0.71	0.85
Severe_v_Critical	Cardiovascular	OID00449	HB-EGF	Q99075	0.09	-0.47	0.65	6.07	0.74	0.86
Severe_v_Critical	Inflammation	OID00487	AXIN1	O15169	0.09	-0.45	0.62	2.95	0.74	0.86
Severe_v_Critical	Inflammation	OID00491	CST5	P28325	0.07	-0.37	0.51	6.43	0.74	0.86
Severe_v_Critical	Inflammation	OID00515	IL-10RB	Q08334	0.04	-0.22	0.31	6.28	0.74	0.86
Control_v_Mild	Neurology	OID00334	GM-CSF-R-alpha	P15509	-0.08	-0.68	0.51	5.50	0.78	0.86
Control_v_Mild	Inflammation	OID00541	EN-RAGE	P80511	-0.08	-0.66	0.50	2.45	0.78	0.86
Control_v_Mild	Cardiovascular	OID00384	PGF	P49763	0.04	-0.27	0.36	7.41	0.78	0.86
Longitudinal_in_Critical	Cardiovascular	OID00417	GH	P01241	0.29	-1.49	2.07	8.37	0.71	0.86
Control_v_Critical	Neurology	OID00369	Dkk-4	Q9UBT3	-0.07	-0.60	0.47	3.63	0.80	0.86
Control_v_Case	Inflammation	OID00560	ADA	P00813	0.04	-0.36	0.44	5.96	0.84	0.86
Longitudinal_in_Mild	Cardiovascular	OID00407	GIF	P27352	0.25	-1.31	1.80	7.43	0.72	0.86
Longitudinal_in_Mild	Cardiovascular	OID00452	THPO	P40225	0.21	-1.10	1.51	3.72	0.72	0.86
Longitudinal_in_Mild	Neurology	OID00292	UNC5C	O95185	-0.16	-1.19	0.86	4.44	0.72	0.86
Control_v_Mild	Immune	OID01027	BTN3A2	P78410	-0.07	-0.55	0.42	3.03	0.78	0.86
Longitudinal_in_Critical	Cardiovascular	OID00404	CXCL1	P09341	0.21	-1.16	1.59	10.51	0.73	0.86
Longitudinal_in_Critical	Immune	OID00990	MGMT	P16455	0.10	-0.54	0.74	4.20	0.72	0.86
Longitudinal_in_Critical	Neurology	OID00297	SMOC2	Q9H3U7	0.09	-0.49	0.68	7.45	0.72	0.86
Longitudinal_in_Critical	Neurology	OID00294	Siglec-9	Q9Y336	0.05	-0.25	0.34	5.22	0.73	0.86
Control_v_Severe	Neurology	OID00296	EZR	P15311	0.22	-0.91	1.36	4.05	0.69	0.86
Control_v_Mild	Cardiovascular	OID00422	SERPINA12	Q8IW75	0.14	-0.89	1.16	3.34	0.79	0.86
Severe_v_Critical	Cardiovascular	OID00388	SRC	P12931	0.11	-0.58	0.80	5.92	0.75	0.86
Severe_v_Critical	Immune	OID00998	SPRY2	O43597	-0.07	-0.53	0.39	2.23	0.75	0.86
Longitudinal_in_Critical	Immune	OID01011	DAPP1	Q9UN19	0.38	-2.11	2.86	3.07	0.73	0.86
Longitudinal_in_Critical	Inflammation	OID00496	CXCL1	P09341	0.20	-1.12	1.52	9.75	0.73	0.86

Control_v_Severe	Neurology	OID00360	CTSS	P25774	-0.24	-1.54	1.05	5.69	0.70	0.86
Control_v_Severe	Neurology	OID00364	PLXNB1	O43157	-0.22	-1.40	0.95	2.02	0.70	0.86
Control_v_Severe	Immune	OID01008	CXCL12	P48061	0.16	-0.69	1.02	1.58	0.70	0.86
Longitudinal_in_Mild	Immune	OID01010	IFNLR1	Q8IU57	0.04	-0.25	0.34	2.70	0.73	0.87
Control_v_Severe	Inflammation	OID00545	FGF-19	O95750	-0.67	-4.29	2.95	8.48	0.71	0.87
Control_v_Severe	Immune	OID01027	BTN3A2	P78410	-0.44	-2.85	1.96	3.25	0.71	0.87
Control_v_Severe	Neurology	OID00304	CRTAM	O95727	-0.28	-1.82	1.26	5.19	0.71	0.87
Control_v_Severe	Inflammation	OID00562	CSF-1	P09603	0.13	-0.59	0.86	10.26	0.72	0.87
Control_v_Severe	Cardiovascular	OID00418	FS	P19883	-0.36	-2.36	1.64	10.77	0.72	0.87
Severe_v_Critical	Immune	OID01003	ICA1	Q05084	0.05	-0.28	0.38	1.25	0.76	0.87
Severe_v_Critical	Immune	OID00991	TREM1	Q9NP99	0.07	-0.41	0.55	3.17	0.77	0.87
Severe_v_Critical	Immune	OID00962	FXYD5	Q96DB9	0.05	-0.32	0.42	0.69	0.77	0.87
Severe_v_Critical	Immune	OID00943	CNTNAP2	Q9UHC6	-0.04	-0.30	0.23	1.24	0.77	0.87
Control_v_Severe	Immune	OID00977	CD28	P10747	-0.21	-1.41	0.99	1.64	0.73	0.87
Control_v_Mild	Neurology	OID00366	CLM-1	Q8TDQ1	-0.09	-0.81	0.63	6.18	0.80	0.87
Longitudinal_in_Mild	Inflammation	OID00561	TNFB	P01374	-0.18	-1.41	1.05	5.01	0.74	0.87
Longitudinal_in_Critical	Cardiovascular	OID00413	SOD2	P04179	0.03	-0.15	0.20	10.20	0.74	0.87
Control_v_Mild	Inflammation	OID00500	SCF	P21583	-0.05	-0.48	0.37	9.30	0.80	0.88
Control_v_Critical	Cardiovascular	OID00379	BMP-6	P22004	0.09	-0.72	0.90	4.75	0.82	0.88
Control_v_Critical	Cardiovascular	OID00408	SCF	P21583	0.07	-0.55	0.69	8.80	0.82	0.88
Control_v_Critical	Neurology	OID00345	TNFRSF12A	Q9NP84	-0.06	-0.61	0.49	5.36	0.82	0.88
Control_v_Severe	Inflammation	OID00533	Flt3L	P49771	-0.27	-1.82	1.29	9.18	0.73	0.88
Longitudinal_in_Critical	Cardiovascular	OID00422	SERPINA12	Q8IW75	0.19	-1.14	1.52	1.68	0.75	0.88
Mild_v_Critical	Neurology	OID00368	IL12	P29460, P29459	-0.07	-0.71	0.58	8.44	0.84	0.88
Mild_v_Critical	Immune	OID01023	LAG3	P18627	0.04	-0.40	0.49	3.19	0.84	0.88
Mild_v_Critical	Inflammation	OID00551	CCL25	O15444	0.04	-0.38	0.47	6.34	0.84	0.88
Longitudinal_in_Mild	Immune	OID00957	TRIM5	Q9C035	0.18	-1.11	1.48	1.60	0.75	0.88
Control_v_Case	Inflammation	OID00498	CCL4	P13236	0.05	-0.49	0.58	6.22	0.86	0.88
Control_v_Severe	Immune	OID00991	TREM1	Q9NP99	0.29	-1.47	2.06	2.54	0.74	0.88
Control_v_Severe	Neurology	OID00361	N-CDase	Q9NR71	0.36	-1.87	2.59	3.54	0.74	0.88

Control_v_Severe	Immune	OID00995	KLRD1	Q13241	-0.22	-1.60	1.16	6.67	0.75	0.88
Control_v_Severe	Neurology	OID00367	SPOCK1	Q08629	0.17	-0.89	1.24	2.42	0.74	0.88
Longitudinal_in_Mild	Neurology	OID00303	ROBO2	Q9HCK4	0.26	-1.63	2.15	5.66	0.75	0.88
Control_v_Severe	Inflammation	OID00503	TGF-alpha	P01135	0.14	-0.75	1.04	2.83	0.75	0.88
Mild_v_Critical	Cardiovascular	OID00418	FS	P19883	-0.04	-0.43	0.36	10.89	0.85	0.88
Control_v_Case	Neurology	OID00341	DRAXIN	Q8NBI3	0.04	-0.46	0.54	3.48	0.87	0.89
Control_v_Severe	Inflammation	OID00536	4E-BP1	Q13541	0.69	-3.79	5.16	10.16	0.76	0.89
Control_v_Severe	Immune	OID01000	ARNT	P27540	-0.22	-1.65	1.21	0.98	0.76	0.89
Longitudinal_in_Mild	Cardiovascular	OID00440	CCL3	P10147	0.19	-1.18	1.55	6.28	0.76	0.89
Mild_v_Severe	Neurology	OID00352	FcRL2	Q96LA5	-0.06	-0.62	0.50	5.04	0.82	0.89
Severe_v_Critical	Immune	OID00940	IRAK4	Q9NWZ3	-0.09	-0.74	0.57	2.20	0.79	0.89
Severe_v_Critical	Neurology	OID00371	LAT	O43561	-0.08	-0.72	0.55	6.47	0.79	0.89
Severe_v_Critical	Cardiovascular	OID00386	BOC	Q9BWV1	-0.03	-0.29	0.23	2.88	0.79	0.89
Longitudinal_in_Mild	Neurology	OID00302	PRTG	Q2VWP7	0.06	-0.43	0.56	6.51	0.77	0.89
Control_v_Severe	Cardiovascular	OID00433	XCL1	P47992	0.30	-1.75	2.35	4.85	0.77	0.89
Control_v_Severe	Cardiovascular	OID00387	IL-4RA	P24394	-0.17	-1.34	1.00	2.17	0.77	0.89
Severe_v_Critical	Immune	OID01016	CLEC7A	Q9BXN2	0.06	-0.41	0.52	3.44	0.80	0.90
Severe_v_Critical	Inflammation	OID00554	NT-3	P20783	-0.06	-0.51	0.40	1.81	0.80	0.90
Longitudinal_in_Mild	Cardiovascular	OID00398	TIE2	Q02763	-0.08	-0.73	0.56	7.40	0.77	0.90
Longitudinal_in_Critical	Immune	OID00983	SH2B3	Q9UQQ2	-0.27	-2.37	1.82	3.14	0.77	0.90
Control_v_Mild	Cardiovascular	OID00454	GT	P51161	0.06	-0.51	0.63	2.45	0.83	0.90
Control_v_Mild	Immune	OID00966	NTF4	P34130	0.04	-0.29	0.36	1.94	0.83	0.90
Control_v_Mild	Cardiovascular	OID00391	TNFRSF10A	O00220	-0.04	-0.36	0.29	2.82	0.83	0.90
Mild_v_Severe	Inflammation	OID00509	FGF-5	P12034	-0.01	-0.16	0.13	1.05	0.84	0.90
Control_v_Critical	Cardiovascular	OID00468	VEGFD	O43915	0.06	-0.54	0.65	7.62	0.85	0.90
Severe_v_Critical	Neurology	OID00298	NBL1	P41271	0.01	-0.08	0.10	4.92	0.81	0.90
Control_v_Case	Neurology	OID00345	TNFRSF12A	Q9NP84	-0.03	-0.39	0.34	5.15	0.89	0.90
Severe_v_Critical	Cardiovascular	OID00392	STK4	Q13043	-0.12	-1.17	0.93	3.26	0.82	0.90
Severe_v_Critical	Immune	OID00995	KLRD1	Q13241	-0.05	-0.45	0.35	7.11	0.82	0.90
Mild_v_Severe	Inflammation	OID00512	FGF-21	Q9NSA1	0.08	-0.79	0.96	5.50	0.84	0.90
Mild_v_Severe	Neurology	OID00310	MSR1	P21757	0.05	-0.48	0.58	6.66	0.84	0.90

Longitudinal_in_Critical	Neurology	OID00342	SCARF2	Q96GP6	0.06	-0.45	0.58	5.70	0.78	0.90
Longitudinal_in_Critical	Immune	OID00971	MILR1	Q7Z6M3	0.05	-0.33	0.42	3.52	0.78	0.90
Longitudinal_in_Critical	Neurology	OID00288	NRP2	O60462	-0.03	-0.25	0.19	8.32	0.78	0.90
Longitudinal_in_Critical	Neurology	OID00306	PLXNB3	Q9ULL4	-0.10	-0.93	0.73	4.02	0.79	0.91
Mild_v_Critical	Neurology	OID00327	NEP	P08473	0.06	-0.72	0.85	2.79	0.87	0.91
Control_v_Severe	Cardiovascular	OID00441	MMP7	P09237	-0.31	-2.64	2.01	8.59	0.79	0.91
Control_v_Severe	Inflammation	OID00507	FGF-23	Q9GZV9	-0.23	-1.91	1.45	2.10	0.78	0.91
Control_v_Severe	Immune	OID01016	CLEC7A	Q9BXN2	-0.20	-1.67	1.27	3.20	0.78	0.91
Longitudinal_in_Critical	Neurology	OID00345	TNFRSF12A	Q9NP84	0.13	-0.97	1.22	5.64	0.79	0.91
Longitudinal_in_Critical	Neurology	OID00349	CLEC10A	Q8IUN9	-0.12	-1.18	0.94	4.48	0.80	0.91
Longitudinal_in_Critical	Inflammation	OID00481	uPA	P00749	0.09	-0.68	0.86	10.12	0.79	0.91
Longitudinal_in_Critical	Immune	OID00940	IRAK4	Q9NWZ3	0.08	-0.64	0.80	2.19	0.80	0.91
Longitudinal_in_Critical	Immune	OID01014	MASP1	P48740	-0.05	-0.54	0.43	1.33	0.80	0.91
Longitudinal_in_Mild	Inflammation	OID00513	CCL19	Q99731	0.17	-1.25	1.60	9.48	0.79	0.91
Longitudinal_in_Critical	Neurology	OID00376	CD200R1	Q8TD46	-0.08	-0.85	0.68	4.10	0.81	0.91
Longitudinal_in_Critical	Neurology	OID00336	SCARA5	Q6ZMJ2	0.05	-0.45	0.56	8.18	0.81	0.91
Control_v_Mild	Inflammation	OID00502	SLAMF1	Q13291	0.04	-0.41	0.49	2.38	0.85	0.91
Severe_v_Critical	Inflammation	OID00498	CCL4	P13236	-0.07	-0.70	0.57	6.58	0.83	0.91
Severe_v_Critical	Neurology	OID00323	LXN	Q9BS40	-0.03	-0.31	0.25	1.77	0.83	0.91
Longitudinal_in_Mild	Inflammation	OID00549	MCP-2	P80075	0.32	-2.46	3.10	9.97	0.80	0.91
Longitudinal_in_Mild	Inflammation	OID00486	CXCL11	O14625	0.30	-2.36	2.96	8.53	0.80	0.91
Longitudinal_in_Mild	Cardiovascular	OID00454	GT	P51161	0.23	-1.74	2.20	2.76	0.79	0.91
Longitudinal_in_Mild	Immune	OID00993	IL10	P22301	0.09	-0.75	0.93	4.29	0.80	0.91

Longitudinal_in_Mild	Neurology	OID00330	WFIKKN1	Q96NZ8	0.04	-0.32	0.40	3.86	0.80	0.91
Longitudinal_in_Critical	Inflammation	OID00488	TRAIL	P50591	-0.03	-0.36	0.29	6.68	0.81	0.91
Mild_v_Severe	Neurology	OID00315	SIGLEC1	Q9BZZ2	0.06	-0.66	0.79	6.42	0.86	0.92
Control_v_Mild	Neurology	OID00288	NRP2	O60462	-0.01	-0.15	0.13	8.21	0.85	0.92
Severe_v_Critical	Inflammation	OID00509	FGF-5	P12034	-0.02	-0.20	0.17	1.03	0.84	0.92
Control_v_Severe	Cardiovascular	OID00431	PRELP	P51888	-0.08	-0.70	0.55	8.31	0.80	0.92
Control_v_Severe	Neurology	OID00348	CPM	P14384	-0.11	-0.97	0.75	6.64	0.80	0.92
Mild_v_Severe	Cardiovascular	OID00384	PGF	P49763	0.02	-0.27	0.32	7.39	0.86	0.92
Control_v_Critical	Neurology	OID00341	DRAXIN	Q8NBI3	-0.07	-0.90	0.77	3.64	0.87	0.92
Control_v_Critical	Inflammation	OID00514	IL-15RA	Q13261	0.04	-0.41	0.49	1.50	0.87	0.92
Mild_v_Critical	Cardiovascular	OID00393	IDUA	P35475	0.03	-0.34	0.39	5.51	0.89	0.92
Longitudinal_in_Critical	Cardiovascular	OID00452	THPO	P40225	-0.07	-0.77	0.63	4.00	0.82	0.92
Longitudinal_in_Critical	Cardiovascular	OID00398	TIE2	Q02763	0.02	-0.23	0.28	7.40	0.83	0.92
Control_v_Severe	Cardiovascular	OID00379	BMP-6	P22004	-0.46	-4.31	3.40	4.63	0.81	0.92
Control_v_Severe	Cardiovascular	OID00426	KIM1	Q96D42	-0.26	-2.50	1.97	8.44	0.81	0.92
Control_v_Severe	Neurology	OID00294	Siglec-9	Q9Y336	-0.15	-1.42	1.12	4.92	0.81	0.92
Longitudinal_in_Mild	Immune	OID00941	TPSAB1	Q15661	0.25	-2.17	2.66	4.43	0.82	0.92
Longitudinal_in_Critical	Neurology	OID00343	GDNFR-alpha-3	O60609	0.05	-0.52	0.63	4.03	0.83	0.92
Longitudinal_in_Critical	Cardiovascular	OID00435	SORT1	Q99523	-0.05	-0.55	0.46	8.77	0.83	0.92
Control_v_Severe	Neurology	OID00370	EDA2R	Q9HAV5	-0.16	-1.60	1.28	4.53	0.82	0.93
Longitudinal_in_Mild	Neurology	OID00300	SCARB2	Q14108	-0.19	-2.16	1.77	4.88	0.82	0.93
Control_v_Critical	Inflammation	OID00512	FGF-21	Q9NSA1	-0.10	-1.50	1.30	5.85	0.88	0.93
Severe_v_Critical	Neurology	OID00350	GCP5	P78333	0.04	-0.40	0.48	4.20	0.85	0.93
Control_v_Critical	Neurology	OID00321	RSPO1	Q2MKA7	-0.04	-0.57	0.49	3.20	0.89	0.93
Control_v_Severe	Inflammation	OID00501	IL18	Q14116	-0.23	-2.38	1.91	9.60	0.83	0.93
Mild_v_Critical	Inflammation	OID05124	CD8A	P01732	-0.04	-0.72	0.64	10.01	0.90	0.93
Control_v_Critical	Neurology	OID00337	CD200	P41217	-0.03	-0.46	0.40	6.22	0.89	0.93
Control_v_Severe	Inflammation	OID00549	MCP-2	P80075	-0.35	-3.73	3.04	9.64	0.84	0.93
Control_v_Severe	Cardiovascular	OID00451	FABP2	P12104	-0.31	-3.29	2.67	7.86	0.84	0.93

Control_v_Severe	Inflammation	OID00539	CCL28	Q9NRJ3	0.15	-1.32	1.62	2.59	0.84	0.93
Control_v_Severe	Immune	OID01007	NCR1	O76036	-0.13	-1.40	1.15	3.51	0.84	0.93
Control_v_Severe	Cardiovascular	OID00385	ADAM-TS13	Q76LX8	0.07	-0.64	0.78	5.00	0.83	0.93
Control_v_Critical	Immune	OID01021	ITGA11	Q9UKX5	-0.02	-0.35	0.30	2.74	0.89	0.93
Control_v_Mild	Immune	OID01009	AREG	P15514	0.05	-0.60	0.71	3.31	0.87	0.93
Longitudinal_in_Critical	Immune	OID01020	TANK	Q92844	-0.15	-1.92	1.62	1.66	0.85	0.93
Longitudinal_in_Critical	Cardiovascular	OID00389	IL-1ra	P18510	0.07	-0.79	0.93	7.43	0.85	0.93
Longitudinal_in_Critical	Neurology	OID00314	RGMB	Q6NW40	0.04	-0.43	0.51	5.62	0.85	0.93
Severe_v_Critical	Inflammation	OID00494	OSM	P13725	-0.06	-0.90	0.78	6.36	0.88	0.94
Severe_v_Critical	Inflammation	OID00536	4E-BP1	Q13541	0.05	-0.63	0.73	9.12	0.88	0.94
Severe_v_Critical	Inflammation	OID00523	IL-12B	P29460	0.04	-0.47	0.55	6.79	0.86	0.94
Severe_v_Critical	Inflammation	OID00480	LAP TGF-beta-1	P01137	-0.04	-0.55	0.47	6.66	0.87	0.94
Severe_v_Critical	Neurology	OID00368	IL12	P29460, P29459	-0.04	-0.55	0.47	8.53	0.88	0.94
Severe_v_Critical	Immune	OID00949	CLEC4C	Q8WTT0	-0.03	-0.50	0.43	3.24	0.88	0.94
Severe_v_Critical	Inflammation	OID00499	CD6	P30203	0.03	-0.41	0.47	5.73	0.89	0.94
Severe_v_Critical	Inflammation	OID00561	TNFB	P01374	0.03	-0.31	0.37	4.35	0.86	0.94
Severe_v_Critical	Neurology	OID00349	CLEC10A	Q8IUN9	-0.03	-0.37	0.32	4.50	0.88	0.94
Severe_v_Critical	Inflammation	OID00539	CCL28	Q9NRJ3	0.02	-0.25	0.29	2.25	0.88	0.94
Severe_v_Critical	Immune	OID01005	DCBLD2	Q96PD2	-0.02	-0.29	0.25	7.91	0.89	0.94
Longitudinal_in_Mild	Cardiovascular	OID00415	FGF-23	Q9GZV9	0.24	-2.25	2.73	3.15	0.83	0.94
Control_v_Severe	Neurology	OID00349	CLEC10A	Q8IUN9	-0.13	-1.46	1.21	5.05	0.85	0.94
Control_v_Severe	Cardiovascular	OID00468	VEGFD	O43915	-0.12	-1.40	1.16	7.79	0.85	0.94
Longitudinal_in_Critical	Cardiovascular	OID00442	IgG Fc receptor II-b	P31994	0.05	-0.55	0.65	3.47	0.85	0.94
Mild_v_Severe	Immune	OID00937	GLB1	P16278	0.03	-0.32	0.37	0.81	0.88	0.94
Control_v_Critical	Neurology	OID00368	IL12	P29460, P29459	0.05	-0.73	0.83	8.40	0.90	0.94
Longitudinal_in_Mild	Immune	OID00990	MGMT	P16455	-0.33	-4.07	3.41	3.82	0.84	0.94
Longitudinal_in_Mild	Cardiovascular	OID00424	DECR1	Q16698	-0.28	-3.42	2.85	3.46	0.84	0.94
Longitudinal_in_Mild	Neurology	OID00333	GFR-alpha-1	P56159	0.13	-1.32	1.58	7.11	0.84	0.94

Mild_v_Severe	Cardiovascular	OID00470	HAOX1	Q9UJM8	0.08	-1.14	1.30	5.88	0.89	0.94
Mild_v_Severe	Cardiovascular	OID00410	FGF-21	Q9NSA1	0.07	-0.98	1.12	6.83	0.89	0.94
Mild_v_Severe	Neurology	OID00326	LAYN	Q6UX15	-0.03	-0.47	0.41	5.24	0.90	0.94
Mild_v_Severe	Neurology	OID00292	UNC5C	O95185	-0.02	-0.40	0.35	4.39	0.89	0.94
Control_v_Severe	Immune	OID00960	CDSN	Q15517	-0.16	-1.89	1.58	2.94	0.85	0.94
Longitudinal_in_Critical	Inflammation	OID00549	MCP-2	P80075	0.11	-1.26	1.47	10.44	0.86	0.94
Control_v_Severe	Inflammation	OID00554	NT-3	P20783	0.16	-1.67	2.00	2.44	0.86	0.94
Mild_v_Severe	Inflammation	OID00523	IL-12B	P29460	-0.04	-0.62	0.55	6.60	0.90	0.94
Mild_v_Severe	Inflammation	OID00552	CX3CL1	P78423	0.02	-0.27	0.31	4.19	0.90	0.94
Control_v_Case	Neurology	OID00373	LAIR-2	Q6ISS4	0.02	-0.52	0.57	5.23	0.93	0.94
Severe_v_Critical	Cardiovascular	OID00401	PDGF subunit B	P01127	-0.04	-0.72	0.63	10.15	0.90	0.94
Control_v_Severe	Cardiovascular	OID00393	IDUA	P35475	0.17	-1.80	2.14	5.77	0.86	0.94
Longitudinal_in_Critical	Inflammation	OID00483	IL-17C	Q9P0M4	-0.08	-1.14	0.99	3.31	0.87	0.94
Longitudinal_in_Critical	Neurology	OID00290	CADM3	Q8N126	0.02	-0.30	0.35	3.08	0.87	0.94
Longitudinal_in_Critical	Neurology	OID00298	NBL1	P41271	-0.01	-0.20	0.17	4.91	0.87	0.94
Mild_v_Critical	Immune	OID01025	CD83	Q01151	0.02	-0.30	0.33	2.93	0.92	0.94
Mild_v_Critical	Neurology	OID00329	THY 1	P04216	0.01	-0.26	0.28	9.86	0.92	0.94
Longitudinal_in_Mild	Immune	OID00968	ITM2A	O43736	0.43	-5.03	5.89	2.63	0.86	0.95
Longitudinal_in_Mild	Neurology	OID00337	CD200	P41217	0.10	-1.21	1.42	6.48	0.86	0.95
Longitudinal_in_Mild	Inflammation	OID00532	CCL3	P10147	0.10	-1.12	1.32	5.63	0.86	0.95
Longitudinal_in_Mild	Immune	OID00967	KRT19	P08727	-0.09	-1.15	0.97	2.13	0.85	0.95
Control_v_Severe	Inflammation	OID00483	IL-17C	Q9P0M4	0.20	-2.23	2.63	2.45	0.87	0.95
Control_v_Mild	Neurology	OID00312	sFRP-3	Q92765	0.02	-0.32	0.37	5.38	0.89	0.95
Longitudinal_in_Critical	Immune	OID01023	LAG3	P18627	-0.04	-0.71	0.62	3.17	0.88	0.95
Longitudinal_in_Critical	Neurology	OID00374	MANF	P55145	-0.06	-0.94	0.83	7.65	0.88	0.95
Control_v_Severe	Immune	OID01012	PADI2	Q9Y2J8	-0.13	-1.83	1.56	1.39	0.87	0.95
Longitudinal_in_Mild	Neurology	OID00322	HAGH	Q16775	-0.21	-3.06	2.64	5.76	0.86	0.95

Control_v_Severe	Cardiovascular	OID00463	LEP	P41159	-0.34	-4.86	4.18	5.92	0.88	0.95
Control_v_Severe	Inflammation	OID00505	CCL11	P51671	-0.13	-1.94	1.68	7.62	0.88	0.95
Control_v_Severe	Inflammation	OID00530	CCL23	P55773	0.13	-1.64	1.89	10.52	0.88	0.95
Control_v_Severe	Neurology	OID00331	TMPRSS5	Q9H3S3	0.10	-1.29	1.48	2.64	0.89	0.95
Control_v_Severe	Inflammation	OID00481	uPA	P00749	-0.08	-1.26	1.10	9.68	0.89	0.95
Longitudinal_in_Mild	Cardiovascular	OID00447	PRSS8	Q16651	-0.08	-1.22	1.05	8.85	0.87	0.95
Control_v_Critical	Inflammation	OID00500	SCF	P21583	-0.03	-0.63	0.57	8.74	0.92	0.95
Control_v_Critical	Inflammation	OID00561	TNFB	P01374	-0.03	-0.54	0.49	4.47	0.92	0.95
Control_v_Mild	Inflammation	OID00508	IL-10RA	Q13651	-0.02	-0.38	0.34	1.03	0.90	0.96
Control_v_Mild	Immune	OID01016	CLEC7A	Q9BXN2	-0.03	-0.47	0.42	3.10	0.90	0.96
Mild_v_Severe	Inflammation	OID00527	MMP-10	P09238	-0.02	-0.46	0.41	9.03	0.92	0.96
Longitudinal_in_Critical	Inflammation	OID00489	IL-20RA	Q9UHF4	0.06	-1.00	1.13	1.17	0.89	0.96
Control_v_Mild	Immune	OID00988	CLEC4D	Q8WXI8	-0.03	-0.62	0.55	3.41	0.91	0.96
Control_v_Mild	Neurology	OID00375	TN-R	Q92752	0.03	-0.57	0.63	4.31	0.91	0.96
Control_v_Mild	Neurology	OID00294	Siglec-9	Q9Y336	0.02	-0.37	0.41	4.90	0.91	0.96
Longitudinal_in_Critical	Immune	OID00985	CKAP4	Q07065	-0.07	-1.38	1.24	6.97	0.90	0.96
Longitudinal_in_Critical	Inflammation	OID00550	CASP-8	Q14790	-0.04	-0.82	0.74	4.22	0.90	0.96
Longitudinal_in_Critical	Immune	OID01026	ITGB6	P18564	0.02	-0.35	0.39	3.08	0.90	0.96
Longitudinal_in_Critical	Cardiovascular	OID00458	PD-L2	Q9BQ51	-0.01	-0.20	0.18	3.53	0.90	0.96
Control_v_Critical	Cardiovascular	OID00433	XCL1	P47992	-0.03	-0.66	0.61	5.19	0.93	0.96
Longitudinal_in_Critical	Cardiovascular	OID00397	PRSS27	Q9BQR3	0.03	-0.65	0.72	8.19	0.91	0.96
Control_v_Severe	Neurology	OID00307	CPA2	P48052	0.13	-2.07	2.33	9.91	0.90	0.96
Control_v_Severe	Immune	OID00978	PTH1R	Q03431	0.08	-1.27	1.43	3.92	0.90	0.96
Longitudinal_in_Mild	Cardiovascular	OID00460	hOSCAR	Q8IYS5	0.02	-0.30	0.34	10.73	0.88	0.96
Control_v_Mild	Neurology	OID00349	CLEC10A	Q8IUN9	0.02	-0.46	0.51	5.17	0.92	0.96
Longitudinal_in_Critical	Immune	OID00991	TREM1	Q9NP99	0.04	-0.83	0.91	3.26	0.92	0.96
Longitudinal_in_Critical	Immune	OID00948	DGKZ	Q13574	-0.03	-0.69	0.63	0.99	0.92	0.96
Control_v_Mild	Neurology	OID00293	VWC2	Q2TAL6	-0.03	-0.57	0.51	5.49	0.92	0.96

Longitudinal_in_Critical	Cardiovascular	OID00454	GT	P51161	-0.07	-1.59	1.46	2.24	0.92	0.97
Severe_v_Critical	Inflammation	OID00520	CXCL5	P42830	-0.05	-1.11	1.01	10.43	0.92	0.97
Control_v_Critical	Immune	OID00968	ITM2A	O43736	-0.03	-1.03	0.97	2.47	0.95	0.97
Control_v_Critical	Immune	OID00941	TPSAB1	Q15661	-0.02	-0.68	0.64	4.65	0.94	0.97
Control_v_Critical	Immune	OID00945	IRF9	Q00978	-0.02	-0.65	0.61	2.85	0.94	0.97
Longitudinal_in_Critical	Immune	OID01010	IFNLR1	Q8IU57	0.03	-0.73	0.79	3.02	0.93	0.97
Control_v_Case	Cardiovascular	OID00415	FGF-23	Q9GZV9	-0.01	-0.39	0.37	2.88	0.96	0.97
Severe_v_Critical	Cardiovascular	OID00403	IL-17D	Q8TAD2	0.01	-0.27	0.29	2.24	0.93	0.97
Severe_v_Critical	Cardiovascular	OID00395	PAR-1	P25116	0.02	-0.39	0.42	8.69	0.93	0.97
Control_v_Critical	Cardiovascular	OID00429	VSIG2	Q96IQ7	0.02	-0.60	0.63	3.93	0.95	0.97
Control_v_Critical	Cardiovascular	OID00430	AMBP	P02760	-0.01	-0.27	0.26	7.58	0.95	0.97
Longitudinal_in_Mild	Inflammation	OID00506	TNFSF14	O43557	0.14	-2.29	2.57	4.40	0.90	0.97
Longitudinal_in_Mild	Cardiovascular	OID00422	SERPINA12	Q8IW75	0.09	-1.47	1.65	3.98	0.89	0.97
Longitudinal_in_Mild	Immune	OID00970	CCL11	P51671	0.06	-1.00	1.11	7.44	0.90	0.97
Longitudinal_in_Mild	Neurology	OID00376	CD200R1	Q8TD46	0.04	-0.63	0.70	4.48	0.90	0.97
Longitudinal_in_Mild	Immune	OID00939	ZBTB16	Q05516	-0.03	-0.59	0.53	0.73	0.90	0.97
Control_v_Critical	Cardiovascular	OID00410	FGF-21	Q9NSA1	-0.04	-1.60	1.52	7.22	0.96	0.97
Control_v_Critical	Immune	OID01007	NCR1	O76036	0.01	-0.45	0.48	3.64	0.96	0.97
Longitudinal_in_Mild	Cardiovascular	OID00469	PARP-1	P09874	-0.08	-1.68	1.52	4.29	0.91	0.97
Control_v_Critical	Cardiovascular	OID00465	HSP 27	P04792	-0.01	-0.40	0.39	10.29	0.97	0.97
Longitudinal_in_Critical	Inflammation	OID05547	IFN-gamma	P01579	0.04	-1.26	1.34	10.58	0.94	0.97
Longitudinal_in_Critical	Immune	OID00949	CLEC4C	Q8WTT0	0.02	-0.44	0.47	3.20	0.94	0.97
Longitudinal_in_Mild	Cardiovascular	OID00432	HO-1	P09601	-0.09	-2.01	1.82	11.67	0.91	0.97
Longitudinal_in_Mild	Cardiovascular	OID00463	LEP	P41159	0.05	-0.96	1.06	6.05	0.91	0.97
Longitudinal_in_Mild	Neurology	OID00332	CDH3	P22223	0.04	-0.93	1.02	7.36	0.92	0.97
Control_v_Severe	Neurology	OID00330	WFIKKN1	Q96NZ8	-0.06	-1.28	1.16	3.41	0.92	0.98

Control_v_Severe	Neurology	OID00352	FcRL2	Q96LA5	-0.08	-1.80	1.64	4.95	0.93	0.98
Control_v_Severe	Inflammation	OID00561	TNFB	P01374	-0.07	-1.77	1.62	4.52	0.93	0.98
Control_v_Severe	Neurology	OID00325	BCAN	Q96GW7	-0.06	-1.46	1.34	4.31	0.93	0.98
Severe_v_Critical	Neurology	OID00330	WFIKKN1	Q96NZ8	-0.01	-0.30	0.28	3.09	0.94	0.98
Control_v_Case	Inflammation	OID00507	FGF-23	Q9GZV9	-0.01	-0.41	0.39	2.15	0.97	0.98
Mild_v_Severe	Immune	OID00971	MILR1	Q7Z6M3	-0.02	-0.45	0.42	3.19	0.94	0.98
Longitudinal_in_Critical	Inflammation	OID00471	IL8	P10145	0.04	-1.37	1.45	7.30	0.95	0.98
Longitudinal_in_Critical	Cardiovascular	OID00459	CTSL1	P07711	-0.02	-0.54	0.50	9.36	0.95	0.98
Longitudinal_in_Critical	Cardiovascular	OID00427	THBS2	P35442	-0.01	-0.41	0.39	5.74	0.95	0.98
Longitudinal_in_Critical	Inflammation	OID00554	NT-3	P20783	0.02	-0.77	0.81	1.64	0.95	0.98
Mild_v_Critical	Immune	OID01011	DAPP1	Q9UN19	-0.02	-0.89	0.84	2.71	0.96	0.98
Control_v_Mild	Neurology	OID00332	CDH3	P22223	0.01	-0.31	0.33	7.24	0.94	0.98
Severe_v_Critical	Neurology	OID00319	ADAM 23	O75077	0.01	-0.35	0.37	3.56	0.95	0.98
Severe_v_Critical	Cardiovascular	OID00439	CCL17	Q92583	-0.03	-0.88	0.83	8.21	0.95	0.98
Severe_v_Critical	Cardiovascular	OID00432	HO-1	P09601	-0.01	-0.39	0.37	12.46	0.95	0.98
Control_v_Severe	Cardiovascular	OID00413	SOD2	P04179	0.02	-0.41	0.44	10.25	0.94	0.98
Severe_v_Critical	Immune	OID00948	DGKZ	Q13574	0.00	-0.10	0.10	0.70	0.96	0.98
Control_v_Mild	Inflammation	OID00527	MMP-10	P09238	-0.02	-0.58	0.54	9.12	0.95	0.98
Control_v_Mild	Cardiovascular	OID00457	ACE2	Q9BYF1	-0.01	-0.48	0.45	3.33	0.95	0.98
Control_v_Mild	Inflammation	OID00509	FGF-5	P12034	0.01	-0.23	0.25	1.07	0.95	0.98
Longitudinal_in_Mild	Inflammation	OID00515	IL-10RB	Q08334	-0.03	-0.82	0.75	6.18	0.93	0.98
Longitudinal_in_Mild	Immune	OID00981	NF2	P35240	0.01	-0.32	0.35	-0.49	0.93	0.98
Control_v_Critical	Neurology	OID00330	WFIKKN1	Q96NZ8	-0.01	-0.51	0.50	3.33	0.98	0.98
Longitudinal_in_Critical	Neurology	OID00304	CRTAM	O95727	0.03	-1.54	1.61	5.15	0.96	0.98
Longitudinal_in_Critical	Cardiovascular	OID00434	IL16	Q14005	0.01	-0.56	0.58	7.11	0.97	0.98
Longitudinal_in_Critical	Cardiovascular	OID00438	PSGL-1	Q14242	0.01	-0.31	0.32	4.26	0.97	0.98
Control_v_Severe	Cardiovascular	OID00423	REN	P00797	0.09	-2.62	2.81	6.49	0.94	0.98
Control_v_Severe	Cardiovascular	OID00462	TGM2	P21980	0.09	-2.60	2.79	8.61	0.95	0.98

Control_v_Severe	Neurology	OID00334	GM-CSF-R-alpha	P15509	0.05	-1.71	1.81	5.40	0.95	0.98
Control_v_Severe	Inflammation	OID00560	ADA	P00813	-0.05	-1.70	1.61	6.17	0.95	0.98
Control_v_Severe	Inflammation	OID00479	OPG	O00300	0.04	-1.28	1.36	10.24	0.95	0.98
Control_v_Severe	Neurology	OID00287	NMNAT1	Q9HAN9	0.09	-3.70	3.87	3.57	0.96	0.98
Control_v_Severe	Immune	OID00997	PIK3AP1	Q6ZUJ8	0.08	-3.33	3.49	4.65	0.96	0.98
Control_v_Severe	Cardiovascular	OID00429	VSIG2	Q96IQ7	-0.05	-2.06	1.96	3.79	0.96	0.98
Control_v_Critical	Cardiovascular	OID00411	PIgR	P01833	0.00	-0.17	0.18	5.74	0.98	0.99
Control_v_Mild	Cardiovascular	OID00410	FGF-21	Q9NSA1	0.02	-1.05	1.09	6.70	0.97	0.99
Control_v_Mild	Cardiovascular	OID00429	VSIG2	Q96IQ7	0.01	-0.48	0.50	3.79	0.97	0.99
Control_v_Mild	Inflammation	OID00507	FGF-23	Q9GZV9	0.01	-0.53	0.55	2.04	0.97	0.99
Control_v_Mild	Immune	OID01024	IL5	P05113	-0.01	-0.45	0.43	0.95	0.97	0.99
Control_v_Mild	Cardiovascular	OID00385	ADAM-TS13	Q76LX8	0.00	-0.15	0.16	5.07	0.96	0.99
Control_v_Mild	Neurology	OID00335	Beta-NGF	P01138	0.00	-0.21	0.21	1.21	0.97	0.99
Severe_v_Critical	Cardiovascular	OID00397	PRSS27	Q9BQR3	0.01	-0.32	0.34	8.19	0.97	0.99
Longitudinal_in_Critical	Inflammation	OID00474	MCP-3	P80098	-0.01	-1.55	1.52	5.85	0.98	0.99
Longitudinal_in_Critical	Cardiovascular	OID00415	FGF-23	Q9GZV9	0.01	-0.96	0.98	3.31	0.98	0.99
Mild_v_Severe	Inflammation	OID00526	ARTN	Q5T4W7	-0.01	-0.37	0.36	0.88	0.96	1.00
Severe_v_Critical	Immune	OID00997	PIK3AP1	Q6ZUJ8	-0.01	-0.70	0.68	3.38	0.98	1.00
Severe_v_Critical	Cardiovascular	OID00438	PSGL-1	Q14242	0.00	-0.13	0.14	4.35	0.98	1.00
Longitudinal_in_Critical	Immune	OID01000	ARNT	P27540	0.00	-0.18	0.18	0.93	0.99	1.00
Mild_v_Critical	Inflammation	OID00523	IL-12B	P29460	0.01	-0.65	0.67	6.68	0.98	1.00
Control_v_Case	Inflammation	OID00547	LIF	P15018	0.00	-0.03	0.03	0.43	0.99	1.00
Severe_v_Critical	Neurology	OID00328	GDF-8	O14793	-0.01	-0.63	0.62	2.50	0.98	1.00
Control_v_Case	Immune	OID01005	DCBLD2	Q96PD2	0.00	-0.25	0.25	7.90	1.00	1.00
Control_v_Case	Neurology	OID05024	MAPT	P10636	0.00	-0.03	0.03	0.31	1.00	1.00
Control_v_Severe	Neurology	OID00310	MSR1	P21757	0.02	-1.52	1.56	6.52	0.98	1.00
Control_v_Mild	Cardiovascular	OID00442	IgG Fc receptor II-b	P31994	0.01	-1.01	1.02	3.00	0.99	1.00
Control_v_Severe	Cardiovascular	OID00430	AMBP	P02760	-0.01	-0.88	0.86	7.58	0.99	1.00
Control_v_Severe	Immune	OID01005	DCBLD2	Q96PD2	0.01	-0.93	0.95	7.86	0.99	1.00
Control_v_Severe	Inflammation	OID00555	TWEAK	O43508	0.01	-0.81	0.83	8.89	0.99	1.00
Control_v_Mild	Inflammation	OID00512	FGF-21	Q9NSA1	0.01	-1.08	1.09	5.39	0.99	1.00
Control_v_Critical	Neurology	OID05024	MAPT	P10636	0.00	-0.09	0.09	0.33	1.00	1.00

Longitudinal_in_Mild	Neurology	OID00363	N2DL-2	Q9BZM5	0.03	-1.18	1.24	2.93	0.95	1.00
Severe_v_Critical	Immune	OID01020	TANK	Q92844	0.01	-0.73	0.74	1.51	0.99	1.00
Severe_v_Critical	Immune	OID01011	DAPP1	Q9UN19	0.00	-1.01	1.01	2.71	1.00	1.00
Severe_v_Critical	Immune	OID00981	NF2	P35240	0.00	-0.07	0.07	-0.47	1.00	1.00
Severe_v_Critical	Immune	OID00989	PRKCQ	Q04759	0.00	-0.06	0.06	0.78	1.00	1.00
Severe_v_Critical	Inflammation	OID00493	IL-1 alpha	P01583	0.00	-0.06	0.06	-0.13	1.00	1.00
Longitudinal_in_Mild	Cardiovascular	OID00402	IL-27	Q8NEV9, Q14213	0.03	-1.18	1.23	6.18	0.96	1.00
Longitudinal_in_Mild	Immune	OID00996	BACH1	O14867	-0.02	-0.64	0.61	1.14	0.96	1.00
Longitudinal_in_Mild	Immune	OID01003	ICA1	Q05084	-0.01	-0.46	0.44	0.87	0.96	1.00
Longitudinal_in_Mild	Neurology	OID00364	PLXNB1	O43157	0.01	-0.31	0.32	1.60	0.96	1.00
Mild_v_Critical	Immune	OID00984	FCRL3	Q96P31	0.00	-0.29	0.30	1.01	0.99	1.00
Mild_v_Critical	Cardiovascular	OID00392	STK4	Q13043	0.00	-0.97	0.97	3.00	1.00	1.00
Mild_v_Critical	Neurology	OID05024	MAPT	P10636	0.00	-0.04	0.04	0.32	1.00	1.00
Mild_v_Critical	Immune	OID00948	DGKZ	Q13574	0.00	-0.06	0.06	0.63	1.00	1.00
Mild_v_Critical	Immune	OID00989	PRKCQ	Q04759	0.00	-0.04	0.04	0.74	1.00	1.00
Mild_v_Critical	Inflammation	OID00493	IL-1 alpha	P01583	0.00	-0.04	0.04	-0.06	1.00	1.00
Mild_v_Critical	Immune	OID00981	NF2	P35240	0.00	-0.05	0.05	-0.49	1.00	1.00
Mild_v_Severe	Neurology	OID00306	PLXNB3	Q9ULL4	-0.01	-0.35	0.33	3.88	0.97	1.00
Mild_v_Severe	Inflammation	OID00531	CD5	P06127	-0.01	-0.36	0.35	5.34	0.98	1.00
Mild_v_Severe	Neurology	OID00341	DRAXIN	Q8NBI3	0.00	-0.31	0.32	3.24	0.98	1.00
Mild_v_Severe	Immune	OID00984	FCRL3	Q96P31	0.00	-0.31	0.31	0.97	1.00	1.00
Mild_v_Severe	Immune	OID01000	ARNT	P27540	0.00	-0.06	0.06	0.75	1.00	1.00
Mild_v_Severe	Inflammation	OID00493	IL-1 alpha	P01583	0.00	-0.04	0.04	-0.05	1.00	1.00
Mild_v_Severe	Immune	OID00948	DGKZ	Q13574	0.00	-0.05	0.05	0.53	1.00	1.00
Mild_v_Severe	Immune	OID00981	NF2	P35240	0.00	-0.04	0.04	-0.57	1.00	1.00
Mild_v_Severe	Inflammation	OID00547	LIF	P15018	0.00	-0.04	0.04	0.27	1.00	1.00
Mild_v_Severe	Immune	OID00989	PRKCQ	Q04759	0.00	-0.04	0.04	0.66	1.00	1.00
Mild_v_Severe	Immune	OID01022	KPNA1	P52294	0.00	-0.05	0.05	-0.26	1.00	1.00
Control_v_Mild	Cardiovascular	OID00423	REN	P00797	0.00	-0.50	0.50	6.46	1.00	1.00
Control_v_Mild	Neurology	OID05024	MAPT	P10636	0.00	-0.07	0.07	0.26	1.00	1.00
Control_v_Mild	Inflammation	OID00547	LIF	P15018	0.00	-0.07	0.07	0.29	1.00	1.00

Control_v_Severe	Immune	OID00970	CCL11	P51671	-0.01	-1.95	1.93	7.20	0.99	1.00
Control_v_Severe	Cardiovascular	OID00398	TIE2	Q02763	0.00	-1.18	1.19	7.32	1.00	1.00
Control_v_Severe	Neurology	OID00329	THY 1	P04216	0.00	-1.11	1.11	9.96	1.00	1.00
Control_v_Severe	Inflammation	OID00547	LIF	P15018	0.00	-0.32	0.32	0.33	1.00	1.00
Longitudinal_in_Mild	Inflammation	OID00475	GDNF	P39905	0.02	-2.11	2.15	2.38	0.98	1.00
Longitudinal_in_Mild	Neurology	OID00365	TNFRSF21	O75509	-0.01	-1.20	1.17	8.30	0.98	1.00
Longitudinal_in_Mild	Cardiovascular	OID00412	RAGE	Q15109	-0.01	-1.33	1.31	13.56	0.99	1.00
Longitudinal_in_Mild	Inflammation	OID00518	PD-L1	Q9NZQ7	0.01	-1.19	1.20	5.74	0.99	1.00
Longitudinal_in_Mild	Cardiovascular	OID00450	GDF-2	Q9UK05	0.00	-1.19	1.19	8.94	1.00	1.00
Longitudinal_in_Mild	Immune	OID00973	NFATC3	Q12968	0.00	-1.79	1.80	0.76	1.00	1.00
Longitudinal_in_Mild	Immune	OID00979	BIRC2	Q13490	0.00	-0.24	0.24	0.40	1.00	1.00
Longitudinal_in_Mild	Inflammation	OID00493	IL-1 alpha	P01583	0.00	-0.20	0.20	-0.14	1.00	1.00
Longitudinal_in_Mild	Immune	OID01000	ARNT	P27540	0.00	-0.30	0.30	0.60	1.00	1.00
Longitudinal_in_Mild	Immune	OID00989	PRKCQ	Q04759	0.00	-0.28	0.28	0.70	1.00	1.00
Longitudinal_in_Mild	Inflammation	OID00547	LIF	P15018	0.00	-0.20	0.20	0.25	1.00	1.00
Longitudinal_in_Mild	Immune	OID00948	DGKZ	Q13574	0.00	-0.22	0.22	0.51	1.00	1.00
Longitudinal_in_Mild	Neurology	OID05024	MAPT	P10636	0.00	-0.23	0.23	0.20	1.00	1.00
Longitudinal_in_Critical	Immune	OID00963	TRAF2	Q12933	0.00	-0.58	0.58	1.93	1.00	1.00
Longitudinal_in_Critical	Immune	OID00989	PRKCQ	Q04759	0.00	-0.18	0.18	1.06	1.00	1.00
Longitudinal_in_Critical	Inflammation	OID00493	IL-1 alpha	P01583	0.00	-0.15	0.15	-0.14	1.00	1.00

Supplementary Table 3: Pathway enrichment analysis results

Pathway	p-value	q-value	size	effective_size	source
Cytokine-cytokine receptor interaction - Homo sapiens (human)	2.05E-44	1.72E-41	294	294	KEGG
Cytokine Signaling in Immune system	2.00E-19	8.42E-17	458	458	Reactome
Immune System	3.25E-16	9.13E-14	1840	1825	Reactome
Interleukin-10 signaling	6.20E-15	1.20E-12	38	38	Wikipathways
Rheumatoid arthritis - Homo sapiens (human)	7.09E-15	1.20E-12	90	89	KEGG
IL-17 signaling pathway - Homo sapiens (human)	1.68E-14	2.36E-12	93	93	KEGG
Signaling by Interleukins	1.30E-13	1.56E-11	254	254	Reactome
Chemokine receptors bind chemokines	4.87E-13	5.13E-11	50	50	Reactome
JAK STAT MolecularVariation 1	5.87E-13	5.50E-11	98	98	INOH
Cytokines and Inflammatory Response	8.68E-13	7.31E-11	29	26	Wikipathways
Lung fibrosis	9.59E-13	7.35E-11	64	63	Wikipathways
PI3K-Akt signaling pathway - Homo sapiens (human)	2.74E-12	1.83E-10	354	353	KEGG
Chemokine signaling pathway - Homo sapiens (human)	2.83E-12	1.83E-10	189	189	KEGG
NF-kappa B signaling pathway - Homo sapiens (human)	3.43E-12	2.06E-10	95	94	KEGG
PI3K-Akt Signaling Pathway	5.22E-12	2.82E-10	340	339	Wikipathways
GPCR signaling-G alpha q	5.36E-12	2.82E-10	274	274	INOH
GPCR signaling-G alpha i	1.16E-11	5.43E-10	262	262	INOH
GPCR signaling-pertussis toxin	1.16E-11	5.43E-10	262	262	INOH
GPCR signaling-G alpha s PKA and ERK	1.35E-11	5.99E-10	285	285	INOH
GPCR signaling-cholera toxin	2.29E-11	9.65E-10	270	270	INOH
GPCR signaling-G alpha s Epac and ERK	2.71E-11	1.09E-09	272	272	INOH
IL23-mediated signaling events	7.92E-11	3.04E-09	37	37	PID
Malaria - Homo sapiens (human)	1.33E-10	4.88E-09	49	49	KEGG
JAK-STAT-Core	1.92E-10	6.74E-09	104	104	Signalink
Hematopoietic cell lineage - Homo sapiens (human)	5.75E-10	1.94E-08	97	96	KEGG
Interleukin-4 and Interleukin-13 signaling	6.68E-10	2.17E-08	97	97	Wikipathways
MAPK signaling pathway - Homo sapiens (human)	8.65E-10	2.70E-08	295	295	KEGG
LTF danger signal response pathway	1.31E-09	3.96E-08	19	19	Wikipathways
Differentiation Pathway	1.73E-09	5.02E-08	48	48	Wikipathways
JAK STAT pathway and regulation	2.42E-09	6.80E-08	310	310	INOH
Allograft Rejection	2.70E-09	7.27E-08	91	91	Wikipathways
TNFR2 non-canonical NF-kB pathway	2.76E-09	7.27E-08	50	50	Reactome
induction of apoptosis through dr3 and dr4/5 death receptors	7.96E-09	2.03E-07	23	23	BioCarta

JAK-STAT signaling pathway - Homo sapiens (human)	2.11E-08	5.23E-07	162	162	KEGG
miRNAs involvement in the immune response in sepsis	2.38E-08	5.64E-07	64	36	Wikipathways
IL27-mediated signaling events	2.41E-08	5.64E-07	26	26	PID
Viral Acute Myocarditis	2.79E-08	6.36E-07	76	76	Wikipathways
Focal Adhesion-PI3K-Akt-mTOR-signaling pathway	3.19E-08	7.09E-07	302	302	Wikipathways
TNF signaling pathway - Homo sapiens (human)	3.32E-08	7.17E-07	110	110	KEGG
Spinal Cord Injury	5.89E-08	1.24E-06	117	115	Wikipathways
Hypertrophy Model	6.79E-08	1.40E-06	20	20	Wikipathways
Ras signaling pathway - Homo sapiens (human)	1.71E-07	3.44E-06	232	232	KEGG
RIPK1-mediated regulated necrosis	3.83E-07	7.21E-06	16	16	Reactome
Regulated Necrosis	3.83E-07	7.21E-06	16	16	Reactome
TRAIL signaling pathway	3.87E-07	7.21E-06	25	25	PID
Fibrin Complement Receptor 3 Signaling Pathway	3.93E-07	7.21E-06	36	36	Wikipathways
Apoptosis - Homo sapiens (human)	4.86E-07	8.72E-06	136	136	KEGG
Protein alkylation leading to liver fibrosis	4.97E-07	8.72E-06	50	50	Wikipathways
Inflammatory bowel disease (IBD) - Homo sapiens (human)	5.28E-07	9.09E-06	65	65	KEGG
Pathways in cancer - Homo sapiens (human)	5.47E-07	9.22E-06	526	525	KEGG
TNF receptor superfamily (TNFSF) members mediating non-canonical NF-kB pathway	5.82E-07	9.63E-06	17	17	Reactome
Chagas disease (American trypanosomiasis) - Homo sapiens (human)	7.71E-07	1.25E-05	102	102	KEGG
Chemokine signaling pathway	9.47E-07	1.49E-05	165	165	Wikipathways
C-type lectin receptor signaling pathway - Homo sapiens (human)	9.53E-07	1.49E-05	104	104	KEGG
Dimerization of procaspase-8	1.22E-06	1.77E-05	11	11	Reactome
Regulation by c-FLIP	1.22E-06	1.77E-05	11	11	Reactome
CASP8 activity is inhibited	1.22E-06	1.77E-05	11	11	Reactome
Regulation of necroptotic cell death	1.22E-06	1.77E-05	11	11	Reactome
Osteoclast differentiation - Homo sapiens (human)	1.46E-06	2.08E-05	128	128	KEGG
Influenza A - Homo sapiens (human)	1.49E-06	2.08E-05	171	171	KEGG
Peptide ligand-binding receptors	1.51E-06	2.08E-05	194	194	Reactome
T-Cell antigen Receptor (TCR) Signaling Pathway	1.53E-06	2.08E-05	92	90	Wikipathways
Measles - Homo sapiens (human)	1.91E-06	2.52E-05	132	131	KEGG
amb2 Integrin signaling	1.91E-06	2.52E-05	31	31	PID
SHP2 signaling	2.13E-06	2.77E-05	59	59	PID
Development and heterogeneity of the ILC family	2.41E-06	3.07E-05	32	32	Wikipathways
RIG-I-like Receptor Signaling	2.47E-06	3.10E-05	60	60	Wikipathways

T-Cell antigen Receptor (TCR) pathway during Staphylococcus aureus infection	2.84E-06	3.52E-05	63	61	Wikipathways
il-10 anti-inflammatory signaling pathway	3.29E-06	4.02E-05	13	13	BioCarta
Intestinal immune network for IgA production - Homo sapiens (human)	4.00E-06	4.81E-05	49	48	KEGG
Photodynamic therapy-induced NF-kB survival signaling	4.57E-06	5.43E-05	35	35	Wikipathways
Toll-like Receptor Signaling Pathway	5.31E-06	6.22E-05	102	102	Wikipathways
Immunoregulatory interactions between a Lymphoid and a non-Lymphoid cell	5.42E-06	6.26E-05	221	214	Reactome
Cell adhesion molecules (CAMs) - Homo sapiens (human)	5.50E-06	6.26E-05	144	144	KEGG
IL1 and megakaryocytes in obesity	5.64E-06	6.34E-05	24	24	Wikipathways
Toll-like receptor signaling pathway - Homo sapiens (human)	6.42E-06	7.13E-05	104	104	KEGG
signal transduction through il1r	6.77E-06	7.41E-05	37	37	BioCarta
TRAIL signaling	9.84E-06	0.00010633	8	8	Reactome
Ligand-dependent caspase activation	1.06E-05	0.00011359	16	16	Reactome
TNF related weak inducer of apoptosis (TWEAK) Signaling Pathway	1.38E-05	0.00014553	41	41	Wikipathways
Toxoplasmosis - Homo sapiens (human)	1.43E-05	0.00014904	113	113	KEGG
Rap1 signaling pathway - Homo sapiens (human)	1.47E-05	0.00015105	206	206	KEGG
Caspase Cascade in Apoptosis	1.50E-05	0.00015243	57	57	PID
Pertussis - Homo sapiens (human)	1.80E-05	0.00018111	76	76	KEGG
Regulation of toll-like receptor signaling pathway	1.99E-05	0.0001969	143	139	Wikipathways
Interleukin-1 family signaling	2.01E-05	0.0001969	77	77	Reactome
PI5P, PP2A and IER3 Regulate PI3K/AKT Signaling	2.13E-05	0.00020668	97	97	Reactome
AGE-RAGE signaling pathway in diabetic complications - Homo sapiens (human)	2.55E-05	0.00024452	99	99	KEGG
NOD-like receptor signaling pathway - Homo sapiens (human)	2.91E-05	0.00027549	168	168	KEGG
IL12-mediated signaling events	3.18E-05	0.00029746	65	63	PID
Adaptive Immune System	3.82E-05	0.0003542	732	722	Reactome
Negative regulation of the PI3K/AKT network	3.92E-05	0.00035941	104	104	Reactome
Apoptosis	4.06E-05	0.00036844	87	84	Wikipathways
Senescence and Autophagy in Cancer	4.26E-05	0.00038214	106	105	Wikipathways
MAPK1/MAPK3 signaling	4.90E-05	0.00043452	203	202	Reactome
Th17 cell differentiation - Homo sapiens (human)	5.01E-05	0.00044027	107	107	KEGG
Photodynamic therapy-induced AP-1 survival signaling.	5.27E-05	0.00045827	50	50	Wikipathways
FAS (CD95) signaling pathway	5.66E-05	0.00048728	35	35	PID
Constitutive Signaling by Aberrant PI3K in Cancer	6.19E-05	0.00052716	69	69	Reactome
RIG-I-like receptor signaling pathway - Homo sapiens (human)	6.87E-05	0.0005793	70	70	KEGG
EBV LMP1 signaling	7.32E-05	0.00060505	23	23	Wikipathways

Regulation of TNFR1 signaling	7.32E-05	0.00060505	23	23	Reactome
Extracellular matrix organization	7.40E-05	0.00060593	294	293	Reactome
Validated transcriptional targets of AP1 family members Fra1 and Fra2	7.85E-05	0.00063624	37	37	PID
Cell surface interactions at the vascular wall	8.69E-05	0.00069797	219	213	Reactome
Necroptosis - Homo sapiens (human)	9.01E-05	0.00071627	162	162	KEGG
ECM proteoglycans	0.000110691	0.00086401	57	56	Reactome
DDX58/IFIH1-mediated induction of interferon-alpha/beta	0.000110691	0.00086401	56	56	Reactome
Osteoblast Signaling	0.000128087	0.00099062	14	14	Wikipathways
Caspase activation via extrinsic apoptotic signalling pathway	0.00013634	0.00101712	26	26	Reactome
Other interleukin signaling	0.00013634	0.00101712	26	26	Reactome
Syndecan-3-mediated signaling events	0.00013634	0.00101712	26	26	PID
TRAF6 mediated NF-kB activation	0.00013634	0.00101712	26	26	Reactome
RAF/MAP kinase cascade	0.000141819	0.00104164	197	196	Reactome
Syndecan-2-mediated signaling events	0.000142098	0.00104164	41	41	PID
Senescence-Associated Secretory Phenotype (SASP)	0.000146451	0.0010643	6	6	Wikipathways
Focal Adhesion	0.000156934	0.00113073	198	198	Wikipathways
Focal adhesion - Homo sapiens (human)	0.000164998	0.00117876	199	199	KEGG
T cell receptor signaling pathway - Homo sapiens (human)	0.000172295	0.00121085	101	101	KEGG
Interleukin-1 signaling	0.000172363	0.00121085	60	60	Reactome
TWEAK	0.000197229	0.00137408	28	28	NetPath
Selenium Micronutrient Network	0.000211613	0.00145505	83	82	Wikipathways
Urokinase-type plasminogen activator (uPA) and uPAR-mediated signaling	0.000212303	0.00145505	44	44	PID
Tuberculosis - Homo sapiens (human)	0.000219375	0.00149139	179	178	KEGG
NAD+ metabolism	0.000225731	0.00149836	16	16	Wikipathways
Osteoclast Signaling	0.000225731	0.00149836	16	16	Wikipathways
bone remodeling	0.000225731	0.00149836	16	16	BioCarta
C-type lectin receptors (CLRs)	0.000230247	0.00150906	83	83	Reactome
MAPK family signaling cascades	0.000233702	0.00150906	237	234	Reactome
TNFs bind their physiological receptors	0.000234503	0.00150906	29	29	Reactome
Dectin-2 family	0.000234503	0.00150906	29	29	Reactome
Natural killer cell mediated cytotoxicity - Homo sapiens (human)	0.000255681	0.00163287	131	130	KEGG
TNF receptor signaling pathway	0.000272566	0.00172762	46	46	PID
IL12 signaling mediated by STAT4	0.000276901	0.001742	32	30	PID
Hemostasis	0.000287216	0.00179351	668	662	Reactome

AGE-RAGE pathway	0.000314314	0.00194829	66	66	Wikipathways
NTF3 activates NTRK2 (TRKB) signaling	0.000389923	0.00239931	2	2	Reactome
VEGF binds to VEGFR leading to receptor dimerization	0.000398164	0.00241477	8	8	Reactome
VEGF ligand-receptor interactions	0.000398164	0.00241477	8	8	Reactome
Apoptosis Modulation and Signaling	0.000401879	0.00241988	91	90	Wikipathways
Fluid shear stress and atherosclerosis - Homo sapiens (human)	0.000413449	0.00245449	139	138	KEGG
Death Receptor Signalling	0.000413449	0.00245449	141	138	Reactome
JAK STAT MolecularVariation 2	0.000432902	0.002552	50	50	INOH
Ectoderm Differentiation	0.000437904	0.00255458	142	139	Wikipathways
ceramide signaling pathway	0.0004394	0.00255458	33	33	BioCarta
p75(NTR)-mediated signaling	0.000452481	0.00259484	71	70	PID
AP-1 transcription factor network	0.000452481	0.00259484	70	70	PID
Overview of nanoparticle effects	0.000458786	0.00261322	19	19	Wikipathways
TNF alpha Signaling Pathway	0.000466511	0.00263939	93	92	Wikipathways
African trypanosomiasis - Homo sapiens (human)	0.000506931	0.00284896	35	34	KEGG
Nuclear Receptors Meta-Pathway	0.000538352	0.00298898	316	315	Wikipathways
PI3K/AKT Signaling in Cancer	0.000539333	0.00298898	94	94	Reactome
Human cytomegalovirus infection - Homo sapiens (human)	0.000542483	0.00298898	225	225	KEGG
Apoptosis	0.000551645	0.00301972	118	118	Reactome
Proteoglycans in cancer - Homo sapiens (human)	0.000578395	0.00314572	201	198	KEGG
Leishmaniasis - Homo sapiens (human)	0.000584906	0.00315611	74	73	KEGG
Role Altered Glycolysation of MUC1 in Tumour Microenvironment	0.00058853	0.00315611	9	9	Wikipathways
Posttranslational regulation of adherens junction stability and dissassembly	0.000595281	0.00315611	53	53	PID
hiv-1 nef: negative effector of fas and tnf	0.000595281	0.00315611	53	53	BioCarta
Heparan sulfate/heparin (HS-GAG) metabolism	0.000658764	0.00343956	54	54	Reactome
Programmed Cell Death	0.000662229	0.00343956	121	121	Reactome
Primary immunodeficiency - Homo sapiens (human)	0.000665062	0.00343956	37	36	KEGG
IL1-mediated signaling events	0.000665062	0.00343956	36	36	PID
nf-kb signaling pathway	0.000686724	0.00352993	21	21	BioCarta
Legionellosis - Homo sapiens (human)	0.000727368	0.0036938	55	55	KEGG
RANKL-RANK (Receptor activator of NFKB (ligand)) Signaling Pathway	0.000727368	0.0036938	55	55	Wikipathways
Lysosome - Homo sapiens (human)	0.000745548	0.00376346	123	123	KEGG
TNF signaling	0.000756714	0.00379708	37	37	Reactome
Antigen processing and presentation - Homo sapiens (human)	0.000807471	0.0040278	77	77	KEGG

NAD+ biosynthetic pathways	0.000826389	0.00408435	22	22	Wikipathways
VEGF and VEGFR signaling network	0.000828497	0.00408435	10	10	PID
CXCR3-mediated signaling events	0.000857477	0.00417834	38	38	PID
Signaling events mediated by TCPTP	0.000857477	0.00417834	38	38	PID
Class A/1 (Rhodopsin-like receptors)	0.000886872	0.00429674	330	330	Reactome
Toll-Like Receptors Cascades	0.000974675	0.00469105	154	154	Reactome
caspase cascade in apoptosis	0.000984954	0.00469105	23	23	BioCarta
tnf/stress related signaling	0.000984954	0.00469105	23	23	BioCarta
Hematopoietic Stem Cell Differentiation	0.001088558	0.00515536	49	40	Wikipathways
Ebola Virus Pathway on Host	0.001107083	0.0052138	130	130	Wikipathways
Dermatan sulfate biosynthesis	0.001122589	0.00525746	11	11	Reactome
Kaposi sarcoma-associated herpesvirus infection - Homo sapiens (human)	0.001188766	0.00553663	186	186	KEGG
Graft-versus-host disease - Homo sapiens (human)	0.001220011	0.00562005	41	41	KEGG
Netrin-1 signaling	0.001220011	0.00562005	41	41	Reactome
Fas Ligand (FasL) pathway and Stress induction of Heat Shock Proteins (HSP) regulation	0.001362841	0.00621014	43	42	Wikipathways
Non-integrin membrane-ECM interactions	0.001362841	0.00621014	42	42	Reactome
Platelet-mediated interactions with vascular and circulating cells	0.001475002	0.00664934	12	12	Wikipathways
NF-kB activation through FADD/RIP-1 pathway mediated by caspase-8 and -10	0.001475002	0.00664934	12	12	Reactome
Salmonella infection - Homo sapiens (human)	0.001552705	0.00696239	86	86	KEGG
Glypican 1 network	0.001587323	0.00700583	26	26	PID
TNFR1-induced NFkappaB signaling pathway	0.001587323	0.00700583	26	26	Reactome
A tetrasaccharide linker sequence is required for GAG synthesis	0.001587323	0.00700583	26	26	Reactome
Beta3 integrin cell surface interactions	0.001684986	0.00739814	44	44	PID
IL4-mediated signaling events	0.001758751	0.00768201	65	65	PID
Control of immune tolerance by vasoactive intestinalpeptide	0.001889623	0.008103	13	13	Wikipathways
the 41bb-dependent immune response	0.001889623	0.008103	13	13	BioCarta
TNFR1-induced proapoptotic signaling	0.001889623	0.008103	13	13	Reactome
Signaling by Receptor Tyrosine Kinases	0.001897883	0.008103	423	423	Reactome
Beta1 integrin cell surface interactions	0.001903195	0.008103	66	66	PID
Innate Immune System	0.002026965	0.00858659	1077	1067	Reactome
Epithelial cell signaling in Helicobacter pylori infection - Homo sapiens (human)	0.002218838	0.00930587	68	68	KEGG
Non-genomic actions of 1,25 dihydroxyvitamin D3	0.002218838	0.00930587	68	68	Wikipathways
Thymic Stromal LymphoPoietin (TSLP) Signaling Pathway	0.002268369	0.00946301	47	47	Wikipathways
Activated NTRK2 signals through FYN	0.002278756	0.00946301	4	4	Reactome

Th1 and Th2 cell differentiation - Homo sapiens (human)	0.002290976	0.00946712	92	92	KEGG
nfkb activation by nontypeable hemophilus influenzae	0.00240737	0.00989958	29	29	BioCarta
G alpha (i) signalling events	0.002488292	0.01014934	400	399	Reactome
Neurotrophin signaling pathway - Homo sapiens (human)	0.002492186	0.01014934	119	119	KEGG
Calcineurin-regulated NFAT-dependent transcription in lymphocytes	0.002731125	0.01092758	49	49	PID
Asthma - Homo sapiens (human)	0.002735135	0.01092758	31	30	KEGG
Beta2 integrin cell surface interactions	0.002735135	0.01092758	30	30	PID
Extracellular vesicle-mediated signaling in recipient cells	0.002735135	0.01092758	30	30	Wikipathways
Amoebiasis - Homo sapiens (human)	0.002749159	0.0109318	96	95	KEGG
ErbB receptor signaling network	0.002919562	0.0114474	15	15	PID
il12 and stat4 dependent signaling pathway in th1 development	0.002919562	0.0114474	15	15	BioCarta
Mammary gland development pathway - Embryonic development (Stage 1 of 4)	0.002919562	0.0114474	18	15	Wikipathways
Chondroitin sulfate/dermatan sulfate metabolism	0.002986483	0.01165558	50	50	Reactome
Toll Like Receptor 3 (TLR3) Cascade	0.003091609	0.01201026	97	97	Reactome
TAK1 activates NFkB by phosphorylation and activation of IKKs complex	0.00347983	0.01345641	32	32	Reactome
TLR4 Signaling and Tolerance	0.003541216	0.0135346	16	16	Wikipathways
Regulation of KIT signaling	0.003541216	0.0135346	16	16	Reactome
Apoptotic execution phase	0.003548217	0.0135346	52	52	Reactome
Toll Like Receptor 4 (TLR4) Cascade	0.003723551	0.0141074	127	127	Reactome
Interleukin-18 signaling	0.003748384	0.0141074	5	5	Reactome
Sudden Infant Death Syndrome (SIDS) Susceptibility Pathways	0.003853624	0.0141074	159	156	Wikipathways
Cardiac Progenitor Differentiation	0.003855815	0.0141074	53	53	Wikipathways
keratinocyte differentiation	0.003855815	0.0141074	53	53	BioCarta
Signaling events mediated by PTP1B	0.003855815	0.0141074	53	53	PID
Apoptosis-related network due to altered Notch3 in ovarian cancer	0.003855815	0.0141074	53	53	Wikipathways
TRIF(TICAM1)-mediated TLR4 signaling	0.003873487	0.0141074	101	101	Reactome
MyD88-independent TLR4 cascade	0.003873487	0.0141074	101	101	Reactome
Inflammatory Response Pathway	0.0038992	0.0141074	33	33	Wikipathways
Resistin as a regulator of inflammation	0.0038992	0.0141074	33	33	Wikipathways
NOD1/2 Signaling Pathway	0.0038992	0.0141074	33	33	Reactome
tnfr1 signaling pathway	0.004237772	0.01520188	17	17	BioCarta
TRAF6 mediated IRF7 activation	0.004237772	0.01520188	17	17	Reactome
Type 2 papillary renal cell carcinoma	0.004351502	0.01554371	34	34	Wikipathways
IL-1 signaling pathway	0.004527512	0.01610419	55	55	Wikipathways

HIV-1 Nef: Negative effector of Fas and TNF-alpha	0.004837892	0.0170642	35	35	PID
RET signaling	0.004837892	0.0170642	35	35	Reactome
Axon guidance	0.004868085	0.01709915	358	356	Reactome
FGF signaling pathway	0.00489282	0.01711472	56	56	PID
Platelet activation, signaling and aggregation	0.005482328	0.01909753	260	259	Reactome
Activation of TRKA receptors	0.005549351	0.01917255	6	6	Reactome
Robo4 and VEGF Signaling Pathways Crosstalk	0.005549351	0.01917255	6	6	Wikipathways
Developmental Biology	0.005601128	0.01927245	620	618	Reactome
Scavenging by Class A Receptors	0.00586543	0.02001845	19	19	Reactome
Interleukin-12 family signaling	0.00586543	0.02001845	19	19	Reactome
Allograft rejection - Homo sapiens (human)	0.005917409	0.02003364	38	37	KEGG
Type II interferon signaling (IFNG)	0.005917409	0.02003364	37	37	Wikipathways
Hypertrophic cardiomyopathy (HCM) - Homo sapiens (human)	0.005990303	0.0201993	83	83	KEGG
Signal Transduction	0.006088178	0.02044755	2647	2634	Reactome
Epstein-Barr virus infection - Homo sapiens (human)	0.006497562	0.02173589	201	200	KEGG
ATF-2 transcription factor network	0.006564356	0.02178643	60	60	PID
TYROBP Causal Network	0.006564356	0.02178643	60	60	Wikipathways
TGF-beta signaling pathway - Homo sapiens (human)	0.006718905	0.02213564	85	85	KEGG
Signaling by NTRK2 (TRKB)	0.006800866	0.02213564	20	20	Reactome
Angiogenesis	0.006800866	0.02213564	20	20	Wikipathways
Syndecan interactions	0.006800866	0.02213564	20	20	Reactome
Chondroitin sulfate biosynthesis	0.006800866	0.02213564	20	20	Reactome
Integrated Lung Cancer Pathway	0.007146385	0.02317078	40	39	Wikipathways
NRF2 pathway	0.007247368	0.02340817	142	142	Wikipathways
Interleukin-1 processing	0.007668074	0.02439316	7	7	Reactome
PDGF receptor signaling network	0.007668074	0.02439316	7	7	PID
Nectin/Necl trans heterodimerization	0.007668074	0.02439316	7	7	Reactome
ATF4 activates genes	0.007668074	0.02439316	7	7	Wikipathways
Bladder Cancer	0.007819477	0.02445354	40	40	Wikipathways
inactivation of gsk3 by akt causes accumulation of b-catenin in alveolar macrophages	0.007819477	0.02445354	40	40	BioCarta
Signaling by FGFR3	0.007819477	0.02445354	40	40	Reactome
HS-GAG degradation	0.007819889	0.02445354	21	21	Reactome
Brain-Derived Neurotrophic Factor (BDNF) signaling pathway	0.007861103	0.02445354	144	144	Wikipathways
Hepatitis B - Homo sapiens (human)	0.007861103	0.02445354	144	144	KEGG

Endochondral Ossification	0.00805513	0.02478276	63	63	Wikipathways
Cytosolic DNA-sensing pathway - Homo sapiens (human)	0.00805513	0.02478276	63	63	KEGG
Integrins in angiogenesis	0.00805513	0.02478276	63	63	PID
Bladder cancer - Homo sapiens (human)	0.008532933	0.02615566	41	41	KEGG
TCR	0.008563418	0.02615566	245	241	NetPath
miRNA targets in ECM and membrane receptors	0.008924126	0.026868	44	22	Wikipathways
ras-independent pathway in nk cell-mediated cytotoxicity	0.008924126	0.026868	22	22	BioCarta
Interleukin-6 family signaling	0.008924126	0.026868	22	22	Reactome
Vitamin D in inflammatory diseases	0.008924126	0.026868	22	22	Wikipathways
Oncostatin M Signaling Pathway	0.009170369	0.02741355	65	65	Wikipathways
Folate Metabolism	0.009170369	0.02741355	66	65	Wikipathways
Glycosaminoglycan metabolism	0.009367864	0.02790498	119	119	Reactome
MAPK Signaling Pathway	0.009901216	0.02920187	246	246	Wikipathways
MicroRNAs in cancer - Homo sapiens (human)	0.009937794	0.02920187	299	150	KEGG

Supplementary Table 4: Correlation of Nfl, GFAP and Tau with OLINK proteins

**SIMOA protein**	**OLINK.ID**	**OLINK Assay**	**Protein.ID**	**Uniprot.ID**	**cor**	**p-value**
NfL	OID00370	Neurology	EDA2R	Q9HAV5	6.62E-01	4.01E-12
NfL	OID00394	Cardiovascular	TNFRSF11A	Q9Y6Q6	6.53E-01	6.85E-12
NfL	OID00396	Cardiovascular	TRAIL-R2	O14763	6.53E-01	7.17E-12
NfL	OID00346	Neurology	SKR3	P37023	6.45E-01	1.96E-11
NfL	OID00479	Inflammation	OPG	O00300	0.64029747	2.42E-11
NfL	OID00326	Neurology	LAYN	Q6UX15	0.64229111	2.64E-11
NfL	OID00985	Immune	CKAP4	Q07065	0.64159379	2.81E-11
NfL	OID00321	Neurology	RSPO1	Q2MKA7	0.63255216	6.44E-11
NfL	OID00518	Inflammation	PD-L1	Q9NZQ7	0.62256724	1.21E-10
NfL	OID00967	Immune	KRT19	P08727	0.62315534	1.48E-10
NfL	OID00363	Neurology	N2DL-2	Q9BZM5	0.61904391	2.11E-10
NfL	OID00391	Cardiovascular	TNFRSF10A	O00220	0.61509748	2.32E-10
NfL	OID00490	Inflammation	CXCL9	Q07325	0.6141179	2.52E-10
NfL	OID00399	Cardiovascular	TF	P13726	0.61225085	2.95E-10
NfL	OID00969	Immune	HNMT	P50135	0.61490993	3.00E-10
NfL	OID00384	Cardiovascular	PGF	P49763	0.61013459	3.53E-10

NfL	OID00300	Neurology	SCARB2	Q14108	0.6099896	4.54E-10
NfL	OID00542	Inflammation	CD40	P25942	0.60621755	4.90E-10
NfL	OID00552	Inflammation	CX3CL1	P78423	0.60429094	5.75E-10
NfL	OID00991	Immune	TREM1	Q9NP99	0.60692713	5.85E-10
NfL	OID00532	Inflammation	CCL3	P10147	0.59633993	1.10E-09
NfL	OID00471	Inflammation	IL8	P10145	0.59596196	1.13E-09
NfL	OID00299	Neurology	EFNA4	P52798	0.59854158	1.15E-09
NfL	OID00521	Inflammation	TRANCE	O14788	-0.5885273	2.04E-09
NfL	OID00345	Neurology	TNFRSF12A	Q9NP84	0.58945296	2.36E-09
NfL	OID00476	Inflammation	CDCP1	Q9H5V8	0.57450459	5.94E-09
NfL	OID01027	Immune	BTN3A2	P78410	0.57521886	6.92E-09
NfL	OID00514	Inflammation	IL-15RA	Q13261	0.5663886	1.08E-08
NfL	OID00320	Neurology	MATN3	O15232	0.56896027	1.09E-08
NfL	OID00522	Inflammation	HGF	P14210	0.55559725	2.33E-08
NfL	OID01009	Immune	AREG	P15514	0.5554737	2.84E-08
NfL	OID00444	Cardiovascular	DCN	P07585	0.55068468	3.27E-08
NfL	OID00355	Neurology	PDGF-R-alpha	P16234	0.54695456	5.07E-08
NfL	OID00409	Cardiovascular	IL18	Q14116	0.54195222	5.92E-08
NfL	OID00501	Inflammation	IL18	Q14116	0.54040689	6.57E-08
NfL	OID00535	Inflammation	CXCL10	P02778	0.53732308	8.05E-08
NfL	OID00381	Cardiovascular	ADM	P35318	0.53654482	8.48E-08
NfL	OID00440	Cardiovascular	CCL3	P10147	0.53638613	8.57E-08
NfL	OID00541	Inflammation	EN-RAGE	P80511	0.53453434	9.67E-08
NfL	OID00333	Neurology	GFR-alpha-1	P56159	0.53516689	1.10E-07
NfL	OID00472	Inflammation	VEGFA	P15692	0.53150904	1.18E-07
NfL	OID00292	Neurology	UNC5C	O95185	0.5317816	1.37E-07
NfL	OID00390	Cardiovascular	IL6	P05231	0.5280754	1.47E-07
NfL	OID00947	Immune	IL6	P05231	0.52860596	1.68E-07
NfL	OID00482	Inflammation	IL6	P05231	0.52347147	1.97E-07
NfL	OID00474	Inflammation	MCP-3	P80098	0.52120099	2.27E-07
NfL	OID05548	Inflammation	TNF	P01375	0.51691619	2.96E-07
NfL	OID00517	Inflammation	IL-18R1	Q13478	0.51449909	3.44E-07
NfL	OID00295	Neurology	CLM-6	Q08708	0.51567642	3.75E-07
NfL	OID00484	Inflammation	MCP-1	P13500	0.51113758	4.22E-07

NfL	OID01018	Immune	DDX58	O95786	0.5132129	4.36E-07
NfL	OID00469	Cardiovascular	PARP-1	P09874	0.50811837	5.06E-07
NfL	OID00965	Immune	LILRB4	Q8NHJ6	0.50837258	5.82E-07
NfL	OID00436	Cardiovascular	CEACAM8	P31997	0.50542795	5.94E-07
NfL	OID00459	Cardiovascular	CTSL1	P07711	0.5022743	7.16E-07
NfL	OID00341	Neurology	DRAXIN	Q8NBI3	0.5015413	8.69E-07
NfL	OID00502	Inflammation	SLAMF1	Q13291	0.49875579	8.81E-07
NfL	OID00562	Inflammation	CSF-1	P09603	0.49799095	9.21E-07
NfL	OID00938	Immune	PSIP1	O75475	0.49931665	9.89E-07
NfL	OID00308	Neurology	CD38	P28907	0.49571279	1.22E-06
NfL	OID00405	Cardiovascular	LOX-1	P78380	0.49313546	1.22E-06
NfL	OID00426	Cardiovascular	KIM1	Q96D42	0.49102441	1.37E-06
NfL	OID00344	Neurology	PVR	P15151	0.49356193	1.37E-06
NfL	OID00369	Neurology	Dkk-4	Q9UBT3	0.49335711	1.39E-06
NfL	OID01007	Immune	NCR1	O76036	0.49295356	1.42E-06
NfL	OID00486	Inflammation	CXCL11	O14625	0.4901258	1.45E-06
NfL	OID00406	Cardiovascular	Gal-9	O00182	0.48624639	1.80E-06
NfL	OID00360	Neurology	CTSS	P25774	0.48760141	1.92E-06
NfL	OID00296	Neurology	EZR	P15311	0.48616741	2.08E-06
NfL	OID00460	Cardiovascular	hOSCAR	Q8IYS5	0.48262666	2.20E-06
NfL	OID00378	Neurology	KYNU	Q16719	0.48365373	2.39E-06
NfL	OID00503	Inflammation	TGF-alpha	P01135	0.47702914	2.99E-06
NfL	OID00506	Inflammation	TNFSF14	O43557	0.47315074	3.69E-06
NfL	OID00364	Neurology	PLXNB1	O43157	0.47530545	3.76E-06
NfL	OID00530	Inflammation	CCL23	P55773	0.47047701	4.26E-06
NfL	OID00972	Immune	EGLN1	Q9GZT9	0.47115429	4.68E-06
NfL	OID00988	Immune	CLEC4D	Q8WXI8	0.46790018	5.56E-06
NfL	OID00464	Cardiovascular	CA5A	P35218	0.4615847	6.80E-06
NfL	OID00387	Cardiovascular	IL-4RA	P24394	0.46158454	6.80E-06
NfL	OID01015	Immune	LAMP3	Q9UQV4	0.46204179	7.53E-06
NfL	OID00315	Neurology	SIGLEC1	Q9BZZ2	0.45959477	8.53E-06
NfL	OID00429	Cardiovascular	VSIG2	Q96IQ7	0.4530089	1.05E-05
NfL	OID00513	Inflammation	CCL19	Q99731	0.4510358	1.16E-05
NfL	OID00328	Neurology	GDF-8	O14793	-0.4533372	1.17E-05

NfL	OID00414	Cardiovascular	CTRC	Q99895	-0.4500775	1.22E-05
NfL	OID00437	Cardiovascular	PTX3	P26022	0.44953044	1.25E-05
NfL	OID00993	Immune	IL10	P22301	0.44803364	1.52E-05
NfL	OID00311	Neurology	Alpha-2-MRAP	P30533	0.44606983	1.67E-05
NfL	OID00527	Inflammation	MMP-10	P09238	0.43629052	2.40E-05
NfL	OID00366	Neurology	CLM-1	Q8TDQ1	0.43782213	2.49E-05
NfL	OID00389	Cardiovascular	IL-1ra	P18510	0.43521126	2.52E-05
NfL	OID00494	Inflammation	OSM	P13725	0.43479864	2.57E-05
NfL	OID00432	Cardiovascular	HO-1	P09601	0.43466349	2.59E-05
NfL	OID00416	Cardiovascular	SPON2	Q9BUD6	0.4335948	2.73E-05
NfL	OID05547	Inflammation	IFN-gamma	P01579	0.43348288	2.74E-05
NfL	OID00412	Cardiovascular	RAGE	Q15109	0.43037792	3.17E-05
NfL	OID00425	Cardiovascular	MERTK	Q12866	0.42898605	3.39E-05
NfL	OID00434	Cardiovascular	IL16	Q14005	0.42787742	3.57E-05
NfL	OID00465	Cardiovascular	HSP 27	P04792	0.42345654	4.38E-05
NfL	OID00455	Cardiovascular	BNP	P16860	0.42334271	4.40E-05
NfL	OID00330	Neurology	WFIKKN1	Q96NZ8	-0.4249502	4.54E-05
NfL	OID00310	Neurology	MSR1	P21757	0.42445804	4.64E-05
NfL	OID01017	Immune	CLEC6A	Q6EIG7	0.42375123	4.79E-05
NfL	OID00402	Cardiovascular	IL-27	Q8NEV9,Q14213	0.42062867	4.98E-05
NfL	OID00995	Immune	KLRD1	Q13241	0.42143165	5.32E-05
NfL	OID00287	Neurology	NMNAT1	Q9HAN9	0.42095917	5.44E-05
NfL	OID00383	Cardiovascular	SLAMF7	Q9NQ25	0.41733888	5.78E-05
NfL	OID00457	Cardiovascular	ACE2	Q9BYF1	0.41712898	5.84E-05
NfL	OID00335	Neurology	Beta-NGF	P01138	0.4171585	6.45E-05
NfL	OID00528	Inflammation	IL10	P22301	0.41365337	6.82E-05
NfL	OID00354	Neurology	IL-5R-alpha	Q01344	0.41068127	8.58E-05
NfL	OID00428	Cardiovascular	TM	P07204	0.40531017	9.85E-05
NfL	OID00515	Inflammation	IL-10RB	Q08334	0.40424865	0.000103
NfL	OID00290	Neurology	CADM3	Q8N126	0.40327663	0.000118
NfL	OID01026	Immune	ITGB6	P18564	0.40227899	0.000123
NfL	OID00318	Neurology	CLEC1B	Q9P126	0.39968428	0.000138
NfL	OID00992	Immune	CXADR	P78310	0.39921963	0.00014
NfL	OID00418	Cardiovascular	FS	P19883	0.39681183	0.000142

NfL	OID00556	Inflammation	CCL20	P78556	0.39572198	0.000148
NfL	OID01016	Immune	CLEC7A	Q9BXN2	0.39469684	0.00017
NfL	OID00447	Cardiovascular	PRSS8	Q16651	0.38888171	0.000197
NfL	OID00499	Inflammation	CD6	P30203	-0.3869738	0.000213
NfL	OID00973	Immune	NFATC3	Q12968	0.38434409	0.000259
NfL	OID00549	Inflammation	MCP-2	P80075	0.3820997	0.00026
NfL	OID00309	Neurology	SMPD1	P17405	0.38387533	0.000264
NfL	OID00957	Immune	TRIM5	Q9C035	0.38349108	0.000268
NfL	OID00367	Neurology	SPOCK1	Q08629	0.38255766	0.000278
NfL	OID00511	Inflammation	LIF-R	P42702	0.3767333	0.000322
NfL	OID00498	Inflammation	CCL4	P13236	0.37037333	0.000414
NfL	OID00524	Inflammation	IL-24	Q13007	0.37036961	0.000414
NfL	OID00461	Cardiovascular	TNFRSF13B	O14836	0.36860958	0.000443
NfL	OID00431	Cardiovascular	PRELP	P51888	0.36716977	0.000468
NfL	OID00433	Cardiovascular	XCL1	P47992	0.36699694	0.000471
NfL	OID00408	Cardiovascular	SCF	P21583	-0.3595716	0.000625
NfL	OID00945	Immune	IRF9	Q00978	0.36075671	0.000644
NfL	OID00971	Immune	MILR1	Q7Z6M3	0.36069005	0.000646
NfL	OID00507	Inflammation	FGF-23	Q9GZV9	0.35682483	0.000693
NfL	OID00547	Inflammation	LIF	P15018	0.35626418	0.000707
NfL	OID00996	Immune	BACH1	O14867	0.35807122	0.000712
NfL	OID00483	Inflammation	IL-17C	Q9P0M4	0.35454673	0.000754
NfL	OID00944	Immune	CLEC4G	Q6UXB4	0.35591206	0.000771
NfL	OID00500	Inflammation	SCF	P21583	-0.3515327	0.000842
NfL	OID00352	Neurology	FcRL2	Q96LA5	0.35323447	0.00085
NfL	OID00288	Neurology	NRP2	O60462	0.35273833	0.000865
NfL	OID00385	Cardiovascular	ADAM-TS13	Q76LX8	-0.3453887	0.001051
NfL	OID00415	Cardiovascular	FGF-23	Q9GZV9	0.34312976	0.00114
NfL	OID00466	Cardiovascular	CD4	P01730	0.33988919	0.001278
NfL	OID00423	Cardiovascular	REN	P00797	0.33947137	0.001297
NfL	OID00964	Immune	TRIM21	P19474	0.33833437	0.001442
NfL	OID00359	Neurology	JAM-B	P57087	0.3361055	0.001557
NfL	OID00481	Inflammation	uPA	P00749	0.33293869	0.001627
NfL	OID00356	Neurology	CTSC	P53634	0.33382827	0.001683

NfL	OID01025	Immune	CD83	Q01151	0.33189939	0.001797
NfL	OID00445	Cardiovascular	Dkk-1	O94907	0.32972854	0.001816
NfL	OID00550	Inflammation	CASP-8	Q14790	0.32600505	0.00206
NfL	OID00509	Inflammation	FGF-5	P12034	0.3248316	0.002143
NfL	OID01019	Immune	IL12RB1	P42701	0.32563099	0.002218
NfL	OID00458	Cardiovascular	PD-L2	Q9BQ51	0.32285653	0.002289
NfL	OID01023	Immune	LAG3	P18627	0.32430411	0.002317
NfL	OID01004	Immune	DFFA	O00273	0.3224628	0.002462
NfL	OID00491	Inflammation	CST5	P28325	0.32028147	0.002492
NfL	OID00955	Immune	PRDX5	P30044	0.31918443	0.00274
NfL	OID00411	Cardiovascular	PIgR	P01833	0.3165808	0.002814
NfL	OID00949	Immune	CLEC4C	Q8WTT0	-0.3170991	0.002932
NfL	OID00987	Immune	HEXIM1	O94992	0.31376485	0.003263
NfL	OID00307	Neurology	CPA2	P48052	-0.3088927	0.003807
NfL	OID00961	Immune	GALNT3	Q14435	0.30620641	0.00414
NfL	OID01012	Immune	PADI2	Q9Y2J8	0.3053103	0.004257
NfL	OID00410	Cardiovascular	FGF-21	Q9NSA1	0.30312723	0.004318
NfL	OID00313	Neurology	EPHB6	O15197	0.30439272	0.004379
NfL	OID00560	Inflammation	ADA	P00813	0.29982047	0.004783
NfL	OID00553	Inflammation	TNFRSF9	Q07011	0.29398078	0.005714
NfL	OID01008	Immune	CXCL12	P48061	0.29417179	0.005974
NfL	OID00324	Neurology	gal-8	O00214	0.29201302	0.00637
NfL	OID00488	Inflammation	TRAIL	P50591	-0.2891121	0.006609
NfL	OID00419	Cardiovascular	GLO1	Q04760	0.28895806	0.00664
NfL	OID01010	Immune	IFNLR1	Q8IU57	0.28914314	0.006932
NfL	OID00512	Inflammation	FGF-21	Q9NSA1	0.28510647	0.007437
NfL	OID01213	Inflammation	DNER	Q8NFT8	-0.2839863	0.007684
NfL	OID00523	Inflammation	IL-12B	P29460	0.28222445	0.008087
NfL	OID00536	Inflammation	4E-BP1	Q13541	0.28185529	0.008174
NfL	OID00496	Inflammation	CXCL1	P09341	0.2808169	0.008423
NfL	OID00368	Neurology	IL12	P29460,P29459	0.28228509	0.008456
NfL	OID00448	Cardiovascular	AGRP	O00253	0.28049802	0.0085
NfL	OID00351	Neurology	BMP-4	P12644	-0.2817185	0.008595
NfL	OID00939	Immune	ZBTB16	Q05516	0.28079522	0.008824

NfL	OID00404	Cardiovascular	CXCL1	P09341	0.27619852	0.009611
NfL	OID00555	Inflammation	TWEAK	O43508	-0.2745142	0.010079
NfL	OID00422	Cardiovascular	SERPINA12	Q8IW75	-0.2727056	0.010604
NfL	OID00435	Cardiovascular	SORT1	Q99523	0.27127993	0.011035
NfL	OID00329	Neurology	THY 1	P04216	0.27229371	0.011203
NfL	OID00951	Immune	CLEC4A	Q9UMR7	-0.2722424	0.011218
NfL	OID00305	Neurology	RGMA	Q96B86	-0.2701957	0.011869
NfL	OID00976	Immune	EIF4G1	Q04637	0.2691853	0.012202
NfL	OID00338	Neurology	NTRK2	Q16620	-0.2688252	0.012323
NfL	OID00950	Immune	IRAK1	P51617	0.26781155	0.012668
NfL	OID00478	Inflammation	IL7	P13232	0.26528443	0.013018
NfL	OID00312	Neurology	sFRP-3	Q92765	0.26444695	0.013876
NfL	OID00485	Inflammation	IL-17A	Q16552	0.262642	0.013985
NfL	OID01005	Immune	DCBLD2	Q96PD2	0.26109453	0.015177
NfL	OID00342	Neurology	SCARF2	Q96GP6	0.26103237	0.015202
NfL	OID00942	Immune	HCLS1	P14317	0.25940811	0.01587
NfL	OID00386	Cardiovascular	BOC	Q9BWV1	-0.2539846	0.017605
NfL	OID00353	Neurology	MDGA1	Q8NFP4	-0.2544278	0.01808
NfL	OID00952	Immune	PRDX1	Q06830	0.25419838	0.018187
NfL	OID00340	Neurology	G-CSF	P09919	0.25408161	0.018243
NfL	OID00376	Neurology	CD200R1	Q8TD46	-0.2529613	0.018779
NfL	OID00561	Inflammation	TNFB	P01374	-0.2514645	0.0188
NfL	OID00452	Cardiovascular	THPO	P40225	0.25067354	0.019189
NfL	OID01021	Immune	ITGA11	Q9UKX5	-0.2513635	0.019567
NfL	OID00373	Neurology	LAIR-2	Q6ISS4	0.25115171	0.019673
NfL	OID00397	Cardiovascular	PRSS27	Q9BQR3	-0.2494689	0.019795
NfL	OID00316	Neurology	CNTN5	O94779	-0.2508531	0.019824
NfL	OID00293	Neurology	VWC2	Q2TAL6	0.25024595	0.020134
NfL	OID00467	Cardiovascular	NEMO	Q9Y6K9	0.24673666	0.02123
NfL	OID00441	Cardiovascular	MMP7	P09237	0.2459027	0.021686
NfL	OID00941	Immune	TPSAB1	Q15661	0.2446511	0.023194
NfL	OID00339	Neurology	GZMA	P12544	0.24287119	0.024247
NfL	OID01000	Immune	ARNT	P27540	0.23926628	0.026505
NfL	OID00493	Inflammation	IL-1 alpha	P01583	-0.2360918	0.027701

NfL	OID00314	Neurology	RGMB	Q6NW40	0.23519429	0.029268
NfL	OID00303	Neurology	ROBO2	Q9HCK4	-0.2350925	0.02934
NfL	OID00442	Cardiovascular	IgG Fc receptor II-b	P31994	0.23206326	0.030552
NfL	OID00438	Cardiovascular	PSGL-1	Q14242	-0.2292917	0.032654
NfL	OID00388	Cardiovascular	SRC	P12931	0.22421884	0.036817
NfL	OID00510	Inflammation	MMP-1	P03956	0.22321739	0.037689
NfL	OID00480	Inflammation	LAP TGF-beta-1	P01137	0.22225127	0.038547
NfL	OID00994	Immune	SRPK2	P78362	0.22267653	0.039327
NfL	OID00374	Neurology	MANF	P55145	0.21991314	0.041898
NfL	OID00348	Neurology	CPM	P14384	-0.219067	0.042713
NfL	OID00332	Neurology	CDH3	P22223	0.21800276	0.043757
NfL	OID00538	Inflammation	SIRT2	Q8IXJ6	0.2155009	0.045005
NfL	OID00297	Neurology	SMOC2	Q9H3U7	0.2152985	0.046504
NfL	OID00450	Cardiovascular	GDF-2	Q9UK05	-0.2093926	0.051598
NfL	OID00953	Immune	PRDX3	P30048	0.20992357	0.052387
NfL	OID01014	Immune	MASP1	P48740	-0.2075858	0.05513
NfL	OID00371	Neurology	LAT	O43561	0.20730903	0.055463
NfL	OID00403	Cardiovascular	IL-17D	Q8TAD2	0.20516784	0.056607
NfL	OID00358	Neurology	DDR1	Q08345	0.20310249	0.060719
NfL	OID00294	Neurology	Siglec-9	Q9Y336	0.20251151	0.061489
NfL	OID00304	Neurology	CRTAM	O95727	0.20246796	0.061547
NfL	OID00962	Immune	FXYD5	Q96DB9	0.20201378	0.062144
NfL	OID00470	Cardiovascular	HAOX1	Q9UJM8	0.20052807	0.062559
NfL	OID00427	Cardiovascular	THBS2	P35442	0.19994913	0.063336
NfL	OID00430	Cardiovascular	AMBP	P02760	0.19099723	0.07638
NfL	OID00551	Inflammation	CCL25	O15444	0.18912936	0.079356
NfL	OID00424	Cardiovascular	DECR1	Q16698	0.18887185	0.079773
NfL	OID00990	Immune	MGMT	P16455	0.18819027	0.082704
NfL	OID00413	Cardiovascular	SOD2	P04179	0.18104835	0.093312
NfL	OID00937	Immune	GLB1	P16278	0.18032081	0.096632
NfL	OID00365	Neurology	TNFRSF21	O75509	0.17395449	0.109195
NfL	OID00350	Neurology	GCP5	P78333	-0.1726377	0.111946
NfL	OID00421	Cardiovascular	PAPPA	Q13219	0.170805	0.113703
NfL	OID00558	Inflammation	STAMBP	O95630	0.1696788	0.11614

NfL	OID00505	Inflammation	CCL11	P51671	0.16929489	0.11698
NfL	OID00958	Immune	DCTN1	Q14203	0.1658896	0.12689
NfL	OID00526	Inflammation	ARTN	Q5T4W7	0.16464308	0.127535
NfL	OID00963	Immune	TRAF2	Q12933	0.16457691	0.129966
NfL	OID00417	Cardiovascular	GH	P01241	0.1620967	0.133612
NfL	OID00400	Cardiovascular	IL1RL2	Q9HB29	0.16139996	0.135313
NfL	OID00984	Immune	FCRL3	Q96P31	0.16198307	0.136212
NfL	OID00997	Immune	PIK3AP1	Q6ZUJ8	0.16185849	0.136517
NfL	OID00960	Immune	CDSN	Q15517	0.16073627	0.139294
NfL	OID01020	Immune	TANK	Q92844	0.16044653	0.140017
NfL	OID00462	Cardiovascular	TGM2	P21980	0.15753518	0.145046
NfL	OID00393	Cardiovascular	IDUA	P35475	-0.1566831	0.147261
NfL	OID00392	Cardiovascular	STK4	Q13043	0.15654107	0.147633
NfL	OID00401	Cardiovascular	PDGF subunit B	P01127	0.15593367	0.149231
NfL	OID00533	Inflammation	Flt3L	P49771	0.15574794	0.149722
NfL	OID00936	Immune	PPP1R9B	Q96SB3	0.15596414	0.151578
NfL	OID00534	Inflammation	CXCL6	P80162	0.15347116	0.15584
NfL	OID00336	Neurology	SCARA5	Q6ZMJ2	0.15434398	0.155928
NfL	OID00298	Neurology	NBL1	P41271	0.15290213	0.159877
NfL	OID00325	Neurology	BCAN	Q96GW7	-0.1514326	0.163977
NfL	OID00347	Neurology	FLRT2	O43155	0.15132184	0.164289
NfL	OID00557	Inflammation	ST1A1	P50225	0.14921765	0.167767
NfL	OID00979	Immune	BIRC2	Q13490	0.14955859	0.169318
NfL	OID00954	Immune	FGF2	P09038	0.14894538	0.171093
NfL	OID00970	Immune	CCL11	P51671	0.14855883	0.17222
NfL	OID01003	Immune	ICA1	Q05084	0.14840819	0.17266
NfL	OID00554	Inflammation	NT-3	P20783	-0.1459458	0.177389
NfL	OID00475	Inflammation	GDNF	P39905	0.14148686	0.191145
NfL	OID00982	Immune	PLXNA4	Q9HCM2	0.14181638	0.192745
NfL	OID00331	Neurology	TMPRSS5	Q9H3S3	-0.1399342	0.198778
NfL	OID00983	Immune	SH2B3	Q9UQQ2	0.13892974	0.202053
NfL	OID00443	Cardiovascular	ITGB1BP2	Q9UKP3	0.13809628	0.202108
NfL	OID00372	Neurology	NTRK3	Q16288	-0.1379307	0.205348
NfL	OID00968	Immune	ITM2A	O43736	0.13781889	0.205719

NfL	OID00379	Cardiovascular	BMP-6	P22004	0.13426122	0.215044
NfL	OID00943	Immune	CNTNAP2	Q9UHC6	0.13449787	0.216961
NfL	OID05124	Inflammation	CD8A	P01732	-0.1315519	0.224529
NfL	OID00948	Immune	DGKZ	Q13574	0.12995965	0.233016
NfL	OID00539	Inflammation	CCL28	Q9NRJ3	0.12885286	0.234266
NfL	OID00487	Inflammation	AXIN1	O15169	0.12680405	0.241851
NfL	OID01022	Immune	KPNA1	P52294	0.12667179	0.245152
NfL	OID00395	Cardiovascular	PAR-1	P25116	0.12179258	0.261113
NfL	OID00468	Cardiovascular	VEGFD	O43915	-0.1120382	0.301527
NfL	OID00453	Cardiovascular	MARCO	Q9UEW3	0.11045665	0.308445
NfL	OID01002	Immune	SH2D1A	O60880	0.10981263	0.314172
NfL	OID00306	Neurology	PLXNB3	Q9ULL4	0.10877835	0.318779
NfL	OID00940	Immune	IRAK4	Q9NWZ3	0.1068814	0.327342
NfL	OID00999	Immune	STC1	P52823	0.09553818	0.381561
NfL	OID00998	Immune	SPRY2	O43597	0.09471706	0.385685
NfL	OID01001	Immune	FAM3B	P58499	0.09335997	0.392559
NfL	OID00317	Neurology	ADAM 22	Q9P0K1	0.0924412	0.397255
NfL	OID01011	Immune	DAPP1	Q9UN19	0.09165046	0.401322
NfL	OID00337	Neurology	CD200	P41217	0.09150327	0.402082
NfL	OID00504	Inflammation	MCP-4	Q99616	0.09062065	0.40386
NfL	OID00956	Immune	DPP10	Q8N608	0.09085063	0.405461
NfL	OID00446	Cardiovascular	LPL	P06858	-0.0900969	0.406594
NfL	OID00301	Neurology	NCAN	O14594	-0.0904569	0.407508
NfL	OID00334	Neurology	GM-CSF-R-alpha	P15509	0.08848702	0.417838
NfL	OID00375	Neurology	TN-R	Q92752	-0.0884451	0.418059
NfL	OID00407	Cardiovascular	GIF	P27352	0.0867909	0.424098
NfL	OID01006	Immune	FCRL6	Q6DN72	0.08613088	0.43039
NfL	OID00377	Neurology	Nr-CAM	Q92823	0.08524981	0.435139
NfL	OID00508	Inflammation	IL-10RA	Q13651	-0.0836677	0.441024
NfL	OID00977	Immune	CD28	P10747	-0.0837399	0.443346
NfL	OID00477	Inflammation	CD244	Q9BZW8	-0.0779595	0.472921
NfL	OID00302	Neurology	PRTG	Q2VWP7	-0.0764125	0.48438
NfL	OID00420	Cardiovascular	CD84	Q9UIB8	0.07371913	0.497401
NfL	OID00989	Immune	PRKCQ	Q04759	0.07305778	0.503821

NfL	OID00322	Neurology	HAGH	Q16775	0.06400796	0.558211
NfL	OID00981	Immune	NF2	P35240	0.06345443	0.561627
NfL	OID00343	Neurology	GDNFR-alpha-3	O60609	-0.0614916	0.573818
NfL	OID00978	Immune	PTH1R	Q03431	-0.0573249	0.600096
NfL	OID00382	Cardiovascular	CD40-L	P29965	0.0553963	0.610317
NfL	OID00456	Cardiovascular	MMP12	P39900	0.05444799	0.616453
NfL	OID00520	Inflammation	CXCL5	P42830	0.05359663	0.621984
NfL	OID01024	Immune	IL5	P05113	0.05281354	0.629136
NfL	OID00974	Immune	LY75	O60449	-0.0524133	0.631741
NfL	OID00454	Cardiovascular	GT	P51161	0.04639243	0.669604
NfL	OID00439	Cardiovascular	CCL17	Q92583	-0.0459951	0.672271
NfL	OID00349	Neurology	CLEC10A	Q8IUN9	-0.0451437	0.679805
NfL	OID00398	Cardiovascular	TIE2	Q02763	0.04379366	0.687121
NfL	OID00489	Inflammation	IL-20RA	Q9UHF4	0.04303685	0.692254
NfL	OID00980	Immune	HSD11B1	P28845	-0.0426989	0.696272
NfL	OID00323	Neurology	LXN	Q9BS40	0.0410753	0.707288
NfL	OID00357	Neurology	CDH6	P55285	-0.040519	0.711076
NfL	OID00361	Neurology	N-CDase	Q9NR71	-0.0395599	0.717624
NfL	OID00380	Cardiovascular	ANGPT1	Q15389	0.03892255	0.720396
NfL	OID00451	Cardiovascular	FABP2	P12104	-0.0389132	0.72046
NfL	OID00362	Neurology	NAAA	Q02083	-0.0338628	0.756923
NfL	OID00291	Neurology	GDNF	P39905	0.02943016	0.787938
NfL	OID00531	Inflammation	CD5	P06127	-0.029185	0.788438
NfL	OID00319	Neurology	ADAM 23	O75077	0.02909267	0.790314
NfL	OID00545	Inflammation	FGF-19	O95750	0.0274284	0.8009
NfL	OID00959	Immune	ITGA6	P23229	-0.0236274	0.829038
NfL	OID00327	Neurology	NEP	P08473	0.02266273	0.835919
NfL	OID00463	Cardiovascular	LEP	P41159	-0.0178826	0.869418
NfL	OID00449	Cardiovascular	HB-EGF	Q99075	0.01604569	0.88273
NfL	OID01013	Immune	SIT1	Q9Y3P8	-0.0142322	0.89652
NfL	OID05024	Neurology	MAPT	P10636	0.00949941	0.930825
NfL	OID00946	Immune	EDAR	Q9UNE0	-0.0091153	0.933615
NfL	OID00966	Immune	NTF4	P34130	-0.0091019	0.933713
Tau	OID00380	Cardiovascular	ANGPT1	Q15389	-0.5989159	8.92E-10

Tau	OID00401	Cardiovascular	PDGF subunit B	P01127	-0.4609265	7.03E-06
Tau	OID00382	Cardiovascular	CD40-L	P29965	-0.424519	4.17E-05
Tau	OID00449	Cardiovascular	HB-EGF	Q99075	-0.4218897	4.70E-05
Tau	OID00439	Cardiovascular	CCL17	Q92583	-0.4210625	4.88E-05
Tau	OID00300	Neurology	SCARB2	Q14108	0.39401636	0.000174
Tau	OID00510	Inflammation	MMP-1	P03956	-0.3800688	0.000282
Tau	OID00340	Neurology	G-CSF	P09919	0.37317456	0.000402
Tau	OID00410	Cardiovascular	FGF-21	Q9NSA1	0.36960077	0.000426
Tau	OID00520	Inflammation	CXCL5	P42830	-0.3657946	0.000494
Tau	OID00985	Immune	CKAP4	Q07065	0.36552372	0.000539
Tau	OID00512	Inflammation	FGF-21	Q9NSA1	0.36275469	0.000554
Tau	OID00445	Cardiovascular	Dkk-1	O94907	-0.3608234	0.000596
Tau	OID00327	Neurology	NEP	P08473	-0.3623049	0.000608
Tau	OID00555	Inflammation	TWEAK	O43508	-0.3545604	0.000753
Tau	OID00341	Neurology	DRAXIN	Q8NBI3	0.35320087	0.000851
Tau	OID00480	Inflammation	LAP TGF-beta-1	P01137	-0.342353	0.001171
Tau	OID00350	Neurology	GCP5	P78333	-0.3418926	0.001274
Tau	OID00556	Inflammation	CCL20	P78556	0.33698669	0.001415
Tau	OID00399	Cardiovascular	TF	P13726	0.32791398	0.001932
Tau	OID00504	Inflammation	MCP-4	Q99616	-0.3263331	0.002037
Tau	OID00514	Inflammation	IL-15RA	Q13261	0.32375382	0.002221
Tau	OID01023	Immune	LAG3	P18627	0.32556191	0.002223
Tau	OID00993	Immune	IL10	P22301	0.3140696	0.003231
Tau	OID00483	Inflammation	IL-17C	Q9P0M4	0.31121375	0.003346
Tau	OID00444	Cardiovascular	DCN	P07585	0.30867201	0.003628
Tau	OID00363	Neurology	N2DL-2	Q9BZM5	0.30867846	0.003832
Tau	OID00513	Inflammation	CCL19	Q99731	0.3059953	0.003947
Tau	OID00528	Inflammation	IL10	P22301	0.30301123	0.004333
Tau	OID00415	Cardiovascular	FGF-23	Q9GZV9	0.30091278	0.004625
Tau	OID00947	Immune	IL6	P05231	0.30140877	0.0048
Tau	OID00323	Neurology	LXN	Q9BS40	-0.2984675	0.00525
Tau	OID00478	Inflammation	IL7	P13232	-0.2955495	0.005449
Tau	OID01006	Immune	FCRL6	Q6DN72	0.29480104	0.005862
Tau	OID00326	Neurology	LAYN	Q6UX15	0.29437037	0.005938

Tau	OID05547	Inflammation	IFN-gamma	P01579	0.29118777	0.006213
Tau	OID00967	Immune	KRT19	P08727	0.28976769	0.006806
Tau	OID00518	Inflammation	PD-L1	Q9NZQ7	0.28783069	0.006865
Tau	OID00414	Cardiovascular	CTRC	Q99895	-0.2869823	0.007039
Tau	OID00400	Cardiovascular	IL1RL2	Q9HB29	0.28653883	0.007131
Tau	OID00507	Inflammation	FGF-23	Q9GZV9	0.28521454	0.007413
Tau	OID00429	Cardiovascular	VSIG2	Q96IQ7	0.28287022	0.007937
Tau	OID01018	Immune	DDX58	O95786	0.28032509	0.008943
Tau	OID00482	Inflammation	IL6	P05231	0.2768133	0.009445
Tau	OID00484	Inflammation	MCP-1	P13500	0.27561585	0.009771
Tau	OID00390	Cardiovascular	IL6	P05231	0.27435391	0.010125
Tau	OID00547	Inflammation	LIF	P15018	0.27200231	0.010815
Tau	OID00309	Neurology	SMPD1	P17405	0.27354817	0.01082
Tau	OID00352	Neurology	FcRL2	Q96LA5	0.27313876	0.010943
Tau	OID01009	Immune	AREG	P15514	0.26980126	0.011998
Tau	OID00423	Cardiovascular	REN	P00797	0.26593728	0.012788
Tau	OID00355	Neurology	PDGF-R-alpha	P16234	0.26534236	0.013545
Tau	OID00552	Inflammation	CX3CL1	P78423	0.26127199	0.014511
Tau	OID00412	Cardiovascular	RAGE	Q15109	0.25553267	0.016904
Tau	OID00299	Neurology	EFNA4	P52798	0.25696552	0.016923
Tau	OID00969	Immune	HNMT	P50135	0.25547496	0.017594
Tau	OID00965	Immune	LILRB4	Q8NHJ6	0.25388278	0.018337
Tau	OID00961	Immune	GALNT3	Q14435	0.25195303	0.019273
Tau	OID00479	Inflammation	OPG	O00300	0.249505	0.019777
Tau	OID00345	Neurology	TNFRSF12A	Q9NP84	0.24984954	0.020339
Tau	OID00490	Inflammation	CXCL9	Q07325	0.24737212	0.020889
Tau	OID00485	Inflammation	IL-17A	Q16552	0.24660512	0.021302
Tau	OID05024	Neurology	MAPT	P10636	-0.2477268	0.021467
Tau	OID00371	Neurology	LAT	O43561	-0.2458936	0.022483
Tau	OID01027	Immune	BTN3A2	P78410	0.24565842	0.022616
Tau	OID01019	Immune	IL12RB1	P42701	0.24109625	0.025338
Tau	OID00287	Neurology	NMNAT1	Q9HAN9	0.23720362	0.027876
Tau	OID00517	Inflammation	IL-18R1	Q13478	0.235484	0.028117
Tau	OID00418	Cardiovascular	FS	P19883	0.23493701	0.028495

Tau	OID00402	Cardiovascular	IL-27	Q8NEV9,Q14213	0.23386462	0.029248
Tau	OID00396	Cardiovascular	TRAIL-R2	O14763	0.23349681	0.029511
Tau	OID00534	Inflammation	CXCL6	P80162	-0.2331812	0.029738
Tau	OID00511	Inflammation	LIF-R	P42702	0.23183979	0.030718
Tau	OID00331	Neurology	TMPRSS5	Q9H3S3	-0.2331148	0.03077
Tau	OID00535	Inflammation	CXCL10	P02778	0.2314488	0.031008
Tau	OID00435	Cardiovascular	SORT1	Q99523	-0.2313177	0.031106
Tau	OID00437	Cardiovascular	PTX3	P26022	0.23082729	0.031475
Tau	OID00420	Cardiovascular	CD84	Q9UIB8	-0.2304012	0.031799
Tau	OID00391	Cardiovascular	TNFRSF10A	O00220	0.23008988	0.032037
Tau	OID00389	Cardiovascular	IL-1ra	P18510	0.23004686	0.03207
Tau	OID00417	Cardiovascular	GH	P01241	-0.2280003	0.033675
Tau	OID00387	Cardiovascular	IL-4RA	P24394	0.22715703	0.034355
Tau	OID00521	Inflammation	TRANCE	O14788	-0.2259456	0.035353
Tau	OID00545	Inflammation	FGF-19	O95750	0.22366763	0.037295
Tau	OID00318	Neurology	CLEC1B	Q9P126	-0.2244306	0.037764
Tau	OID00407	Cardiovascular	GIF	P27352	0.22282396	0.038037
Tau	OID00308	Neurology	CD38	P28907	0.2236126	0.038486
Tau	OID00434	Cardiovascular	IL16	Q14005	0.2213847	0.03933
Tau	OID00383	Cardiovascular	SLAMF7	Q9NQ25	0.21704001	0.043459
Tau	OID00466	Cardiovascular	CD4	P01730	0.216297	0.0442
Tau	OID00381	Cardiovascular	ADM	P35318	0.2160103	0.044488
Tau	OID00406	Cardiovascular	Gal-9	O00182	0.21526088	0.04525
Tau	OID00995	Immune	KLRD1	Q13241	0.21409247	0.047774
Tau	OID00448	Cardiovascular	AGRP	O00253	0.21214954	0.04853
Tau	OID01000	Immune	ARNT	P27540	0.21276366	0.049206
Tau	OID00315	Neurology	SIGLEC1	Q9BZZ2	0.21258463	0.049402
Tau	OID00503	Inflammation	TGF-alpha	P01135	0.21131455	0.049443
Tau	OID00428	Cardiovascular	TM	P07204	0.20818737	0.052988
Tau	OID00384	Cardiovascular	PGF	P49763	0.20774925	0.053501
Tau	OID00296	Neurology	EZR	P15311	0.20204474	0.062103
Tau	OID00474	Inflammation	MCP-3	P80098	0.20022517	0.062965
Tau	OID00346	Neurology	SKR3	P37023	0.20073304	0.063855
Tau	OID00524	Inflammation	IL-24	Q13007	0.1991982	0.064356

Tau	OID00422	Cardiovascular	SERPINA12	Q8IW75	-0.1985072	0.065306
Tau	OID05548	Inflammation	TNF	P01375	0.19786916	0.066193
Tau	OID00404	Cardiovascular	CXCL1	P09341	-0.1965511	0.068057
Tau	OID01008	Immune	CXCL12	P48061	-0.1953195	0.071512
Tau	OID00353	Neurology	MDGA1	Q8NFP4	-0.194724	0.072397
Tau	OID00290	Neurology	CADM3	Q8N126	0.19469468	0.072441
Tau	OID01015	Immune	LAMP3	Q9UQV4	0.19408388	0.073359
Tau	OID00367	Neurology	SPOCK1	Q08629	0.19386295	0.073693
Tau	OID00320	Neurology	MATN3	O15232	0.19338009	0.074428
Tau	OID05124	Inflammation	CD8A	P01732	0.19068867	0.076865
Tau	OID00459	Cardiovascular	CTSL1	P07711	0.18967398	0.078479
Tau	OID00971	Immune	MILR1	Q7Z6M3	0.18954729	0.080473
Tau	OID00295	Neurology	CLM-6	Q08708	0.18915596	0.081111
Tau	OID00394	Cardiovascular	TNFRSF11A	Q9Y6Q6	0.18648199	0.083731
Tau	OID00999	Immune	STC1	P52823	0.18675621	0.085116
Tau	OID00496	Inflammation	CXCL1	P09341	-0.1845074	0.087118
Tau	OID00549	Inflammation	MCP-2	P80075	0.182063	0.09146
Tau	OID00988	Immune	CLEC4D	Q8WXI8	0.18062022	0.09607
Tau	OID00397	Cardiovascular	PRSS27	Q9BQR3	-0.1792367	0.096691
Tau	OID01016	Immune	CLEC7A	Q9BXN2	0.17952413	0.098139
Tau	OID00330	Neurology	WFIKKN1	Q96NZ8	-0.1791942	0.098769
Tau	OID00468	Cardiovascular	VEGFD	O43915	-0.1776856	0.099661
Tau	OID01017	Immune	CLEC6A	Q6EIG7	0.17705528	0.102926
Tau	OID00469	Cardiovascular	PARP-1	P09874	0.17547297	0.10402
Tau	OID00348	Neurology	CPM	P14384	-0.1753219	0.106395
Tau	OID00532	Inflammation	CCL3	P10147	0.1725314	0.110043
Tau	OID00431	Cardiovascular	PRELP	P51888	0.17144307	0.112339
Tau	OID00980	Immune	HSD11B1	P28845	0.1720977	0.113089
Tau	OID00411	Cardiovascular	PIgR	P01833	0.17046017	0.114445
Tau	OID00291	Neurology	GDNF	P39905	-0.1713551	0.114676
Tau	OID00471	Inflammation	IL8	P10145	0.16945498	0.116629
Tau	OID00562	Inflammation	CSF-1	P09603	0.16870901	0.118271
Tau	OID00334	Neurology	GM-CSF-R-alpha	P15509	-0.1685144	0.120905
Tau	OID00539	Inflammation	CCL28	Q9NRJ3	-0.1649604	0.126792

Tau	OID00938	Immune	PSIP1	O75475	0.16469854	0.129679
Tau	OID00332	Neurology	CDH3	P22223	0.16432916	0.130553
Tau	OID00306	Neurology	PLXNB3	Q9ULL4	-0.1634065	0.132757
Tau	OID00338	Neurology	NTRK2	Q16620	-0.1631955	0.133265
Tau	OID00959	Immune	ITGA6	P23229	-0.1619135	0.136382
Tau	OID00526	Inflammation	ARTN	Q5T4W7	0.16066466	0.137126
Tau	OID00360	Neurology	CTSS	P25774	0.16083453	0.139049
Tau	OID01025	Immune	CD83	Q01151	0.16023581	0.140546
Tau	OID01005	Immune	DCBLD2	Q96PD2	0.15924378	0.143052
Tau	OID01213	Inflammation	DNER	Q8NFT8	-0.1582232	0.143276
Tau	OID01007	Immune	NCR1	O76036	0.15907914	0.143471
Tau	OID00523	Inflammation	IL-12B	P29460	0.15591306	0.149285
Tau	OID01014	Immune	MASP1	P48740	-0.1564828	0.150205
Tau	OID00464	Cardiovascular	CA5A	P35218	0.15511258	0.151411
Tau	OID00944	Immune	CLEC4G	Q6UXB4	0.15451592	0.155462
Tau	OID00293	Neurology	VWC2	Q2TAL6	-0.151826	0.162872
Tau	OID00322	Neurology	HAGH	Q16775	-0.1507156	0.166006
Tau	OID00328	Neurology	GDF-8	O14793	-0.1500088	0.168024
Tau	OID00508	Inflammation	IL-10RA	Q13651	-0.1477273	0.172101
Tau	OID00419	Cardiovascular	GLO1	Q04760	-0.1474113	0.173031
Tau	OID01026	Immune	ITGB6	P18564	0.14797844	0.173921
Tau	OID00349	Neurology	CLEC10A	Q8IUN9	-0.1473837	0.175676
Tau	OID00386	Cardiovascular	BOC	Q9BWV1	-0.1446651	0.181264
Tau	OID00344	Neurology	PVR	P15151	0.14551113	0.181289
Tau	OID00430	Cardiovascular	AMBP	P02760	0.14379644	0.183927
Tau	OID00465	Cardiovascular	HSP 27	P04792	0.14372067	0.18416
Tau	OID00409	Cardiovascular	IL18	Q14116	0.14369719	0.184233
Tau	OID00501	Inflammation	IL18	Q14116	0.14158802	0.190824
Tau	OID01001	Immune	FAM3B	P58499	0.14238418	0.190952
Tau	OID00960	Immune	CDSN	Q15517	0.14207824	0.191917
Tau	OID00329	Neurology	THY 1	P04216	0.14201434	0.192119
Tau	OID00307	Neurology	CPA2	P48052	-0.1414811	0.19381
Tau	OID00530	Inflammation	CCL23	P55773	0.14040681	0.194589
Tau	OID00481	Inflammation	uPA	P00749	0.13985012	0.196382

Tau	OID00385	Cardiovascular	ADAM-TS13	Q76LX8	-0.1393349	0.198052
Tau	OID00998	Immune	SPRY2	O43597	-0.1387521	0.202636
Tau	OID00379	Cardiovascular	BMP-6	P22004	-0.1373076	0.204722
Tau	OID00454	Cardiovascular	GT	P51161	0.13661935	0.207023
Tau	OID00527	Inflammation	MMP-10	P09238	0.13474418	0.213384
Tau	OID00554	Inflammation	NT-3	P20783	-0.1340007	0.215943
Tau	OID00376	Neurology	CD200R1	Q8TD46	-0.1341864	0.218037
Tau	OID00432	Cardiovascular	HO-1	P09601	0.13187759	0.223373
Tau	OID01003	Immune	ICA1	Q05084	-0.1314179	0.227769
Tau	OID00370	Neurology	EDA2R	Q9HAV5	0.12766818	0.241429
Tau	OID00441	Cardiovascular	MMP7	P09237	0.12481951	0.249358
Tau	OID01013	Immune	SIT1	Q9Y3P8	-0.1251379	0.250961
Tau	OID00436	Cardiovascular	CEACAM8	P31997	0.12410532	0.252098
Tau	OID00476	Inflammation	CDCP1	Q9H5V8	0.1235175	0.254369
Tau	OID00957	Immune	TRIM5	Q9C035	0.12399954	0.255332
Tau	OID00443	Cardiovascular	ITGB1BP2	Q9UKP3	-0.1223582	0.258888
Tau	OID00427	Cardiovascular	THBS2	P35442	0.12134587	0.262879
Tau	OID00305	Neurology	RGMA	Q96B86	-0.1220398	0.262978
Tau	OID00981	Immune	NF2	P35240	-0.1216499	0.264518
Tau	OID00438	Cardiovascular	PSGL-1	Q14242	-0.1201445	0.267669
Tau	OID00302	Neurology	PRTG	Q2VWP7	-0.1172255	0.282416
Tau	OID00509	Inflammation	FGF-5	P12034	0.11653438	0.282417
Tau	OID00368	Neurology	IL12	P29460,P29459	0.11700197	0.283341
Tau	OID00324	Neurology	gal-8	O00214	-0.1160002	0.287512
Tau	OID00949	Immune	CLEC4C	Q8WTT0	-0.1133917	0.298562
Tau	OID00301	Neurology	NCAN	O14594	0.11284573	0.30091
Tau	OID00493	Inflammation	IL-1 alpha	P01583	-0.1119009	0.302123
Tau	OID00954	Immune	FGF2	P09038	-0.1124228	0.302737
Tau	OID00522	Inflammation	HGF	P14210	0.11174706	0.302793
Tau	OID00551	Inflammation	CCL25	O15444	0.11123285	0.305037
Tau	OID00946	Immune	EDAR	Q9UNE0	-0.1113818	0.307264
Tau	OID00992	Immune	CXADR	P78310	0.11046293	0.311297
Tau	OID00416	Cardiovascular	SPON2	Q9BUD6	0.10959364	0.312263
Tau	OID00982	Immune	PLXNA4	Q9HCM2	-0.1092203	0.316805

Tau	OID00979	Immune	BIRC2	Q13490	-0.1089557	0.317986
Tau	OID00506	Inflammation	TNFSF14	O43557	-0.108284	0.318116
Tau	OID00298	Neurology	NBL1	P41271	-0.108439	0.3203
Tau	OID00461	Cardiovascular	TNFRSF13B	O14836	0.10726498	0.322719
Tau	OID00375	Neurology	TN-R	Q92752	-0.1071564	0.326091
Tau	OID00374	Neurology	MANF	P55145	-0.1058542	0.332039
Tau	OID00502	Inflammation	SLAMF1	Q13291	0.10514713	0.332419
Tau	OID00558	Inflammation	STAMBP	O95630	-0.1051147	0.33257
Tau	OID00313	Neurology	EPHB6	O15197	0.10544888	0.333904
Tau	OID00940	Immune	IRAK4	Q9NWZ3	-0.1046715	0.3375
Tau	OID00487	Inflammation	AXIN1	O15169	-0.1039423	0.33802
Tau	OID00983	Immune	SH2B3	Q9UQQ2	-0.1043967	0.338776
Tau	OID00972	Immune	EGLN1	Q9GZT9	0.10376748	0.341712
Tau	OID00536	Inflammation	4E-BP1	Q13541	-0.1008704	0.352567
Tau	OID00433	Cardiovascular	XCL1	P47992	0.09967814	0.358316
Tau	OID00462	Cardiovascular	TGM2	P21980	-0.0995585	0.358896
Tau	OID01012	Immune	PADI2	Q9Y2J8	0.09874079	0.365731
Tau	OID00369	Neurology	Dkk-4	Q9UBT3	0.09862271	0.366308
Tau	OID00413	Cardiovascular	SOD2	P04179	-0.0965265	0.37379
Tau	OID01011	Immune	DAPP1	Q9UN19	-0.0960942	0.378783
Tau	OID00978	Immune	PTH1R	Q03431	0.09433216	0.387627
Tau	OID00561	Inflammation	TNFB	P01374	0.09327678	0.390164
Tau	OID00499	Inflammation	CD6	P30203	-0.093265	0.390225
Tau	OID00463	Cardiovascular	LEP	P41159	0.09250561	0.394112
Tau	OID01024	Immune	IL5	P05113	-0.0926664	0.396101
Tau	OID00446	Cardiovascular	LPL	P06858	0.09210616	0.396166
Tau	OID00964	Immune	TRIM21	P19474	0.09247503	0.397081
Tau	OID00372	Neurology	NTRK3	Q16288	-0.0922777	0.398093
Tau	OID00366	Neurology	CLM-1	Q8TDQ1	0.09210231	0.398995
Tau	OID00488	Inflammation	TRAIL	P50591	-0.0900024	0.407088
Tau	OID00553	Inflammation	TNFRSF9	Q07011	0.0892445	0.411066
Tau	OID00424	Cardiovascular	DECR1	Q16698	-0.088891	0.412929
Tau	OID00958	Immune	DCTN1	Q14203	-0.0886943	0.416744
Tau	OID01020	Immune	TANK	Q92844	-0.0884147	0.41822

Tau	OID00456	Cardiovascular	MMP12	P39900	0.08538449	0.431673
Tau	OID00948	Immune	DGKZ	Q13574	-0.0841416	0.441154
Tau	OID00388	Cardiovascular	SRC	P12931	0.08330857	0.442994
Tau	OID00538	Inflammation	SIRT2	Q8IXJ6	-0.0831249	0.444004
Tau	OID00336	Neurology	SCARA5	Q6ZMJ2	-0.0835394	0.444442
Tau	OID00321	Neurology	RSPO1	Q2MKA7	0.08340629	0.44517
Tau	OID00426	Cardiovascular	KIM1	Q96D42	0.08257184	0.447052
Tau	OID00977	Immune	CD28	P10747	0.08295872	0.447625
Tau	OID00378	Neurology	KYNU	Q16719	-0.0827551	0.448745
Tau	OID00491	Inflammation	CST5	P28325	0.08215954	0.449332
Tau	OID00457	Cardiovascular	ACE2	Q9BYF1	-0.0817468	0.45162
Tau	OID01010	Immune	IFNLR1	Q8IU57	0.07983109	0.464989
Tau	OID00333	Neurology	GFR-alpha-1	P56159	0.07906264	0.46931
Tau	OID00498	Inflammation	CCL4	P13236	-0.0770865	0.477907
Tau	OID00996	Immune	BACH1	O14867	0.07574975	0.488189
Tau	OID00440	Cardiovascular	CCL3	P10147	0.0747551	0.49136
Tau	OID00942	Immune	HCLS1	P14317	-0.074367	0.496186
Tau	OID00359	Neurology	JAM-B	P57087	0.07306104	0.503802
Tau	OID00343	Neurology	GDNFR-alpha-3	O60609	-0.0729257	0.504594
Tau	OID00314	Neurology	RGMB	Q6NW40	0.07284803	0.50505
Tau	OID00316	Neurology	CNTN5	O94779	-0.0727318	0.505731
Tau	OID00997	Immune	PIK3AP1	Q6ZUJ8	-0.0711532	0.515035
Tau	OID00968	Immune	ITM2A	O43736	0.07094771	0.516252
Tau	OID00335	Neurology	Beta-NGF	P01138	0.07091011	0.516475
Tau	OID00936	Immune	PPP1R9B	Q96SB3	-0.0703205	0.519978
Tau	OID00288	Neurology	NRP2	O60462	-0.0688256	0.52891
Tau	OID00303	Neurology	ROBO2	Q9HCK4	-0.0662204	0.54466
Tau	OID00991	Immune	TREM1	Q9NP99	0.06553406	0.548846
Tau	OID00458	Cardiovascular	PD-L2	Q9BQ51	0.06494443	0.550086
Tau	OID00486	Inflammation	CXCL11	O14625	-0.0637205	0.557644
Tau	OID00405	Cardiovascular	LOX-1	P78380	0.0630413	0.561859
Tau	OID00984	Immune	FCRL3	Q96P31	0.06306403	0.564042
Tau	OID00339	Neurology	GZMA	P12544	0.06239579	0.568187
Tau	OID00976	Immune	EIF4G1	Q04637	-0.0612813	0.575131

Tau	OID00541	Inflammation	EN-RAGE	P80511	0.06015798	0.57992
Tau	OID00408	Cardiovascular	SCF	P21583	0.05991035	0.581483
Tau	OID00395	Cardiovascular	PAR-1	P25116	-0.0587636	0.588749
Tau	OID00542	Inflammation	CD40	P25942	0.05800835	0.593556
Tau	OID00966	Immune	NTF4	P34130	0.05778051	0.597197
Tau	OID00304	Neurology	CRTAM	O95727	0.05633457	0.60642
Tau	OID00963	Immune	TRAF2	Q12933	-0.0561478	0.607616
Tau	OID00557	Inflammation	ST1A1	P50225	-0.0552378	0.611341
Tau	OID00403	Cardiovascular	IL-17D	Q8TAD2	-0.0548088	0.614116
Tau	OID00494	Inflammation	OSM	P13725	0.05457141	0.615653
Tau	OID00467	Cardiovascular	NEMO	Q9Y6K9	-0.0539825	0.619475
Tau	OID00373	Neurology	LAIR-2	Q6ISS4	0.0534673	0.624891
Tau	OID00337	Neurology	CD200	P41217	0.05216344	0.633369
Tau	OID00990	Immune	MGMT	P16455	-0.0507862	0.642375
Tau	OID00425	Cardiovascular	MERTK	Q12866	0.04952161	0.648746
Tau	OID00500	Inflammation	SCF	P21583	0.04752468	0.662027
Tau	OID00951	Immune	CLEC4A	Q9UMR7	-0.0472672	0.665621
Tau	OID00292	Neurology	UNC5C	O95185	0.04467919	0.682922
Tau	OID00319	Neurology	ADAM 23	O75077	0.04425643	0.685764
Tau	OID00955	Immune	PRDX5	P30044	-0.0432188	0.692758
Tau	OID00358	Neurology	DDR1	Q08345	0.04255299	0.69726
Tau	OID01022	Immune	KPNA1	P52294	-0.0421378	0.700072
Tau	OID00393	Cardiovascular	IDUA	P35475	0.04133346	0.703857
Tau	OID01021	Immune	ITGA11	Q9UKX5	-0.0414188	0.704952
Tau	OID00460	Cardiovascular	hOSCAR	Q8IYS5	0.04091057	0.706748
Tau	OID00421	Cardiovascular	PAPPA	Q13219	-0.0408428	0.707212
Tau	OID00351	Neurology	BMP-4	P12644	-0.0406787	0.709988
Tau	OID00953	Immune	PRDX3	P30048	-0.0401223	0.713782
Tau	OID00392	Cardiovascular	STK4	Q13043	-0.0389971	0.719883
Tau	OID00310	Neurology	MSR1	P21757	0.03749757	0.731771
Tau	OID00325	Neurology	BCAN	Q96GW7	-0.0373342	0.732896
Tau	OID00362	Neurology	NAAA	Q02083	0.03685245	0.736216
Tau	OID00357	Neurology	CDH6	P55285	-0.0367403	0.736989
Tau	OID00956	Immune	DPP10	Q8N608	-0.0336045	0.75872

Tau	OID00347	Neurology	FLRT2	O43155	-0.0330838	0.762347
Tau	OID00973	Immune	NFATC3	Q12968	0.03281956	0.76419
Tau	OID00442	Cardiovascular	IgG Fc receptor II-b	P31994	-0.0319498	0.768932
Tau	OID00937	Immune	GLB1	P16278	-0.0321288	0.769013
Tau	OID00950	Immune	IRAK1	P51617	-0.0312525	0.775144
Tau	OID00943	Immune	CNTNAP2	Q9UHC6	0.03103758	0.77665
Tau	OID00365	Neurology	TNFRSF21	O75509	-0.0298418	0.785044
Tau	OID00505	Inflammation	CCL11	P51671	-0.0294722	0.786405
Tau	OID00941	Immune	TPSAB1	Q15661	0.02951514	0.787341
Tau	OID00560	Inflammation	ADA	P00813	0.02712908	0.803029
Tau	OID00356	Neurology	CTSC	P53634	0.02651788	0.808501
Tau	OID00452	Cardiovascular	THPO	P40225	-0.0256137	0.813827
Tau	OID00354	Neurology	IL-5R-alpha	Q01344	0.02350451	0.829914
Tau	OID00317	Neurology	ADAM 22	Q9P0K1	-0.0228121	0.834853
Tau	OID00453	Cardiovascular	MARCO	Q9UEW3	-0.0210869	0.846284
Tau	OID00377	Neurology	Nr-CAM	Q92823	0.01937176	0.859482
Tau	OID00994	Immune	SRPK2	P78362	-0.0188999	0.862871
Tau	OID00398	Cardiovascular	TIE2	Q02763	-0.0177969	0.870038
Tau	OID00361	Neurology	N-CDase	Q9NR71	-0.0177963	0.870807
Tau	OID00451	Cardiovascular	FABP2	P12104	-0.0170619	0.875361
Tau	OID00974	Immune	LY75	O60449	0.01700532	0.876503
Tau	OID00945	Immune	IRF9	Q00978	0.01689337	0.877309
Tau	OID00470	Cardiovascular	HAOX1	Q9UJM8	-0.0158616	0.884066
Tau	OID01002	Immune	SH2D1A	O60880	0.01561663	0.886518
Tau	OID00970	Immune	CCL11	P51671	-0.0152497	0.889167
Tau	OID00962	Immune	FXYD5	Q96DB9	-0.0131785	0.904143
Tau	OID00939	Immune	ZBTB16	Q05516	0.01306346	0.904976
Tau	OID00294	Neurology	Siglec-9	Q9Y336	-0.0118022	0.914114
Tau	OID00952	Immune	PRDX1	Q06830	0.01146558	0.916554
Tau	OID00342	Neurology	SCARF2	Q96GP6	-0.0109766	0.920101
Tau	OID00455	Cardiovascular	BNP	P16860	-0.0105482	0.922753
Tau	OID00364	Neurology	PLXNB1	O43157	0.01028371	0.92513
Tau	OID00515	Inflammation	IL-10RB	Q08334	0.00766938	0.943794
Tau	OID00297	Neurology	SMOC2	Q9H3U7	0.00696885	0.949224

Tau	OID00531	Inflammation	CD5	P06127	0.00560494	0.958908
Tau	OID00447	Cardiovascular	PRSS8	Q16651	-0.0052559	0.961465
Tau	OID00550	Inflammation	CASP-8	Q14790	0.00463094	0.966044
Tau	OID00312	Neurology	sFRP-3	Q92765	-0.0040266	0.970649
Tau	OID00450	Cardiovascular	GDF-2	Q9UK05	-0.0038307	0.971909
Tau	OID00472	Inflammation	VEGFA	P15692	-0.0037264	0.972674
Tau	OID00489	Inflammation	IL-20RA	Q9UHF4	0.00311836	0.977131
Tau	OID00533	Inflammation	Flt3L	P49771	0.00294814	0.978379
Tau	OID00987	Immune	HEXIM1	O94992	-0.0016503	0.987968
Tau	OID00477	Inflammation	CD244	Q9BZW8	-0.0014444	0.989407
Tau	OID00475	Inflammation	GDNF	P39905	0.00133943	0.990176
Tau	OID01004	Immune	DFFA	O00273	-0.0012845	0.990635
Tau	OID00311	Neurology	Alpha-2-MRAP	P30533	-0.0010528	0.992324
Tau	OID00989	Immune	PRKCQ	Q04759	0.00085372	0.993776
GFAP	OID00455	Cardiovascular	BNP	P16860	0.50853006	4.94E-07
GFAP	OID00394	Cardiovascular	TNFRSF11A	Q9Y6Q6	0.47483406	3.37E-06
GFAP	OID00552	Inflammation	CX3CL1	P78423	0.47481626	3.37E-06
GFAP	OID01018	Immune	DDX58	O95786	0.46783903	5.57E-06
GFAP	OID00300	Neurology	SCARB2	Q14108	0.46496289	6.47E-06
GFAP	OID00399	Cardiovascular	TF	P13726	0.46236771	6.53E-06
GFAP	OID00969	Immune	HNMT	P50135	0.46202607	7.53E-06
GFAP	OID00985	Immune	CKAP4	Q07065	0.46157393	7.71E-06
GFAP	OID00384	Cardiovascular	PGF	P49763	0.45895295	7.79E-06
GFAP	OID00326	Neurology	LAYN	Q6UX15	0.45285375	1.20E-05
GFAP	OID00370	Neurology	EDA2R	Q9HAV5	0.44782447	1.54E-05
GFAP	OID00517	Inflammation	IL-18R1	Q13478	0.44455759	1.61E-05
GFAP	OID00346	Neurology	SKR3	P37023	0.44309815	1.93E-05
GFAP	OID00514	Inflammation	IL-15RA	Q13261	0.43690466	2.33E-05
GFAP	OID00320	Neurology	MATN3	O15232	0.43807979	2.46E-05
GFAP	OID00414	Cardiovascular	CTRC	Q99895	-0.4328589	2.82E-05
GFAP	OID00518	Inflammation	PD-L1	Q9NZQ7	0.42889411	3.40E-05
GFAP	OID00967	Immune	KRT19	P08727	0.4286352	3.83E-05
GFAP	OID00436	Cardiovascular	CEACAM8	P31997	0.42620561	3.86E-05
GFAP	OID00391	Cardiovascular	TNFRSF10A	O00220	0.42143937	4.80E-05

GFAP	OID00521	Inflammation	TRANCE	O14788	-0.4176861	5.69E-05
GFAP	OID00991	Immune	TREM1	Q9NP99	0.41693564	6.51E-05
GFAP	OID00542	Inflammation	CD40	P25942	0.41073626	7.76E-05
GFAP	OID00299	Neurology	EFNA4	P52798	0.41260648	7.89E-05
GFAP	OID00355	Neurology	PDGF-R-alpha	P16234	0.40867634	9.37E-05
GFAP	OID00434	Cardiovascular	IL16	Q14005	0.40578583	9.65E-05
GFAP	OID00363	Neurology	N2DL-2	Q9BZM5	0.40703747	0.000101
GFAP	OID01027	Immune	BTN3A2	P78410	0.40571081	0.000106
GFAP	OID00535	Inflammation	CXCL10	P02778	0.39842871	0.000132
GFAP	OID00296	Neurology	EZR	P15311	0.39970407	0.000137
GFAP	OID00307	Neurology	CPA2	P48052	-0.396153	0.00016
GFAP	OID00396	Cardiovascular	TRAIL-R2	O14763	0.39310499	0.000166
GFAP	OID00532	Inflammation	CCL3	P10147	0.39021615	0.000187
GFAP	OID00444	Cardiovascular	DCN	P07585	0.38680088	0.000215
GFAP	OID00412	Cardiovascular	RAGE	Q15109	0.38608533	0.000221
GFAP	OID00947	Immune	IL6	P05231	0.38630175	0.000239
GFAP	OID00409	Cardiovascular	IL18	Q14116	0.38397107	0.000241
GFAP	OID00369	Neurology	Dkk-4	Q9UBT3	0.38590867	0.000243
GFAP	OID00360	Neurology	CTSS	P25774	0.38304846	0.000272
GFAP	OID00367	Neurology	SPOCK1	Q08629	0.38277963	0.000275
GFAP	OID00471	Inflammation	IL8	P10145	0.37929187	0.000291
GFAP	OID00501	Inflammation	IL18	Q14116	0.37250246	0.000381
GFAP	OID00972	Immune	EGLN1	Q9GZT9	0.37401308	0.000389
GFAP	OID00562	Inflammation	CSF-1	P09603	0.3717912	0.000392
GFAP	OID01009	Immune	AREG	P15514	0.37312956	0.000403
GFAP	OID00479	Inflammation	OPG	O00300	0.37080672	0.000407
GFAP	OID00486	Inflammation	CXCL11	O14625	0.37020569	0.000417
GFAP	OID00440	Cardiovascular	CCL3	P10147	0.36860772	0.000443
GFAP	OID00995	Immune	KLRD1	Q13241	0.36938021	0.000465
GFAP	OID00432	Cardiovascular	HO-1	P09601	0.36702013	0.000471
GFAP	OID00321	Neurology	RSPO1	Q2MKA7	0.36898597	0.000472
GFAP	OID00315	Neurology	SIGLEC1	Q9BZZ2	0.3673809	0.000502
GFAP	OID05547	Inflammation	IFN-gamma	P01579	0.36501713	0.000509
GFAP	OID00405	Cardiovascular	LOX-1	P78380	0.36426495	0.000523

GFAP	OID00387	Cardiovascular	IL-4RA	P24394	0.36306027	0.000548
GFAP	OID00472	Inflammation	VEGFA	P15692	0.36222678	0.000566
GFAP	OID00406	Cardiovascular	Gal-9	O00182	0.36220668	0.000566
GFAP	OID00390	Cardiovascular	IL6	P05231	0.36138793	0.000584
GFAP	OID00295	Neurology	CLM-6	Q08708	0.36004155	0.000662
GFAP	OID00482	Inflammation	IL6	P05231	0.35601049	0.000714
GFAP	OID00402	Cardiovascular	IL-27	Q8NEV9,Q14213	0.35390765	0.000772
GFAP	OID00965	Immune	LILRB4	Q8NHJ6	0.35417112	0.000821
GFAP	OID00938	Immune	PSIP1	O75475	0.3488672	0.000995
GFAP	OID00524	Inflammation	IL-24	Q13007	0.34615698	0.001023
GFAP	OID00490	Inflammation	CXCL9	Q07325	0.34613401	0.001024
GFAP	OID00474	Inflammation	MCP-3	P80098	0.34352688	0.001124
GFAP	OID00993	Immune	IL10	P22301	0.34249595	0.001247
GFAP	OID00945	Immune	IRF9	Q00978	0.3423599	0.001253
GFAP	OID01015	Immune	LAMP3	Q9UQV4	0.33715718	0.001501
GFAP	OID05548	Inflammation	TNF	P01375	0.33394829	0.001572
GFAP	OID00541	Inflammation	EN-RAGE	P80511	0.33324158	0.001611
GFAP	OID00333	Neurology	GFR-alpha-1	P56159	0.3341656	0.001664
GFAP	OID00506	Inflammation	TNFSF14	O43557	0.32543313	0.0021
GFAP	OID00460	Cardiovascular	hOSCAR	Q8IYS5	0.32533868	0.002107
GFAP	OID00469	Cardiovascular	PARP-1	P09874	0.32518923	0.002117
GFAP	OID00381	Cardiovascular	ADM	P35318	0.32482198	0.002143
GFAP	OID00437	Cardiovascular	PTX3	P26022	0.32438484	0.002175
GFAP	OID00344	Neurology	PVR	P15151	0.32619019	0.002177
GFAP	OID00442	Cardiovascular	IgG Fc receptor II-b	P31994	0.32387655	0.002212
GFAP	OID00292	Neurology	UNC5C	O95185	0.32372667	0.002362
GFAP	OID00419	Cardiovascular	GLO1	Q04760	0.32176063	0.002373
GFAP	OID00426	Cardiovascular	KIM1	Q96D42	0.32163177	0.002384
GFAP	OID00378	Neurology	KYNU	Q16719	0.32128901	0.002559
GFAP	OID00431	Cardiovascular	PRELP	P51888	0.31877092	0.002619
GFAP	OID00418	Cardiovascular	FS	P19883	0.31297694	0.003162
GFAP	OID00494	Inflammation	OSM	P13725	0.31256008	0.003204
GFAP	OID00459	Cardiovascular	CTSL1	P07711	0.31255592	0.003205
GFAP	OID00513	Inflammation	CCL19	Q99731	0.3125376	0.003207

GFAP	OID00484	Inflammation	MCP-1	P13500	0.310026	0.003475
GFAP	OID01007	Immune	NCR1	O76036	0.31128318	0.00353
GFAP	OID00422	Cardiovascular	SERPINA12	Q8IW75	-0.3090457	0.003585
GFAP	OID00476	Inflammation	CDCP1	Q9H5V8	0.30774768	0.003735
GFAP	OID00560	Inflammation	ADA	P00813	0.30543912	0.004017
GFAP	OID00425	Cardiovascular	MERTK	Q12866	0.30280486	0.004361
GFAP	OID00287	Neurology	NMNAT1	Q9HAN9	0.30347328	0.004505
GFAP	OID00290	Neurology	CADM3	Q8N126	0.30216848	0.00469
GFAP	OID00364	Neurology	PLXNB1	O43157	0.30159344	0.004773
GFAP	OID00988	Immune	CLEC4D	Q8WXI8	0.30008248	0.004998
GFAP	OID00366	Neurology	CLM-1	Q8TDQ1	0.29870043	0.005213
GFAP	OID00313	Neurology	EPHB6	O15197	0.29795106	0.005332
GFAP	OID00403	Cardiovascular	IL-17D	Q8TAD2	0.29469275	0.005592
GFAP	OID00353	Neurology	MDGA1	Q8NFP4	-0.2960249	0.005651
GFAP	OID00528	Inflammation	IL10	P22301	0.29414319	0.005686
GFAP	OID00464	Cardiovascular	CA5A	P35218	0.29292543	0.005898
GFAP	OID00503	Inflammation	TGF-alpha	P01135	0.2917743	0.006105
GFAP	OID00340	Neurology	G-CSF	P09919	0.29321165	0.006147
GFAP	OID00354	Neurology	IL-5R-alpha	Q01344	0.29065403	0.006631
GFAP	OID00389	Cardiovascular	IL-1ra	P18510	0.28824565	0.006781
GFAP	OID00345	Neurology	TNFRSF12A	Q9NP84	0.28911315	0.006938
GFAP	OID00416	Cardiovascular	SPON2	Q9BUD6	0.28743581	0.006945
GFAP	OID00308	Neurology	CD38	P28907	0.28810304	0.007146
GFAP	OID00445	Cardiovascular	Dkk-1	O94907	0.2853909	0.007375
GFAP	OID00992	Immune	CXADR	P78310	0.28476253	0.007875
GFAP	OID00499	Inflammation	CD6	P30203	-0.281886	0.008167
GFAP	OID00318	Neurology	CLEC1B	Q9P126	0.28173245	0.008591
GFAP	OID00522	Inflammation	HGF	P14210	0.27995856	0.008633
GFAP	OID00328	Neurology	GDF-8	O14793	-0.2780505	0.009538
GFAP	OID00341	Neurology	DRAXIN	Q8NBI3	0.2765115	0.009961
GFAP	OID00996	Immune	BACH1	O14867	0.2762702	0.010028
GFAP	OID00309	Neurology	SMPD1	P17405	0.27540729	0.010273
GFAP	OID00527	Inflammation	MMP-10	P09238	0.2717014	0.010906
GFAP	OID01025	Immune	CD83	Q01151	0.27220722	0.011229

GFAP	OID00496	Inflammation	CXCL1	P09341	0.27009609	0.011404
GFAP	OID00498	Inflammation	CCL4	P13236	0.26586887	0.012812
GFAP	OID00964	Immune	TRIM21	P19474	0.2661589	0.013249
GFAP	OID00549	Inflammation	MCP-2	P80075	0.26369033	0.013594
GFAP	OID00961	Immune	GALNT3	Q14435	0.26487349	0.013717
GFAP	OID01017	Immune	CLEC6A	Q6EIG7	0.26452269	0.013847
GFAP	OID00944	Immune	CLEC4G	Q6UXB4	0.26279548	0.014504
GFAP	OID00288	Neurology	NRP2	O60462	0.26157318	0.014985
GFAP	OID00485	Inflammation	IL-17A	Q16552	0.25904287	0.015403
GFAP	OID00428	Cardiovascular	TM	P07204	0.25776899	0.015934
GFAP	OID01005	Immune	DCBLD2	Q96PD2	0.25673461	0.017025
GFAP	OID01026	Immune	ITGB6	P18564	0.2561922	0.017268
GFAP	OID00361	Neurology	N-CDase	Q9NR71	-0.2559116	0.017395
GFAP	OID00502	Inflammation	SLAMF1	Q13291	0.25415229	0.017528
GFAP	OID00348	Neurology	CPM	P14384	-0.2550317	0.017798
GFAP	OID00429	Cardiovascular	VSIG2	Q96IQ7	0.25201863	0.018532
GFAP	OID00515	Inflammation	IL-10RB	Q08334	0.25153093	0.018768
GFAP	OID00957	Immune	TRIM5	Q9C035	0.2516288	0.019434
GFAP	OID00509	Inflammation	FGF-5	P12034	0.24972473	0.019665
GFAP	OID00404	Cardiovascular	CXCL1	P09341	0.24966297	0.019696
GFAP	OID01010	Immune	IFNLR1	Q8IU57	0.2509557	0.019772
GFAP	OID01016	Immune	CLEC7A	Q9BXN2	0.24689769	0.021921
GFAP	OID00310	Neurology	MSR1	P21757	0.24557354	0.022664
GFAP	OID00465	Cardiovascular	HSP 27	P04792	0.24174046	0.024086
GFAP	OID00411	Cardiovascular	PIgR	P01833	0.24052263	0.024829
GFAP	OID00971	Immune	MILR1	Q7Z6M3	0.24079281	0.025528
GFAP	OID00330	Neurology	WFIKKN1	Q96NZ8	-0.2387352	0.026852
GFAP	OID00311	Neurology	Alpha-2-MRAP	P30533	0.23689151	0.028088
GFAP	OID01004	Immune	DFFA	O00273	0.23636725	0.028448
GFAP	OID00511	Inflammation	LIF-R	P42702	0.2344357	0.028845
GFAP	OID00507	Inflammation	FGF-23	Q9GZV9	0.23349951	0.029509
GFAP	OID00441	Cardiovascular	MMP7	P09237	0.23088	0.031435
GFAP	OID00435	Cardiovascular	SORT1	Q99523	0.23061951	0.031633
GFAP	OID00356	Neurology	CTSC	P53634	0.2297704	0.033322

GFAP	OID01023	Immune	LAG3	P18627	0.22962002	0.033441
GFAP	OID00550	Inflammation	CASP-8	Q14790	0.22718877	0.034329
GFAP	OID01019	Immune	IL12RB1	P42701	0.22675944	0.035769
GFAP	OID00556	Inflammation	CCL20	P78556	0.22353347	0.037412
GFAP	OID00433	Cardiovascular	XCL1	P47992	0.21620213	0.044295
GFAP	OID00483	Inflammation	IL-17C	Q9P0M4	0.21484544	0.045677
GFAP	OID00952	Immune	PRDX1	Q06830	0.21600602	0.045772
GFAP	OID00415	Cardiovascular	FGF-23	Q9GZV9	0.2146438	0.045886
GFAP	OID01008	Immune	CXCL12	P48061	0.21417496	0.047686
GFAP	OID00478	Inflammation	IL7	P13232	0.21069555	0.050129
GFAP	OID00939	Immune	ZBTB16	Q05516	0.2100227	0.052273
GFAP	OID05024	Neurology	MAPT	P10636	0.20702217	0.055809
GFAP	OID01213	Inflammation	DNER	Q8NFT8	-0.2031963	0.059077
GFAP	OID00304	Neurology	CRTAM	O95727	0.20371857	0.059925
GFAP	OID00350	Neurology	GCP5	P78333	-0.2036043	0.060072
GFAP	OID00324	Neurology	gal-8	O00214	0.20066427	0.063948
GFAP	OID00987	Immune	HEXIM1	O94992	0.1981169	0.067468
GFAP	OID00352	Neurology	FcRL2	Q96LA5	0.19796487	0.067683
GFAP	OID00401	Cardiovascular	PDGF subunit B	P01127	0.19304357	0.073223
GFAP	OID00457	Cardiovascular	ACE2	Q9BYF1	0.19304273	0.073224
GFAP	OID00530	Inflammation	CCL23	P55773	0.19283006	0.073547
GFAP	OID00963	Immune	TRAF2	Q12933	0.19377426	0.073828
GFAP	OID00458	Cardiovascular	PD-L2	Q9BQ51	0.19208428	0.07469
GFAP	OID00329	Neurology	THY 1	P04216	0.19268444	0.075497
GFAP	OID00955	Immune	PRDX5	P30044	0.19168558	0.077054
GFAP	OID00942	Immune	HCLS1	P14317	0.18939456	0.080721
GFAP	OID00303	Neurology	ROBO2	Q9HCK4	-0.1874745	0.083901
GFAP	OID00461	Cardiovascular	TNFRSF13B	O14836	0.18488472	0.086463
GFAP	OID01014	Immune	MASP1	P48740	-0.1857204	0.086892
GFAP	OID00410	Cardiovascular	FGF-21	Q9NSA1	0.18346572	0.088948
GFAP	OID00438	Cardiovascular	PSGL-1	Q14242	-0.1815046	0.092475
GFAP	OID05124	Inflammation	CD8A	P01732	-0.1810489	0.093311
GFAP	OID00447	Cardiovascular	PRSS8	Q16651	0.18100821	0.093386
GFAP	OID00359	Neurology	JAM-B	P57087	0.17937639	0.09842

GFAP	OID00467	Cardiovascular	NEMO	Q9Y6K9	0.17785441	0.099334
GFAP	OID00335	Neurology	Beta-NGF	P01138	0.17785195	0.101362
GFAP	OID00977	Immune	CD28	P10747	-0.1738575	0.109396
GFAP	OID00512	Inflammation	FGF-21	Q9NSA1	0.17107247	0.11313
GFAP	OID00951	Immune	CLEC4A	Q9UMR7	-0.1707897	0.115895
GFAP	OID00941	Immune	TPSAB1	Q15661	0.17060829	0.116289
GFAP	OID00950	Immune	IRAK1	P51617	0.16606714	0.126478
GFAP	OID00383	Cardiovascular	SLAMF7	Q9NQ25	0.16467717	0.127455
GFAP	OID00480	Inflammation	LAP TGF-beta-1	P01137	0.16389732	0.129292
GFAP	OID00966	Immune	NTF4	P34130	0.16441106	0.130359
GFAP	OID00376	Neurology	CD200R1	Q8TD46	-0.1639397	0.13148
GFAP	OID00314	Neurology	RGMB	Q6NW40	0.16280785	0.134202
GFAP	OID00997	Immune	PIK3AP1	Q6ZUJ8	0.16233014	0.135363
GFAP	OID00342	Neurology	SCARF2	Q96GP6	0.15965291	0.142014
GFAP	OID00439	Cardiovascular	CCL17	Q92583	-0.1572213	0.145859
GFAP	OID00536	Inflammation	4E-BP1	Q13541	0.15714226	0.146065
GFAP	OID00555	Inflammation	TWEAK	O43508	-0.1561616	0.14863
GFAP	OID00380	Cardiovascular	ANGPT1	Q15389	0.15314942	0.15672
GFAP	OID00450	Cardiovascular	GDF-2	Q9UK05	-0.1530847	0.156897
GFAP	OID00421	Cardiovascular	PAPPA	Q13219	0.15178365	0.160494
GFAP	OID00293	Neurology	VWC2	Q2TAL6	0.1497909	0.168649
GFAP	OID00393	Cardiovascular	IDUA	P35475	-0.1488677	0.168777
GFAP	OID00362	Neurology	NAAA	Q02083	-0.1477522	0.174587
GFAP	OID00982	Immune	PLXNA4	Q9HCM2	0.14774848	0.174598
GFAP	OID00547	Inflammation	LIF	P15018	0.1439667	0.183402
GFAP	OID00523	Inflammation	IL-12B	P29460	0.1422694	0.188676
GFAP	OID00553	Inflammation	TNFRSF9	Q07011	0.14170009	0.19047
GFAP	OID00386	Cardiovascular	BOC	Q9BWV1	-0.1416413	0.190655
GFAP	OID00994	Immune	SRPK2	P78362	0.14216841	0.191632
GFAP	OID00976	Immune	EIF4G1	Q04637	0.14142393	0.193992
GFAP	OID00962	Immune	FXYD5	Q96DB9	0.14025655	0.197735
GFAP	OID00385	Cardiovascular	ADAM-TS13	Q76LX8	-0.1371543	0.205233
GFAP	OID00368	Neurology	IL12	P29460,P29459	0.13784034	0.205647
GFAP	OID00466	Cardiovascular	CD4	P01730	0.13515406	0.211981

GFAP	OID00339	Neurology	GZMA	P12544	0.13384241	0.21923
GFAP	OID00974	Immune	LY75	O60449	-0.1325841	0.223634
GFAP	OID00534	Inflammation	CXCL6	P80162	0.1312347	0.225658
GFAP	OID00488	Inflammation	TRAIL	P50591	-0.1311384	0.226002
GFAP	OID00302	Neurology	PRTG	Q2VWP7	-0.1308782	0.229701
GFAP	OID00481	Inflammation	uPA	P00749	0.13008262	0.229793
GFAP	OID00531	Inflammation	CD5	P06127	-0.1289609	0.233871
GFAP	OID00316	Neurology	CNTN5	O94779	-0.1278143	0.240886
GFAP	OID00351	Neurology	BMP-4	P12644	-0.1273399	0.242652
GFAP	OID00538	Inflammation	SIRT2	Q8IXJ6	0.12586378	0.245388
GFAP	OID00943	Immune	CNTNAP2	Q9UHC6	0.12627244	0.246655
GFAP	OID00984	Immune	FCRL3	Q96P31	0.12624684	0.246752
GFAP	OID00417	Cardiovascular	GH	P01241	0.12430234	0.25134
GFAP	OID00493	Inflammation	IL-1 alpha	P01583	-0.1230548	0.256166
GFAP	OID00388	Cardiovascular	SRC	P12931	0.12148247	0.262338
GFAP	OID00452	Cardiovascular	THPO	P40225	0.121083	0.263922
GFAP	OID00371	Neurology	LAT	O43561	0.1193355	0.273782
GFAP	OID00937	Immune	GLB1	P16278	0.11859754	0.276781
GFAP	OID00338	Neurology	NTRK2	Q16620	-0.1183875	0.277639
GFAP	OID00413	Cardiovascular	SOD2	P04179	0.11767287	0.277709
GFAP	OID00973	Immune	NFATC3	Q12968	0.11730027	0.282107
GFAP	OID01021	Immune	ITGA11	Q9UKX5	-0.1165217	0.285336
GFAP	OID00953	Immune	PRDX3	P30048	0.11354519	0.297905
GFAP	OID00297	Neurology	SMOC2	Q9H3U7	0.11058056	0.310779
GFAP	OID00424	Cardiovascular	DECR1	Q16698	0.10968636	0.311852
GFAP	OID00468	Cardiovascular	VEGFD	O43915	0.10924968	0.313794
GFAP	OID00561	Inflammation	TNFB	P01374	-0.1069313	0.324235
GFAP	OID00408	Cardiovascular	SCF	P21583	-0.1046209	0.334858
GFAP	OID00491	Inflammation	CST5	P28325	0.10458554	0.335023
GFAP	OID00462	Cardiovascular	TGM2	P21980	0.10391183	0.338162
GFAP	OID00301	Neurology	NCAN	O14594	0.10392659	0.340968
GFAP	OID00500	Inflammation	SCF	P21583	-0.1019219	0.347544
GFAP	OID00365	Neurology	TNFRSF21	O75509	0.10247431	0.347794
GFAP	OID00958	Immune	DCTN1	Q14203	0.10208429	0.349642

GFAP	OID01003	Immune	ICA1	Q05084	0.10175608	0.351201
GFAP	OID00430	Cardiovascular	AMBP	P02760	0.10115458	0.351205
GFAP	OID00979	Immune	BIRC2	Q13490	0.09926708	0.363169
GFAP	OID01024	Immune	IL5	P05113	-0.0984788	0.367011
GFAP	OID00374	Neurology	MANF	P55145	0.09694317	0.374566
GFAP	OID00558	Inflammation	STAMBP	O95630	0.09513202	0.380765
GFAP	OID00505	Inflammation	CCL11	P51671	0.09451167	0.383892
GFAP	OID00551	Inflammation	CCL25	O15444	0.09441554	0.384378
GFAP	OID00954	Immune	FGF2	P09038	0.09381504	0.390246
GFAP	OID00960	Immune	CDSN	Q15517	0.09126203	0.403329
GFAP	OID00398	Cardiovascular	TIE2	Q02763	-0.0869651	0.423164
GFAP	OID00427	Cardiovascular	THBS2	P35442	0.08624905	0.427007
GFAP	OID00325	Neurology	BCAN	Q96GW7	0.08602546	0.430957
GFAP	OID00337	Neurology	CD200	P41217	0.08579482	0.432198
GFAP	OID00331	Neurology	TMPRSS5	Q9H3S3	-0.085338	0.434662
GFAP	OID00294	Neurology	Siglec-9	Q9Y336	0.08514692	0.435695
GFAP	OID00948	Immune	DGKZ	Q13574	0.08508277	0.436043
GFAP	OID00554	Inflammation	NT-3	P20783	-0.0837414	0.44062
GFAP	OID00334	Neurology	GM-CSF-R-alpha	P15509	0.08393152	0.442299
GFAP	OID00298	Neurology	NBL1	P41271	0.08277796	0.448619
GFAP	OID00456	Cardiovascular	MMP12	P39900	-0.0817965	0.451345
GFAP	OID00397	Cardiovascular	PRSS27	Q9BQR3	-0.0813315	0.45393
GFAP	OID00382	Cardiovascular	CD40-L	P29965	0.08085434	0.456591
GFAP	OID00375	Neurology	TN-R	Q92752	0.07846949	0.472661
GFAP	OID00510	Inflammation	MMP-1	P03956	0.07671903	0.480014
GFAP	OID00949	Immune	CLEC4C	Q8WTT0	-0.0771289	0.480282
GFAP	OID00423	Cardiovascular	REN	P00797	0.07663971	0.48047
GFAP	OID00305	Neurology	RGMA	Q96B86	-0.0759688	0.486929
GFAP	OID00332	Neurology	CDH3	P22223	0.074425	0.495849
GFAP	OID00557	Inflammation	ST1A1	P50225	0.07374941	0.497224
GFAP	OID00336	Neurology	SCARA5	Q6ZMJ2	0.07312018	0.503456
GFAP	OID00970	Immune	CCL11	P51671	0.07232591	0.508115
GFAP	OID00446	Cardiovascular	LPL	P06858	-0.0716685	0.509472
GFAP	OID00312	Neurology	sFRP-3	Q92765	0.07208236	0.509548

GFAP	OID00379	Cardiovascular	BMP-6	P22004	0.07151681	0.510371
GFAP	OID00392	Cardiovascular	STK4	Q13043	0.07034589	0.517337
GFAP	OID00420	Cardiovascular	CD84	Q9UIB8	0.06905818	0.525054
GFAP	OID00508	Inflammation	IL-10RA	Q13651	-0.067365	0.535287
GFAP	OID00323	Neurology	LXN	Q9BS40	0.06700233	0.539909
GFAP	OID00306	Neurology	PLXNB3	Q9ULL4	0.0668411	0.540887
GFAP	OID00504	Inflammation	MCP-4	Q99616	0.06499841	0.549754
GFAP	OID00990	Immune	MGMT	P16455	0.06524218	0.550632
GFAP	OID00936	Immune	PPP1R9B	Q96SB3	0.0644689	0.555375
GFAP	OID00470	Cardiovascular	HAOX1	Q9UJM8	0.06132518	0.572576
GFAP	OID00463	Cardiovascular	LEP	P41159	-0.0602683	0.579224
GFAP	OID00448	Cardiovascular	AGRP	O00253	0.06008484	0.580381
GFAP	OID01002	Immune	SH2D1A	O60880	0.05811184	0.595093
GFAP	OID00956	Immune	DPP10	Q8N608	-0.0566552	0.60437
GFAP	OID01013	Immune	SIT1	Q9Y3P8	-0.0558117	0.609771
GFAP	OID00477	Inflammation	CD244	Q9BZW8	-0.0553673	0.610505
GFAP	OID01000	Immune	ARNT	P27540	0.05525419	0.613352
GFAP	OID00978	Immune	PTH1R	Q03431	-0.0538669	0.622303
GFAP	OID00533	Inflammation	Flt3L	P49771	0.05278768	0.627259
GFAP	OID00443	Cardiovascular	ITGB1BP2	Q9UKP3	0.05162388	0.634881
GFAP	OID00980	Immune	HSD11B1	P28845	-0.0512232	0.639512
GFAP	OID00373	Neurology	LAIR-2	Q6ISS4	0.05075645	0.642571
GFAP	OID00487	Inflammation	AXIN1	O15169	0.04980015	0.646902
GFAP	OID00343	Neurology	GDNFR-alpha-3	O60609	-0.0491117	0.653396
GFAP	OID01006	Immune	FCRL6	Q6DN72	0.04852607	0.657268
GFAP	OID00940	Immune	IRAK4	Q9NWZ3	0.047721	0.662605
GFAP	OID01020	Immune	TANK	Q92844	0.04723011	0.665868
GFAP	OID00449	Cardiovascular	HB-EGF	Q99075	0.0467857	0.666969
GFAP	OID00526	Inflammation	ARTN	Q5T4W7	-0.0463176	0.670107
GFAP	OID00400	Cardiovascular	IL1RL2	Q9HB29	0.04586055	0.673176
GFAP	OID00377	Neurology	Nr-CAM	Q92823	0.04486387	0.681682
GFAP	OID00968	Immune	ITM2A	O43736	0.04407638	0.686976
GFAP	OID00989	Immune	PRKCQ	Q04759	0.04404454	0.68719
GFAP	OID00999	Immune	STC1	P52823	0.04322383	0.692724

GFAP	OID01012	Immune	PADI2	Q9Y2J8	0.04283227	0.69537
GFAP	OID00349	Neurology	CLEC10A	Q8IUN9	0.04263352	0.696715
GFAP	OID00983	Immune	SH2B3	Q9UQQ2	0.04025655	0.712866
GFAP	OID00998	Immune	SPRY2	O43597	0.03707304	0.734695
GFAP	OID00959	Immune	ITGA6	P23229	-0.0355709	0.74507
GFAP	OID00451	Cardiovascular	FABP2	P12104	-0.0342709	0.752665
GFAP	OID00520	Inflammation	CXCL5	P42830	0.03401527	0.754452
GFAP	OID01001	Immune	FAM3B	P58499	-0.0326431	0.765421
GFAP	OID00372	Neurology	NTRK3	Q16288	-0.0320374	0.769652
GFAP	OID00946	Immune	EDAR	Q9UNE0	-0.0318712	0.770814
GFAP	OID01022	Immune	KPNA1	P52294	0.03114163	0.775921
GFAP	OID00357	Neurology	CDH6	P55285	-0.0302321	0.782301
GFAP	OID00347	Neurology	FLRT2	O43155	0.02842339	0.795031
GFAP	OID00317	Neurology	ADAM 22	Q9P0K1	0.02711393	0.804281
GFAP	OID00981	Immune	NF2	P35240	0.0261245	0.811288
GFAP	OID00291	Neurology	GDNF	P39905	-0.022605	0.836331
GFAP	OID00319	Neurology	ADAM 23	O75077	0.02160429	0.843482
GFAP	OID00475	Inflammation	GDNF	P39905	0.01874466	0.863182
GFAP	OID00395	Cardiovascular	PAR-1	P25116	0.01835045	0.866033
GFAP	OID00545	Inflammation	FGF-19	O95750	-0.0167407	0.877689
GFAP	OID01011	Immune	DAPP1	Q9UN19	0.01669467	0.878741
GFAP	OID00453	Cardiovascular	MARCO	Q9UEW3	0.01373756	0.899504
GFAP	OID00327	Neurology	NEP	P08473	-0.0124493	0.909424
GFAP	OID00489	Inflammation	IL-20RA	Q9UHF4	-0.0087645	0.935785
GFAP	OID00407	Cardiovascular	GIF	P27352	-0.0080325	0.941138
GFAP	OID00539	Inflammation	CCL28	Q9NRJ3	0.00774695	0.943227
GFAP	OID00322	Neurology	HAGH	Q16775	0.00680223	0.950436
GFAP	OID00358	Neurology	DDR1	Q08345	0.00559164	0.959249
GFAP	OID00454	Cardiovascular	GT	P51161	0.00334867	0.975443
